# Discovery of *Trypanosoma brucei* inhibitors enabled by a unified synthesis of diverse sulfonyl fluorides

**DOI:** 10.1038/s42004-024-01327-8

**Published:** 2024-10-19

**Authors:** Brian S. Mantilla, Jack S. White, William R. T. Mosedale, Andrew Gomm, Adam Nelson, Terry K. Smith, Megan H. Wright

**Affiliations:** 1 School of Chemistry and Astbury Centre for Structural Molecular Biology, University of Leeds, Leeds, LS2 9JT UK; 2 Schools of Biology and Chemistry, Biomedical Sciences Research Complex, University of St Andrews, St Andrews, KY16 9ST UK

**Keywords:** Diversity-oriented synthesis, Target identification, Chemical tools, Proteomics

## Abstract

Sets of electrophilic probes are generally prepared using a narrow toolkit of robust reactions, which tends to limit both their structural and functional diversity. A unified synthesis of skeletally-diverse sulfonyl fluorides was developed that relied upon photoredox-catalysed dehydrogenative couplings between hetaryl sulfonyl fluorides and hydrogen donor building blocks. A set of 32 diverse probes was prepared, and then screened against *Trypanosoma brucei*. Four of the probes were found to have sub-micromolar anti-trypanosomal activity. A chemical proteomic approach, harnessing an alkynylated analogue and broad-spectrum fluorophosphonate tools, provided insights into the observed anti-trypanosomal activity, which likely stems from covalent modification of multiple protein targets. It is envisaged that the unified diversity-oriented approach may enable the discovery of electrophilic probes that have value in the elucidation of biological and biomedical mechanisms.

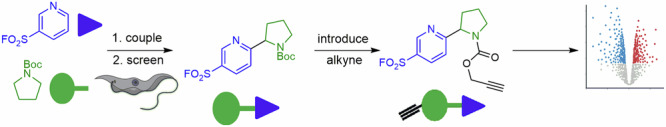

## Abstract (web summary)

Electrophilic bioactive compounds are useful chemical tools for identifying and modulating protein targets through reaction with nucleophilic amino acid side chain residues. Here, the authors report a modular synthesis of electrophilic sulfonyl fluoride probes, and evaluate their anti-trypanosomal activity using a chemoproteomics approach

## Introduction

Electrophilic bioactive compounds can serve as useful chemical tools for elucidating biological mechanisms^1,2^. Within this context, electrophilic bioactive molecules that are armed with a biorthogonal tag can enable the identification and validation of target proteins. Such chemical tools can also enable investigation of the engagement of drugs and other bioactive molecules with target proteins within a cellular context. A resurgence of interest in electrophilic bioactive molecules has yielded FDA-approved drugs in therapeutic areas as diverse as cancer, virology and sickle cell anaemia^3,4^. These drugs include ibrutinib (Bruton Tyrosine Kinase), nirmatrelvir (SARS-CoV-2 main protease), afatinib (epidermal growth factor receptor) and sotorasib (K-Ras G12C)^3^.

The structural diversity of screening sets, like that of explored chemical space more generally ^5^, is inherently limited by that of available building blocks, and the reaction classes that are used to functionalise them. Established sets^6–10^ of electrophilic screening compounds, such as α-halo and α,β-unsaturated amides, have generally been prepared using reactions from the narrow toolkit^11,12^ that dominates molecular discovery. In addition, diversity of reactivity is typically limited by the focus on warheads that target reactive, often catalytic, cysteine residues^13^. To address this issue, S(VI) exchange chemistry has been developed to yield diverse fragments with complementary warheads^14–17^. In addition, simple sulfonyl fluoride fragments have exploited in screens against specific proteins^18–20^. In proteome-wide screens of the reactivity of simple electrophiles, it has been shown that sulfonyl fluoride warheads predominantly target lysine and tyrosine^13,21^, though tailored sulfonyl fluoride probes can target a broader range of (often catalytic) residues including histidine, serine, threonine and cysteine^22^.

**Fig. 1:**
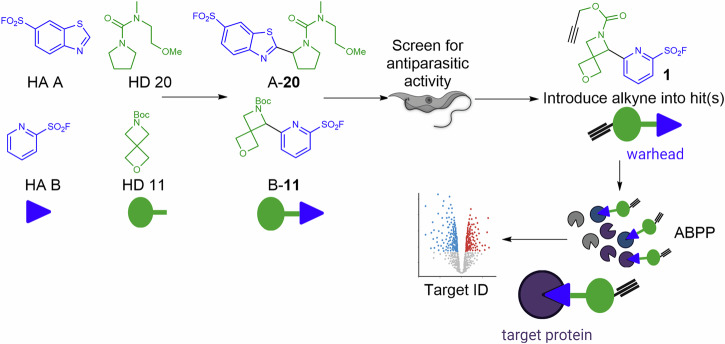
Overview of envisaged approach in which pairs of hetaryl sulfonyl fluoride (e.g., **HA A** or **HA B**; blue) and hydrogen donor (e.g., **HD 11** or **HD 20**; green) building blocks would be combined to give diverse sulfonyl fluoride probes (such as **A-20** or **B-11**). The probe set would then be screened for activity against *T. brucei* bloodstream forms. Introduction of an alkyne to hits (i.e., active compounds) would then yield chemical tools (such as **1**, a derivative of the hypothetical hit **B-11**) that may facilitate target identification/validation using established chemical proteomic workflows.

Here, report the development and execution of a unified connective synthesis of skeletally-diverse sulfonyl fluorides (Fig. 1). In contrast to approaches involving S(VI) exchange chemistries, we exploited photoredox-catalysed dehydrogenative coupling reactions^23–26^ between pairs of available building blocks: hetaryl sulfonyl fluorides and hydrogen donors (based upon saturated nitrogen and/or oxygen heterocycles). The products would be formed by connecting sp^2^- and sp^3^-hybridised carbons in the building blocks and are expected to complement those accessible using the narrow reaction toolkit that dominates bioactive molecular discovery^11,12^. Within the context of this paper we use the term “probe” to refer to chemical tools of this nature. We then demonstrated that screening of the resulting probe set could enable discovery of novel inhibitors of *Trypanosoma brucei*, a parasitic kinetoplastid that causes vector-borne African trypanosomiasis (sleeping sickness)^27^. These parasites are known to have two specific enzymes in their glycosylphosphatidylinositol biosynthetic pathway – an inositol acyltransferase and an inositol deacylase – that are inhibited by phenylmethylsulfonyl fluoride and diisopropylfluorophosphate respectively ^28^. Finally, we show that chemical proteomic workflows can enable identification of targets of these inhibitors that may provide new opportunities for therapeutic intervention.

## Results

### Design and synthesis of set of diverse probes

**Fig. 2:**
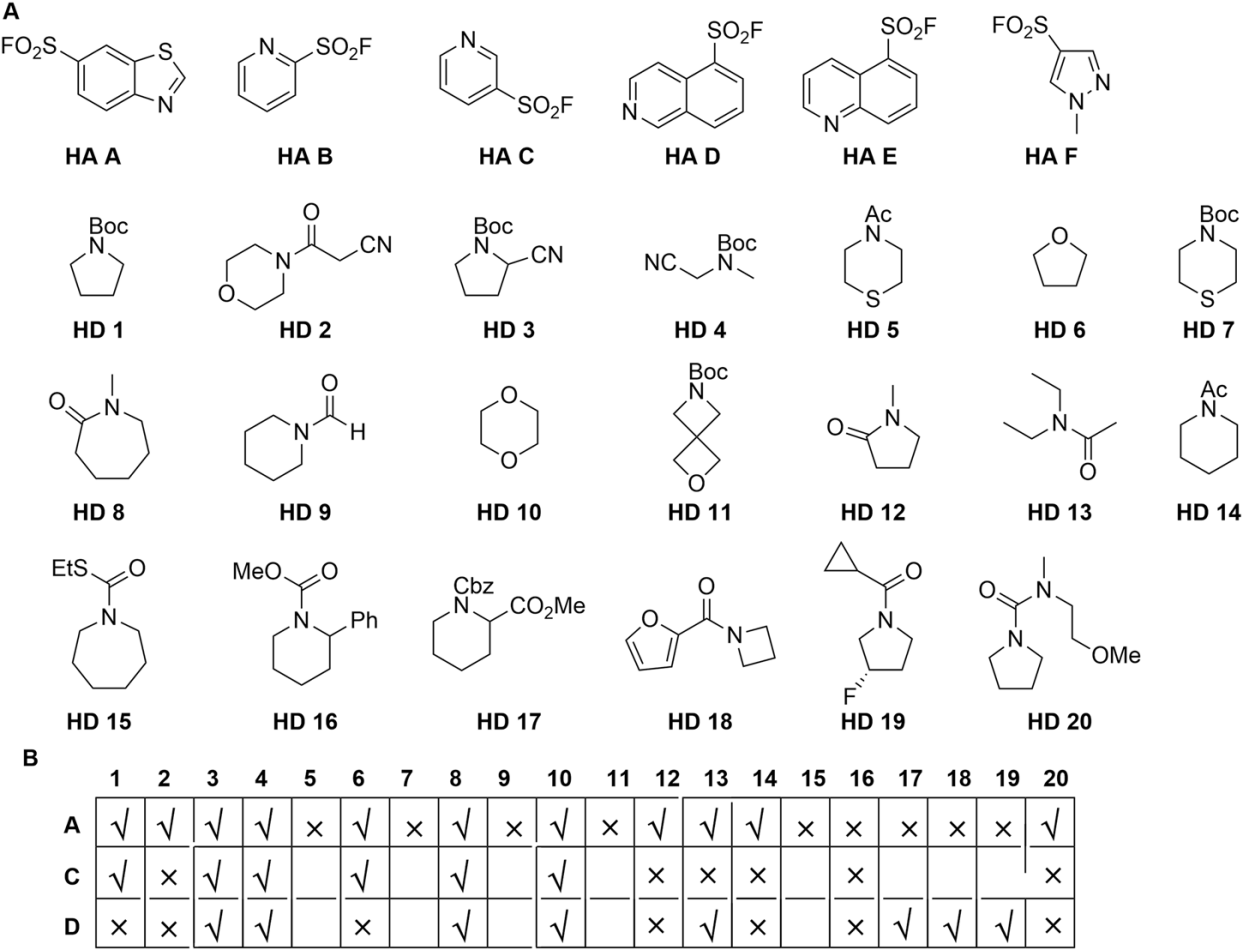
Exploration of photoredox-catalysed couplings of pairs of building blocks. **A** Structures of hetarene (**HA A-F**) and hydrogen donor (**HD 1-20**) substrates. **B** Explored combinations are denoted by a tick or a cross depending on whether sulfonyl fluoride probes were successfully obtained. Hetarenes **HA B,** **E** and **F** were only explored in combination with **HD 1** and no coupled product was obtained.

We selected a range of diverse hetaryl sulfonyl fluorides (**HA A-F**) and hydrogen donors (**HD 1-20**) as potential substrates (Fig. 2A). The sulfonyl fluorides **HA A-F**, selected on the basis of both their diversity and their likely synthetic/commercial accessibility, were based on a range of different hetaromatic rings (benzothiazole, pyridine, quinoline, isoquinoline, pyrazole) that have potential to participate in Minisci reactions^29^. The hydrogen donors **HD 1-20** were generally based on diverse saturated heterocyclic ring systems, and each included at least one type of C−H bond α to nitrogen or oxygen. These substrates included *N-*alkoxycarbonyl and *N*-acyl derivatives of secondary amines, cyclic ethers, *N*-methyl lactams and a urea. The envisaged dehydrogenative coupling reaction would result in the C−H functionalisation of both participating substrates, with concomitant formation of a C−C bond between the building blocks. For many of the substrate pairs, alternative products might be formed due to the presence of multiple potential functionalisation sites in one or both substrates.

We adopted a screening approach to identify substrate pairs that could be productively connected (Fig. 2B). For most of the investigated substrate pairs, a hetaryl sulfonyl fluoride, a hydrogen donor (5 eq.), TFA, 1 mol% Ir[dF(CF_3_)ppy]_2_(dtbbpy)PF_6_ and *tert*-butyl peracetate (TBPA) were added sequentially to a glass vial as stock solutions in acetone (total reaction volume: 1 mL). However, 30 equivalents of the formamides or cyclic ether substrates (**HD 6,** **HD 9** and **HD 10**) were used^24^. The stirred reactions were irradiated using a 390 nm Kessil lamp in a fan-cooled HepatoChem lightbox for 24 hr, and the crude reaction products were analysed using LC-MS, and ^1^H and ^19^F nuclear magnetic resonance (NMR) spectroscopy (Supporting Information). The most promising reactions were purified by mass-directed high performance liquid chromatography (HPLC) (Supplementary Table S1).

Initially, we investigated the coupling of each of the hetaryl sulfonyl fluorides (**HA A-F**) with *N*-Boc pyrrolidine (**HD 1**), a well-established hydrogen donor substrate^24^. Analysis of the crude reaction mixtures revealed three promising reactions: those involving **HA A,** **HA C** and **HA D**. Mass-directed HPLC purification of two of these reactions yielded respective coupled products: **A-1** and **C-1**. It was therefore decided to focus subsequently on reactions involving these three hetaryl sulfonyl fluorides.

**Fig. 3:**
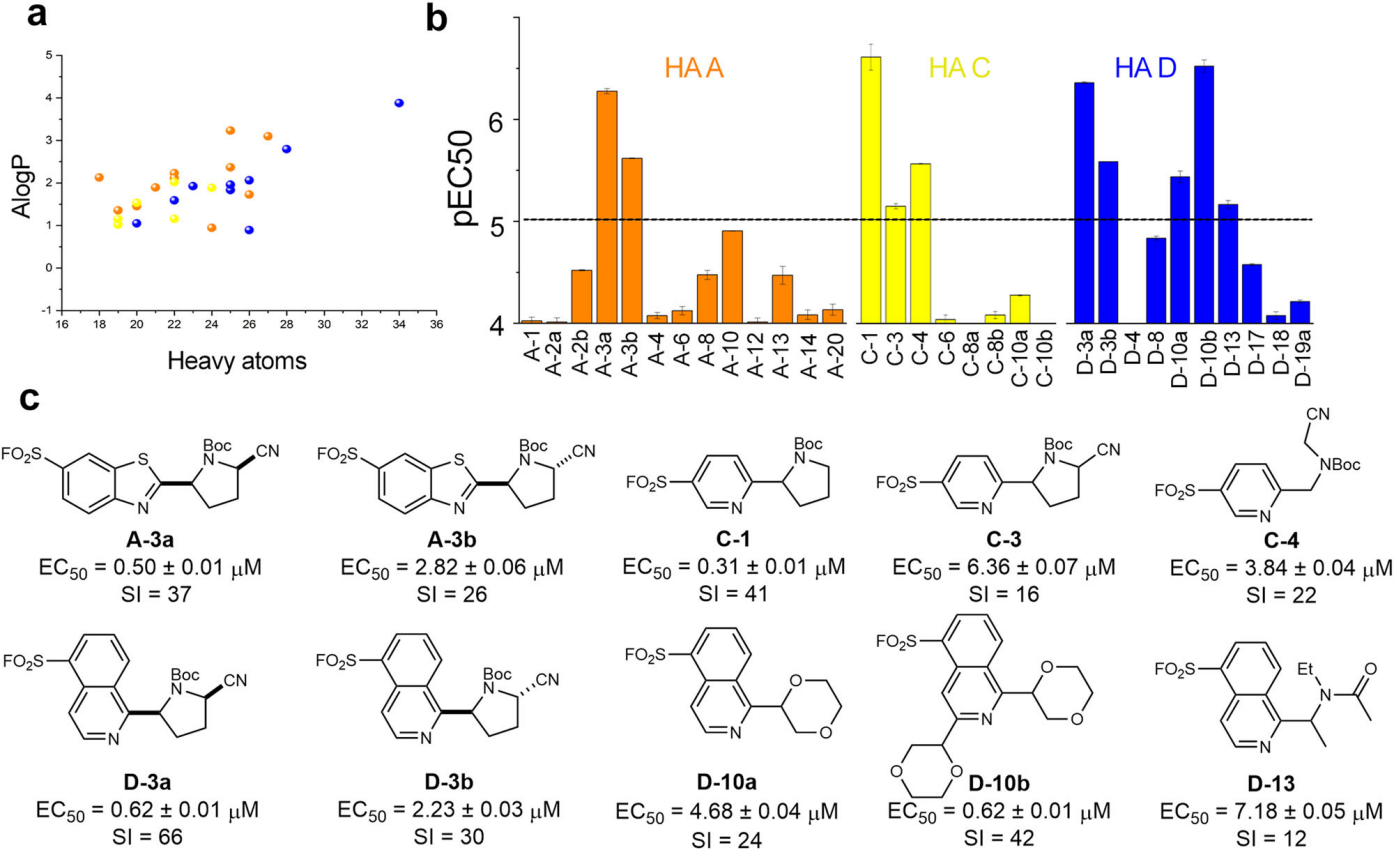
Screen of the set of sulfonyl fluoride probes against *T. brucei* bloodstream form. **a** Molecular properties of the probe set; probes derived from the hetaryl sulfonyl fluorides **HA A** (orange), **HA C** (yellow) and **HA D** (blue) are indicated by colour (see Supplementary Fig. S1 for all structures). **b** Activity of the probes against *T. brucei* bloodstream forms; initial screen in single replicate, dose-response; bars indicate mean values ± standard errors (fitting error); pEC_50_ = −log_10_(EC_50_). **c** Chemical structures of probes with pEC_50_ > 5 against *T. brucei* (*n* = 4 technical replicates, mean ± S.D.); selectivity indices (SI) over cytotoxicity against HeLa cells (*n* = 4 technical replicates) are indicated; see Supplementary Table S4 for EC_50_ values against HeLa.

Next, we investigated the coupling of the hetaryl sulfonyl fluoride **HA A** with all twenty of the hydrogen donors (**HD 1-20**). After analysis, 11 of these reactions were deemed sufficiently promising for purification; and mass-directed HPLC yielded a total of 13 productively-coupled products. It was decided to focus subsequently on reactions involving selected hydrogen donors: the *N*-alkoxycarbonyl derivatives **HD1,** **HD 3,** **HD 4,** **HD 16** and **HD 17**; the amides **HD 2,** **HD 13** and **HD 14**; the cyclic ethers **HD 6** and **HD 10**; the *N*-methyl lactams **HD 8** and **HD 12**, and the urea **HD 20**. These prioritised hydrogen donors were then investigated in combination with two remaining hetaryl sulfonyl fluorides (**HA C** and **HA D**). After analysis, and purification of the most promising reaction mixtures, a further 19 sulfonyl fluoride probes were obtained. It was found that **HA C** was selectively substituted initially at the 6-position (followed by substitution at the 2- or 4-position to give 2,6- or 2,4-disubstituted analogues) (see Supplementary Tables S2 and S3). **HA D** was selectively substituted initially at the 1-position (with one 1,3-disubstituted analogue also observed). In total, 32 sulfonyl fluoride probes were prepared (Supplementary Fig. S1) whose molecular properties are presented in Fig. 3a.

### Screen of probes against *T. brucei*

The set of diverse sulfonyl fluoride probes was screened against *Trypanosoma brucei brucei* bloodstream forms in 96-well plate format (final concentrations: 0.1–100 μM in 0.5% dimethyl sulfoxide (DMSO); *n* = 1) (Fig. 3b). Ten of the probes had EC_50_ < 10 μM (i.e., pEC_50_ > 5), four of which had half maximal effective concentration (EC_50_) < 1 μM (i.e., pEC_50_ > 6) (Fig. 3c). Hits were re-screened against *T. brucei* (*n* = 4 technical replicates) to confirm EC_50_ values (Supplementary Table S4). To assess selectivity, the probe set was screened against HeLa cells (Supplementary Table S4), and the ten most active probes were found to have between 12- and 66-fold selectivity for *T. brucei*. Furthermore, the ten most active probes had between 17- and 500-fold higher activity against *T. brucei* than the parental hetaryl sulfonyl fluoride substrates from which they were derived: **HA A** (EC_50_ = 111 ± 3 μM), **HA C** (EC_50_ = 154 ± 2 μM) and **HA D** (EC_50_ = 124 ± 3 μM). Probe activity is thus critically dependent upon the specific substitution pattern of the hetarene.

### Chemical proteomics using an alkynylated probe

We noted that alkynylated probes based upon **A-3a** and **C-1**, which both had promising activity against *T. brucei* (EC_50_ < 1 μM), might be readily preparable. The probe **A-3a** contains two potential warheads: in addition to the sulfonyl fluoride, it contains an α-cyano amine, a warhead found in reversible covalent inhibitors of cysteine proteases^30^. We therefore compared the activity of **A-3a** (EC_50_ = 0.50 ± 0.01 μM) with that of analogues lacking either the sulfonyl fluoride (EC_50_ = 1.37 ± 0.01 μM) or the α-cyano (**A-1**; EC_50_ = 101 ± 2 μM) groups (Supplementary Fig. S1). Surprisingly, the α-cyano group in **A-3a**, rather that its sulfonyl fluoride, was critical for antitrypanosomal activity; as a result, we deprioritised preparation of an alkynylated analogue of **A-3a**. We prepared an alkynylated analogue of **C-1** – **C-1alk** – by deprotection and subsequent reaction with propargyl chloroformate (Supporting Information).

**Fig. 4:**
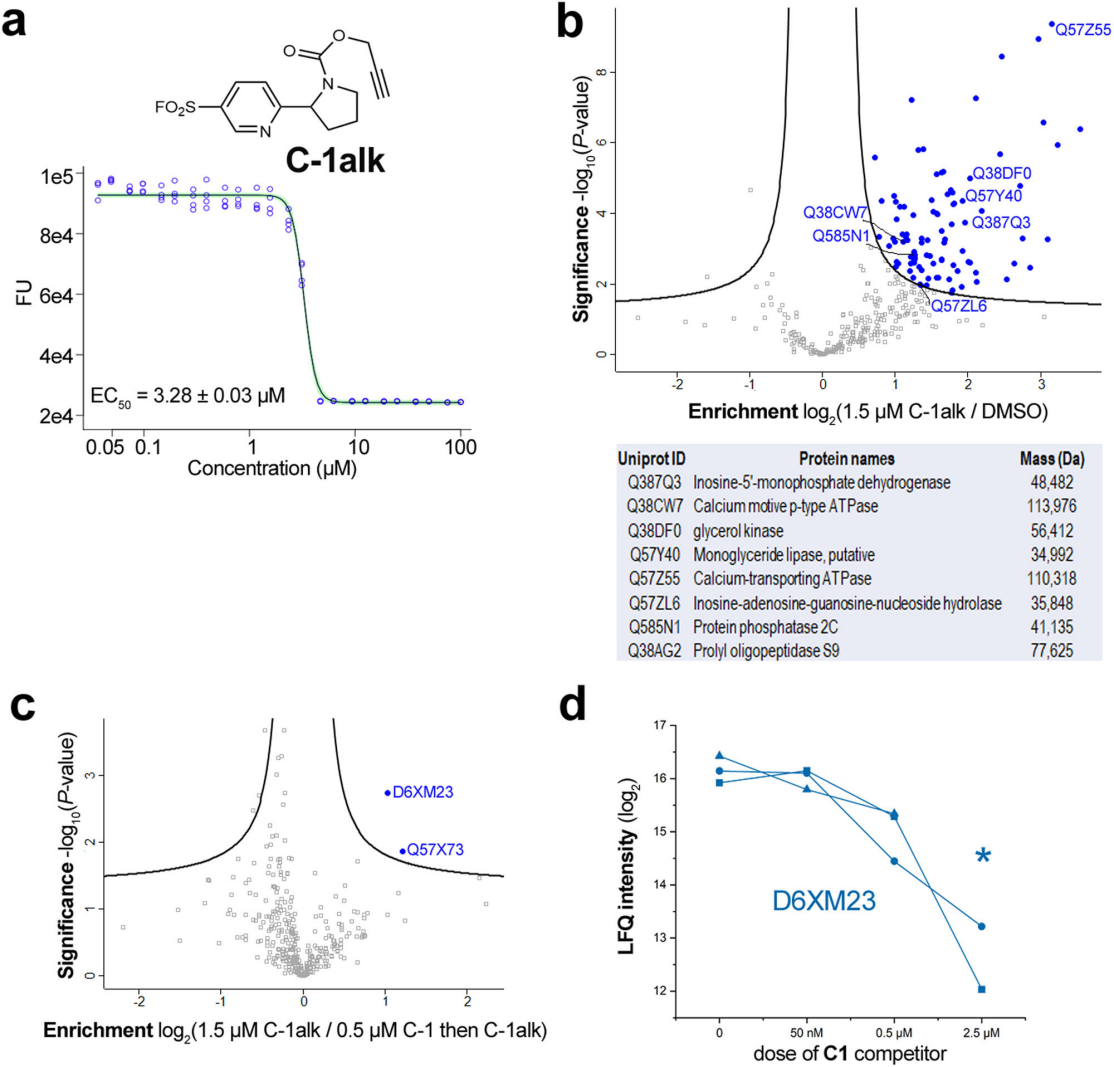
Mass spectrometry analysis of enriched proteins using C-1alk. **a** Structure and dose-response activity of **C-1alk**. Viability is expressed in terms of changes in fluorescence units (FU) in the resazurin assay; fitting errors are shown from experiments performed in biological triplicate. **b** Scatter plot showing the protein hits quantified following incubation of 1.5 μM **C-1alk** with *T. brucei* lysates, compared with DMSO-treated control. Blue dots indicate those protein hits significantly enriched after comparing data from three biological replicates injected separately at least twice. The list summarises representative proteins whose Uniprot IDs are highlighted in the scatter plot. **c** Scatter plot showing proteins quantified in proteomics experiments following incubation with 0.5 μM **C-1** and subsequent treatment with 1.5 μM **C-1alk**, compared with treatment with 1.5 μM **C-1alk** alone. A modified two-sample *t*-test with permutation based false discovery rate (FDR) statistics was applied (250 randomisations, FDR = 0.01 and s0 = 0.2) for group comparisons shown in scatter plot. Two proteins whose abundance was significantly changed in the two conditions are indicated in blue. **d** Changes in label-free quantification (LFQ) of D6XM23 as a function of the concentration of **C-1** used prior to incubation with 1.5 μM **C-1alk**. *N* = 3 independent replicates are plotted. Note that values at 2.5 µM **C-1** are imputed (indicated by an asterisk), as the protein was not quantified by LFQ in these samples.

Crucially, **C-1alk** retained some activity against *T. brucei* (EC_50_ = 3.28 ± 0.03 μM) (Fig. 4a). We therefore used **C-1alk** as a tool to investigate the mode of action of **C-1**. We initially incubated varying concentrations of **C-1alk** with *T. brucei* lysates and, following click reaction with rhodamine-N_3_, visualised the labelled proteins in-gel (Supplementary Fig. S2). On the basis of the observed labelling patterns, we profiled the proteins that were pulled down following treatment of lysates with 1.5 μM **C-1alk** and subsequent click reaction with 100 μM biotin-N_3_ (Fig. 4b). Consistent with our in-gel fluorescence scanning analysis, which showed a large number of labelled bands, ninety-five proteins were significantly enriched in **C-1alk**-treated samples compared to the DMSO background control (Fig. 4c and Table S4). Gene ontology (GO)-enrichment analysis showed an over-representation of nucleotide-binding, translation machinery and hydrolytic enzymes within the **C-1alk**-enriched proteins (Supplementary Fig. S3). We sought to detect **C-1alk**-modifed peptides by using a trypsin-cleavable biotin-azide reagent^31^. We were able to identify peptides matching the expected mass shift for *T. brucei* glycerol kinase (Q38DF0), with the modification site annotated as S137, T138 or Y139 (Supplementary Fig. S4). Notably, the tyrosine residue (Y139 in this enzyme) is highly conserved across many species and is proximal to the substrate binding site^32^.

To pinpoint targets of the parent ligand **C-1**, we used it to outcompete **C-1alk** labelling. For this, parasite lysates were treated with **C-1** below (50 nM), around (500 nM) and above (2.5 μM) its EC_50_ (310 ± 10 nM), and then labelled with 1.5 μM **C-1alk**. Two proteins were significantly depleted in the 0.5 μM **C-1**-pre-treated samples: D6XM23, a putative *lyso*-phospholipase; and Q57X73, a putative nucleoside hydrolase (Fig. 4c). We investigated the enrichment of these two proteins as a function of the concentration of **C-1** used in the pre-treatment (Table S5); Q57X73 is only found in the **C-1alk** sample and not detected when samples were pre-treated with **C-1** at any concentration, whereas D6XM23 exhibited decreasing intensity with increasing **C-1** dose and was undetectable at 2.5 µM **C-1**.

### Chemical proteomics using fluorophosphonate probes

**Fig. 5:**
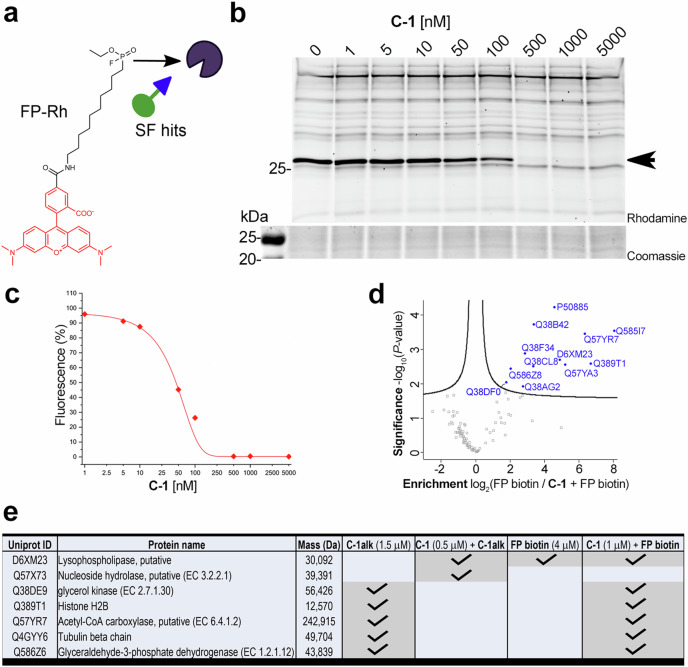
Analysis of proteins enriched using fluorophosphonate probes. **a** Structure of a fluorophosphonate-rhodamine probe, **FP-Rh**. **b** In-gel fluorescence analysis of **FP-Rh** targets as a function of the concentration of **C-1**. In-gel fluorescence scanning and Coomassie staining (bottom panel) corresponding to the region of interest are shown (full gel image presented in SI Figure S2b. **c** Densitometry analysis of the ~25 kDa predominant protein band (red arrow) showed that labelling was blocked by **C-1** (EC_50_: 46.4 ± 5.5 nM; error from fitting). **d** Scatter plot showing proteins quantified in proteomics experiments following incubation with 1 μM **C-1** and subsequent treatment with 4 μM **FP-biotin**, compared with treatment with 4 μM **FP-biotin** alone. A modified two-sample *t*-test with permutation based false discovery rate (FDR) statistics was applied (250 randomisations, FDR = 0.01 and s0 = 0.2) for group comparisons shown in scatter plot. Proteins whose abundance was significantly changed in the two conditions are indicated in blue. **e** Summary of protein hits identified across the pulldown assays. Grey boxes indicate the condition for which this protein was enriched.

The identification of a serine hydrolase (D6XM23) as a potential target of **C-1**, and the more general irreversible inhibition of serine hydrolases by sulfonyl fluorides^22^, prompted us to investigate more generally whether serine hydrolases were targeted by our anti-trypanosomal compounds. In these experiments, we used fluorophosphonate tool compounds, which are useful broad-spectrum probes of serine hydrolases^33^. Initially, we incubated *T. brucei* lysates with the sulfonyl fluoride probes (100 μM; **A-3a,** **D-3a** and **D-10b** in addition to **C-1**), followed by treatment with 2 μM fluorophosphonate rhodamine (**FP-Rh)** and in-gel visualisation (Fig. 5a, b; and Supplementary Fig. S5a). For each of the probes, it was noted that several bands had disappeared, or were less intense, compared to DMSO control. We showed that **C-1** and **C-1alk** outcompete similar targets in a gel-based competition assay using **FP-Rh** (Supplementary Fig. S5b). We also quantified the intensity of a selected band as a function of the concentration of **C-1** (Fig. 5c); here, an intense ~25 kDa band labelled by **FP-Rh** was outcompeted by sub-micromolar concentrations of **C-1**. Using **FP-biotin**, the enriched proteins, compared to DMSO control, included four hydrolases (Supplementary Fig. S6). Finally, we profiled the serine hydrolases that were targeted by incubation of 1 μM **C-1** in lysates, and subsequent treatment with 4 μM **FP-biotin** (Fig. 5d, e). Fifteen proteins were significantly depleted, compared to incubation with **FP-biotin** alone, including *lyso*-phospholipase (D6XM23), glycerol kinase (Q38DF3), prolyl endo-/oligo-peptidase S9 (Q38AG2), which had been previously enriched with our **C-1alk** probe (Fig. 4b).

## Discussion

Our unified synthetic approach enabled diverse sulfonyl fluoride probes to be prepared in a single step from pairs of readily-available building blocks. The probes are based on 23 distinct graph-node-bond frameworks^34^, demonstrating that the connective approach can yield skeletally-diverse products that are inaccessible via established diversity-oriented S(VI) exchange chemistries^14–17^. Remarkably, the photoredox-catalysed coupling chemistry was compatible with sulfonyl fluoride functionality, enabling the direct preparation of diverse probes complementing those bearing cysteine-targeted warheads.

The compounds were also functionally diverse, displaying activities against *Trypanosoma brucei* that varied >300-fold. We had designed the compounds such that alkynylated analogues might be readily prepared, to facilitate rapid target identification by chemical proteomics. Indeed, **C-1** (EC_50_ = 0.31 ± 0.01 μM) was readily converted into **C-1alk** (EC_50_ = 3.28 ± 0.03 μM). We undertook a series of chemical proteomic experiments to deconvolute **C-1alk** and **C-1** targets in parasite lysates. **C-1alk** labelled ~100 proteins (Supp. Table S5). This is consistent with the literature as the sulfonyl fluoride compounds prepared here are intermediate in complexity between structurally simple sulfonyl fluoride probes, which enrich a broad range of protein targets from proteomes^35,36^, and complex scaffolds based upon selective kinase inhibitors that show greater specificity for their target class in chemical proteomics experiments^16^.

Enzymes targeted by **C-1alk** included those that enable: bases to be salvaged for nucleic acid and cofactor synthesis; de novo synthesis of eukaryotic glycerophospholipid and sphingolipids; and glycolysis. To further pinpoint specific **C-1** targets, we pre-incubated parasite lysates with **C-1** before **C-1alk** treatment (Supp. Table S6); this revealed two clear hits that showed a loss of enrichment in response to increasing **C-1** concentrations: nucleoside hydrolase (Q57ZL6) and *lyso*-phospholipase (D6XM23).

Noting that *lyso*-phospholipase is a serine hydrolase, we used broad spectrum fluorophosphonate probes, **FP-Rhodamine** and **FP-biotin**, to show that **C-1** (and **C-1alk**) target a subset of this class of enzymes (Supp. Table S7). This is consistent with the extensive use of sulfonyl fluorides as protease inhibitors and probes that engage hyper-reactive serine residues^22,37^. However, modification of residues other than lysine and tyrosine is not always detected through proteomic approaches^13^, likely stemming from the lability of the resultant linkages with other residues, including serine^38^. On a technical note, avoiding the use of thiol reducing agents in our **C-1alk** chemical proteomic workflow may have facilitated retention of serine hydrolases in this case.

*Lyso*-phospholipase emerged as the sole common hit labelled by both **C-1alk** and **FP-biotin** whose enrichment was reduced in the presence of **C-1**. *Lyso*-phospholipase (Tb927.8.6390 in TriTrypDB^39^) was identified as acyl-protein thioesterase-like, but was characterised as lacking activity on acylated proteins, instead possessing esterase activity on short and medium-chain fatty acids^40^. As one of the most highly enriched serine hydrolases from **FP-biotin** pull-downs (Supp. Figure S6) and with a molecular weight of 30 kDa, *lyso*-phospholipase is a strong candidate for the prominent ~27 kDa band labelled by **FP-Rh** that is also competed by **C-1** and **C-1alk** by SDS-PAGE (Fig. 5, Supp. Figure S5). However, a genome-wide RNA interference (RNAi) screen and RNAi knockdown or overexpression of this enzyme showed no effect on parasite proliferation^41^ nor on mouse virulence^42^, making it unlikely that *lyso*-phospholipase is a physiologically relevant **C-1** target for impairing proliferation of the parasite.

The other hit identified as a **C-1** target from **C-1alk** proteomics, Q57ZL6, is an inosine-adenosine-guanosine-nucleoside hydrolase (IAGNH; Tb927.3.2960 in TriTrypDB)^39^, a glycosomal enzyme involved in purine salvage. IAGNH has been structurally characterised^43^ and investigated as a drug target^44^ due to the essentiality of the purine salvage pathway in all stages of *T. brucei* and the lack of nucleoside hydrolases in mammalian cells. A potent inhibitor was found to be effective against bloodstream forms and in a mouse model of infection^44^. However, the gene was shown to lack essentiality as reported in the genome-wide RNAi screen^41^, as well as in the inhibitor study, suggesting that inhibition of this enzyme is not sufficient for parasite growth inhibition and that the inhibitor acts on multiple targets^45^. We speculate the bioactivity of **C-1** may be attributed, in part, to IAGNH inhibition. Of special interest we found that all the three enzymes, *lyso*-phospholipase, IAGNH and glycerol kinase, present glycosomal localisation suggest that perturbations on these organelles may be attributed to **C-1** anti-trypanosomal effect (selectivity and potency) observed on bloodstream forms^46^.

Other proteins enriched by **C-1alk** that were also hits from competition of **C-1** with **FP-biotin** (glycerol kinase, glyceraldehyde 3-phosphate dehydrogenase (GAPDH), Histone H2B, tubulin beta and acetyl-CoA carboxylase; Fig. 5e) are all abundant housekeeping proteins and show no significant depletion by **C-1** in **C-1alk** labelling (Supp. Table S5), suggesting that these are unlikely to be robust targets of **C-1**.

In conclusion, we generated a small library of structurally diverse sulfonyl fluorides, offering straightforward access to alkynylated versions for target deconvolution via chemical proteomics. Applying a set of proteomics experiments, we identified protein binders of compound **C-1**, revealing potential targets for further follow-up and validation. The anti-trypanosomal activity of **C-1** may well stem from covalent modification of multiple protein targets. It is notable that, despite some unique biochemical features, there are very few robustly validated anti-trypanosomal drug targets. Indeed, given the size of the small size of the set of compounds screened, it is perhaps unsurprising that strong candidate protein targets were not identified. We anticipate that our approach, perhaps executed on a larger scale, may be used in the future for the discovery of high-quality tools^47–49^ for specific protein targets relevant to the underlying biology of this parasite and other pathogens.

## Methods

### Synthesis of diverse sulfonyl fluorides

To a LC-MS vial, a hetaryl sulfonyl fluoride (200 µL of a 1.5 M solution in acetone), a hydrogen donor (333 µL of a 1.5 M solution in acetone), TFA (100 µL of a 2 M solution in acetone), Ir[dF(CF_3_)ppy]_2_(dtbbpy)PF_6_ (100 µL of a 0.01 M solution in acetone) and *tert*-butyl peracetate (159 µL, 50% w/v in acetone) were added along with acetone (107 µL) to give a total volume of 1 mL. The solutions were then irradiated with a 390 nm Kessil Lamp whilst stirring in a fan-cooled HepatoChem lightbox for 24 hours. The mixtures were evaporated to give crude products, dissolved in acetonitrile and purified via mass-directed HPLC (gradient elution 5:95 → 95:5 acetonitrile−water) and lyophilisation to give pure products.

### Viability screening against *T. brucei*

Cell viability assays were carried out in 96 well plates with 200 µL of culture per well as described previously ^50^. For this study we used bloodstream forms (BSF) of *Trypanosoma brucei brucei* strain 427 single marker. Axenic cultures of parasites were maintained at the logarithmic phase of cell proliferation (density below 2 ×10^6^ parasites/mL) by successive passages in HMI-11 media supplemented with 10% heat inactivated foetal calf serum and 2.5 mg/L geneticin (Ge418) at 37°C under an atmosphere of 5% CO_2_. Parasites were seeded at 5 ×10^3^ cells/mL and incubated with drug for 66 hours. The plate included wells containing the positive control pentamidine (100 nM) and the negative control of 0.5% DMSO. After 66 hours, 10 µL of 1.1 mg/mL Resazurin sodium salt was added in PBS and incubated for a further 6 hours. Plates were then read on a plate reader using excitation/emission 560/590 nm. EC_50_ values and errors were calculated using a sigmoidal dose response fitting algorithm. The initial screen was performed at *n* = 1 with follow-up screen of hits *n* = 4 technical replicates.

### HeLa resazurin cell viability assay

HeLa cells (ATCC) were grown at 37 °C in an atmosphere containing 5% CO_2_, in Dulbecco’s Modified Eagle Medium (DMEM), containing foetal bovine serum (FBS) (10%), L-glutamine (1%) and penicillin/ streptomycin (1%). Cell viability assays were carried out in 96 well plates with 200 μL of media per well. Cells were seeded at 2.5 × 10^4^ cells/mL and incubated with the library compounds in quadruplicate (technical replicates) for 66 hours. The plate included wells containing a no-cell positive control, and the negative control of 0.5% DMSO. After 66 hours, 10 μL of 1.1 mg/mL Resazurin sodium salt (in phosphate-buffered saline, PBS) was added and plates incubated for a further 6 hours (for a total assay duration of 72 hours). Plates were then read on a plate reader using excitation/emission 560/590 nm. EC_50_ values were calculated using a sigmoidal dose response fitting algorithm programmed in R using the LL.4 logarithmic fitting function and plotting the replicate data to output an EC_50_ and error values.

### Parasite protein sample preparation

Parasite cultures were scaled up to obtain a cell pellet containing approximately 5 × 10^7^ cells at the mid-log phase of proliferation. Cells were harvested by centrifugation (800 x *g* for 10 min), resuspended in 1 mL of Trypanosome dilution buffer (TDB: 5 mM KCl, 80 mM NaCl, 1 mM MgSO_4_, 20 mM Na_2_HPO_4_, 2 mM NaH_2_PO_4_, 20 mM d-glucose, pH adjusted to 7.7), and transferred to a 1.5 ml microcentrifuge tube. Cell suspension was centrifuged at 3,000 r.p.m for 3 min and this step was repeated once more. Parasites were counted in a haemocytometer and cell pellet was resuspended thoroughly in de-ionised water up to achieve a concentration of 5 × 10^8^ cells/mL. Samples were incubated on ice for 5 min to allow osmotic lysis and snap frozen in liquid nitrogen. Protein content was determined by bicinchoninic acid (BCA) assay using bovine serum albumin (BSA) as standard (ThermoFisher Scientific), and aliquots of 1 mg/mL parasite lysate were stored at -80 °C until further use.

### In-gel visualisation of proteins labelled by C-1alk

Parasite lysates were thawed on ice and 25 µg of lysate were mixed with either 1% DMSO in 1x PBS, or 1.5 µM of **C-1alk** in a 25 µL final volume for 1 h at 25 °C under constant mixing (600 r.p.m). After this time 1% SDS was added for copper-catalysed alkyne-azide cycloaddition (CuAAC) in the presence of 100 µM rhodamine-azide, 50 µM CuSO_4_, 100 µM tris(2-carboxyethyl)phosphine (TCEP) (pH 7), and 50 µM of ligand (Tris[(1-benzyl-1H-1,2,3-triazol-4-yl)methyl]amine (TBTA) dissolved in 20% DMSO) and incubated for an additional 1 hour. The reaction was stopped by adding 20 mM of ethylenediaminetetraacetic acid (EDTA) and proteins were precipitated with acetone. Briefly, 1 mL of chilled acetone was added, mixed by vortex, and incubated at -20 °C. After MeOH washing and drying, samples were resolved in 1x Laemmli sample loading buffer (0.125 M Tris base, 4% SDS, 20% glycerol and 10% 2-mercaptoethanol) sample loading buffer, analysed by sodium dodecyl-sulphate polyacrylamide gel electrophoresis (SDS-PAGE) and fluorescent bands visualised in a Chemidoc Imaging System (Bio-Rad) under UV-tray and rhodamine detection filters. Total protein was assessed by Coomassie staining for loading control.

### In-gel visualisation of proteins labelled by FP-Rh

Parasite lysates were labelled with 2 µM fluorophosphonate **FP-Rh** (FP-TAMRA, ThermoFisher Scientific) and protein content visualised in SDS-PAGE as described above. For competition assays, lysates were preincubated for 30 min with 100 µM of our hit compounds as detailed in the figure legends.

### Biotin enrichment and Label-free proteomic profiling

Parasite lysates (1 mg/mL) were incubated with 1.5 µM **C-1alk** for 1 hour at 25 °C under constant agitation (600 r.p.m). When required addition of either 1% DMSO or parent compound was included for 30 min prior to alkyne treatment. Samples were mixed with 1% SDS and added of 100 µM biotin-azide (in house produced^31^), 50 µM CuSO_4_, 100 µM TCEP (pH 7), and 50 µM of TBTA ligand in a final volume of 0.25 mL, following incubation for one hour. CuAAC reaction was quenched by addition of 20 mM EDTA and protein samples were precipitated with methanol – chloroform - water (4:1.5:1), washed with methanol, and air dried.

For pulldown assays using **FP-biotin**, the lysates were incubated with 4 μM ActivX FP-desthiobiotin (ThermoFisher Scientific) for 30 min at 25 °C under constant agitation, and then followed by protein precipitation as described above. The protein pellet was resuspended at 10 mg/mL in 2% SDS-PBS, then diluted to 0.2% SDS-PBS.

100 µL NeutrAvidin agarose beads (ThermoFisher Scientific) were pre-equilibrated with 0.2% SDS-PBS, and then mixed with the protein sample for 2 hours in a head-to-tail rotor at room temperature. Beads were sequentially washed (3 times each solution) and centrifuged (1,500 x *g* at RT) with 0.2% SDS-PBS, 8 M urea, 1x PBS, and 50 mM tetraethylammonium bicarbonate (TEAB). Beads were resuspended in 250 µL TEAB containing 12.5 mM TCEP-HCl (1 h at 30°C) for reduction, followed by one washing step with TEAB before the addition 18.75 mM iodoacetamide for alkylation (1 h at room temperature under light protection). Beads were washed again in TEAB, centrifuged, and resuspended in 250 µL TEAB added of 5 µg trypsin protease – MS grade (ThermoFisher Scientific) dissolved in 50 mM acetic acid. Protease digestion was carried out for 16 h at 37°C under constant mixing. Digested peptides were recovered from supernatant and the pH adjusted to ~3 with formic acid. Samples were evaporated in a speed vac concentrator, resuspended in 1 mL of 0.1% trifluoroacetic acid (TFA) in MS-grade water and desalted in tC18 sep-pak 50 mg cartridges (Waters) using a vacuum manifold according to manufacturer’s instructions. The tC18 cartridges were pre-conditioned with 90% MeOH: water 0.1% TFA, followed by water 0.1% TFA, which was also used in subsequent washing steps, and 5% MeOH: water 0.1% TFA used for desalting. Peptides were eluted after 10 min on-column incubation with 1 mL 50% MeCN: water, 0.1% TFA and, after gentle elution, samples were again dried in a speed vac concentrator.

Tryptic peptides were resuspended in 15 µL of 2% MeCN in MS grade water, 0.1% formic acid from which 1 µL were injected into a nanoElute 2 liquid chromatography system equipped with an Aurora Elite C18 analytical column (IonOpticks, Australia) connected to a trapped ion mobility timsTOF Pro 2 mass spectrometer (Bruker Daltonik GmbH, Germany).

### Liquid chromatography mass spectrometry (LC-MS) for proteomics

Chromatographic separation was performed on a nanoElute sample analyser (Bruker, Daltonik GmbH, Germany) equipped with a two-column separation stage. The trap column was a Thermo Trap cartridge (5 mm) followed by an IonOpticks Aurora Elite C18 separation column (150 mm, 0.1 mm, 5 µm) held at 40 °C, with a flow rate of 0.4 µL/min and injection volume of 2 µL loaded at 84.7 bar. Mobile phase A was 0.1% formic acid dissolved in mass spectrometry- (MS) grade water, and mobile phase B comprised 0.1% formic acid dissolved in MeCN. The gradient was as follows: initial 2% B, 2-35% B over 30 minutes, to 95% B in 0.5 minutes, followed by 3.42 minutes re-equilibration.

The ESI module used a BrukerCaptive spray ionisation interface for the Bruker ZDV sprayer (i.d = 20 µm). Mass detection was performed using a quadrupole to select precursor ions and a time-of-flight analyser for spectra acquisition, which are coupled to a trapped ion mobility (TIMS) device for an additional parameter of ion separation. Ordinary collision-dissociation in the reaction cell was applied by nitrogen gas at 6 bar. Data dependent acquisition mode (DDA) was performed using 10 parallel accumulation and serial fragmentation (PASEF) scans (1.16 s cycle time) in a mass range of 100-1,700 *m/z* in the positive mode within a charge set from 0 as minimum and 5 as maximum. A capillary voltage of 1,500 V at 180°C and 3.0 L/min of dry gas flow was applied in the source, and 0.60-1.60 V s/cm^2^ (1/*K*_*0*_) with a ramp and accumulation time of 100 ms at a rate of 9.52 Hz, and 100% duty cycle for the TIMS settings. The Bruker timsControl v3.1 and Hystar v6.030.0 were used as the data acquisition software. Raw Bruker TDF datasets were analysed in MaxQuant (v1.4.10.0) software using the Andromeda search engine^51^ for label free quantification (LFQ)^52^ against the *T. brucei* strain 927/4 GUTat10.1 proteome dataset retrieved from Uniprot database (UP000008524) as reference.

### Data searching and analysis

The resulting tims-DDA data were quantified on MaxQuant using pre-defined parameters for protein modifications (Cysteine carbamidomethylation as a fixed modification, and methionine oxidation and N-terminal acetylation as variable modifications) and enabling the match-between runs option for LFQ analysis. The false discovery rate was set to 0.01 for peptides, proteins and sites. “Unique and razor peptides” mode was selected to allow for protein grouping; this calculates ratios from unique and razor peptides (razor peptides are uniquely assigned to protein groups and not to individual proteins). Protein groups were used as input matrix in Perseus software (v2.0.11)^53^ and following removal of protein contaminants, the LFQ intensities were converted to logarithmic values (base 2) from which empty values were imputed with random numbers from a normal distribution (downshift: 1.8; width: 0.3). LFQ mean values were computed from three biological replicates performed independently with at least two injections in our timsTOF analyser. A modified *t*-test with permutation based false discovery rate (FDR) statistics was applied (250 randomisations, FDR = 0.01 and s0 = 0.2) for group comparisons. For analysis of probe modifications, we used the timsTOF Parallel Accumulation Serial Fragmentation (PASEF) raw data to search modified peptides in FragPipe (v22.0) software^54^. A mass offset search workflow consisted of the Localisation-aware Open Search (LOS = +620.12 Da, variable residue) algorithm in MSFragger, followed by PeptideProphet and PTM-Shepherd tools for PSM validation and mass shift summarisation, respectively^55,56^. Panels in all figures were prepared using Adobe Photoshop v24.5.0 and proteomics data was tabulated in Microsoft Excel (v2402).

### Reporting summary

Further information on research design is available in the Nature Portfolio Reporting Summary linked to this article.

## Supplementary information

Supplementary Information

Description of Additional Supplementary Files

Supplementary Data 1

Supplementary Table 5

Supplementary Table 6

Supplementary Table 7

Supplementary Table 8

Reporting summary

## Data Availability

The mass spectrometry proteomics data have been deposited to the ProteomeXchange Consortium via the PRIDE [1] partner repository with the dataset identifier PXD056504. Additional supplementary data is provided in the Supplementary Information (including NMR spectra for novel compounds); and Supplementary Data 1 (primary data plotted in figures in the manuscript) and Supplementary Tables 5-8 (proteins identified in chemical proteomics experiments).
