# Supplementary material for: Discovery of Trypanosoma brucei inhibitors enabled by a unified synthesis of diverse sulfonyl fluorides
Source: Commun Chem. 2024 Oct 19;7:237. doi: 10.1038/s42004-024-01327-8 (PMC11490619; doi:10.1038/s42004-024-01327-8)

Discovery of *Trypanosoma brucei* inhibitors enabled by a unified synthesis of diverse sulfonyl fluoride probes

Brian S. Mantilla[1], Jack S. White[1], William R. T. Mosedale[2], Andrew Gomm[1], Adam Nelson,*[,1] Terry K. Smith*[,2] and Megan H. Wright*[,1]

[1]School of Chemistry and Astbury Centre for Structural Molecular Biology, University of Leeds, Leeds, LS2 9JT, UK.

[2]Schools of Biology and Chemistry, Biomedical Sciences Research Complex, University of St Andrews, St Andrews, KY16 9ST, UK.

Email: a.s.nelson@leeds.ac.uk; tks1@st-andrews.ac.uk; m.h.wright@leeds.ac.uk

Supplementary information

Contents

Supplementary Figures 1
Supplementary Methods 10
General Experimental 10
General Procedures 12
Compound synthesis and characterisation 13
NMR spectra 32

Supplementary Figures

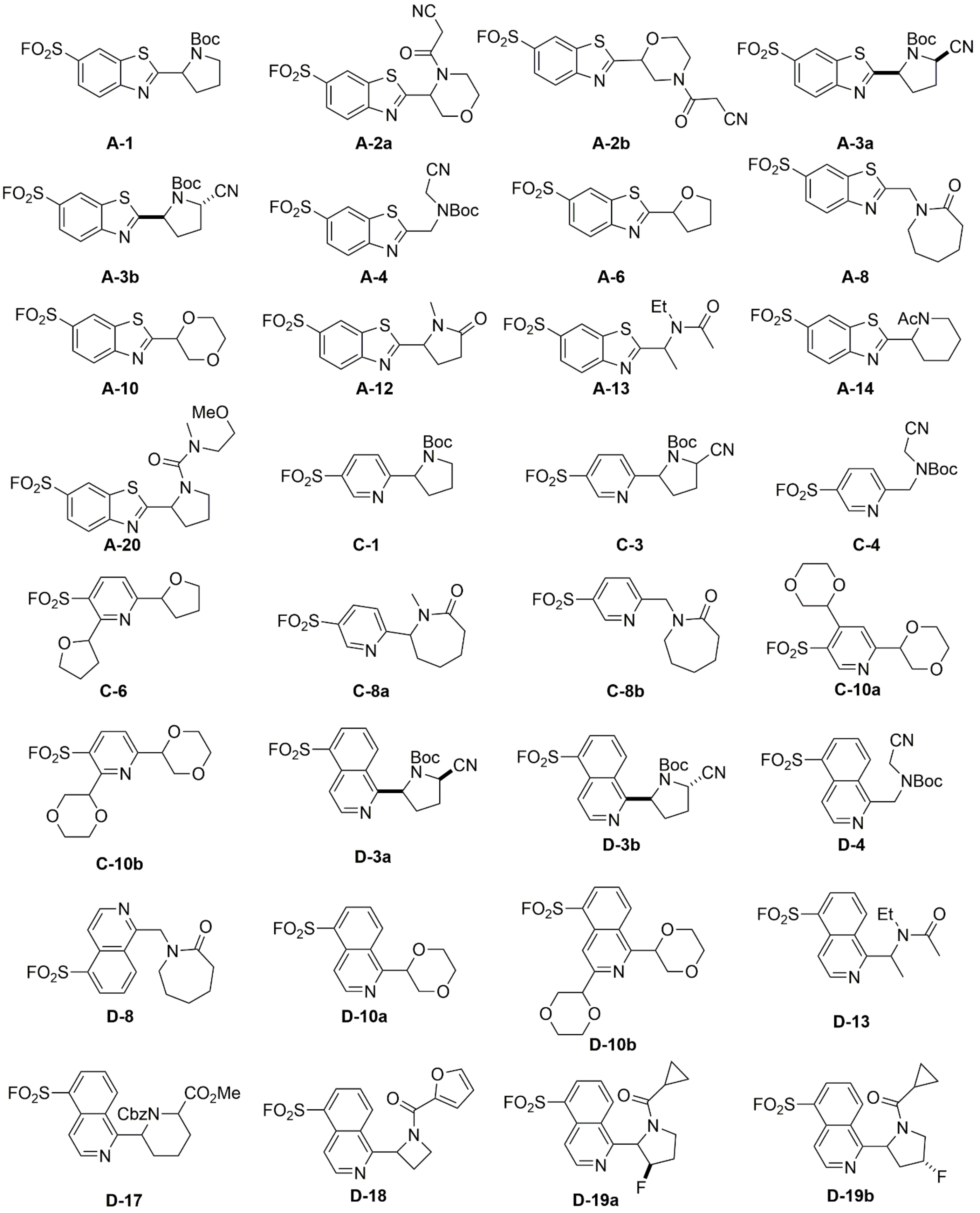

Figure S1. Chemical structures of the 32 sulfonyl fluoride compounds reported in this study.

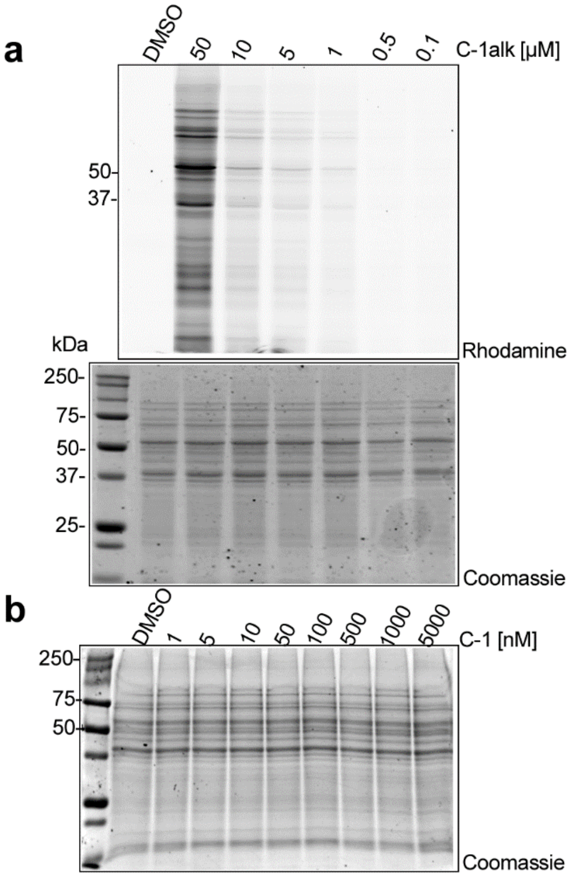

Figure S2. Reactivity profile for probes generated in this study. A) In-gel fluorescence scanning analysis of proteins following incubation of varying concentrations of **C-1alk** on HeLa cell lysates and click reaction with 100 μM rhodamine-N_3. Lower panel shows the protein loading control after Coomassie gel staining. B) Full gel image for the Coomassie gel staining presented in Fig. 5B.

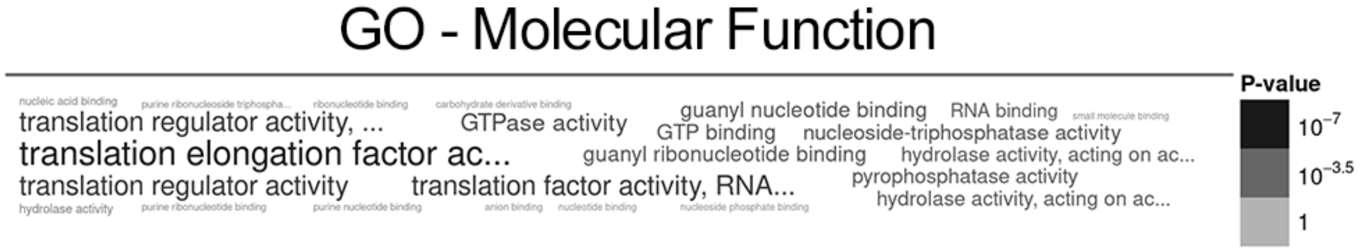

Figure S3. Gene Ontology (GO) enrichment analysis for **C-1 alk** enriched protein hits. The 95 protein hits identified in pulldown experiments using **C-1alk** probe were queried against *T. brucei* proteome dataset and restricted to a GO search associated to molecular function terms. GO-MF search was set to *P*-value = 0.01.

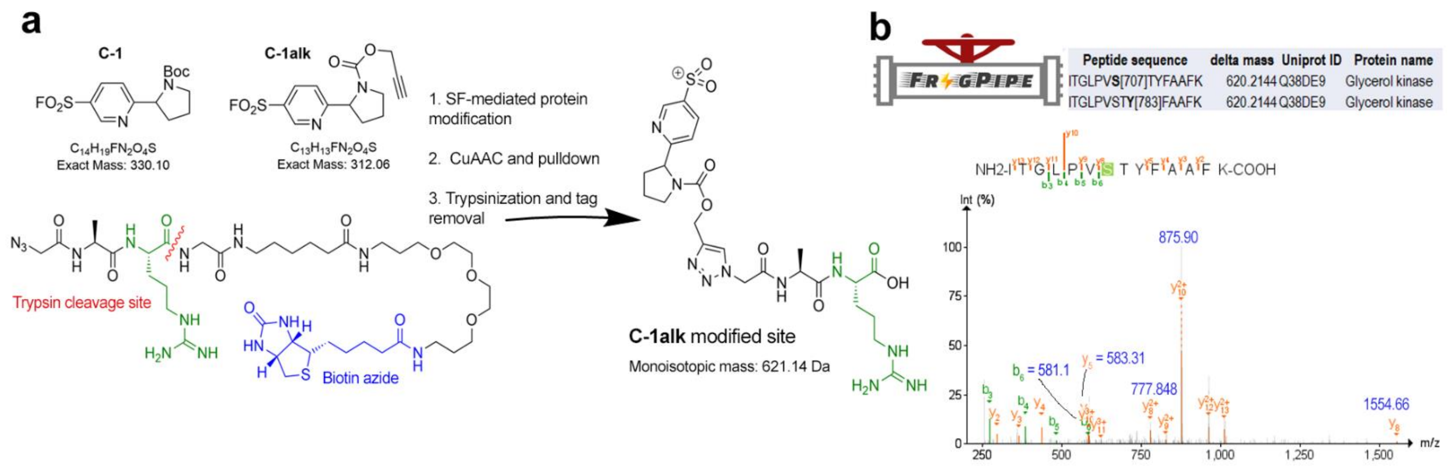

Figure S4. Sulfonyl fluoride mediated site ID. **a)** Potential **C-1 alk** modified peptide(s) were identified based on the reaction product predicted mass of +620.21 Da. Our biotin-azide reagent contains a trypsin cleavable site thus releasing the affinity tag. The modified peptide will contain the **C-1** analogue ligand where a proton loss is predicted to occur on the reactive site. **b)** An offset mass search was performed with our **C-1alk** pulldown TIMS-TOF proteomic dataset in the FragPipe software. Information retrieved from the ptm.csv output shows the modified peptide and the protein ID of the identified glycerol kinase. MS spectrum obtained from the **C-1alk** modified peptide from *T. brucei* glycerol kinase (Q38DE9). The *m/z* values for the relevant ions (blue) show the expected mass shift on y_8, y_8^{++} and b_6 ions as compared to that on y_5 ion (δ mass = 1554.66 [y_8] – 583.31 [y_5] – 369.15 [STY] + 18 [H_2O]).

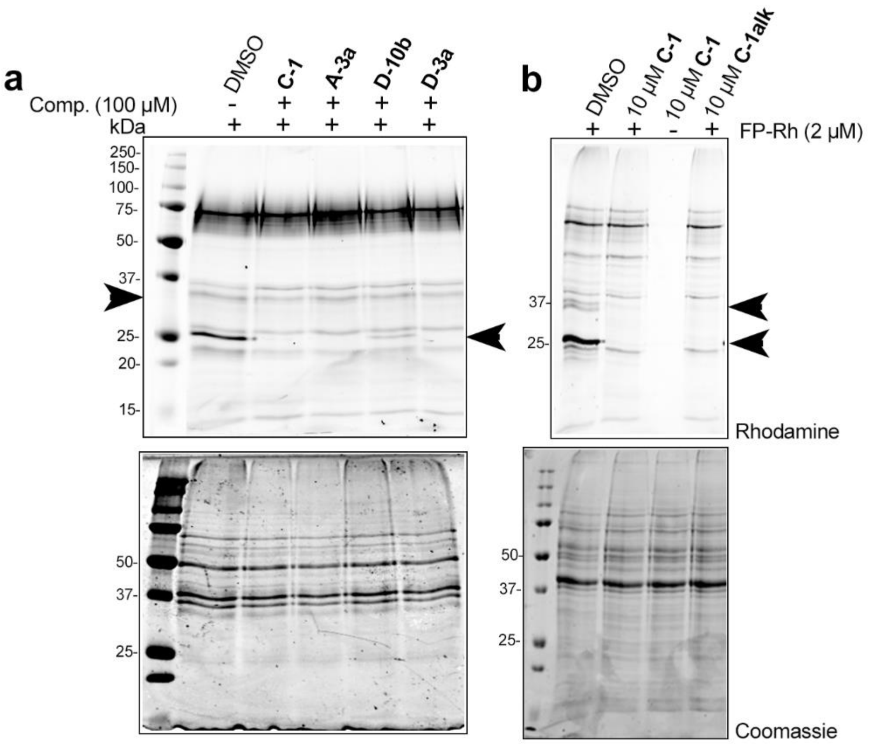

Figure S5. **a**) In-gel analysis of proteins following incubation of *T. brucei* lysates with 100 µM probe (or DMSO control), and treatment with 2 µM FP-Rh. Examples of proteins that were targeted by specific probes are indicated with pink and blue arrows. **b**) Effect of **C-1** and its alkyne analogue on the reactivity of a fluorophosphonate probe. *T. brucei* lysates were pre-incubated with 10 µM of each compound or DMSO (control) followed by addition of FP-Rh. Proteins were resolved by SDS-PAGE and fluorescently labelled proteins were imaged under the rhodamine detector. Coomassie staining was used as load control.

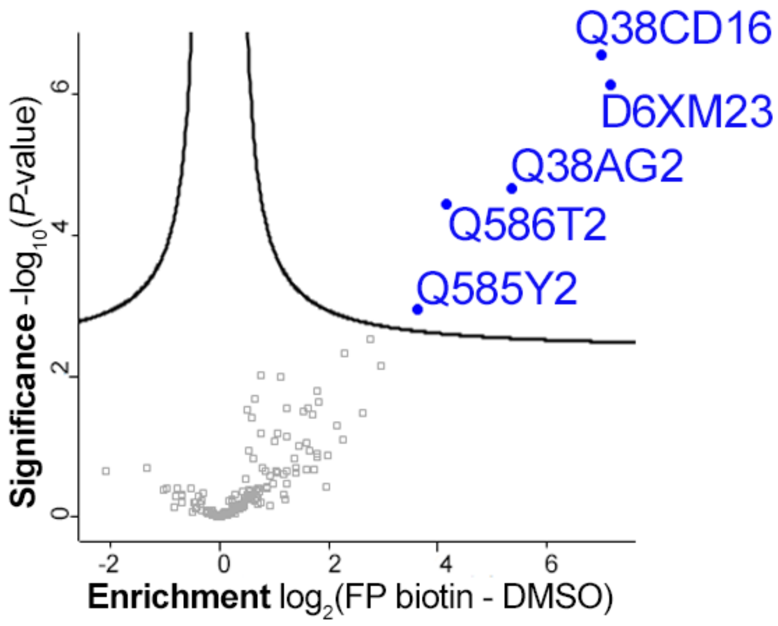

Uniprot ID	Protein name	Mass (Da)
D6XM23	Lysophospholipase, AB_hydrolase	30,092
Q38AG2	Prolyl oligopeptidase S9	77,625
Q38CD6	Carboxypeptidase S10	51,510
Q585Y2	Reticulon-like protein	21,071
Q586T2	Esterase, lipid hydrolase	27,191

Figure S6. Affinity binding proteome profiling using a fluorophosphonate probe (**FP-biotin**) in *T. brucei*. Parasite lysates were probed with 4 µM of FP-biotin and proteins were pulled down using streptavidin coated beads. Scatter plot shows the significantly enriched protein hits (blue dots) against the DMSO background control. Values in plot (164 total number of proteins) were the resultant of two biological replicates injected (n=3) separately. Statistical *t*-test was performed using 250 randomizations with a false discovery rate = 0.05 and s0 = 0.2.

S1 Table. Yield of the products of photoredox-catalysed dehydrogenative couplings

Hetarene	Hydrogen donor	Product(s)[a]	Yield[b]
**HA A**	**HD 1**	**A-1**	30
**HA A**	**HD 2**	**A-2a**	25
		**A-2b**	13
**HA A**	**HD 3**	**A-3a**	7
		**A-3b**	6
**HA A**	**HD 4**	**A-4**	8
**HA A**	**HD 6**[c]	**A-6**	15
**HA A**	**HD 8**	**A-8**	19
**HA A**	**HD 10**[c]	**A-10**	26
**HA A**	**HD 12**	**A-12**	25
**HA A**	**HD 13**	**A-13**	15
**HA A**	**HD 14**	**A-14**	24
**HA A**	**HD 20**	**A-20**	18
**HA C**	**HD 1**	**C-1**	2
**HA C**	**HD 3**	**C-3**	11
**HA C**	**HD 4**	**C-4**	9
**HA C**	**HD 6**[c]	**C-6**	4
**HA C**	**HD 8**	**C-8a**	8
		**C-8b**	17
**HA C**	**HD 10**[c]	**C-10a**	11
		**C-10b**	10
**HA D**	**HD 3**	**D-3a**	13
		**D-3b**	12
**HA D**	**HD 4**	**D-4**	11
**HA D**	**HD 8**	**D-8**	22
**HA D**	**HD 10**[c]	**D-10a**	9

		**D-10b**	3
**HA D**	**HD 13**	**D-13**	8
**HA D**	**HD 17**	**D-17**	7
**HA D**	**HD 18**	**D-18**	16
**HA D**	**HD 19**	**D-19a**	3
		**D-19b**	5

[a] Reaction conditions: hetarene (100 mM), hydrogen donor (500 mM), TBPA (500 mM), TFA (200 mM), Ir[dF(CF_3)ppy]$_2$(dtbpy)PF_6 (1 mM), light. [b] Yield after purification by mass-directed HPLC. [c] Concentration of hydrogen donor: 15 M.

S2 Table. Characteristic ^{1}H NMR spectroscopic data for coupled products derived from **HA C**

	2-H / ppm	6-H / ppm	4-H / ppm	5-H / ppm
**HA C**	9.24	9.01	8.31	7.61
**C-1**	9.11	*a*	8.21	7.47
**C-3**	9.15,[b] 9.09	*a*	8.29, 8.24[b]	7.60, 7.52[b]
**C-4**	9.14, 8.89[b]	*a*	8.38,[b] 8.29	7.59-7.51[c]
**C-6**	*a*	*a*	8.28	7.61
**C-8a**	9.12	*a*	8.22	7.43
**C-8b**	9.10	*a*	8.21	7.59
**C-10a**	*a*	*a*	8.38	7.74
**C-10b**	9.09	*a*	*a*	8.04

[a]Substituted at this position. [b]Minor rotomer. [c]Multiplet.

S3 Table. Characteristic ^{1}H NMR spectroscopic data for coupled products derived from **HA D**

	1-H / ppm	3-H / ppm	8-H / ppm	6-H / ppm	4-H / ppm	7-H / ppm
**HA D**	9.40	8.78	8.54	8.39	8.28	7.77
**D-2**	*a*	8.86, 8.78[b]	8.73-8.69[c]	8.58, 8.53[b]	8.36, 8.28[b]	7.84-7.79[c]
**D-3a**	*a*	8.63	8.66	8.58	8.25	7.79
**D-3b**	*a*	8.65	8.57	8.57	8.24	7.81
**D-4**	*a*	8.89, 8.80[b]	8.72	8.61	8.39, 8.32[b]	7.89-7.83[c]
**D-8**	*a*	9.03	8.70	8.57	8.33	7.82
**D-10a**	*a*	8.84	8.76	8.56	8.25	7.78
**D-10b**	*a*	*a*	8.68	8.54	8.36	8.23
**D-13**	*a*	8.89	8.75	8.55	8.30	7.80
**D-17**	*a*	8.72	8.55	8.39	8.19	7.82
**D-18**	*a*	8.93	8.84	8.58	8.29	7.81
**D-19a**	*a*	8.78	8.64	8.60	8.31	7.83
**D-19b**	*a*	8.99	8.72	8.59	8.28	7.83

[a]Substituted at this position. [b]Minor rotomer. [c]Multiplet.

S4 Table. EC_{50} values of the sulfonyl fluoride probes.

Probe	*T. brucei*[a] EC$_{50}$ / µM (pEC$_{50}$)	HeLa[b] EC$_{50}$ / µM	Fold selectivity[c]
**HA A**	111 ± 3 (3.95)		
**HA C**	154 ± 2 (3.81)		
**HA D**	124 ± 3 (3.90)		
**A-3a**	0.50 ± 0.01 (6.30)	18 ± 2	37
**A-3b**	2.82 ± 0.06 (5.55)	72 ± 5	26
**C-1**	0.31 ± 0.01 (6.50)	13 ± 2	41
**C-3**	6.36 ± 0.07 (5.19)	102 ± 4	16
**C-4**	3.84 ± 0.04 (5.41)	83 ± 8	22
**D-3a**	0.62 ± 0.01 (6.21)	41 ± 6	66
**D-3b**	2.23 ± 0.03 (5.65)	67 ± 9	30
**D-10a**	4.68 ± 0.04 (5.32)	114 ±39	24
**D-10b**	0.62 ± 0.01 (6.21)	26 ± 4	42
**D-13**	7.18 ± 0.05 (5.14)	85 ± 6	12

[a] Activity against *Trypanosoma brucei*. [b] HeLa cell toxicity. [c] Fold selectivity over HeLa toxicity.

Tables provided as separate Excel files

S5 Table. List of *T. brucei* protein hits identified with the **C-1alk** probe

S6 Table. List of *T. brucei* protein hits identified with the **C-1alk** probe following pre-treatment with the **C-1** parent compound.

S7 Table. List of *T. brucei* protein hits identified with the commercial probe **FP biotin**.

S8 Table. Summary of protein hits obtained across all experimental conditions assayed.

Supplementary Methods

General Experimental

Commercially available starting materials were purchased from SigmaAldrich, Fluorochem and Alfa Aesar. Reactions were carried out under nitrogen atmosphere unless stated otherwise. Anhydrous solvents for most reactions were obtained from a PureSolv MDS Purification System; anhydrous 1,4-dioxane and ethyl acetate were obtained from SureSeal bottles. All other solvents and reagents were of analytical grade and used as supplied. Solvents were removed using a reduced pressure, Büchi rotary evaporator and a Vacuubrand PC2001 Vario Diaphragm pump.

Thin layer chromatography was carried out with commercial, aluminium backed silica plates (Merck silica 2880 gel, 60 F254) and visualised using an ultraviolet lamp (λ_{max} = 254 nm). Flash column chromatography was carried out with silica gel 60 (35-75 µm particles).

Analytical LC-MS was performed using Ultimate3000 HPLC, with a UV diode array detector and a MS detector Bruker Amazon Speeds with electrospray ionisation run positive and negative switching mode. This used a Phenomenex Kinetex C_{18} 2.1 × 50 mm 2.6 micron column and two solvent systems: MeCN/H_2O + 0.1% formic acid or MeCN/H_2O. Accurate masses were obtained with a Bruker MaXis Impact with electrospray ionisation in positive mode. Purifications by mass-directed HPLC was carried out using a Agilent 1290 Infinity with Diode Array Detection and an Ascentis Express C_{18} Column, 50 × 2.1 mm, 2.7 µm particle size.

Infrared analysis was performed using a Perkin-Elmer One FT-IR spectrometer. Unless otherwise stated, ^{1}H, ^{13}C and ^{19}F NMR spectra were acquired at 300 K on Bruker 300, 400 or 500 MHz NMR spectrometers; ^{13}C and ^{19}F NMR spectra were proton-decoupled. Chemical shifts (δ) are stated in parts per million (ppm) and referenced to the residual solvent peak trimethylsilane (0 ppm) and coupling constants (*J*) are reported in Hertz (Hz); trifluoroacetic acid was often used as an internal standard for ^{19}F NMR spectroscopy. The splitting patterns of ^{1}H NMR data is reported: s (singlet), d (doublet), dd (doublet of doublets, t (triplet), m (multiplet). Assignment of signals were often aided by COSY, DEPT and HMQC spectra.

All buffers were prepared in 18.2 MΩ H_2O to the final volume stated. The pH of the solutions was adjusted using 1 M NaOH or 5 M HCl. Thermo Electron Corporation Holten LaminAir laminar flow cabinet was used to maintain a sterile environment when necessary. Centrifugation was performed using either a Heraeus multifuge 3 S-R centrifuge or a Heraeus Fresco-17 centrifuge. SDS-PAGE was carried out using a BioRad Mini-PROTEAN Tetra Cell system and a BioRad Power PAC 1000.

A BioRad ChemiDoc MP Imaging System was used to image polyacrylamide gels using a combination of UV and white light (Rhodamine- DyLight 550 602/50 green epifluorescence, Fluorescein- DyLight 488 532/28, Coomassie Blue- 715/30 far red epifluorescence).

General Procedures

A: Synthesis of hetaryl sulfonyl fluorides

The relevant hetarene sulfonic acid was dissolved in thionyl chloride to a final concentration of 1 M with 0.1 eq. DMF. The solution was sealed in a crimped vial and heated to reflux at 75 °C for 2 hr. Solvent was removed *in vacuo* and the crude product resuspended in DCM and washed with saturated $NaHCO_3$. The organic layer was dried with Na_2SO_4 and concentrated *in vacuo* to afford the sulfonyl chloride. Without further purification, the hetarene sulfonyl chloride was dissolved in acetonitrile (1 mM, 1 eq) and added to a suspension of potassium hydrogenfluoride (KHF_2) in water (4.5 mM, 2.3 eq), stirring for 12 hours at RT. The reaction mixture was diluted in water (5 ´ volumes) and the product extracted with DCM three times. The combined extracts were washed once with a 10% solution of $NaHCO_3$, and saturated NaCl before drying (Na_2SO_4) and concentrated *in vacuo* to give the sulfonyl fluoride which was used without further purification.

B: Synthesis of sulfonyl fluoride probes by dehydrogenative coupling

To a LC-MS vial as acetone stock solutions, a functionalised hetarene (20.0 µL of a 1.5 M stock) was added, followed by a hydrogen donor (33.3 µL of a 1.5 M stock), TFA (10.0 µL of a 2 M stock), $(Ir[dF(CF_3)ppy]_2(dtbpy))PF_6$ (10.0 µL of a 0.01 M stock, CAS: 870987-63-6) and *t*-butyl peracetate (15.9 µL of a 50% w/v solution) were added along with acetone (10.7 µL) to a total volume of 100 µL, giving final concentrations of hetarene (100 mM), hydrogen donor (500 mM), TFA (200 mM), catalyst (1 mM) and *t*-butyl peracetate (500 mM). The reaction vials were sealed and stirred on a fan-cooled HepatoChem lightbox with irradiation under a 390 nm Kessil Lamp for 24 hrs. The mixtures were evaporated to give crude products. Crude reaction products were analysed using LC-MS, and 500 MHz ^1H and 565 MHz ^{19}F NMR spectroscopy. For promising reactions, the crude reaction mixture was dissolved in acetonitrile and purified via mass-directed HPLC (gradient elution: 5:95 → 95:5 MeCN:water); eluted samples were lyophilised to give the pure product.

Compound synthesis and characterisation

1,3-Benzothiazole-6-sulfonyl fluoride HA A

Following the second half of General Procedure A, 1,3-benzothiazole-5-sulfonyl chloride (500 mg, 2.1 mmol) was reacted to form the sulfonyl fluoride **HA A** as a yellow amorphous solid (347 mg, 75%). δ_H (500 MHz, $CDCl_3$) 9.33 (1H, s, 2-H), 8.71 (1H, d, *J* 1.9, 7-H), 8.36 (1H, d, *J* 8.7, 4-H), 8.14 (1H, dd, *J* 8.7, 1.9, 5-H). δ_C (125 MHz, $CDCl_3$) 159.9 (2-C), 157.4 (7a-C), 134.8 (3a-C), 130.0 (1C, d, *J* 25.2, 6-C), 125.7 (5-C) 125.1 (4-C). 124.1 (7-C). δ_F (565 MHz, $CDCl_3$) 68.2. λ_{max} (neat)/cm^{-1} 3091, 3069, 1405, 1200, 1017. HRMS (ESI) $C_7H_4NO_2S_2$ requires [M+H]+, calculated 217.9746, found 217.9740.

Pyridine-3-sulfonyl fluoride HA C

Following the second half of General Procedure A, pyridine-3-sulfonyl chloride (500 mg, 2.82 mmol) was reacted to form the sulfonyl fluoride **HA C** as a clear oil (392 mg, 86%). δ_H (500 MHz, $CDCl_3$) 9.24 (1H, appt t, *J* 1.6, 2-H), 9.01 (1H, dd, *J* 4.9, 1.6, 6-H), 8.31 (1H, ddd, *J* 8.2, 2.5, 1.6, 4-H), 7.61 (1H, dd, *J* 8.2, 4.9, 5-H). δ_C (125 MHz, $CDCl_3$) 156.1 (6-C), 149.2 (2-C), 136.2 (4-H), 130.4 (1C, d, *J* 25.5, 3-C), 124.3 (5-H). δ_F (565 MHz, $CDCl_3$) 69.2. λ_{max} (neat)/cm^{-1} 3071, 1572, 1406, 1209, 1111. HRMS (ESI) Unable to acquire.

Isoquinoline-5-sulfonyl fluoride HA D

Following General Procedure A, isoquinoline-5-sulfonyl chloride (500 mg, 2.37 mmol) was reacted to form the sulfonyl fluoride **HA D** as a colourless amorphous solid (254 mg, 55%). δ_H (500 MHz, $CDCl_3$) 9.40 (1H, d, *J* 1.2, 1-H), 8.78 (1H, d, *J* 6.1, 3-H), 8.54 (dt, *J* 8.3, 1.2, 8-H), 8.39 (1H, dd, *J* 8.3, 1.2, 6-H), 8.28 (1H, ddd, *J* 6.1, 2.6, 1.2, 4H), 7.77 (1H, td, *J* 7.8, 1.2, 7-H). δ_C (125 MHz, $CDCl_3$) 153.4 (1-C), 146.5 (3-C), 136.5 (8-C), 135.0 (7-C), 131.5 (4a-C),128.7 (8a-C), 128.6 (1C, d, *J* 24.8, 5-C), 126.0 (6-C), 116.7 (4-C). δ_F (565 MHz, $CDCl_3$) 64.9. v_{max} (neat)/cm^{-1} 3061, 2982, 1491, 1400, 1319, 1078. HRMS (ESI) $C_9H_6FNO_2S$ requires $[M+H]^+$, calculated 212.0182, found 212.0176.

Quinoline-5-sulfonyl fluoride HA E

Following General Procedure A, quinoline-6-sulfonic acid (1.00 g, 4.74 mmol) was reacted to form the sulfonyl fluoride **HA E** as a cream amorphous solid (236 mg, 26%). δ_H (500 MHz, $CDCl_3$) 9.19 (1H, dd, *J* 4.3, 1.8, 2-H), 8.53 (1H, dd, *J* 7.4, 1.4, 6-H), 8.32 (1H, dd, *J* 8.3, 1.8, 4-H), 8.24 (1H, dd, *J* 8.2, 1.4, 8-H), 7.72 (1H, ddd, *J* 8.5, 7.5, 1.3, 7-H), 7.64 (1H, dd, *J* 8.3, 4.3, 3-H). δ_C (125 MHz, $CDCl_3$) 152.7 (2-C), 143.8 (4a-C), 136.6 (4-C), 136.1 (8-C), 133.1 (6-C), 131.4 (1C, d, *J* 21.0, 5-C), 129.1 (8a-C), 125.3 (7-C), 123.0 (3-C). δ_F (565 MHz, $CDCl_3$) 65.3. λ_{max} (neat)/cm^{-1} 3050, 1491, 1399, 1197, 1156. HRMS (ESI) $C_9H_6FNO_2S$ requires $[M+H]^+$, calculated 212.0182, found 212.0173.

1-Methylpyrazole-4-sulfonyl fluoride HA F

Following the second half of General Procedure A, 1-methylpyrazole-4-sulfonyl chloride (500 mg, 3.05 mmol) was reacted to form the sulfonyl fluoride **HA F** as a colourless amorphous solid (300 mg, 67%). δ_H (500 MHz, $CDCl_3$) 8.02 (1H, s, 5-H), 7.94 (1H, s, 3-H), 4.01 (3H, s, Me). δ_C (125 MHz, $CDCl_3$) 140.0 (3-C), 133.9 (5-C), 144.6 (1C, d, *J* 31.4, 4-C), 40.1 (Me). δ_F (565 MHz, $CDCl_3$) 71.4. λ_{max} (neat)/cm^{-1} 2136, 2958, 1525, 1410, 1389, 1068. HRMS (ESI) Unable to acquire.

***N*-Boc-pyrrolidine HD 1**

Di-tert-butyl dicarbonate (6.98 g, 54 mmol) was dissolved in DCM (250 mL, 0.2 M) with DMAP (659 mg, 5.4 mmol) and cooled to 0 °C. Pyrrolidine (5 mL, 60 mmol) was added dropwise via an addition funnel before warming to room temperature and stirring overnight for 24 hours, with consumption of starting material shown by TLC. The solution was then washed with brine (2 × 50 mL) and water (2 × 50 mL) before drying with Na_2SO_4 and concentrated *in vacuo.* The crude product was purified through a silica plug, eluting with DCM to give *N*-Boc-pyrrolidine, **HD 1** as a colourless oil (8.23 g, 81%). δ_H (500 MHz, $CDCl_3$) 3.30 (2H, t, *J* 6.2, 2- and 5-H_a), 3.24 (2H, t, *J* 6.2, 2- and 5-H_b), 1.83-1.80 (4H, m, 3- and 4-H_2), 1.44 (9H, s, ^{t}Bu 2-H_3). δ_C (125 MHz, $CDCl_3$) 154.8 (^{t}Bu C=O), 79.0 (^{t}Bu C_1), 46.1 (2- or 5-C), 45.7 (2- or 5-C), 28.7 (^{t}Bu C_3), 25.9 (3- or 4-C), 25.1 (3- or 4-C). R_F 0.42 (DCM). λ_{max} (neat)/cm^{-1} 2974, 2876, 1692. HRMS (ESI) $C_9H_{17}NO_2$ requires $[2M+Na]^+$, calculated 365.2416 found 365.2413.

4-Acetyl-thiomorpholine HD 5

Thiomorpholine (500 mg, 4.85 mmol), was dissolved in dry DCM (20 mL) and cooled to 0 °C on ice, to which triethylamine (850 µL, 5.50 mmol) was added and the solution stirred. Acetyl chloride (3.15 mL, 4.4 mmol) was added dropwise to the solution, and stirred for 1 hour before warming to room temperature overnight, until consumption of acetyl chloride was shown by TLC. The reaction mixture was then washed with brine (2 × 10 mL) and water (2 × 10 mL), dried with Na_2SO_4 and concentrated *in vacuo.* The crude product was purified through a silica plug, eluting with DCM to give 4-acetyl-thiomorpholine, **HD 5** as a yellow oil (332 mg, 52%). δ_H (500 MHz, $CDCl_3$) 3.84 (2H, t, *J* 5.2, 3- and 5-H_a), 3.70 (2H, t, *J* 5.2, 3- and 5-H_b), 2.61-2.57 (4H, m, 2- and 6-H_2), 2.07 (3H, s, Ac 2-H_3). δ_C (125 MHz, $CDCl_3$) 169.0 (Ac C=O), 49.1 (3- or 5-C), 44.1 (3- or 5-C), 27.9 (2- or 6-C), 27.4 (2- or 6-C), 21.7 (Ac 2-C). λ_{max} (neat)/cm-1 2910, 1628. R_F 0.51 (DCM). HRMS (ESI) $C_6H_{11}NOS$ requires $[M+Na]^+$, calculated 168.0459, found 168.0446.

Methyl 2-phenylpiperidine-1-carboxylate HD 16

2-Phenyl piperidine (500 mg, 3.20 mmol) was dissolved in dry DCM with triethylamine (500 µL, 3.55 mmol) and cooled to 0 °C on ice. Methyl chloroformate (225 µL, 2.91 mmol) was added dropwise, and the solution stirred for 1 hour before warming to room temperature overnight, until consumption of the chloroformate shown by TLC. The reaction mixture was then washed with brine (2 × 10 mL) and water (2 × 10 mL), dried with Na_2SO_4 and concentrated *in vacuo.* The crude product was purified through a silica plug to elute methyl 2-phenylpiperidine-1-carboxylate, **HD 16** as a colourless oil (427, 67%), δ_H (500 MHz, $CDCl_3$) 7.35 (2H, m, Ph 2- and 6-H), 7.24 (3H, m, Ph 3-, 4- and 5-H), 5.48 (1H, appt br. s, 2-H), 4.13-4.07 (1H, br. m, 6-H_a), 3.74 (3H, s, Me) 2.82 (1H, ddd, *J* 13.8, 10.4, 3.9, 3-H_a), 2.32 (1H, dd, *J* 10.4, 3.9, 6-H_b), 1.93-1.87 (1H, m, 3-H_b), 1.63-1.42 (4H, m, 3- and 4-H_2). δ_C (125 MHz, $CDCl_3$) 156.8 (C=O), 139.8 (Ph 1-C), 128.6 (Ph 3- and 5-C), 126.5 (Ph 2-, 4- and 6-C), 53.4 (2-C), 52.7 (Me), 40.4 (6-C), 28.1 (3-C), 25.5 (5-C), 19.3 (4-C). λ_{max} (neat)/cm^{-1} 3048, 3021, 2931, 2895, 1675, 1525, 1486. R_F 0.80 (DCM). HRMS (ESI) $C_{13}H_{17}NO_2$ requires $[M+Na]^+$, calculated 242.1157, found 242.1160.

1-Benzyl-2-methyl piperidine-1,2-dicarboxylate HD 17

1-Benzyloxy-2-piperidine carboxylic acid (500 mg, 1.9 mmol) was dissolved in methanol (1.9 mL) and sealed in a vial under nitrogen. The vial was cooled to 0 °C on ice, before thionyl chloride (179 µL, 2.5 mmol) was added dropwise, and the solution stirred for 1 hour. The reaction was them warmed to room temperature and stirred overnight. The reaction diluted in DCM (10 mL) and quenched with water (2 × 10 mL), dried with Na_2SO_4 and concentrated *in vacuo* to afford the pure methyl ester, **HD 17** as a colourless solid (444 mg, 84%, 50:50 Rot_a:$Rot_{b)}$. δ_H (500 MHz, $CDCl_3$) 7.38-7.35 (2H, m, Ph 2- and 6-H), 7.32-7.29 (3H, m, Ph 3-, 4- and 5-H), 5.16 (2H, s, Cbz CH_2), 4.97-4.94 (1H, br. m, 2-H_{rotA}), 4.86-4.84 (1H, br. m, 2-H_{rotB}), 4.11 (1H, appt d, *J* 11.0, 6-Ha_{rotA}), 4.06 (1H, appt d, *J* 11.0, 6-Ha_{rotB}), 3.74 (3H, s, Me_{rotA}), 3.68 (3H, s, Me_{rotB}), 3.06 (1H, appt t, *J* 13.0, 6-Hb_{rotA}), 2.96 (1H, appt t, J 13.0, 6-Hb_{rotA}), 2.25-2.19 (1H, m, 3-H_a), 1.70-1.61 (4H, m, 3H_b 4-H_a and 5-$H_{a/b}$), 1.46-1.40 (1H, m, 4-H_b), 1.30-1.24 (1H, m 4-H_b). δ_C (125 MHz, $CDCl_3$) 172.3 (Ester C=O), 156.6 (Cbz C=O_a), 156.1 (Cbz C=O_b), 136.8 (Ph 1-C), 128.6 (Ph 3- and 5-C), 128.0 (Ph 2-, 4- and 6-C), 67.5 (Cbz 1-C_a), 67.3 (Cbz 1-C_b), 54.8 (2-C_a), 54.5 (2-C_b), 52.3 (Me), 42.0 (6-C_a), 41.9 (6-C_b), 26.9 (3-C_a), 26.8 (3-C_b), 24.9 (5-C_a) 24.7 (5-C_b), 20.9 (4-C_a), 20.8 (4-C_b). λ_{max} (neat)/cm^{-1} 3032, 2946, 2860, 1739, 1697, 1586, 1498. R_F 0.80 (10:90 EtOAc–DCM). HRMS (ESI) $C_{15}H_{19}NO_4$ requires $[M+Na]^+$, calculated 300.1212, found 300.1205.

1-(Furan-2-carbonyl)azetidine HD 18

Azetidine hydrogenchloride (500 mg, 5.34 mmol) was dissolved in DCM with triethylamine (1.60 mL, 11.40 mmol) and cooled to 0 °C on ice. 2-Furoyl chloride (480 µL, 4.86 mmol) was added dropwise,

and the solution stirred for 1 hour before warming to room temperature overnight, until consumption of the chloroformate shown by TLC. The reaction mixture was then washed with brine (2 × 10 mL) and water (2 × 10 mL), dried with Na_2SO_4 and concentrated *in vacuo.* The crude product was purified via column chromatography, eluting with a gradient of 0 – 50% EtOAc in DCM to give the amide, **HD 18** as a colourless amorphous solid (351 mg, 44%). δ_H (500 MHz, $CDCl_3$) 7.46 (1H, dd, *J* 1.8, 0.9, 3-H), 7.00 (1H, dd, *J* 3.5, 0.9, 5-H), 6.45 (1H, dd, *J* 1.8, 3.5, 4-H), 4.51 (2H, t, *J* 7.8, 2-H_2), 4.17 (2H, t, *J* 7.8, 4-H_2), 2.36 (2H, p, *J* 7.8, 3-H_2). δ_C (125 MHz, $CDCl_3$) 158.9 (C=O), 148.2 (2-C), 144.4 (3-C), 115.2 (4-C), 111.5 (5-C), 52.6 (2-C), 48.6 (4-C), 16.5 (3-C). λ_{max} (neat)/cm^{-1} 3121, 3094, 3013, 2951, 2875, 1621, 1564, 1484, 1474, 1450. R_F 0.2 (50:50 DCM–EtOAc). HRMS (ESI) $C_8H_9NO_2$ requires $[2M+Na]^+$, calculated 325.1164, found 325.1161.

(3*S*)-1-Cyclopropanecarbonyl-3-fluoropyrrolidine HD 19

(3*S*)-Fluoropyrrolidine hydrogenchloride (500 mg, 3.98 mmol) was dissolved in dry DCM with triethylamine (1.14 mL, 8.15 mmol) and cooled to 0 °C on ice. Cyclopropane carbonyl chloride (328 µL, 3.62 mmol) was added dropwise, and the solution stirred for 1 hour before warming to room temperature overnight, until consumption of the chloroformate shown by TLC. The reaction mixture was then washed with brine (2 × 10 mL) and water (2 × 10 mL), dried with Na_2SO_4 and concentrated *in vacuo.* The crude product was purified through a silica plug to elute (3S)-1-cyclopropanecarbonyl-3-fluoropyrrolidine, **HD 19** as a yellow oil (412 mg, 66%, 50:50 mixture of rotamers). δ_H (500 MHz, $CDCl_3$) 5.29 (1H, ddt, *J* 13.5, 8.2, 3.0, 3-H), 3.90-3.50 (4H, m, 2- and 5-H_2), 2.40-1.90 (2H, m, 4-H_2), 1.63 (1H, m, prop 1-H_{Rota}), 1.53 (1H, m, 1-H_{Rotb}), 1.00 (2H, m, prop 2- and 3-H_a), 0.77 (2H, m, prop 2- and 3-H_b). δ_C (125 MHz, $CDCl_3$) 172.6 (C=O_a) 172.4 (C=O_b), 93.5 (1C, d, *J* 175.0, 3-C_a), 91.63 (1C, d, *J* 175.0, 3-C_b), 53.2 (1C, d, *J* 22.4, 2-C_a), 52.7 (1C, d, *J* 22.4, 2-C_b), 44.3 (5-C_a), 43.8 (5-C_b), 32.8 (1C, d, *J* 22.4, 4-C_a), 31.2 (1C, d, *J* 22.4, 4-C_b), 12.7 (prop 1-C_{Rota}), 12.6 (prop 1-C_{Rotb}), 7.9 (prop 2- or 3-C_a), 7.8 (prop 2- or 3-C_a), 7.6 (prop 2- or 3-C_b), 7.5 (prop 2- or 3-C_b). δ_F (565 MHz, $CDCl_3$) 177.0 (Rot_a), -177.8 (Rot_b). λ_{max} (neat)/cm^{-1} 2979, 2889, 1689, 1435. R_F 0.43 (50:50 DCM–EtOAc). HRMS (ESI) $C_8H_{12}FNO$ requires $[2M+Na]^+$, calculated 337.1704, found 337.1704.

***N*-(2-methoxyethyl)-*N*-methylpyrrolidine-1-carboxamide HD 20**

Pyrrolidine (355 mg, 5.00 mmol) was dissolved in water (10 mL) and cooled to 0 °C over ice. CDI (890 mg, 5.50 mmol) was added, and the solution stirred for 1 hour before warming to room temperature. Formation of the carbonylimidazolide was monitored by TLC. Once completed, (2-methoxyethyl)methylamine (650 µL, 6.00 mmol) and stirred for 2 hours. The coupled product was then extracted with ethyl acetate (3 × 5 mL) and concentrated *in vacuo* without further purification to afford the carboxamide, **HD 20** as a yellow oil (398 mg, 43%). δ_H (500 MHz, $CDCl_3$) 3.52 (2H, t, *J* 5.9, Et 2-H_2), 3.36 (2H, t, *J* 5.9, Et 1-H_2), 3.33-3.30 (7H, m, NMe and pyrrolidinyl 2- and 5-H_2), 2.87

(3H, s, OMe), 1.79 (4H, m, 3- and 4-H_2). δ_C (125 MHz, $CDCl_3$) 163.3 (C=O), 71.3 (Et 2-C), 58.9 (OMe), 49.7 (Et 1-C), 48.5 (pyrrolidinyl 2- and 5-C), 37.4 (NMe), 25.7 (pyrrolidinyl 3- and 4-C). λ_{max} (neat)/cm^{-1} 2979, 2889, 1689, 1435. R_F 0.27 (DCM). HRMS (ESI) $C_9H_{18}N_2O_2$ requires $[M+H]^+$, calculated 187.1447, found 187.1437.

2-(*N*-Boc-pyrrolidin-2-yl)-1,3-benzothiazole S1

Following General Procedure B, 1,3-benzothiazole (13.5 mg, 0.10 mmol) was reacted with *N*-boc-pyrrolidine (85.6 mg, 0.5 mmol) following purification by Mass-Directed HPLC from a 10 minute gradient of 35 – 95% acetonitrile in water, collecting at 8.5 mins to isolate the cross coupled product, as a pale brown amorphous solid (8.7 mg, 28%, 60:40 mixture of rotamers). δ_H (500 MHz, $CDCl_3$) 7.9 (1H, d, *J* 8.1, 7-H), 7.87-7.84 (1H, m, 4-H), 7.48-7.44 (1-H, m, 6-H), 7.38-7.34 (1H, m, 5-H), 5.24 (1H, br. s, pyrrolidinyl 2-H_{min}), 5.22 (1H, br. d, pyrrolidinyl 2-H_{maj}), 3.60-3.55 (2H, m, pyrrolidinyl 5-H), 2.43-2.33 (2H, m, pyrrolidinyl 3-H), 2.02-1.92 (2H, m, pyrrolidinyl 4-H), 1.49 (9H, s, ^{t}Bu 2-H_{min}) 1.30 (9H, s, ^{t}Bu 2-H_{maj}). δ_C (125 MHz, $CDCl_3$) 177.1 (^{t}Bu C=O_{maj}), 176.3 (^{t}Bu C=O_{min}) 162.0 (2-C), 154.9 (7a-C), 153.6 (3a-C), 126.1 (6-C), 124.9 (5-C), 122.8 (7-C), 121.9 (4-C), 80.5 (^{t}Bu C_{2maj}), 80.3 (^{t}Bu C_{2min}) 60.2 (pyrrolidinyl 2-C_{maj}), 59.7 (pyrrolidinyl 2-C_{min}), 47.2 (pyrrolidinyl 5-C_{min}), 46.9 (pyrrolidinyl 5- C_{maj}), 34.3 (pyrrolidinyl 3- C_{maj}), 33.1 (pyrrolidinyl 3- C_{min}), 28.6 (^{t}Bu C_{3min}), 28.4 (^{t}Bu C_{3maj}) 24.2 (pyrrolidinyl 4- C_{min}), 23.5 (pyrrolidinyl 4-C_{maj}). λ_{max} (neat)/cm^{-1} 3064, 2975, 2930, 2879, 1697, 1477, 1455, 1437. HRMS (ESI) $C_{16}H_{20}N_2O_2S$ requires $[M+H]^+$, calculated 212.0182, found 212.0176.

2-(*N*-Boc-pyrrolidin-2-yl)-1,3-benzothiazole-6-sulfonyl fluoride A-1

Following General Procedure B, 1,3-benzothiazole-6-sulfonyl fluoride (16.2 mg, 0.10 mmol) was reacted with *N*-Boc-pyrrolidine (85.6 mg, 0.5 mmol), following purification by Mass-Directed HPLC from a 15 minute gradient of 5 – 95% acetonitrile in water, collecting at 12.5 mins to isolate the cross coupled product as a colourless amorphous solid (9.9 mg, 25%, 50:50 mixture of rotamers). δ_H (500 MHz, $CDCl_3$) 8.58 (1H, appt br. d, *J* 17.5, 7-H), 8.14 (1H, appt br. d, *J* 9.0, 4-H), 8.06 (1H, m, 5-H), 5.28 (1H, appt br. s, pyrrolidinyl 2-H_{RotA}), 5.26 (1-H, appt br. s, pyrrolidinyl 2-H_{RotB}), 3.60-3.54 (2H, m, pyrrolidinyl 5-H_2), 2.45-2.25 (2H, m, pyrrolidinyl 3-H_2), 2.05-1.95 (2H, m, pyrrolidinyl 4-H_2), 1.49 (9H, s, ^{t}Bu 2-$H_{3\ RotA}$), 1.31 (9H, s, ^{t}Bu 2-H_{RotB}). δ_C (125 MHz, $CDCl_3$) 184.1 (^{t}Bu C=O_{maj}), 183.4 (^{t}Bu C=O_{min}) 158.1 (2-C), 155.0 (7a-C), 135.6 (3a-C), 128.9 (6-C), 125.6 (5-C), 124.1 (7-C), 123.7 (4-C), 84.6 (^{t}Bu 1-C_{RotA}), 81.0 (^{t}Bu 1-C_{RotB}) 60.4 (pyrrolidinyl 2-C_{RotA}), 60.0 (pyrrolidinyl 2-C_{RotB}), 47.4 (pyrrolidinyl 5-C_{RotA}), 47.0 (pyrrolidinyl 5-C_{RotB}), 34.3 (pyrrolidinyl 3-C_{RotA}), 33.0 (pyrrolidinyl 3-C_{RotB}), 28.5 (^{t}Bu 3-C_{RotA}), 28.4 (^{t}Bu 3-C_{RotB}) 24.4 (pyrrolidinyl 4-C_{RotA}), 23.6 (pyrrolidinyl 4-C_{RotB}). δ_F (565 MHz, $CDCl_3$) 67.1. λ_{max} (neat)/cm^{-1} 3066, 2976, 2931, 1693, 1507, 1477, 1409, 1208, 1116. HRMS (ESI) $C_{16}H_{19}F\ N_2O_4S_2$ requires $[M+Na]^+$, calculated 409.0668, found 409.0675.

2-[*N*-(2-Cyanoacetyl)morpholin-3-yl]-1,3-benzothiazole-6-sulfonyl fluoride A-2a

Following General Procedure B, 1,3-benzothiazole-6-sulfonyl fluoride (21.7 mg, 0.10 mmol) was reacted with 4-cyanoacetylmorpholine (77.1 mg, 0.5 mmol), following purification by Mass-Directed HPLC from a 20 minute gradient of 25 – 40% acetonitrile in water, isolating two cross coupled products, initially collecting at 13.0 mins, **1-2a** as a colourless amorphous solid (2.0 mg, 5%, 65:35 mixture of rotamers). δ_H (500 MHz, $CDCl_3$) 8.64 (1H, appt br. s, 7-H_{min}), 8.60 (1H, d, *J* 1.9, 7-H_{maj}), 8.24 (1H, appt d *J* 8.4, 4-H_{min}), 8.23 (1H, appt d *J* 8.4, 4-H_{maj}), 8.14 (1H, br. d *J* 8.7, 5-H_{min}), 8.10 (1H, dd *J* 1.9, 8.4, 5-H_{maj}), 5.93 (1H, s, morph 3-H_{maj}), 5.19 (1H, s, morph 3-H_{min}), 4.75 (1H, appt d, *J* 12.2, morph 5-Ha_{maj}), 4.52 (1H, appt d, *J* 12.2, morph 5-Ha_{min}), 4.31 (1H, appt d, *J* 12.1, morph 5-Hb_{min}), 4.09 (1H, appt d, *J* 12.1, 5-Hb_{maj}), 4.01-3.60 (4H, m, morph 2- and 6-H_2). δ_C (125 MHz, $CDCl_3$) 174.5 (C=O_{maj}), 173.0 (C=O_{min}), 162.5 (2-C_{min}), 161.9 (2-C_{maj}), 157.2 (7a-C_{maj}), 156.6 (7a-C_{min}), 136.3 (3a-C_{maj}), 136.2 (3a-C_{min}), 129.8 (1C, d, *J* 25.5, 6-C), 126.2 (5-C_{min}), 125.9 (5-C_{maj}), 125.0 (4-C_{min}), 124.8 (4-C_{maj}), 123.9 (7-C_{min}), 123.8 (7-C_{maj}), 113.7 (CN_{min}), 113.1 (CN_{maj}), 69.0 (morph 2-C_{maj}), 68.5 (morph 2-C_{min}), 66.5 (morph 6-C_{min}), 66.9 (morph 6-C_{maj}), 56.2 (morph 3- or 5-C_{min}), 52.9 (morph 3- or 5-C_{maj}), 43.9 (morph 3- or 5-C_{maj}), 39.3 (morph 3- or 5-C_{min}), 25.3 (acetyl 2-C_{min}), 25.1 (acetyl 2-C_{maj}). δ_F (565 MHz, $CDCl_3$) 67.8. λ_{max} (neat)/cm^{-1} 3047, 2950, 1725, 1669, 1409, 1198, 1043. HRMS (ESI) $C_{14}H_{12}FN_3O_4S_2$ requires $[M+Na]^+$, calculated 392.0151, found 392.0141.

Also obtained was 2-[*N*-(2-cyanoacetyl)morpholin-2-yl]-1,3-benzothiazole-6-sulfonyl fluoride **A-2b** as a colourless amorphous solid (4.9 mg, 13%, 70:30 mixture of rotamers). δ_H (500 MHz, $CDCl_3$) 8.68-8.68-8.62 (1H, br. m, 7-H), 8.24-8.20 (1H, br. m, 4-H), 8.14-8.10 (1H, br. m, 5-H), 5.04 (1H, dd, *J* 7.9, 3.3, morph 2-H_{maj}), 4.95 (1H, dd, *J* 7.9, 3.3, morph 2-H_{min}), 4.18-4.12 (2H, m, morph 3-H_2), 3.92-3.86 (2H, m, morph 5- and 6-Ha), 3.76 (2H, s, acetyl 2-H_2), 3.58-3.45 (2H, m, morph 5- and 6-Hb). δ_C (125 MHz, $CDCl_3$) 175.8 (C=O_{maj}), 175.1 (C=O_{min}), 160.8 (2-C_{maj}), 160.5 (2-C_{min}), 157.3 (7a-C_{min}), 157.0 (7a-C_{maj}), 135.9 (3a-C_{maj}), 135.6 (3a-C_{min}), 129.9 (1C, d, *J* 25.3, 6-C), 126.0 (5-C_{maj}), 125.8 (5-C_{min}), 124.8 (4-C_{min}), 124.6 (4-C_{maj}), 124.0 (7-C_{maj}), 123.9 (7-C_{min}), 113.6 (CN_{maj}), 113.5 (CN_{min}), 75.5 (morph 2-C_{maj}), 74.6 (morph 2-C_{min}), 66.6 (morph 3-C_{min}), 66.0 (morph 3-C_{min}), 49.3 (morph 5-C_{maj}), 46.4 (morph 5-C_{min}), 46.3 (morph 6-C_{min}), 42.3 (morph 6-C_{maj}), 25.1 (acetyl 2-C_{min}), 25.0 (acetyl 2-C_{maj}). δ_F (565 MHz, $CDCl_3$) 67.9. λ_{max} (neat)/cm^{-1} 3087, 2995, 2877, 1684, 1439, 1412, 1210, 1094. HRMS (ESI) $C_{14}H_{12}FN_3O_4S_2$ requires $[M+Na]^+$, calculated 392.0151, found 392.0147.

2-[*N*-Boc-(2R)-5-cyano-pyrrolidin-2-yl]-1,3-benzothiazole-6-sulfonyl fluoride A-3a

Following General Procedure B, 1,3-benzothiazole-6-sulfonyl fluoride (21.7 mg, 0.10 mmol) was reacted with *N*-Boc-2-cyano-pyrrolidine (98.1 mg, 0.5 mmol), following purification by Mass-Directed HPLC from a 20 minute gradient of 25 – 60% acetonitrile in water, isolating two cross coupled products, initially collecting at 16.5 mins, **A-3a**, as a yellow oil (2.4 mg, 6%, 55:45 mixture of

rotamers). δ_H (500 MHz, $CDCl_3$) 8.60 (1H, br. s, 7-H), 8.17 (1H, d, *J* 8.7, 4-H), 8.10 (1H, br. s, 5-H), 5.42 (1H, br. s, pyrrolidinyl 2-H_{maj}), 5.31 (1H, br. s, pyrrolidinyl 2-H_{min}), 4.81 (1H, br. s, pyrrolidinyl 5-H_{maj}), 4.65 (1H, br. s, pyrrolidinyl 5-H_{min}), 2.56-2.44 (4H, m, pyrrolidinyl 3- and 4-H_2), 1.56 (9H, s, ^{t}Bu 2-H_{3min}), 1.35 (9H, s, ^{t}Bu 2-H_{3maj}). δ_C (125 MHz, $CDCl_3$) 180.2 (^{t}Bu C=O_{min}), 178.1 (^{t}Bu C=O_{maj}), 170.3 (2-C), 157.9 (7a-C) 153.0 (3a-C), 129.5 (1C, d, *J* 25.0, 6-C), 125.8 (7-C), 124.4 (4-C), 123.9 (5-C), 118.5 (CN_{maj}), 118.4 (CN_{min}), 83.7 (^{t}Bu 1-C_{maj}), 83.3 (^{t}Bu 1-C_{min}), 61.1 (pyrrolidinyl 5-C_{min}), 60.5 (pyrrolidinyl 5-C_{maj}), 48.3 (pyrrolidinyl 2-C_{maj}), 48.0 (pyrrolidinyl 2-C_{min}), 33.4 (pyrrolidinyl 4-C_{min}), 32.6 (pyrrolidinyl 4-C_{maj}), 31.9 (pyrrolidinyl 3-C_{maj}), 30.6 (pyrrolidinyl 3-C_{min}), 28.4 (^{t}Bu 2-C_{min}), 28.3 (^{t}Bu 2-C_{maj}). δ_F (565 MHz, $CDCl_3$) 67.9. λ_{max} (neat)/cm^{-1} 3026, 2980, 2959, 2930, 1722, 1405, 1212, 1129. HRMS (ESI) $C_{17}H_{18}FN_3O_4S_2$ requires $[M+Na]^+$, calculated 434.062, found 434.0611.

Also obtained was 2-[*N*-Boc-(2S)-5-cyano-pyrrolidin-2-yl]-1,3-benzothiazole-6-sulfonyl fluoride **A-3b**, as a pale yellow oil (3.1 mg, 7%, 55:45 mixture of rotamers).δ_H (500 MHz, $CDCl_3$) 8.57 (1H, d, *J* 7.6, 7-H), 8.16 (1H, d, *J* 7.6, 4-H), 8.08 (1H, dd, *J* 5.6, 7.6, 5-H), 5.45 (1H, d, *J* 8.5, pyrrolidinyl 2-H_{maj}), 5.37 (1H, d, *J* 8.5, pyrrolidinyl 2-H_{min}), 4.85 (1H, d, *J* 7.5, pyrrolidinyl 2-H_{maj}), 4.75 (1H, d, *J* 7.5, pyrrolidinyl 2-H_{min}), 2.84-2.38 (4H, m, pyrrolidinyl 3- and 4-H_2), 1.55 (9H, s, ^{t}Bu 2-H_{3min}), 1.33 (9H, s, ^{t}Bu 2-H_{3maj}). δ_C (125 MHz, $CDCl_3$) 180.3 (^{t}Bu C=O_{min}), 179.5 (^{t}Bu C=O_{maj}), 157.6 (2-C_{maj}), 157.5 (2-C_{min}), 152.9 (7a-C_{min}), 152.6 (7a-C_{maj}), 135.9 (3a-C_{maj}), 135.4 (3a-C_{min}), 129.4 (1C, d, *J* 24.9, 6-C), 126.1 (5-C_{min}), 125.8 (5-C_{maj}), 124.5 (7-C), 123.8 (4-C_{min}), 123.7 (4-C_{maj}), 118.5 (CN_{maj}), 118.4 (CN_{min}), 83.5 (^{t}Bu 1-C_{maj}), 83.1 (^{t}Bu 1-C_{min}), 59.7 (pyrrolidinyl 5-C_{min}), 59.4 (pyrrolidinyl 5-C_{maj}), 48.5 (pyrrolidinyl 2-C_{maj}), 48.3 (pyrrolidinyl 2-C_{min}), 32.7 (pyrrolidinyl 4-C_{min}), 31.2 (pyrrolidinyl 4-C_{maj}), 30.0 (pyrrolidinyl 3-C_{maj}), 28.8 (pyrrolidinyl 3-C_{min}), 28.4 (^{t}Bu 2-C_{maj}), 28.2 (^{t}Bu 2-C_{min}). δ_F (565 MHz, $CDCl_3$) 67.8. λ_{max} (neat)/cm^{-1} 3082, 3054, 2987, 2955, 1702, 1409, 1210, 1149. HRMS (ESI) $C_{17}H_{18}FN_3O_4S_2$ requires $[M+Na]^+$, calculated 434.0620, found 434.0618.

2-(*N*-Boc-*N*-cyanomethyl-methyl)-1,3-benzothiazole-6-sulfonyl fluoride A-4

Following General Procedure B, 1,3-benzothiazole-6-sulfonyl fluoride (21.7 mg, 0.10 mmol) was reacted with 2-(*N*-Boc-*N*-methylamino)-acetonitrile (85.1 mg, 0.5 mmol), following purification by Mass-Directed HPLC from a 15 minute gradient of 30 – 80% acetonitrile in water, collecting at 6.0 mins to isolate the cross coupled product as a brown amorphous solid (2.4 mg, 6%, 50:50 mixture of rotamers). δ_H (500 MHz, $CDCl_3$) 8.61 (1H, appt br. s, 7-H), 8.22 (1H, appt br. d, *J* 8.7, 4-H), 8.10 (1H, appt br. d, *J* 8.7, 5-H), 4.96 (2H, br. s, *N*-cyanomethyl), 4.43 (2H, br. s, 2-H_{2RotA}), 4.30 (2H, br. s, 2- H_{2RotB}), 1.55 (9H, br. s, ^{t}Bu 2-H_{3RotA}), 1.50 (9H, br. s, ^{t}Bu 2-H_{3RotB}). δ_C (125 MHz, $CDCl_3$) 173.4 (^{t}Bu C=O_{RotA}), 173.3 (^{t}Bu C=O_{RotB}), 157.2 (2-C), 153.5 (7a-C), 136.0 (3a-C), 129.9 (1C, d, *J* 24.1, 6-C), 126.1 (5-C), 124.6 (7-C), 123.9 (4-C), 115.4 (CN_{RotA}). 115.2 (CN_{RotB}), 83.9 (^{t}Bu 1-C_{RotA}), 83.7 (^{t}Bu 1-C_{RotB}), 49.6 (*N*-cyano 1-C_{RotA}), 49.4 (*N*-cyano 1-C_{RotB}), 36.6 (Me_{RotA}), 35.9 (Me_{RotB}), 28.6 (^{t}Bu 2-C_{RotA}), 28.2 (^{t}Bu 2-C_{RotB}). δ_F (565 MHz, $CDCl_3$) 66.9. λ_{max} (neat)/cm^{-1} 3068, 2977, 2932, 1706,

1592, 1508, 1410, 1210, 1051. HRMS (ESI) $C_{15}H_{16}FN_3O_4S_2$ requires $[M+Na]^+$, calculated 408.0464, found 408.0456.

2-(Oxolan-2-yl)-1,3-benzothiazole-6-sulfonyl fluoride A-6

Following General Procedure B, 1,3-benzothiazole-6-sulfonyl fluoride (21.7 mg, 0.10 mmol) was reacted with tetrahydrofuran (216.3 mg, 3 mmol), following purification by Mass-Directed HPLC from a 15 minute gradient of 5 – 95% acetonitrile in water, collecting at 11.5 mins to isolate the cross coupled product, **1-6**, as a yellow amorphous solid (2.9 mg, 10%). δ_H (500 MHz, $CDCl_3$) 8.60 (1H, d, *J* 1.9, 7-H), 8.15 (1H, d, *J* 8.7, 4-H), 8.07 (1H, dd, *J* 8.7, 1.9, 5-H), 5.38 (1H, dd, *J* 8.0, 5.4, oxo 2-H), 4.19 (1H, ddd, *J* 8.4, 7.3, 5.7, oxo 5-Ha), 4.05 (1H, ddd, *J* 8.4, 7.3, 5.7, oxo 5-Hb), 2.56 (1H, appt ddd, *J* 8.4, 7.3, 5.8, oxo 3-Ha), 2.28 (1H, appt ddd, *J* 8.4, 7.3, 5.8, oxo 3-Hb), 2.04 (2H, m, oxo 4-H_2). δ_C (125 MHz, $CDCl_3$) 182.7 (2-C), 157.1 (7a-C), 134.6 (3a-C), 127.8 (1C, d, *J* 24.8, 6-C), 124.4 (5-C), 122.9 (4-C), 122.7 (7-C). 77.2 (oxo 2-C), 68.5 (oxo 5-C), 32.4 (oxo 3-C), 24.7 (oxo 4-C). δ_F (565 MHz, $CDCl_3$) 67.0. λ_{max} (neat)/cm^{-1} 3065, 2955, 2875, 1697, 1591, 1509, 1409, 1210, 1066. HRMS (ESI) $C_{11}H_{10}FNO_3S_2$ requires $[M+H]^+$, calculated 266.0164, found 288.0153.

2-((2-Oxoazepan-1-yl)-methyl)-1,3-benzothiazole-6-sulfonyl fluoride A-8

Following General Procedure B, 1,3-benzothiazole-6-sulfonyl fluoride (21.7 mg, 0.10 mmol) was reacted with *N*-methylcaprolactam (381.6 mg, 3 mmol), following purification by Mass-Directed HPLC from a 10 minute gradient of 5 – 95% acetonitrile in water, collecting at 3.5 mins to isolate the cross coupled product as a brown oil (5.6 mg, 16%). δ_H (500 MHz, $CDCl_3$) 8.57 (1H, d, *J* 1.9, 7-H), 8.17 (1H, d, *J* 8.7, 4-H), 8.06 1H, dd, *J* 8.7, 1.9), 5.02 (2H, s, Me H_2), 3.54 (2H, t, *J* 5.1, 7-H_2), 2.64 (2H, t, *J* 5.1, 3-H_2), 1.76-1.73 (4H, m, 4- and 6-H_2), 1.65-1.60 (2H, m, 5-H_2). δ_C (125 MHz, $CDCl_3$) 176.5 (C=O), 175.6 (2-C), 157.1 (7a-C), 136.7 (3a-C), 129.4 (1C, d, *J* 25.0, 6-C), 125.7 (5-C), 124.2 (4-C), 123.7 (7-C), 50.9 (aze 7-C), 50.7 (aze 3-C), 36.9 (Me), 30.0 (aze 6-C), 28.5 (aze 5-C), 23.3 (aze 4-C). δ_F (565 MHz, $CDCl_3$) 69.0. λ_{max} (neat)/cm^{-1} 3063, 2933, 2857, 1643, 1508, 1481, 1408, 1209, 1161. HRMS (ESI) $C_{14}H_{15}FN_2O_3S_2$ requires $[M+H]^+$, calculated 343.0586, found 343.0586.

2-(1,4-Dioxan-2-yl)-1,3-benzothiazole-6-sulfonyl fluoride A-10

Following General Procedure B, 1,3-benzothiazole-6-sulfonyl fluoride (21.7 mg, 0.10 mmol) was reacted with 1 4-dioxane (264.3 mg, 3 mmol), following purification by Mass-Directed HPLC from a 15 minute gradient of 5 – 50% acetonitrile in water, collecting at 7.0 mins to isolate the cross coupled product as a cream amorphous solid (4.5 mg, 15%). δ_H (500 MHz, $CDCl_3$) 8.64 (1H, d, *J* 2.0, 7-H), 8.18 (1H, d, *J* 8.7, 4-H), 8.09 1H, dd, *J* 8.7, 2.0), 5.10 (1H, dd, *J* 9.6, 3.2, dio 2-H), 4.05-3.98 (2H, m, dio 3-H_2), 3.88-3.68 (4H, m, dio 5- and 6-H_2). δ_C (125 MHz, $CDCl_3$) 176.3 (2-C), 157.4 (7a-C), 135.6 (3a-C), 129.4 (1C, d, *J* 25.1, 6-C), 125.7 (5-C), 124.4 (4-C), 123.9 (7-C), 75.4 (dio 2-C), 70.3

(dio 3-C), 67.2 (dio 6-C), 66.5 (dio 5-C). δ_F (565 MHz, $CDCl_3$) 69.1. λ_{max} (neat)/cm^{-1} 3067, 2968, 2920, 2859, 1696, 1515, 1444, 1409, 1211, 1064. HRMS (ESI) $C_{11}H_{10}FNO_4S_2$ requires $[M+H]^+$, calculated 304.0114, found 304.0103.

2-(1-Methyl-5-oxopyrrolidin-2-yl)-1,3-benzothiazole-6-sulfonyl fluoride A-12

Following General Procedure B, 1,3-benzothiazole-6-sulfonyl fluoride (21.7 mg, 0.10 mmol) was reacted with 1-methyl-pyrrolidin-2-one (49.6 mg, 0.5 mmol), following purification by Mass-Directed HPLC from a 10 minute gradient of 5 – 95% acetonitrile in water, collecting at 6.5 mins to isolate the cross coupled product as a yellow oil (7.78 mg, 25%). δ_H (500 MHz, $CDCl_3$) 8.63 (1H, d, *J* 1.9, 7-H), 8.22 (1H, d, *J* 8.7, 4-H), 8.11 (1H, dd, *J* 8.7, 1.9), 5.09 (1H, dd, *J* 7.6 4.0, pyrrolidinyl 2-H), 2.91 (3H, s, Me), 2.74-2.70 (2H, m, pyrrolidinyl 4-H_2), 2.55-2.49 (1H, m, pyrrolidinyl 3-H_a), 2.24-2.19 (1H, m, pyrrolidinyl 3-H_b). δ_C (125 MHz, $CDCl_3$) 179.3 (C=O), 175.3 (2-C), 157.5 (7a-C), 136.7 (3a-C), 129.9 (1C, d, *J* 25.3, 6-C), 126.1 (5-C), 124.7 (4-C), 123.9 (7-C), 62.9 (pyrrolidinyl 2-C), 29.2 (pyrrolidinyl 4-C), 26.8 (pyrrolidinyl 3-C). δ_F (565 MHz, $CDCl_3$) 69.0. λ_{max} (neat)/cm^{-1} 3068, 2929, 2162, 1693, 1591, 1508, 1409, 1209, 1050. HRMS (ESI) $C_{12}H_{11}FN_2O_3S_2$ requires $[M+Na]^+$, calculated 337.0093, found 337.0089.

2-[1-(*N*-Ethylacetamido)ethyl]-1,3-benzothiazole-6-sulfonyl fluoride A-13

Following General Procedure B, 1,3-benzothiazole-6-sulfonyl fluoride (21.7 mg, 0.10 mmol) was reacted with *N*,*N*-diethylacetamide (57.6 mg, 0.5 mmol), following purification by Mass-Directed HPLC from a 15 minute gradient of 20 – 80% acetonitrile in water, collecting at 9.5 mins to isolate the cross coupled product as a yellow oil (5.1 mg, 15%). δ_H (500 MHz, $CDCl_3$) 8.55 (1H, d, *J* 2.0, 7-H), 8.18 (1H, d, *J* 8.7, 4-H), 8.05 (1H, dd, *J* 8.7, 2.0), 6.02 (1H, q, *J* 7.1, Et 1-H), 3.52-3.44 (1H, m, *N*-Et 1-Ha), 3.38-3.30 (1-H, m, *N*-Et 1-Hb), 2.22 (3H, s, Ac 2-H_3), 1.83 (3H, d, *J* 7.1, Et 2-H_3), 1.21 (3H, t, *J* 7.1 *N*-Et 2-H_3). δ_C (125 MHz, $CDCl_3$) 179.5 (Ac C=O), 171.1 (2-C), 157.2 (7a-C), 136.5 (3a-C), 129.2 (1C, d, *J* 24.9, 6-C), 125.5 (5-C), 124.3 (4-C), 123.6 (7-C), 52.4 (Et 1-C), 40.9 (*N*-Et 1-C), 21.9 (Ac 2-C), 17.2 (Et 2-C), 16.1 (*N*-Et 2-C). δ_F (565 MHz, $CDCl_3$) 66.7. λ_{max} (neat)/cm^{-1} 3052, 3020, 2990, 2891, 2779, 1679, 1415, 1193. HRMS (ESI) $C_{13}H_{15}FN_2O_3S_2$ requires $[M+Na]^+$, calculated 337.0089, found 353.0400.

2-(*N*-Acetylpiperidin-2-yl)-1,3-benzothiazole-6-sulfonyl fluoride A-14

Following General Procedure B, 1,3-benzothiazole-6-sulfonyl fluoride (21.7 mg, 0.10 mmol) was reacted with *N*-Acetylpiperidine (63.6 mg, 0.5 mmol), following purification by Mass-Directed HPLC from a 10 minute gradient of 5 – 95% acetonitrile in water, collecting at 8.0 mins to isolate the cross coupled product as a yellow oil (8.4 mg, 24%). δ_H (500 MHz, $CDCl_3$) 8.55 (1H, d, *J* 1.9, 7-H), 8.17 (1H, d, *J* 8.7, 4-H), 8.07 (1H, dd, *J* 8.7, 1.9, 5-H), 6.30 (1H, appt d, *J* 5.7, pip 2-H), 3.84-3.82 (1H,

m, pip 6-Ha), 3.29-3.24 (1H, m, pip 6-Hb), 2.70-2.67 (1H, m, 3-Ha), 2.26 (3H, s, Ac 2-H_3), 1.97-1.90 (1H, m, pip 3-Hb), 1.83-1.81 (1H, m, pip 5-Ha), 1.74-1.56 (3H, m, pip 4-H_2 and 5-Hb). δ_C (125 MHz, $CDCl_3$) 179.2 (Ac C=O), 170.4 (2-C), 157.7 (7a-C), 136.6 (3a-C), 129.2 (1C, d, *J* 25.0, 6-C), 125.6 (5-C), 124.3 (4-C), 123.5 (7-C), 51.7 (pip 2-C), 44.1 (pip 6-C), 27.9 (pip 3-C), 25.6 (pip 5-C), 21.8 (pip 4-C), 20.1 (Ac 2-C). δ_F (565 MHz, $CDCl_3$) 69.0. λ_{max} (neat)/cm^{-1} 2935, 2861, 1720, 1648, 1591, 1503, 1410, 1209, 1048. HRMS (ESI) $C_{14}H_{15}FN_2O_3S_2$ requires $[M+Na]^+$, calculated 365.0406, found 365.0402.

2-[*N*-[(2-Methoxyethyl)(methyl)carbamoyl]pyrrolidin-2-yl]-1,3-benzothiazole-6-sulfonyl fluoride A-20

Following General Procedure B, 1,3-benzothiazole-6-sulfonyl fluoride (21.7 mg, 0.10 mmol) was reacted *N*-(2-methoxyethyl)-*N*-methylpyrrolidine-1-carboxamide (93.1 mg, 0.5 mmol), following purification by Mass-Directed HPLC from a 10 minute gradient of 20 – 95% acetonitrile in water, collecting at 6.5 mins to isolate the cross coupled product as a brown oil (7.1 mg, 18%). δ_H (500 MHz, $CDCl_3$) 8.52 (1H, d, *J* 1.9, 7-H), 8.12 (1H, d, *J* 8.7, 4-H), 8.03 (1H, dd, *J* 8.7, 1.9, 5-H), 5.64 (1H, t, *J* 7.4, pyrrolidinyl 2-H), 3.71-3.69 (2H, m, OCHa and NCHa), 3.59-3.55 (2H, m, OCHb and NCHb), 3.46-3.44 (1H, m, pyrrolidinyl 5-Ha), 3.35 (OMe), 3.33-3.29 (1H, m, pyrrolidinyl 5-Hb), 3.07 (NMe), 2.61-2.59 (1H, m, pyrrolidinyl 3-Ha), 2.10-2.02 (2H, m, pyrrolidinyl 3-Hb and 4-H_a), 1.90-1.88 (1H, m, 4-Hb). δ_C (125 MHz, $CDCl_3$) 184.2 (C=O), 163.0 (2-C), 158.4 (7a-C), 135.6 (3a-C), 128.7 (1C, d, *J* 24.7, 6-C), 125.5 (5-C), 124.0 (4-C), 123.6 (7-C), 71.1 (OCH_2), 61.3 (pyrrolidinyl 2-C), 59.0 (OMe), 51.5 (NCH_2), 49.6 (pyrrolidinyl 5-C), 37.4 (NMe), 33.3 (pyrrolidinyl 3-C), 26.3 (pyrrolidinyl 4-C). δ_F (565 MHz, $CDCl_3$) 69.0. λ_{max} (neat)/cm^{-1} 2931, 2889, 1727, 1635 1496, 1445, 1408, 1209, 1068. HRMS (ESI) $C_{17}H_{20}FN_3O_4S_2$ requires $[M+Na]^+$, calculated 412.1706, found 412.1699.

6-(*N*-Boc-pyrrolidin-2-yl)pyridine-3-sulfonyl fluoride C-1

Following General Procedure B, pyridine-3-sulfonyl fluoride (16.1 mg, 0.10 mmol) was reacted *N*-Boc pyrrolidine (85.6 mg, 0.5 mmol), following purification by Mass-Directed HPLC from a 15 minute gradient of 40 – 80% acetonitrile in water, collecting at 8.5 mins to isolate the cross coupled product as a colourless oil (0.8 mg, 2%, 55:45 mixture of rotamers). δ_H (500 MHz, $CDCl_3$) 9.11 (1H, s, 2-H), 8.21 (1H, appt t, *J* 9.8, 4-H), 7.47 (1H, m, 5-H), 5.06-5.04 (1H, m, pyrrolidinyl 2-H_{min}), 4.99-4.95 (1H, m, pyrrolidinyl 2-H_{maj}), 3.66-3.57 (2H, m, 5-H_2), 2.44-2.38 (1H, m, 3-H_a), 2.02-1.94 (3H, m, 4-H_2 and 3-H_b), 1.45 (9H, s, ^{t}Bu 3-H_{3min}), 1.21 (9H, s, ^{t}Bu 2-H_{3maj}). δ_C (125 MHz, $CDCl_3$) 172.1 (^{t}Bu C=O_{maj}), 171.0 (^{t}Bu C=O_{min}), 154.9 (6-C_{min}), 154.2 (6-C_{maj}), 149.0 (2-C_{min}), 148.8 (2-C_{maj}), 136.7 (4-C_{min}), 136.4 (4-C_{maj}), 128.1 (1C, d, *J* 25.3, 3-C) , 121.0 (5-C_{min}), 120.4 (5-C_{maj}), 80.3 (^{t}Bu 1-C), 63.0 (pyrrolidinyl 2-C_{maj}), 62.5 (pyrrolidinyl 2-C_{min}), 47.7 (5-C_{min}), 47.3 (5-C_{maj}), 34.4 (3-C_{maj}) 33.2 (3-C_{min}), 28.6 (^{t}Bu 2-C_{min}) 28.3 (^{t}Bu 2-C_{maj}), 24.2 (4-C_{min}), 23.5 (4-C_{maj}). δ_F (565 MHz, $CDCl_3$) 69.5 (SO_2F_{maj}),

69.3 (SO_2F_{min}). λ_{max} (neat)/cm^{-1} 2977, 2928, 1697, 1477, 1391, 1214, 1117. HRMS (ESI) $C_{14}H_{19}FN_2O_4S$ requires $[M+Na]^+$, calculated 353.0947, found 353.0931.

6-(*N*-Boc-5-cyanopyrrolidin-2-yl)pyridine-3-sulfonyl fluoride C-3

Following General Procedure B, pyridine-3-sulfonyl fluoride (16.1 mg, 0.10 mmol) was reacted *N*-Boc-2-cyano-pyrrolidine (98.1 mg, 0.5 mmol), following purification by Mass-Directed HPLC from a 20 minute gradient of 30 – 40% acetonitrile in water, collecting at 11.0 mins to isolate the cross coupled product as a yellow oil (3.9 mg, 11%, 50:50 mixture of diastereomers, 60:40 mixture of rotamers,). δ_H (500 MHz, $CDCl_3$) 9.15 (1H, s, 2-H_{min}), 9.09 (1H, s, 2-H_{maj}), 8.29 (1H, dd, *J* 8.3, 2.4, 4-H_{maj}), 8.24 (1H, dd, *J* 8.3, 2.4, 4-H_{min}), 7.60 (1H, d, *J* 8.3, 5-H_{maj}), 7.52 (1H, d, *J* 8.3, 5-H_{min}), 5.17 (1H, appt d, *J* 8.0, pyrrolidinyl 2S-H_{maj}), 5.13-5.11 (1H, m, pyrrolidinyl 2R-H_{maj}), 5.07 (1H, appt d, *J* 8.0, pyrrolidinyl 2S-H_{min}), 5.02-5.00 (1H, m, pyrrolidinyl 2R-H_{min}), 4.89 (1H, appt d, *J* 8.0, pyrrolidinyl 5S-H_{min}), 4.89-4.87 (1H, m, pyrrolidinyl 2R-H_{min}), 4.77 (1H, appt d, *J* 8.0, pyrrolidinyl 5S-H_{maj}), 4.72-4.70 (1H, m, pyrrolidinyl 2R-H_{maj}), 2.62-2.22 (4H, m, pyrrolidinyl 3- and 4-H_2), 1.50 (9H, s, ^{t}Bu 2-H_{3maj}), 1.22 ((9H, s, ^{t}Bu 2-H_{3min}). δ_C (125 MHz, $CDCl_3$) 168.6 (^{t}Bu C=O_{min}), 168.5 (^{t}Bu C=O_{maj}), 153.0 (6-C_{min}), 152.9 (6-C_{maj}), 149.0 (2-C_{min}), 148.8 (2-C_{maj}), 137.0 (4-C_{maj}), 136.7 (4-C_{min}), 129.0 (1C, d, *J* 25.7, 3-C_{min}), 128.8 (1C, d, *J* 25.7, 3-C_{maj}), 122.1 (5-C_{maj}), 121.1 (5-C_{min}), 120.4 (CN_{min}), 119.0 (CN_{maj}), 83.0 (^{t}Bu 1-C_{maj}), 82.8 (^{t}Bu 1-C_{min}), 63.2 (pyrrolidinyl 5-C_{min}), 61.7 (pyrrolidinyl 5-C_{maj}), 48.7 (pyrrolidinyl 2-C_{maj}), 48.6 (pyrrolidinyl 2-C_{min}), 32.5 (pyrrolidinyl 4-C_{min}), 31.3 (pyrrolidinyl 4-C_{maj}), 30.4 (pyrrolidinyl 3-C_{min}), 29.7 (pyrrolidinyl 3-C_{maj}), 28.4 (^{t}Bu 2-C_{maj}), 28.2 (^{t}Bu 2-C_{min}). δ_F (565 MHz, $CDCl_3$) 67.7 and 67.6 (S_{min} and R_{min}), 67.5 and 67.4 (S_{maj} and R_{maj}). λ_{max} (neat)/cm^{-1} 2975, 2930, 1702, 1582, 1475, 1367, 1214, 1120. HRMS (ESI) $C_{15}H_{18}FN_3O_4S$ requires $[2M+Na]^+$, calculated 733.1902, found 733.1906.

6-(*N*-Boc-*N*-cyanomethyl-methyl) pyridine-3-sulfonyl fluoride C-4

Following General Procedure B, pyridine-3-sulfonyl fluoride (16.1 mg, 0.10 mmol) was reacted 2-(*N*-Boc-*N*-methylamino)-acetonitrile (85.1 mg, 0.5 mmol), following purification by Mass-Directed HPLC from a 15 minute gradient of 30 – 60% acetonitrile in water, collecting at 11.0 mins to isolate the cross coupled product as a yellow oil (3.0 mg, 9%, 90:10 mixture of rotamers). δ_H (500 MHz, $CDCl_3$) 9.14 (1H, s, 2-H_{maj}), 8.89 (1H, s, 2-H_{min}), 8.38 (1H, appt s, 4-H_{min}), 8.29 (1H, appt s, 4-H_{maj}), 7.59-7.51 (1H, m, 5-H), 4.73 (2H, s, *N*-cyanomethyl), 4.43 (2H, br. s, 2-H_{2min}), 4.31 (2H, br. s, 2-H_{2min}, 1.52 (9H, br. s, ^{t}Bu 2-H_{3maj}), 1.40 (9H, br. s, ^{t}Bu 2-H_{3min}). δ_C (125 MHz, $CDCl_3$) 164.1 (^{t}Bu C=O_{min}), 164.3 (^{t}Bu C=O_{maj}), 154.8 (6-C_{min}), 154.4 (6-C_{maj}), 149.0 (2-C_{min}), 148.9 (2-C_{maj}), 137.2 (4-C), 129.3 (1C, d, *J* 26.3, 3-C), 121.0 (5-C_{min}), 120.4 (5-C_{maj}), 115.7 (CN), 83.2 (^{t}Bu 1-C_{maj}), 82.9 (^{t}Bu 1-C_{min}), 52.6 (*N*-cyano 1-C_{min}), 52.4 (*N*-cyano 1-C_{maj}), 37.1 (Me_{maj}), 36.3 (Me_{min}), 28.3 (^{t}Bu 2-C_{maj}), 28.1 (^{t}Bu 2-C_{min}). δ_F (565 MHz, $CDCl_3$) 67.5 ($SO_2F_{maj,}$) 63.5 (SO_2F_{min}). λ_{max} (neat)/cm^{-1} 3068, 2979,

2934, 1703, 1564, 1452, 1313, 1213, 1160. HRMS (ESI) $C_{13}H_{16}FN_3O_4S$ requires $[M+H]^+$, calculated 330.0924, found 330.0910.

2,6-bis(oxolan-2-yl)pyridine-3-sulfonyl fluoride C-6

Following General Procedure B, pyridine-3-sulfonyl fluoride (16.1 mg, 0.10 mmol) was reacted tetrahydrofuran (216.3 mg, 3 mmol), following purification by Mass-Directed HPLC from a 15 minute gradient of 30–50% acetonitrile in water, collecting at 12.0 mins to isolate the cross coupled product as a pale yellow oil (1.2 mg, 4%). δ_H (500 MHz, $CDCl_3$) 8.28 (1H, appt dd, *J* 8.4, 2.1, 4-H), 7.61 (1H, appt t, *J* 8.4, 5-H), 5.67 and 5.13 (2H, dd, *J* 7.7, 5.8, oxo 2-H), 4.23-4.18 and 4.08-4.02 (4H, m, oxo 5-H_2), 2.51-2.48 and 2.40-2.36 (2H, m, oxo 3-H_a), 2.24-2.17 (2H, m, oxo 3-H_b), 2.07-2.01 (4H, m, oxo 4-H_2). δ_C (125 MHz, $CDCl_3$) 170.4 (6-C), 161.9 (2-C), 138.9 (4-C), 126.8 (1C, d, *J* 25.3, 3-C), 118.7 (5-C), 81.1 and 80.8 (oxo 2-C), 70.13 and 69.5 (oxo 5-C), 33.1 and 32.2 (oxo 3-C), 26.4 and 25.8 (4-C). δ_F (565 MHz, $CDCl_3$) 66.2. λ_{max} (neat)/cm^{-1} 3107, 3061, 2981, 1406, 1211, 1132. HRMS (ESI) $C_{13}H_{16}FNO_4S$ requires $[M+Na]^+$, calculated 324.0682, found 374.0847.

6-(*N*-methyl-7-oxoazepan-2-yl)pyridine-3-sulfonyl fluoride 3-8a

Following General Procedure B, pyridine-3-sulfonyl fluoride (16.1 mg, 0.10 mmol) was reacted *N*-methylcaprolactam (381.6 mg, 3 mmol), following purification by Mass-Directed HPLC from a 20 minute gradient of 20 – 30% acetonitrile in water, initially collected at 10.0 mins as a yellow oil (2.3 mg, 8%,). δ_H (500 MHz, $CDCl_3$) 9.12 (1H, s, 2-H), 8.22 (1H, td, *J* 10.3, 3.1, 4-H), 7.43 (1H, appt t, *J* 10.3, 5-H), 3.76-3.70 (1H, m, aze 2-H), 3.44-3.30 (2H, m, aze 6-H_2), 3.05 (3H, s, Me), 2.83-2.77 (2H, m, aze 3-H_2), 1.75-1.65 (4H, m aze 4- and 5-H_2). δ_C (125 MHz, $CDCl_3$) 175.6 (C=O), 171.9 (6-C), 148.9 (2-C), 136.9 (4-C), 128.0 (1C, d, *J* 25.3, 3-C), 122.5 (5-C), 51.2 (aze 7-C), 42.6 (aze 3-C), 36.4 (aze 3-C), 36.3 (NMe), 35.3 (aze 5-C), 28.8 (aze 4-C). δ_F (565 MHz, $CDCl_3$) 67.7. λ_{max} (neat)/cm^{-1} 2939, 1708, 1637, 1413, 1212, 1120. HRMS (ESI) $C_{12}H_{15}FN_2O_3S$ requires $[M+H]^+$, calculated 287.0866, found 287.0854.

Also obtained was 6-[(2-oxoazepan-1-yl)methyl]pyridine-3-sulfonyl fluoride **C-8b**, isolated at 11.0 min as a colourless oil (4.7 mg, 17%). δ_H (500 MHz, $CDCl_3$) 9.10 (1H, s, 2-H), 8.21 (1H, dd, *J* 8.4, 2.4, 4-H), 7.59 (1H, d, *J* 8.4, 5-H), 4.80 (2H, s, NCH_2), 3.49-3.47 (2H, m, aze 7-H_2), 2.63-2.60 (2H, m, aze 3-H_2), 1.77-1.73 (4H, m, aze 6- and 4-H_2), 1.64-1.61 (2H, m, aze 5-H_2). δ_C (125 MHz, $CDCl_3$) 176.6 (C=O), 166.0 (6-C), 148.7 (2-C), 136.8 (4-C), 128.7 (1C, d, *J* 25.5, 3-C), 123.1 (5-C), 54.1 (NMe), 50.8 (aze 7-C), 37.0 (aze 3-C), 30.0 (aze 6-C), 28.3 (aze 5-C), 23.4 (aze 4-C). δ_F (565 MHz, $CDCl_3$) 67.6. λ_{max} (neat)/cm^{-1} 3061, 2930, 2859, 1643, 1562, 1444, 1413, 1211, 1110. HRMS (ESI) $C_{12}H_{15}FN_2O_3S$ requires $[M+H]^+$, calculated 287.0866, found 287.0856.

2,6-bis(1,4-dioxan-2-yl)pyridine-3-sulfonyl fluoride C-10a

Following General Procedure B, pyridine-3-sulfonyl fluoride (16.1 mg, 0.10 mmol) was reacted 1 4-dioxane (264.3 mg, 3 mmol), following purification by Mass-Directed HPLC from a 10 minute gradient of 25 – 60% acetonitrile in water, isolating two cross coupled products as disubstituted regioisomers, initially collecting at 6.0 mins, **C-10a**, as a pale yellow oil (3.7 mg, 11%, 50:50 mixture of diastereomers). δ_H (500 MHz, $CDCl_3$) 8.38 (1H, d, *J* 8.4, 4-H), 7.74 (1H, appt t, *J* 8.4, 5-H), 5.28 (1H, dd, *J* 9.1 ,3.3, 2- or 6-dio 2-H_{DiaA}), 5.25 (1H, dd, *J* 9.1 ,3.3, 2- or 6-dio 2-H_{DiaB}), 4.88 (1H, dd, *J* 9.9 ,3.1, 2- or 6-dio 2-H_{DiaA}), 4.80 (1H, dd, *J* 9.9 ,3.1, 2- or 6-dio 2-H_{DiaB}), 4.26 and 4.12 (1H, appt td, *J* 11.0, 3.1, 2- and 6-dio 3-Ha), 4.04-3.92 (2H, m, dio 3-H_2), 3.83-3.39 (8H, m, 5- and 6-H_2). δ_C (125 MHz, $CDCl_3$) 164.5 (6-C_{DiaA}), 164.2 (6-C_{DiaB}), 155.8 (2-C_{DiaA}), 155.7 (2-C_{DiaB}), 139.8 (4-C_{DiaA}) 139.7 (4-C_{DiaB}), 129.0 (1C, d, *J* 25.5, 3-C_{DiaA}), 128.1 (1C, d, *J* 25.5, 3-C_{DiaB}), 120.4 (5-C_{diaA}) 120.3 (5-C_{DiaB}), 74.5 and 74.4 (dio 2-C), 70.9 and 70.7 (dio 3-C), 69.1 and 69.0 (dio 6-C), 66.5 and 66.4 (dio 5-C). δ_F (565 MHz, $CDCl_3$) 65.3 (SO_2F_{DiaA}), 65.2 (SO_2F_{DiaB}). λ_{max} (neat)/cm^{-1} 2965, 2918, 2858, 1730, 1656, 1577, 1449, 1415, 1209, 965. HRMS (ESI) $C_{13}H_{16}FNO_6S$ requires $[M+Na]^+$, calculated 356.0580, found 356.0572.

Also obtained was 4,6-bis(1,4-dioxan-2-yl)pyridine-3-sulfonyl fluoride **C-10b**, isolated at 6.5 mins as a pale yellow oil (3.2 mg, 10%, 50:50 mixture of diastereomers). δ_H (500 MHz, $CDCl_3$) 9.09 (1H, s, 2-H), 8.04 (1H, appt d, *J* 5.0, 5-H), 5.30 (1H, appt t, *J* 2.7, 4- or 6-dio 2-H_{DiaA}), 5.25 (1H, appt t, *J* 2.7, 4- or 6-dio 2-H_{DiaB}), 4.84 (1H, appt t, *J* 2.7, 4- or 6-dio 2-H_{DiaA}), 4.26 (1H, appt t, *J* 2.7, 4- or 6-dio 2-H_{DiaB}), 4.11-3.96 (4H, m, dio 3-H_2), 3.87-3.70 (8H, m, 5- and 6-H_2). δ_C (125 MHz, $CDCl_3$) 166.5 (6-C_{DiaA}), 166.3 (6-C_{DiaB}), 149.6 (2-C_{DiaA}), 148.5 (2-C_{DiaB}), 149.0 (4-C), 126.4 (1C, d, *J* 23.5, 3-C_{DiaA}), 126.3 (1C, d, *J* 23.5, 3-C_{DiaB}), 120.3 (5-C_{DiaA}) 120.1 (5-C_{DiaB}), 73.9 and 73.8 (dio 2-C), 70.7 and 70.6 (dio 3-C), 67.1 and 66.9 (dio 6-C), 66.4 and 66.3 (dio 5-C). δ_F (565 MHz, $CDCl_3$) 69.2 (SO_2F_{DiaA}), 69.1 (SO_2F_{DiaB}). λ_{max} (neat)/cm^{-1} 2963, 2920, 2857, 1730, 1590, 1545, 1449, 1416, 1211, 1056. HRMS (ESI) $C_{13}H_{16}FNO_6S$ requires $[M+Na]^+$, calculated 356.0580, found 356.0583.

1-[*N*-Boc-(5S)-5-cyanopyrrolidin-2-yl]isoquinoline-5-sulfonyl fluoride D-3a

Following General Procedure B, isoquinoline-8-sulfonyl fluoride (21.1 mg, 0.10 mmol) was reacted *N*-Boc-2-cyano-pyrrolidine (98.1 mg, 0.5 mmol), following purification by Mass-Directed HPLC from a 20 minute gradient of 40 – 60% acetonitrile in water, isolating two cross coupled products, initially collecting at 11.0 mins as a yellow amorphous solid (5.3 mg, 13%, 60:30 mixture of rotamers). δ_H (500 MHz, $CDCl_3$) 8.66 (1-H, m, 8-H), 8.63 (1H, m, 3-H), 8.58 (1H, m, 6-H), 8.25 (1H, m, 4-H), 7.79 (1H, m, 7-H), 5.95 (1H, br. s, pyrrolidinyl 2-H_{maj}), 5.75 (1H, br. s, pyrrolidinyl 2-H_{min}), 4.87 (1H, br. s, pyrrolidinyl 5-H_{min}), 4.75 (1H, br. s, pyrrolidinyl 5-H_{maj}), 2.63-2.49 (4H, m, pyrrolidinyl 3- and 4-H_2), 1.49 (9H, s, ^{t}Bu 2-H_{3min}), 1.00 (9H, s, ^{t}Bu 2-H_{3maj}). δ_C (125 MHz, $CDCl_3$) 161.7 (^{t}Bu C=O_{min}), 160.1

(^{t}Bu C=O$_{maj}$), 153.3 (1-C$_{min}$), 153.1 (1-C$_{maj}$), 145.6 (3-C$_{min}$), 145.4 (3-C$_{maj}$), 134.9 (8-C$_{min}$), 134.8 (8-C$_{maj}$), 134.4 (4a-C), 132.6 (7-C$_{maj}$), 132.4 (7-C$_{min}$), 132.0 (8a-C), 129.5 (1C, d, *J* 24.2, 5-C), 126.2 (6-C$_{min}$), 126.1 (6-C$_{maj}$), 119.6 (4-C$_{min}$), 119.1 (4-C$_{maj}$), 116.2 (CN$_{maj}$), 116.1 (CN$_{min}$), 82.3 (^{t}Bu 1-C$_{maj}$), 81.1 (^{t}Bu 1-C$_{min}$), 59.1 (pyrrolidinyl 5-C$_{min}$), 58.5 (pyrrolidinyl 5-C$_{maj}$), 48.3 (pyrrolidinyl 2-C$_{maj}$), 48.2 (pyrrolidinyl 2-C$_{min}$), 32.3 (pyrrolidinyl 4-C$_{min}$), 31.9 (pyrrolidinyl 4-C$_{maj}$), 30.3 (pyrrolidinyl 3-C$_{maj}$), 29.5 (pyrrolidinyl 3-C$_{min}$), 28.4 (^{t}Bu 2-C$_{min}$), 28.3 (^{t}Bu 2-C$_{maj}$). δ_F (565 MHz, $CDCl_3$) 64.6 (SO_2F_{min}), 64.5 (SO_2F_{maj}). λ_{max} (neat)/cm^{-1} 3101, 2978, 2940, 1701, 1614, 1580, 1561, 1387, 1197, 1131. HRMS (ESI) $C_{19}H_{19}FN_3O_4S$ requires [M+Na]$^+$, calculated 428.1056, found 428.1061.

Also obtained was 1-[*N*-Boc-(5R)-5-cyanopyrrolidin-2-yl]isoquinoline-5-sulfonyl fluoride **D-3b**, isolated at 11.5 mins as a yellow amorphous solid (4.8 mg, 12%, 60:40 mixture of rotamers). δ_H (500 MHz, $CDCl_3$) 8.65 (1H, m, 3-H), 8.57 (1-H, m, 8-H), 8.57 (1H, m, 6-H), 8.24 (1H, m, 4-H), 7.81 (1H, m, 7-H), 5.99 (1H, d, *J* 8.3, pyrrolidinyl 2-H$_{maj}$), 5.87 (1H, d, *J* 8.3, pyrrolidinyl 2-H$_{min}$), 5.06 (1H, d, *J* 8.3, pyrrolidinyl 5-H$_{maj}$), 4.97 (1H, d, *J* 8.3, pyrrolidinyl 5-H$_{min}$), 2.75 (1H, m, pyrrolidinyl 3-Ha), 2.62 (1H, m, pyrrolidinyl 3-Hb), 2.32 (1H, m, pyrrolidinyl 4-Ha), 2.06 (1H, m, pyrrolidinyl 4-Ha), 1.49 (9H, s, ^{t}Bu 2-H$_{3min}$), 0.99 (9H, s, ^{t}Bu 2-H$_{3maj}$). δ_C (125 MHz, $CDCl_3$) 161.7 (^{t}Bu C=O$_{min}$), 160.9 (^{t}Bu C=O$_{maj}$), 153.3 (1-C$_{min}$), 153.2 (1-C$_{maj}$), 144.9 (3-C$_{min}$), 144.5 (3-C$_{maj}$), 134.9 (8-C$_{min}$), 134.7 (8-C$_{maj}$), 134.6 (4a-C), 132.5 (7-C$_{maj}$), 132.5 (8a-C), 132.0 (7-C$_{min}$), 129.5 (1C, d, *J* 24.7, 5-C), 126.2 (6-C$_{min}$), 126.1 (6-C$_{maj}$), 119.6 (4-C$_{min}$), 119.5 (4-C$_{maj}$), 116.2 (CN$_{maj}$), 116.1 (CN$_{min}$), 82.3 (^{t}Bu 1-C$_{maj}$), 81.1 (^{t}Bu 1-C$_{min}$), 58.7 (pyrrolidinyl 5-C$_{min}$), 58.5 (pyrrolidinyl 5-C$_{maj}$), 48.4 (pyrrolidinyl 2-C$_{maj}$), 48.3 (pyrrolidinyl 2-C$_{min}$), 32.3 (pyrrolidinyl 4-C$_{min}$), 31.3 (pyrrolidinyl 4-C$_{maj}$), 29.5 (pyrrolidinyl 3-C$_{min}$), 28.4 (^{t}Bu 2-C$_{min}$), 28.3 (^{t}Bu 2-C$_{maj}$), 28.0 (pyrrolidinyl 3-C$_{maj}$). δ_F (565 MHz, $CDCl_3$) 64.7 (SO_2F_{maj}), 64.5 (SO_2F_{min}). λ_{max} (neat)/cm^{-1} 2977, 2933, 1701, 1614, 1581, 1560, 1381, 1209, 1154. HRMS (ESI) $C_{19}H_{19}FN_3O_4S$ requires [M+Na]$^+$, calculated 428.1056, found 428.1055.

1-(*N*-Boc-*N*-cyanomethyl-methyl)isoquinoline-5-sulfonyl fluoride D-4

Following General Procedure B, isoquinoline-8-sulfonyl fluoride (21.1 mg, 0.10 mmol) was reacted 2-(*N*-Boc-*N*-methylamino)-acetonitrile (85.1 mg, 0.5 mmol), following purification by Mass-Directed HPLC from a 10 minute gradient of 30 – 95% acetonitrile in water, collecting at 9.0 mins to isolate the cross coupled product as a brown oil (4.1 mg, 11%, 60:40 mixture of rotamers). δ_H (500 MHz, $CDCl_3$) 8.89 (1H, br. d, *J* 8.4, 3-H$_{maj}$), 8.80 (1H, br. d, *J* 8.4, 3-H$_{min}$), 8.72 (1-H, d *J* 6.2, 8-H), 8.61 (1H, d *J* 6.2, 6-H), 8.39 (1H, br. s, 4-H$_{maj}$), 8.32 (1H, br. s, 4-H$_{min}$), 7.89-7.83 (1H, m, 7-H), 5.26 (2H, br. s, CH_{2maj}), 5.21 (2H, br. s, CH_{2min}),4.48 (2H, br. s, *N*-cyanomethyl$_{min}$), 4.33 (2H, br. s, *N*-cyanomethyl$_{maj}$), 1.53 (9H, br. s, ^{t}Bu 2-H$_3$). δ_C (125 MHz, $CDCl_3$) 156.9 (^{t}Bu C=O), 154.2 (1-C), 143.4 (3-C), 135.8 (8-C), 135.3 (4a-C), 134.1 (7-C), 132.8 (8a-C), 129.6 (1C, d, *J* 25.6, 5-C), 127.0 (6-C), 118.3 (4-C), 115.9 (CN), 82.3 (^{t}Bu 1-C$_{maj}$), 81.1 (^{t}Bu 1-C$_{min}$), 49.3 (*N*-cyano 1-C), 35.9 (Me), 28.3 (^{t}Bu 2-C). δ_F (565 MHz, $CDCl_3$) 65.2. λ_{max} (neat)/cm^{-1} 3086, 2978, 2934, 1811, 1703, 1615, 1581,

1561, 1411, 1208, 1158. HRMS (ESI) $C_{17}H_{18}FN_3O_4S$ requires $[M+Na]^+$, calculated 402.0900, found 402.0885.

1-(2-Oxoazepan-1-yl)methyl-isoquinoline-5-sulfonyl fluoride D-8

Following General Procedure B, isoquinoline-8-sulfonyl fluoride (21.1 mg, 0.10 mmol) was reacted *N*-methylcaprolactam (381.6 mg, 3 mmol), following purification by Mass-Directed HPLC from a 10 minute gradient of 40 – 80% acetonitrile in water, collecting at 7.0 mins to isolate the cross coupled product as a brown oil (7.3 mg, 22%). δ_H (500 MHz, $CDCl_3$) 9.03 (1H, dd, *J* 8.6, 2.2, 3-H), 8.70 (1H, d, *J* 6.2, 8-H), 8.57 (1H, dd, *J* 7.5, 1.2, 6-H), 8.33 (1H, br. dd, *J* 6.1, 1.9, 4-H), 7.82 (1H, t, *J* 7.6, 7-H), 5.28 (2H, s, Me H_2), 3.46 (2H, t, *J* 5.2, aze 7-H_2), 2.62-2.58 (2H, m, aze 3-H_2), 1.65-1.60 (4H, m, aze 4- and 6-H_2), 1.34-1.30 (2H, m, aze 5-H_2). δ_C (125 MHz, $CDCl_3$) 176.0 (C=O), 158.8 (1-C), 143.4 (8-C), 135.3 (3-C), 135.2 (6-C), 132.4 (4a-C), 129.1 (1C, d, *J* 24.7, 5-C), 127.4 (8a-C), 126.7 (7-C), 117.1 (4-C), 50.7 (aze 7-C), 48.2 (aze 3-C), 37.0 (Me), 29.8 (aze 6-C), 27.8 (aze 5-C), 23.3 (aze 4-C). δ_F (565 MHz, $CDCl_3$) 64.9. λ_{max} (neat)/cm^{-1} 3088, 2932, 2857, 1635, 1543, 1408, 1205, 979. HRMS (ESI) $C_{16}H_{17}FN_2O_3S$ requires $[M+H]^+$, calculated 337.1022, found 337.1017.

1-(1,4-Dioxan-2-yl)isoquinoline-5-sulfonyl fluoride D-10a

Following General Procedure B, isoquinoline-8-sulfonyl fluoride (21.1 mg, 0.10 mmol) was reacted 1,4-dioxane (264.3 mg, 3 mmol), following purification by Mass-Directed HPLC from a 10 minute gradient of 40 – 50% acetonitrile in water, isolating two cross coupled products, initially collecting at 11.0 mins as a yellow amorphous solid (2.7 mg, 9%). δ_H (500 MHz, $CDCl_3$) 8.84 (1H, dt, *J* 8.6, 1.1, 3-H), 8.76 (1H, d, *J* 6.1, 8-H), 8.56 (1H, dd, *J* 7.5, 1.1, 6-H), 8.25 (1H, dd, *J* 6.1, 0.9, 4-H), 7.78 (1H, t, *J* 7.5, 7-H), 5.42 (1H, dd, *J* 9.5,3.0, oxo 2-H), 4.21 (2H, m, oxo 3-H_2), 4.11 (2H, m, oxo 6-H_2), 3.89 (2H, m, oxo 5-H_2). δ_C (125 MHz, $CDCl_3$) 157.6 (1-C), 144.6 (3-C), 134.9 (8-C), 134.2 (7-C), 132.5 (8a-C), 129.3 (1C, d, *J* 24.7, 5-C), 127.1 (4a-C), 126.0 (6-C), 117.2 (4-C), 76.1 (oxo 2-C), 70.0 (oxo 3-C) 67.7 (oxo 6-C) 66.6 (oxo 5-C). δ_F (565 MHz, $CDCl_3$) 64.8. λ_{max} (neat)/cm^{-1} 3096, 2958, 2818, 2853, 1755, 1406, 1202, 1065. HRMS (ESI) $C_{13}H_{12}FNO_4S$ requires $[M+H]^+$, calculated 298.0549, found 298.0547.

Also obtained was 1,3-bis(1,4-Dioxan-2-yl)isoquinoline-5-sulfonyl fluoride **D-10b**, isolated at 7.5 min as a pale yellow amorphous solid (1.2 mg, 3%). δ_H (500 MHz, $CDCl_3$) 8.68 (1-H, d, *J* 5.9, 8-H), 8.54 (1H, d, *J* 8.0, 6-H), 8.36 (1H, dd, *J* 5.9, 2.7, 4-H), 8.23 (1H, br. d, *J* 8.0, 7-H), 5.70 (1H, dd, *J* 9.8, 2.3, oxo_1 2-H), 5.20 (1H, t, *J* 5.7, oxo_3 2-H), 4.31 (2H, appt d, *J* 6.2, oxo_3 3-H_2),4.16-4.03 (4H, m, oxo 6-H_2), 3.98-3.81 (4H, m, oxo 5-H_2), 3.40 (2H, dd, *J* 12.0, 9.8, oxo_1 3-H_2). δ_C (125 MHz, $CDCl_3$) 156.0 (1-C), 145.5 (3-C), 143.1 (8-C), 134.3 (6-C), 133.7 (8a-C), 129.2 (1C, d, *J* 24.6, 5-C), 126.2 (7-C) 125.5 (4a-C) 117.4 (4-C). 76.6 (oxo_3 2-C), 75.7 (oxo_1 2-C), 73.9 (oxo_3 3-C), 70.3 (oxo_1 3-C), 68.0 and 67.6 ($oxo_{1,3}$ 6-C), 66.9 and 66.4 ($oxo_{1,3}$ 5-C). δ_F (565 MHz, $CDCl_3$) 64.5. λ_{max} (neat)/cm^{-1}

3119, 2963, 2853, 1752, 1402, 1211, 981. HRMS (ESI) $C_{17}H_{18}FNO_6S$ requires $[M+H]^+$, calculated 384.0917, found 384.0923.

1-[1-(*N*-Ethylacetamido)ethyl]isoquinoline-5-sulfonyl fluoride D-13

Following General Procedure B, isoquinoline-8-sulfonyl fluoride (21.1 mg, 0.10 mmol) was reacted *N,N*-diethylacetamide (57.6 mg, 0.5 mmol), following purification by Mass-Directed HPLC from a 10 minute gradient of 5 – 95% acetonitrile in water, collecting at 9.0 mins to isolate the cross coupled product as a brown oil (2.8 mg, 8%). δ_H (500 MHz, $CDCl_3$) 8.89 (1H, dt, *J* 8.7,1.1, 3-H), 8.75 (1H, d, *J* 6.1, 8-H), 8.55 (1H, dd, *J* 7.4, 1.1, 6-H), 8.30 (1H, dd, *J* 6.1, 2.8, 4-H), 7.80 (1H, t, *J* 8.1, 7-H), 6.90 (1H, q, *J* 6.8, Et 1-H), 3.29 (1H, m, *N*-Et 1-Ha), 3.35 (1-H, m, *N*-Et 1-Hb), 2.15 (3H, s, Ac 2-H_3), 1.69 (3H, d, *J* 6.8, Et 2-H_3), 0.6 (3H, t, *J* 7.2 *N*-Et 2-H_3). δ_C (125 MHz, $CDCl_3$) 170.6 (Ac C=O), 160.9 (1-C), 144.1 (3-C), 135.0 (8-C), 134.3 (7-C), 132.2 (8a-C), 129.2 (1C, d, *J* 24.5, 5-C), 127.5 (4a-C), 126.7 (6-C), 116.8 (4-C), 48.9 (Et 1-C), 38.4 (*N*-Et 1-C), 21.7 (Ac 2-C), 16.6 (Et 2-C), 15.9 (*N*-Et 2-C). δ_F (565 MHz, $CDCl_3$) 64.8. λ_{max} (neat)/cm^{-1} 3139, 2980, 2936, 1649, 1576 1410, 1201, 978. HRMS (ESI) $C_{15}H_{17}FN_2O_3S$ requires $[M+H]^+$, calculated 325.1022, found 325.1009.

1-(*N*-Phenyl-6-methylcarbonylpiperidin-2-yl)isoquinoline-5-sulfonyl fluoride D-17

Following General Procedure B, isoquinoline-8-sulfonyl fluoride (21.1 mg, 0.10 mmol) was reacted 2-methyl 1-phenyl piperidine-1,2-dicarboxylate (131.6 mg, 0.5 mmol), following purification by Mass-Directed HPLC from a 15 minute gradient of 5 – 95% acetonitrile in water, collecting at 14.5 mins to isolate the cross coupled product as a brown oil (3.3 mg, 7%). δ_H (500 MHz, $CDCl_3$) 8.72 (1H, d, *J* 5.9, 3-H), 8.55 (1H, d, *J* 7.5, 8-H), 8.39 (1H, dd, *J* 7.5, 2.8, 6-H), 8.19 (1H, dd, *J* 6.1, 2.8, 4-H), 7.82 (1H, t, *J* 8.1, 7-H), 7.38 (5H, m, Cbz Ph), 5.23 (2H, s, Cbz CH_2), 5.15 (1H, m, pip 2-H), 5.09 (1H, m, pip 6-H), 3.80 (3H, s, Me), 2.26-1.89 (6H, m, pip 3-, 4- and 5-H). δ_C (125 MHz, $CDCl_3$) 172.5 (Me C=O), 171.7 (Cbz C=O), 164.0 (1-C), 144.8 (3-C), 134.8 (8-C), 133.2 (7-C), 132.4 (8a-C), 128.7 (Cbz 3- and 5-C), 128.6 (Cbz 4-C), 128.4 (1C, d, *J* 23.8, 5-C), 128.1 (Cbz 2- and 6-C), 126.7 (4a-C), 126.0 (6-C), 115.7 (4-C), 67.7 (Cbz CH_2), 67.0 (Me), 55.6 (pip 2-C), 42.0 (pip 6-C), 30.1 (pip 3-C), 24.8 (pip 5-C), 20.9 (pip 4-C). δ_F (565 MHz, $CDCl_3$) 63.8. λ_{max} (neat)/cm^{-1} 3064, 2945, 2867, 1721, 1675, 1409, 1205. HRMS (ESI) $C_{24}H_{23}FN_2O_6S$ requires $[M+H]^+$, calculated 487.1339, found 487.1335.

1-[*N*-(furan-2-carbonyl)azetidin-2-yl]isoquinoline-5-sulfonyl fluoride D-18

Following General Procedure B, isoquinoline-8-sulfonyl fluoride (21.1 mg, 0.10 mmol) was reacted 1-(furan-2-carbonyl)azetidine (75.6 mg, 0.5 mmol), following purification by Mass-Directed HPLC from a 15 minute gradient of 5 – 95% acetonitrile in water, collecting at 11.0 mins to isolate the cross coupled product as a brown oil (5.7 mg, 16%). δ_H (500 MHz, $CDCl_3$) 8.93 (1H, appt d, *J* 8.5, 3-H),

8.84 (1H, d, *J* 6.0, 8-H), 8.58 (1H, d, *J* 7.5, 6-H), 8.29 (1H, br. s, 4-H), 7.81 (1H, t, *J* 8.0, 7-H), 7.52 (1H, br. s, fur 3-H), 7.05 (1H, br. s, fur 5-H), 6.48 (1H, br. s, fur 4-H), 4.91 (1H, m, aze 2-H), 4.68 (2H, m, aze 4-H_2), 2.86 (2H, m, aze 3-H_2). δ_C (125 MHz, $CDCl_3$) 162.1 (C=O), 159.3 (1-C), 145.5 (3-C), 144.9 (fur 3-C), 134.8 (8-C), 133.6 (7-C), 132.3 (8a-C), 129.2 (1C, d, *J* 24.4, 5-C), 126.7 (4a-C), 125.8 (6-C), 116.7 (4-C), 116.2 (fur 4-C), 111.8 (fur 5-C), 60.0 (aze 2-C), 51.4 (aze 4-C), 24.1 (aze 3-C). δ_F (565 MHz, $CDCl_3$) 62.8. λ_{max} (neat)/cm^{-1} 3100, 3014, 2966, 2933, 2893, 1747, 1624, 1411, 1204, 1135. HRMS (ESI) $C_{17}H_{13}FN_2O_4S$ requires $[M+Na]^+$, calculated 383.0478, found 383.0471.

1-[(3R)-1-cyclopropanecarbonyl-3-fluoropyrrolidin-2-yl]isoquinoline-5-sulfonyl fluoride D-19a

Following the general procedure (**Section 5.2.2**), isoquinoline-8-sulfonyl fluoride (21.1 mg, 0.10 mmol) was reacted (3S)-1-cyclopropanecarbonyl-3-fluoropyrrolidine (78.6 mg, 0.5 mmol), following purification by Mass-Directed HPLC from a 20 minute gradient of 20 – 60% acetonitrile in water, isolating two cross coupled products, initially collecting at 12.5 mins, **D-19a** as a brown amorphous solid (1.0 mg, 3%, 50:50 mixture of rotamers). δ_H (500 MHz, $CDCl_3$) 8.78 (1H, appt d, *J* 6.3, 3-H), 8.64 (1H, d, *J* 8.4, 8-H), 8.60 (1H, d, *J* 7.3, 6-H), 8.31 (1H, br. s, 4-H), 7.83 (1H, t, *J* 7.9, 7-H), 6.24 (1H, m, pyrrolidinyl 2-H), 5.51 (1H, br. s, pyrrolidinyl 3-H_{Rota}), 5.40 (1H, br. s, pyrrolidinyl 3-H_{Rotb}), 4.58 (1H, m, pyrrolidinyl 5-H_a), 4.31 (1H, m, pyrrolidinyl 5-H_b), 2.86 (1H, m, pyrrolidinyl 4-H_a), 2.45 (1H, m, pyrrolidinyl 4-H_b), 1.03 (1H, m, prop 1-H), 0.91 (4H, m, prop 2- and 3-H_2). δ_C (125 MHz, $CDCl_3$) 173.0 (C=O), 160.6 (1-C), 145.4 (3-C), 134.5 (8-C), 132.8 (7-C), 131.9 (8a-C), 129.0 (1C, d, *J* 24.3, 5-C), 126.2 (6-C), 125.9 (4a-C), 116.3 (4-C), 92.2 (1C, d, *J* 179.9 3-C), 57.6 (1C, d, *J* 25.2, 3-C), 54.0 (5-C), 38.9 (1C, d, *J* 21.2, 4-C), 13.1 (prop 1-C), 8.4 and 8.3 (prop 2- or 3-C). δ_F (565 MHz, $CDCl_3$) 63.0 (SO_2F), -172.3 (pyrrolidinyl CF). λ_{max} (neat)/cm^{-1} 3066, 2929, 1629, 1583, 1402, 1201, 1098. HRMS (ESI) $C_{17}H_{16}F_2N_2O_3S$ requires $[M+Na]^+$, calculated 389.0747, found 389.0742.

Also obtained was 1-[(4S)-1-cyclopropanecarbonyl-4-fluoropyrrolidin-2-yl]isoquinoline-5-sulfonyl fluoride **D-19b**, isolated at 15.5 mins as a brown amorphous solid (1.7 mg, 5%, 50:50 mixture of rotamers). δ_H (500 MHz, $CDCl_3$) 8.99 (1H, appt d, *J* 8.6, 3-H), 8.72 (1H, d, *J* 6.1, 8-H), 8.59 (1H, d, *J* 7.5, 6-H), 8.28 (1H, br. s, 4-H), 7.83 (1H, t, *J* 8.0, 7-H), 6.20 (1H, t, *J* 8.1, pyrrolidinyl 2-H), 5.63 (1H, br. s, pyrrolidinyl 4-H_{Rota}), 5.53 (1H, br. s, pyrrolidinyl 4-H_{Rotb}), 4.44 (1H, m, pyrrolidinyl 5-H_a), 4.29 (1H, appt dd, *J* 12.6, 10.5, pyrrolidinyl 5-H_b), 2.74 (1H, m, pyrrolidinyl 3-H_a), 2.60-2.55 (1H, m, pyrrolidinyl 3-H_b), 1.70-1.65 (1H, m, prop 1-H), 0.87-0.76 (4H, m, prop 2- and 3-H_2). δ_C (125 MHz, $CDCl_3$) 172.8 (C=O), 162.3 (1-C), 145.9 (3-C), 135.4 (8-C), 133.5 (7-C), 132.5 (8a-C), 129.3 (1C, d, *J* 25.2, 5-C), 127.0 (6-C), 126.3 (4a-C), 116.7 (4-C), 92.8 (1C, d, *J* 177.6 4-C), 56.0 (2-C), 54.2 (1C, d, *J* 22.5, 5-C), 31.2 (1C, d, *J* 21.2, 3-C), 13.0 (prop 1-C), 8.3 and 7.8 (prop 2- or 3-C). δ_F (565 MHz,

$CDCl_3$) 62.8 (SO_2F), -176.2 (pyrrolidinyl CF) λ_{max} (neat)/cm^{-1} 3086, 3061, 3003, 2977, 1629, 1402, 1200, 1087. HRMS (ESI) $C_{17}H_{16}F_2N_2O_3S$ requires $[M+Na]^+$, calculated 389.0747, found 389.0740.

2-[*N*-Boc-(2R)-5-cyano-pyrrolidin-2-yl]-1,3-benzothiazole S2a

Following General Procedure B, 1,3-benzothiazole (13.5 mg, 0.10 mmol) was reacted with *N*-Boc-2-cyano-pyrrolidine (98.1 mg, 0.5 mmol), following purification by Mass-Directed HPLC from a 15 minute gradient of 20 – 60% acetonitrile in water, isolating two cross coupled products, initially collecting at 11.0 mins as a pale brown amorphous solid (8.9 mg, 27%, 55:45 mixture of rotamers). δ_H (500 MHz, $CDCl_3$) 7.96 (1H, dd, *J* 8.2, 1.9, 7-H), 7.88 (1H, br. s, 4-H), 7.48 (1H, br. s, 6-H), 7.39 (1H, br. s, 5-H), 5.41 (1H, br. s, pyrrolidinyl 2-H_{maj}), 5.27 (1H, br. s, pyrrolidinyl 2-H_{min}), 4.77 (1H, br. s, pyrrolidinyl 5-H_{maj}), 4.61 (1H, br. s, pyrrolidinyl 5-H_{min}), 2.56-2.45 (4H, m, pyr, 3- and 4-H), 1.62 (9H, s, ^{t}Bu 2-H_{min}), 1.56 (9H, s, ^{t}Bu 2-H_{maj}). δ_C (125 MHz, $CDCl_3$) 173.4 (^{t}Bu C=O_{min}), 172.7 (^{t}Bu C=O_{maj}), 153.4 (2-C_{maj}), 153.3 (2-C_{min}), 152.9 (7a-C_{min}), 152.8 (7a-C_{maj}), 135.0 (3a-C_{maj}), 134.6 (3a-C_{min}), 126.5 (6-C_{min}), 126.3 (6-C_{maj}), 125.4 (5-C_{min}), 125.3 (5-C_{maj}), 123.2 (7-C), 121.9 (4-C_{min}), 121.8 (4-C_{maj}), 118.9 (CN_{maj}), 118.7 (CN_{min}), 82.9 (^{t}Bu C_{1maj}), 82.5 (^{t}Bu C_{1min}), 59.6 (pyrrolidinyl 5-C_{min}), 59.2 (pyrrolidinyl 5-C_{maj}), 48.4 (pyrrolidinyl 2-C_{maj}), 48.1 (pyrrolidinyl 2-C_{min}), 32.7(pyrrolidinyl 4-C_{min}), 31.4 (pyrrolidinyl 4-C_{maj}), 29.9(pyrrolidinyl 3-C_{maj}), 28.8(pyrrolidinyl 3-C_{min}), 28.4 (^{t}Bu C_{2maj}), 28.2 (^{t}Bu C_{2min}). λ_{max} (neat)/cm^{-1} 3042, 2993, 2921, 1722, 1409. HRMS (ESI) $C_{17}H_{19}N_3O_2S$ requires $[M+H]^+$, calculated 330.1276, found 330.1265.

Also obtained was 2-[*N*-Boc-(2S)-5-cyano-pyrrolidin-2-yl]-1,3-benzothiazole **S2b**, isolated at 12.0 mins as a pale brown solid (5.3 mg, 16%, 55:45 mixture of rotamers). δ_H (500 MHz, $CDCl_3$) 7.97 (1H, d, *J* 8.2, 7-H), 7.84 (1H, dd, *J* 8.0, 7.5 4-H), 7.47 (1-H, ddd, *J* 8.0, 7.7, 4.3, 6-H), 7.38 (1H, ddd, *J* 8.0, 7.7, 4.3 5-H), 5.43 (1H, d, *J* 8.0, pyrrolidinyl 2-H_{maj}), 5.32 (1H, d, *J* 8.0, pyrrolidinyl 2-H_{min}), 4.85 (1H, d, *J* 8.1, pyrrolidinyl 5-H_{maj}), 4.74 (1H, d, *J* 8.1, pyrrolidinyl 5-H_{min}), 2.55-2.30 (4H, m, pyr, 3- and 4-H), 1.54 (9H, s, ^{t}Bu 2-H_{maj}), 1.31 (9H, s, ^{t}Bu 2-H_{min}). δ_C (125 MHz, $CDCl_3$) 173.6 (^{t}Bu C=O_{min}), 172.7 (^{t}Bu C=O_{maj}), 153.8 (2-C_{maj}), 153.5 (2-C_{min}), 153.1 (7a-C_{min}), 152.8 (7a-C_{maj}), 135.2 (3a-C_{maj}), 135.0 (3a-C_{min}), 126.4 (6-C_{min}), 126.3 (6-C_{maj}), 125.4 (5-C_{min}), 125.3 (5-C_{maj}), 123.2 (7-C), 121.9 (4-C_{min}), 121.8 (4-C_{maj}), 118.8 (CN_{maj}), 118.7 (CN_{min}), 83.1 (^{t}Bu C_{1maj}), 82.7 (^{t}Bu C_{1min}), 61.0 (pyrrolidinyl 5-C_{min}), 60.3 (pyrrolidinyl 5-C_{maj}), 48.2 (pyrrolidinyl 2-C_{maj}), 48.1 (pyrrolidinyl 2-C_{min}), 32.7(pyrrolidinyl 4-C_{min}), 31.4 (pyrrolidinyl 4-C_{maj}), 29.9(pyrrolidinyl 3-C_{maj}), 28.8(pyrrolidinyl 3-C_{min}), 28.4 (^{t}Bu C_{2maj}), 28.2 (^{t}Bu C_{2min}). λ_{max} (neat)/cm^{-1} 3078, 2985, 2942, 1698, 1412. HRMS (ESI) $C_{17}H_{19}N_3O_2S$ requires $[M+H]^+$, calculated 330.1276, found 330.1264.

2-[N-Boc-(2R)-5-cyano-pyrrolidin-2-yl]-1,3-benzothiazole

Following General Procedure B, 1,3-benzothiazole (13.5 mg, 0.10 mmol) was reacted with *N*-Boc-2-cyano-pyrrolidine (98.1 mg, 0.5 mmol) to form, following purification by mass-directed HPLC, the

cross-coupled product as a pale brown solid (8.86 mg, 27%, 45:55 rot_{min}:rot_{maj}). δ_H (500 MHz, d-chloroform) 7.97 (1H, dd, *J* 8.2, 1.9, 7-H), 7.88 (1H, br. s, 4-H), 7.47 (1H, br. s, 6-H), 7.39 (1H, br. s, 5-H), 5.41 (1H, br. s, pyr 2-H_{maj}), 5.27 (1H, br. s, pyr 2-H_{min}), 4.77 (1H, br. s, pyr 5-H_{maj}), 4.61 (1H, br. s, pyr 5-H_{min}), 2.56-2.45 (4H, m, pyr, 3- and 4-H), 1.62 (9H, s, ^{t}Bu 2-H_{min}), 1.56 (9H, s, ^{t}Bu 2-H_{maj}). δ_C (125 MHz, d^6-chloroform) 173.5 (^{t}Bu C=O_{min}), 172.7 (^{t}Bu C=O_{maj}), 153.4 (2-C_{maj}), 153.3 (2-C_{min}), 152.9 (7a-C_{min}), 152.8 (7a-C_{maj}), 135.0 (3a-C_{maj}), 134.6 (3a-C_{min}), 126.5 (6-C_{min}), 126.3 (6-C_{maj}), 125.4 (5-C_{min}), 125.3 (5-C_{maj}), 123.2 (7-C), 121.9 (4-C_{min}), 121.8 (4-C_{maj}), 118.9 (CN_{maj}), 118.7 (CN_{min}), 82.9 (^{t}Bu C_{1maj}), 82.5 (^{t}Bu C_{1min}), 59.6 (pyr 5-C_{min}), 59.2 (pyr 5-C_{maj}), 48.4 (pyr 2-C_{maj}), 48.1 (pyr 2-C_{min}), 32.7(pyr 4-C_{min}), 31.4 (pyr 4-C_{maj}), 29.9(pyr 3-C_{maj}), 28.8(pyr 3-C_{min}), 28.4 (^{t}Bu C_{2maj}), 28.2 (^{t}Bu C_{2min}). vmax (neat)/cm^{-1} 3042, 2993, 2921, 1722, 1409. HRMS (ESI): $C_{17}H_{19}N_3O_2S$ requires $[M+H]^+$, calculated 330.1276, found 330.1265.

6-(*N*-Poc-pyrrolidin-2-yl)pyridine-3-sulfonyl fluoride C-1alk

Deprotection of 6-(*N*-Boc-pyrrolidin-2-yl)pyridine-3-sulfonyl fluoride, **C-1**, (4 mg, 0.012 mmol) was carried out using TFA (20% in DCM), stirring for 2 hours at room temperature. The solution was concentrated *in vacuo* to remove excess TFA, before resuspending in DCM (1 mL). The solution was cooled to 0 ºC, and the propargyl chloride (2 µL, 0.024 mmol) and triethylamine (4 µL, 0.026 mmol) were added dropwise. The reaction was allowed to warm to room temperature and left to stir overnight. The mixture was concentrated *in vacuo*, dissolved in acetonitrile, and purified via Mass-Directed HPLC with acetonitrile to water as a gradient of 5-95%, the eluted samples were lyophilised to give the pure product as a brown oil (1.6 mg, 42%, 60:40 mixture of rotamers). δ_H (500 MHz, $CDCl_3$) 9.13 (1H, s, 2-H), 8.22 (1H, m, 5-H), 7.49 (1H, appt dd, *J* 8.4, 12.7, 4H), 5.10 (1H, appt td, *J* 3.5, 8.4, pyrrolidinyl 2-H), 4.68 (1H, appt qd, *J* 2.0, 12.2, 5-H_{maj}), 4.55 (1H, appt qd, *J* 2.0, 12.2, 5-H_{min}), 3.71 (2H, m, poc 1-H_2), 2.47 (1H, t, *J* 2.6, poc- 3-H), 2.39 (2H, m, pyrrolidinyl 3- and 4-H_a), 2.03 (2H, m, pyrrolidinyl 3- and 4-H_b). δ_C (125 MHz, $CDCl_3$) 170.6 (poc C=O_{min}), 169.9 (poc C=O_{maj}), 154.4 (6-C_{maj}), 153.9 (6-C_{min}),149.1 (2-C), 136.8 (4-C), 128.5 (1C, d, *J* 25.4, 3-C), 121.4 (5-C_{maj}), 120.8 (5-C_{min}).78.4 (poc 2-C_{maj}), 78.1 (poc 2-C_{min}), 74.8 (poc 3-C_{maj}), 74.6 (poc 3-C_{min}), 63.0 (pyrrolidinyl 2-C_{maj}), 62.6 (pyrrolidinyl 2-C_{min}), 53.2 (pyrrolidinyl 5-C_{maj}), 52.9 (pyrrolidinyl 5-C_{min}), 48.0 (poc 1-C_{min}), 47.6 (poc 1-C_{maj}), 34.2 (pyrrolidinyl 3-C_{min}), 33.0 (pyrrolidinyl 3-C_{maj}), 24.3 (pyrrolidinyl 4-C_{maj}), 23.3 (pyrrolidinyl 4-C_{min}). δ_F (565 MHz, $CDCl_3$) 68.7. λ_{max} (neat)/cm^{-1} 3104, 3054, 2938, 2214, 1749, 1619, 1409,1221, 1153. HRMS (ESI) $C_{13}H_{13}FN_2O_4S$ requires $[M+H]^+$, calculated 313.0658, found 313.0650.

500 MHz ^{1}H spectrum in $CDCl_3$

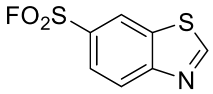

HA A

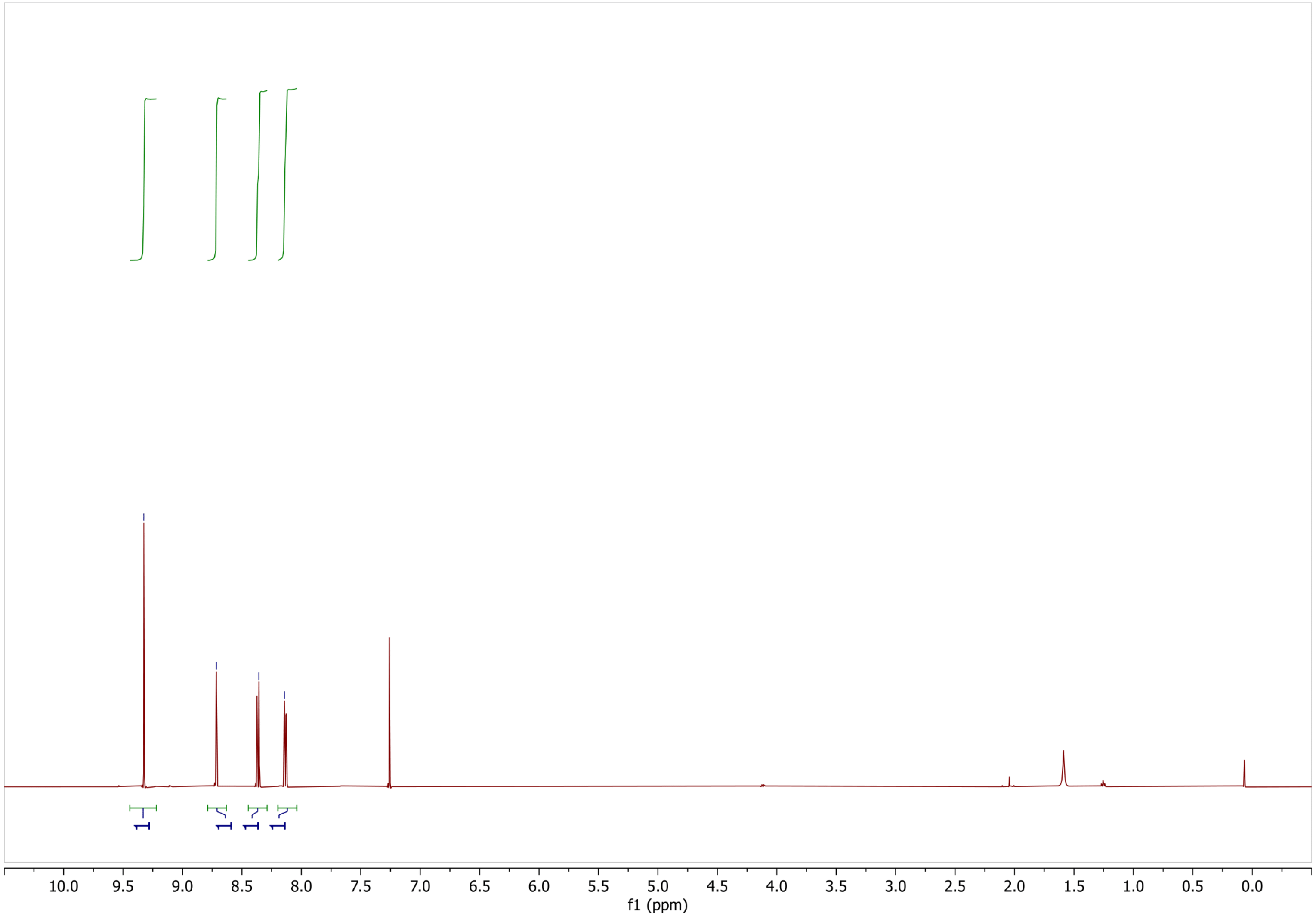

125 MHz ^{13}C spectrum in $CDCl_3$

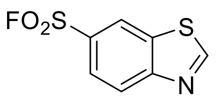

HA A

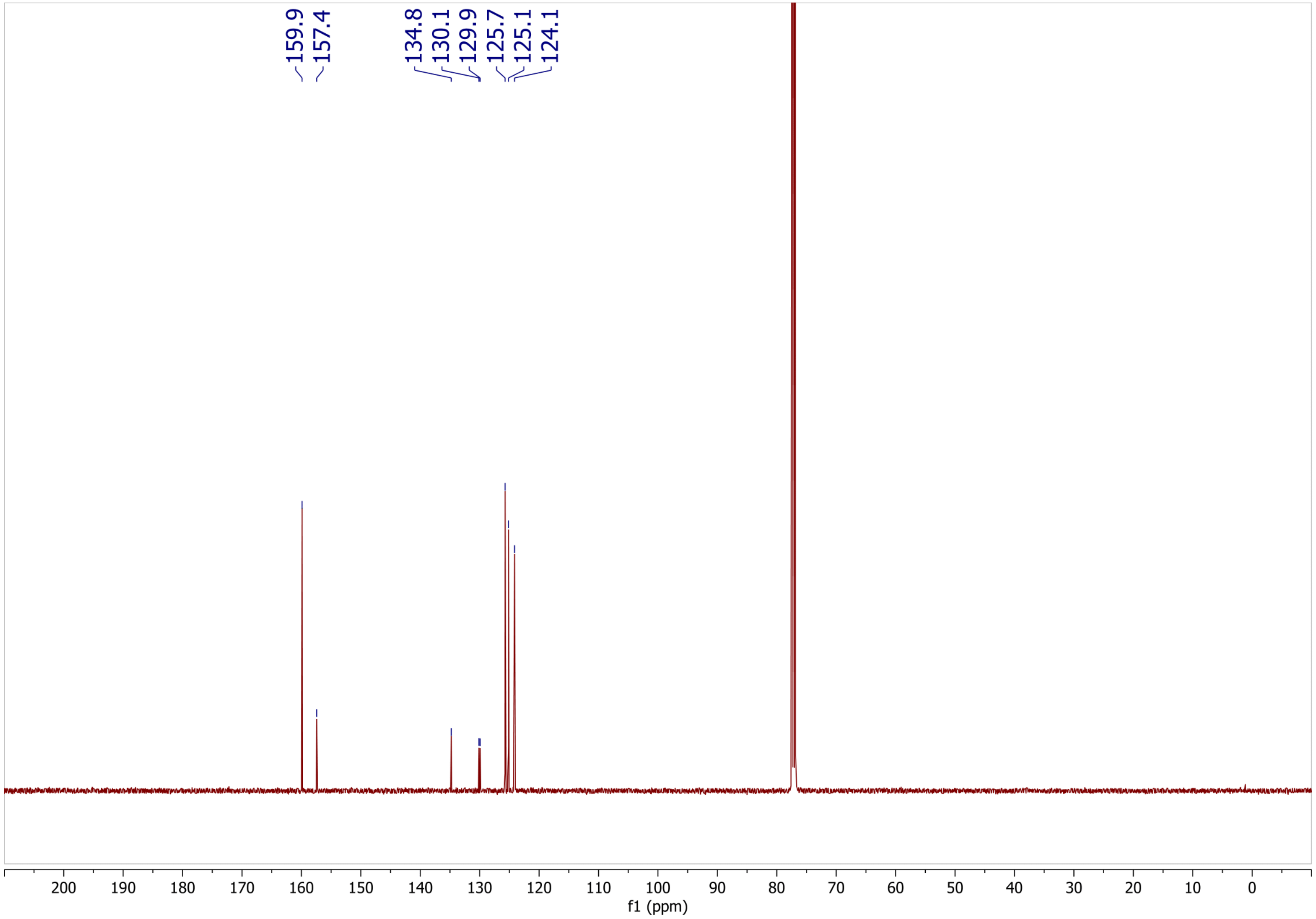

565 MHz ^{19}F spectrum in $CDCl_3$

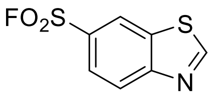

HA A

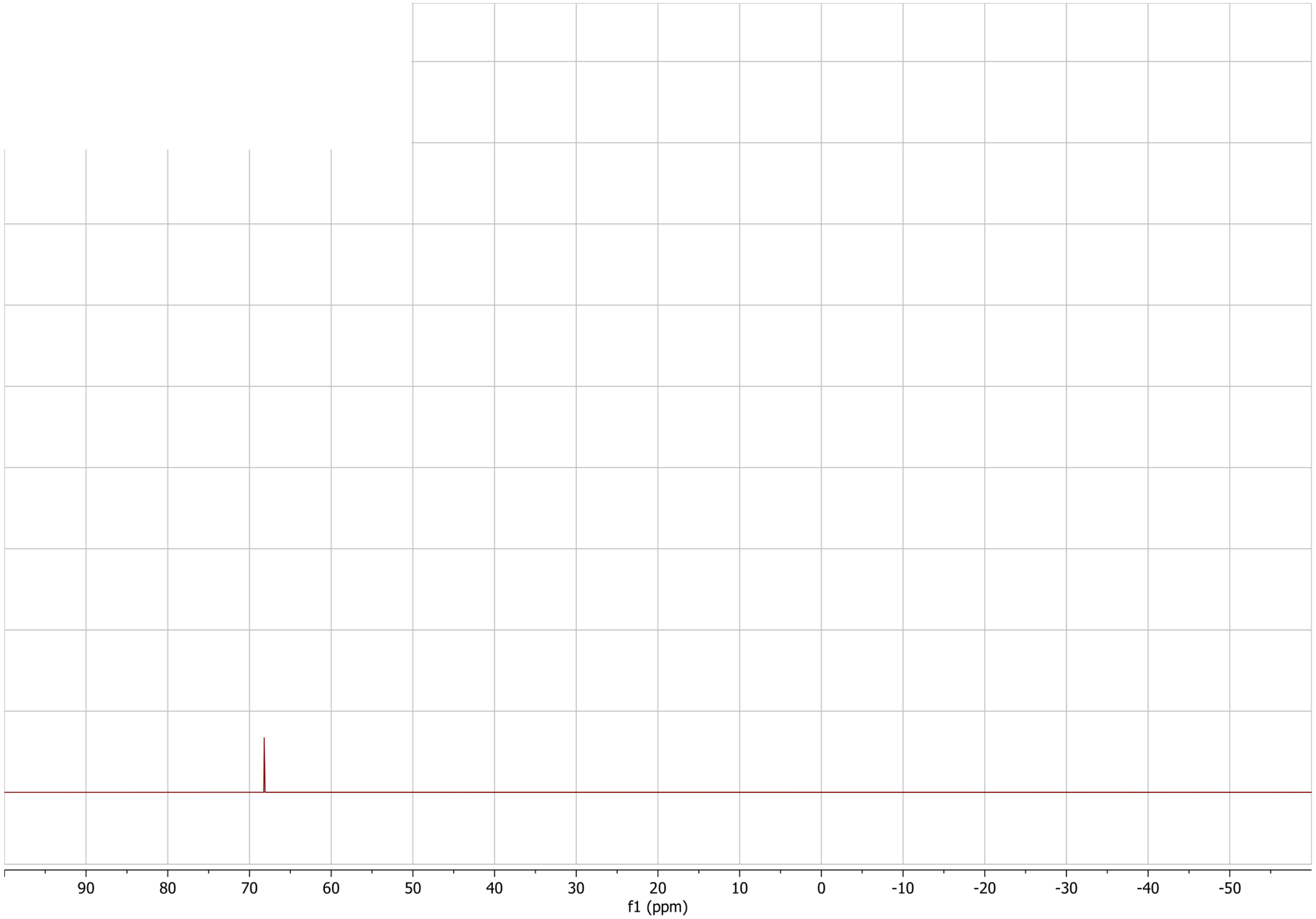

500 MHz ^1H spectrum in $CDCl_3$

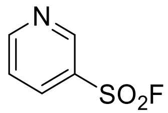

HA C

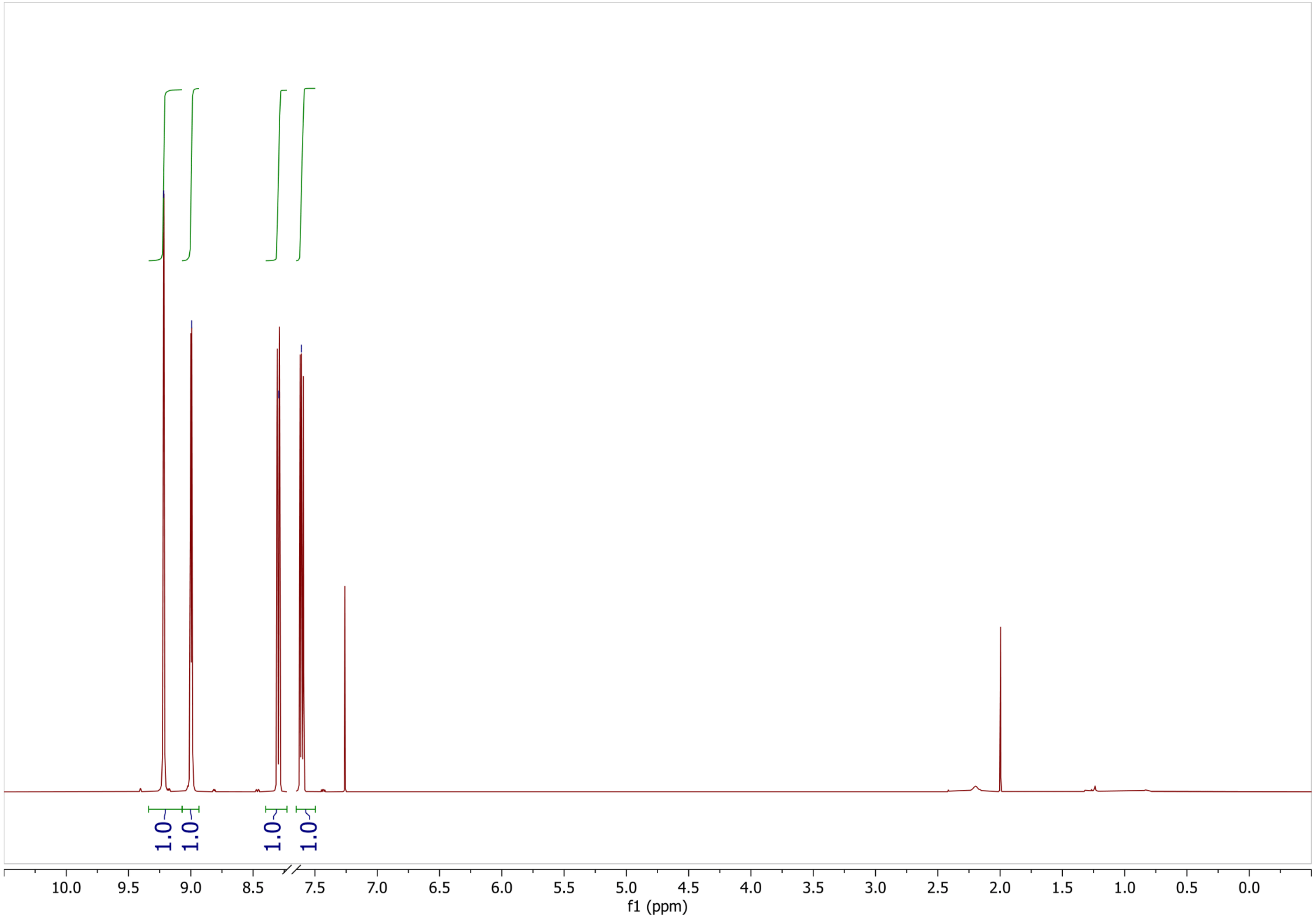

125 MHz ^{13}C spectrum in $CDCl_3$

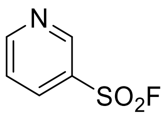

HA C

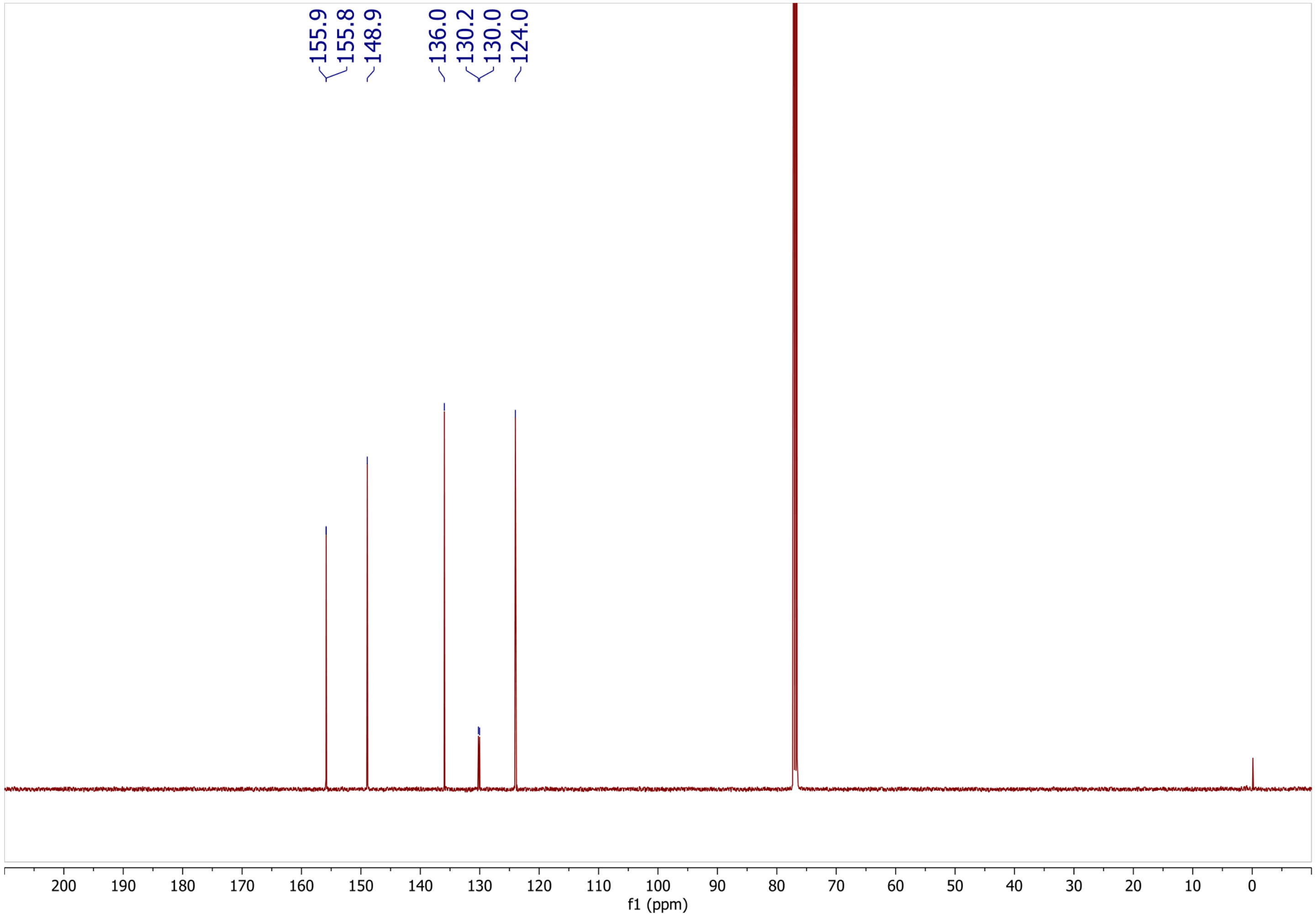

565 MHz ^{19}F spectrum in $CDCl_3$

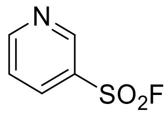

HA C

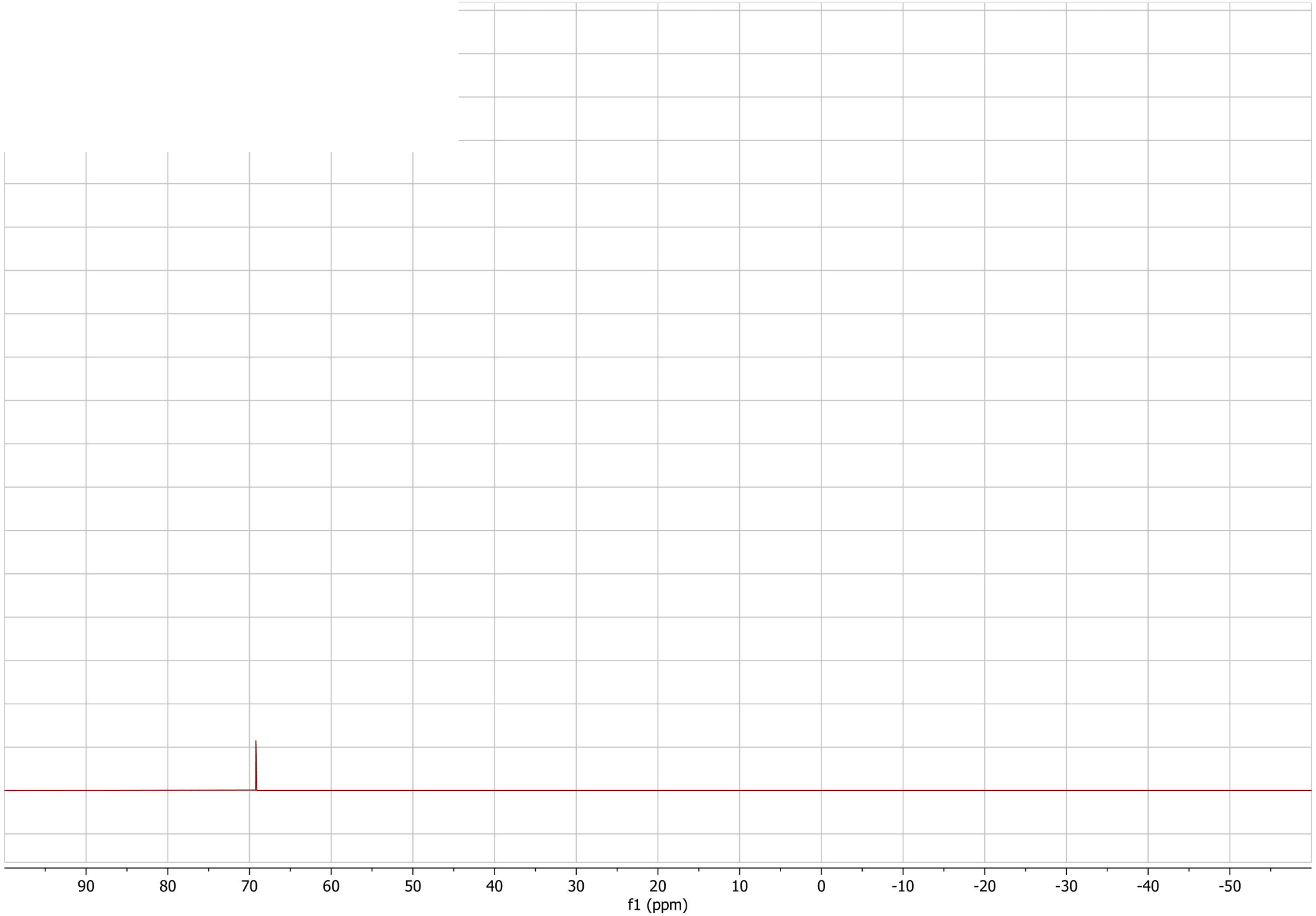

500 MHz ^{1}H spectrum in $CDCl_3$

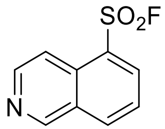

HA D

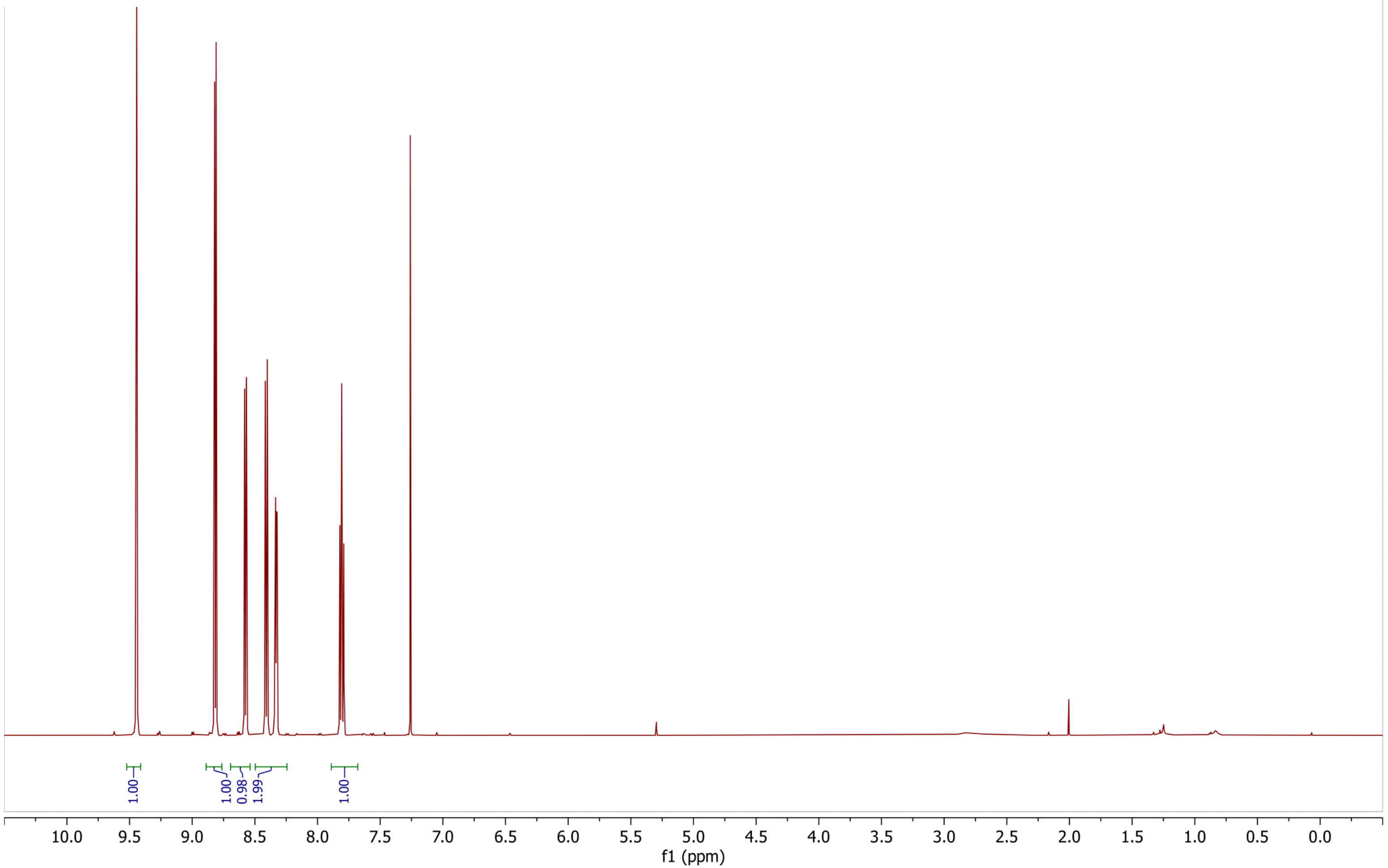

125 MHz ^{13}C spectrum in $CDCl_3$

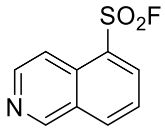

HA D

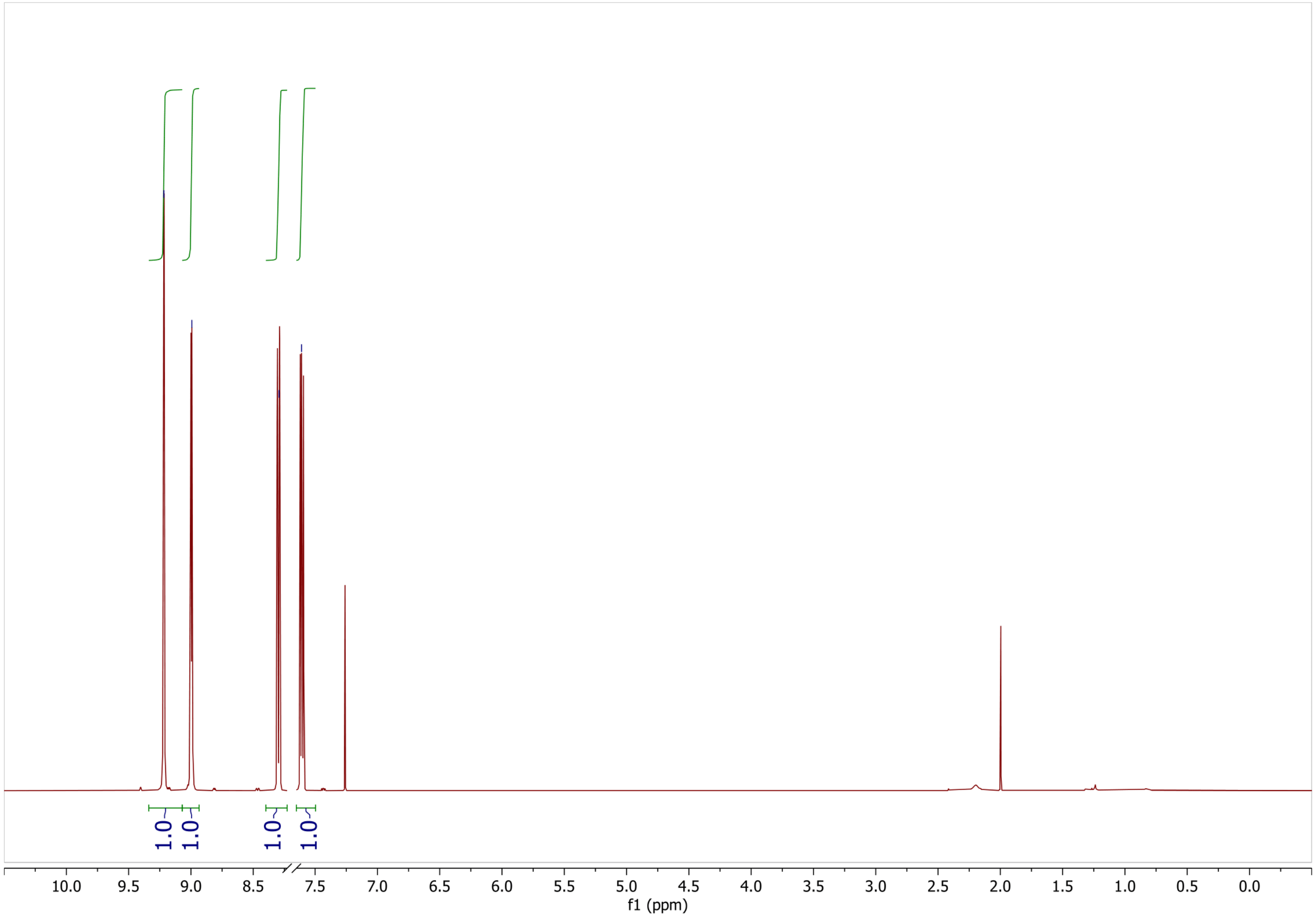

500 MHz ^{1}H spectrum in $CDCl_3$

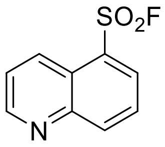

HA E

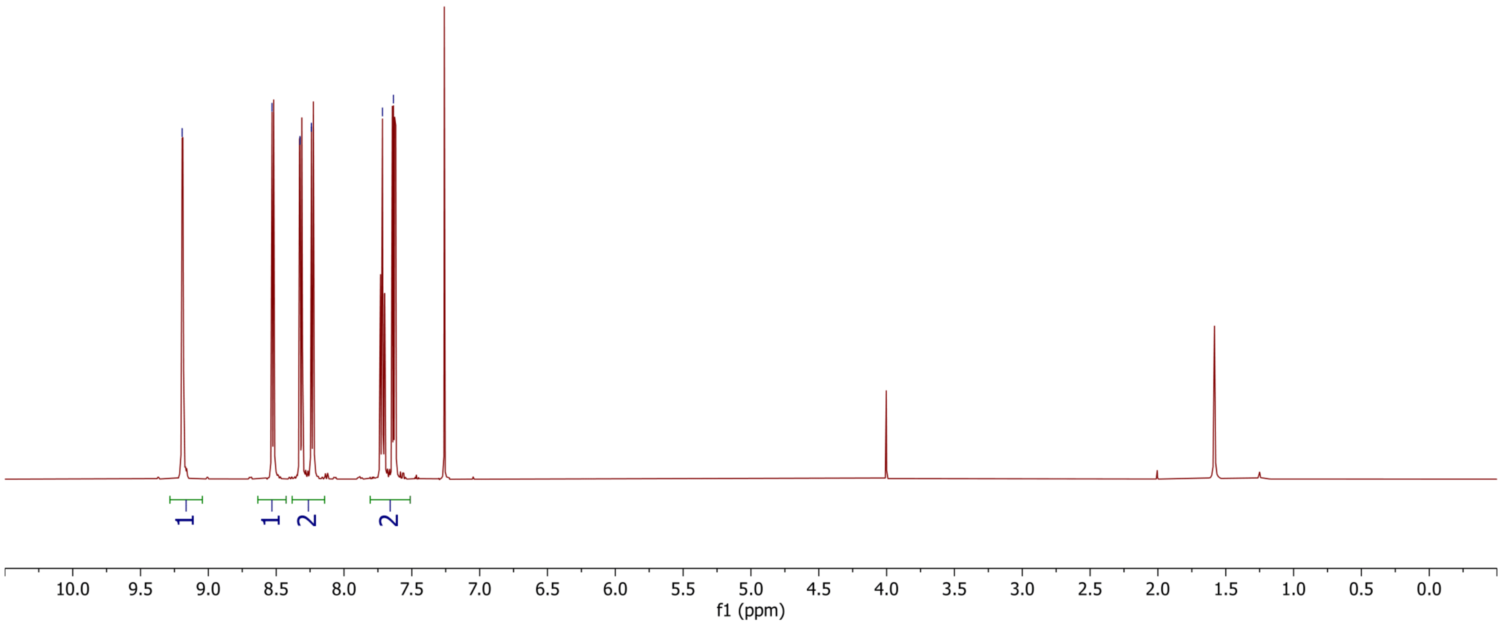

125 MHz ^{13}C spectrum in $CDCl_3$

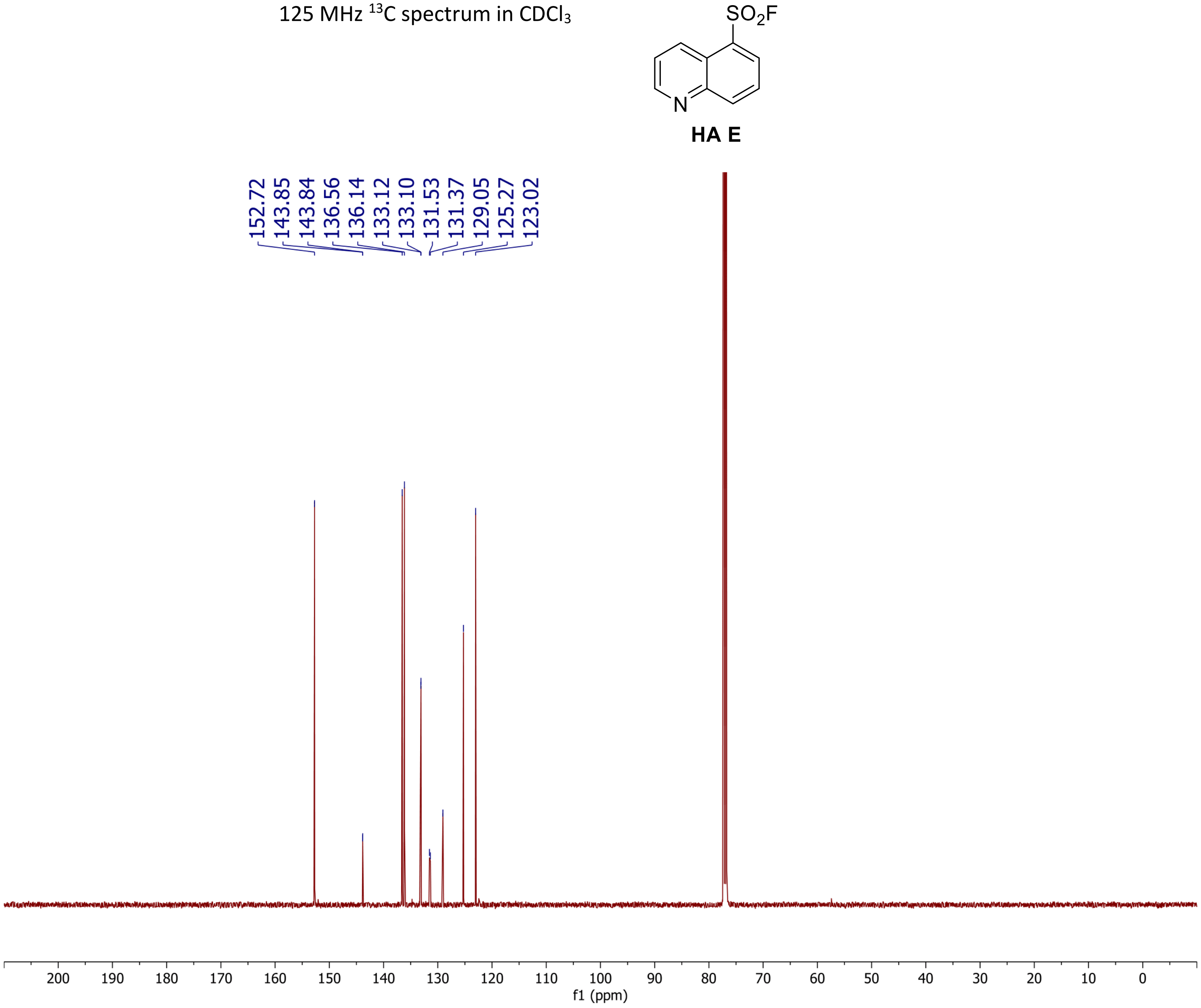

HA E

500 MHz ^1H spectrum in $CDCl_3$

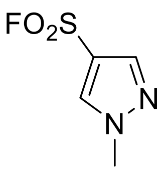

HA F

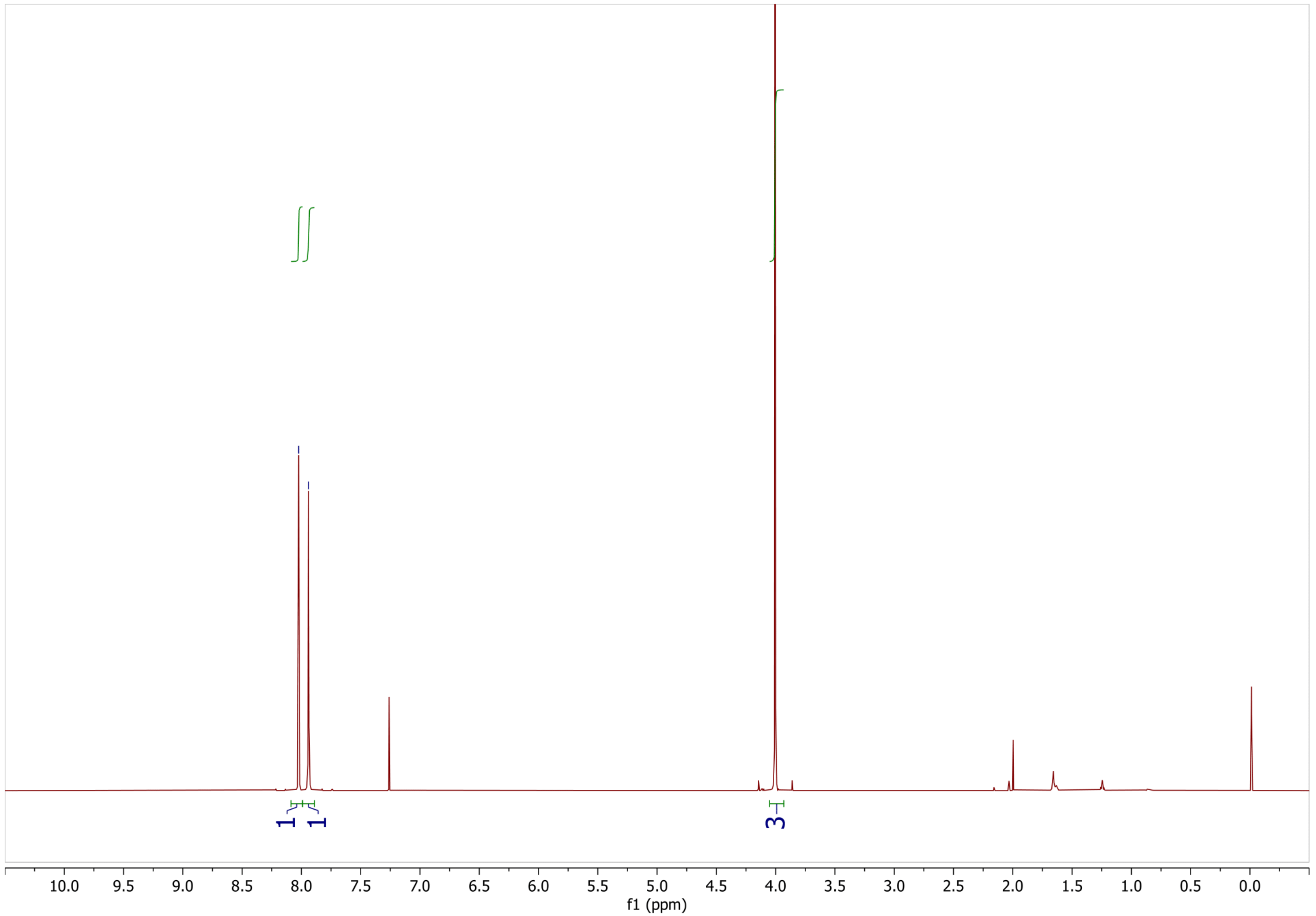

125 MHz ^{13}C spectrum in $CDCl_3$

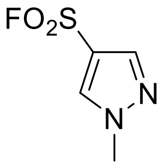

HA F

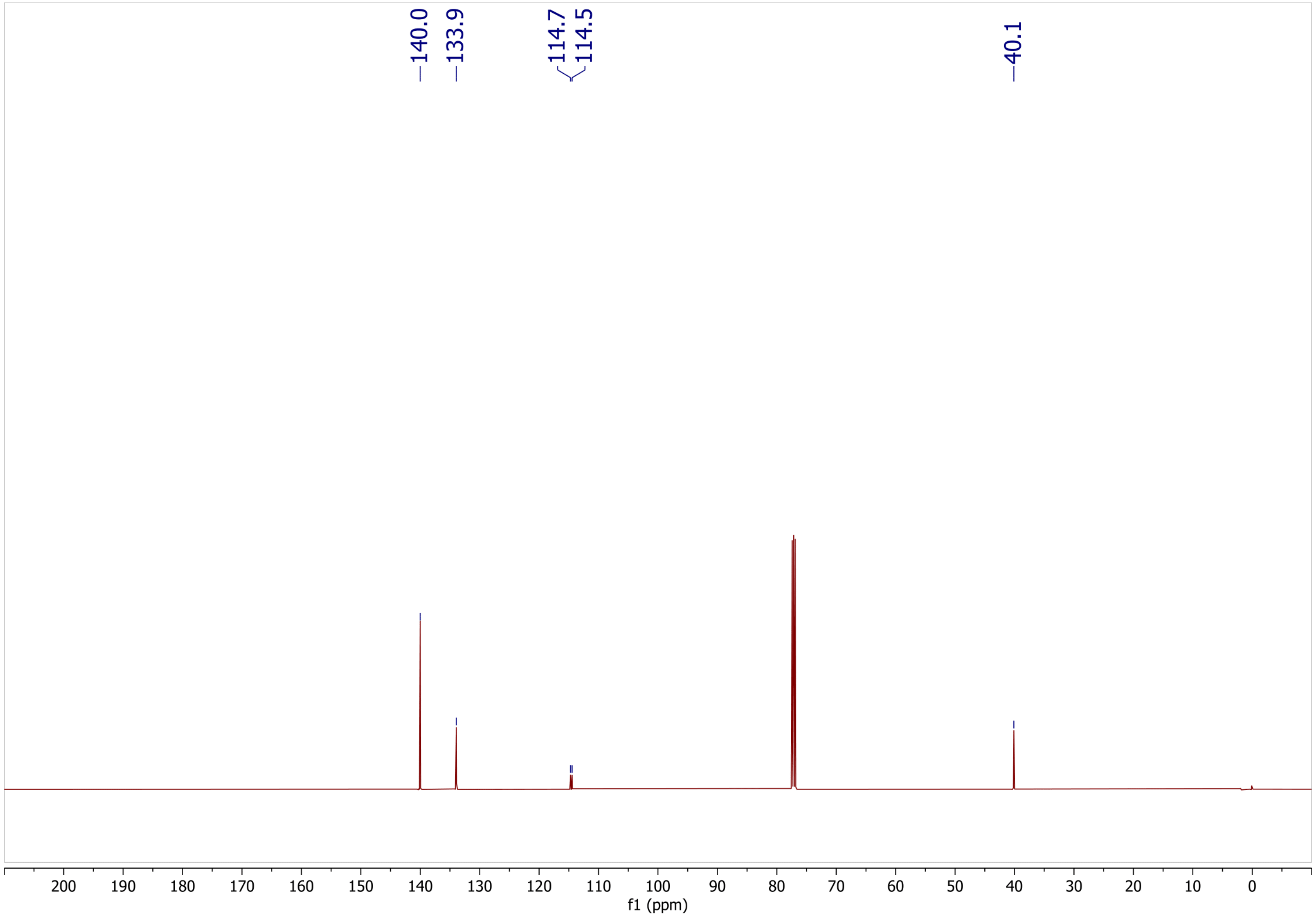

565 MHz ^{19}F spectrum in $CDCl_3$

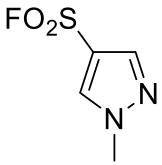

HA F

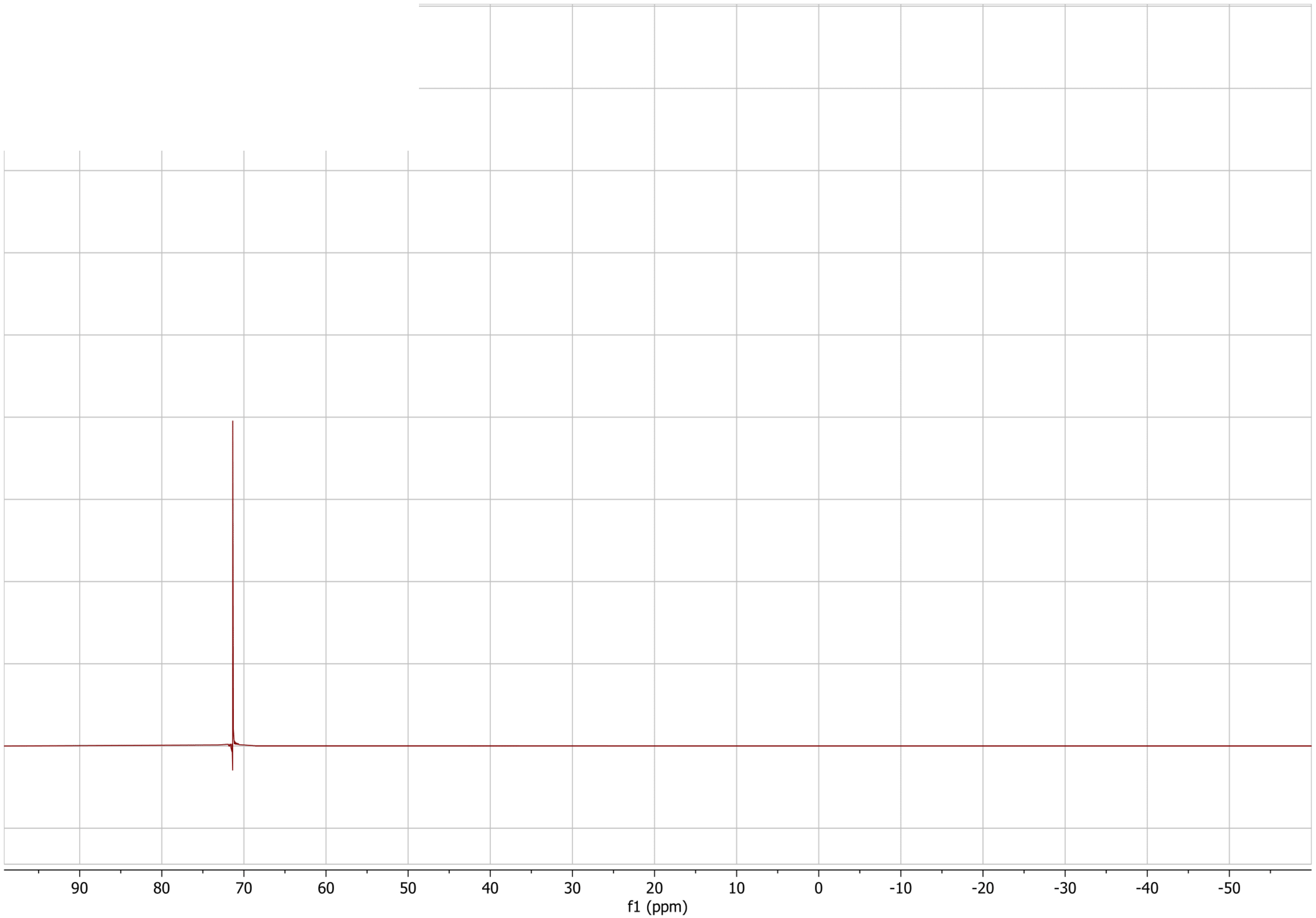

500 MHz ^{1}H spectrum in $CDCl_3$

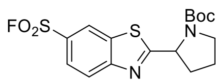

A-1

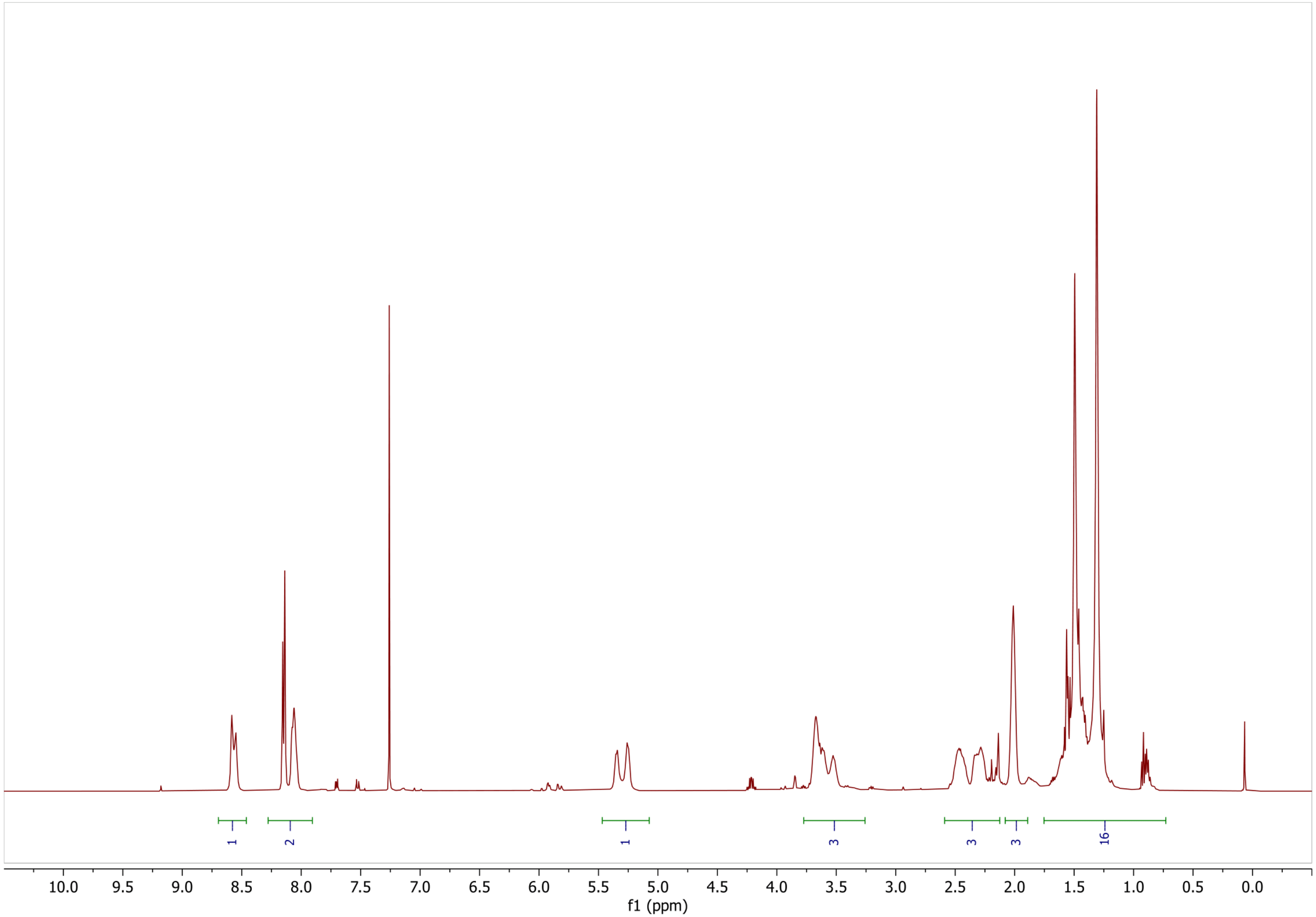

125 MHz ^{13}C spectrum in $CDCl_3$

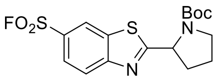

A-1

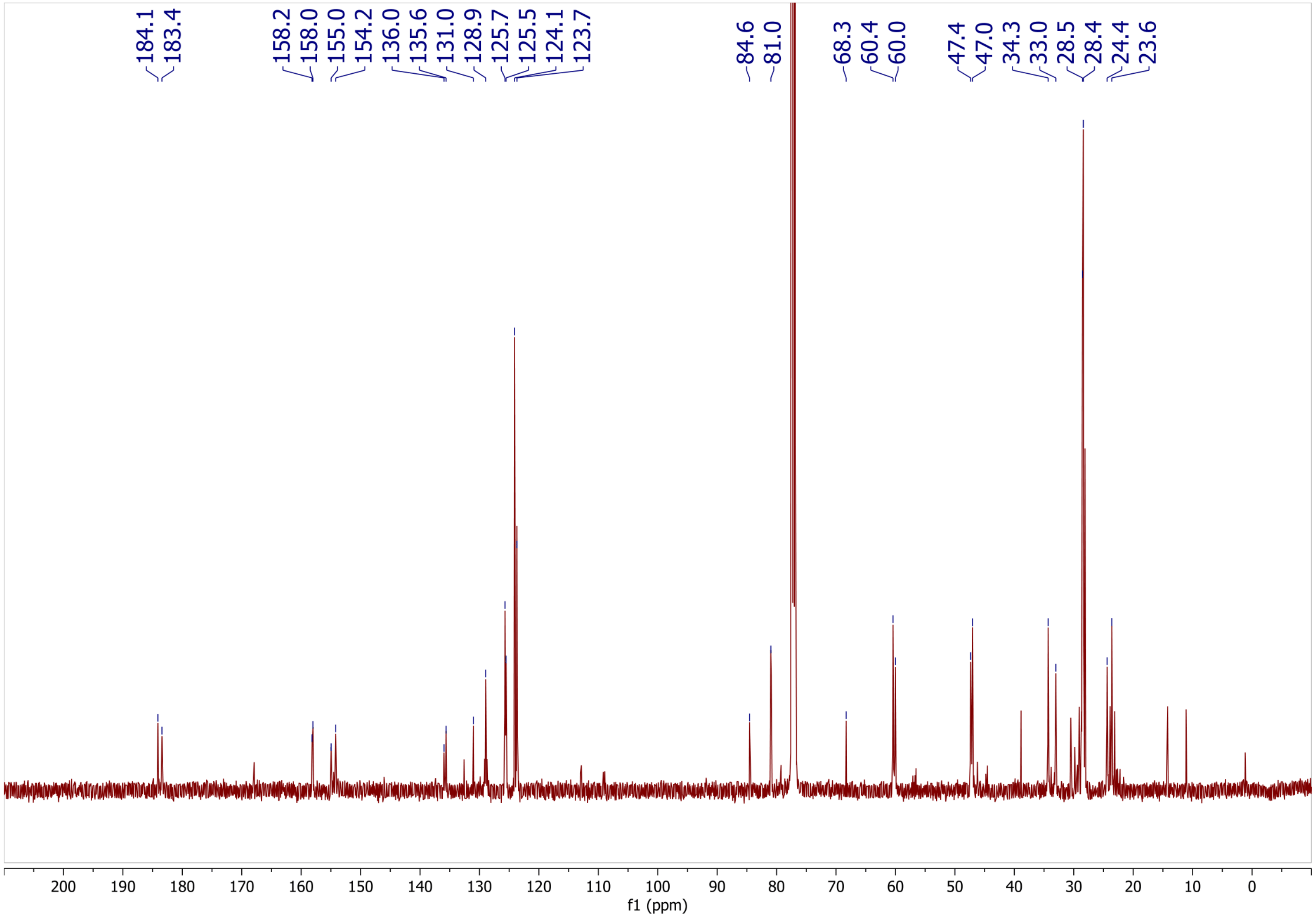

565 MHz ^{19}F spectrum in $CDCl_3$

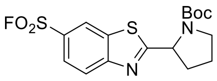

A-1

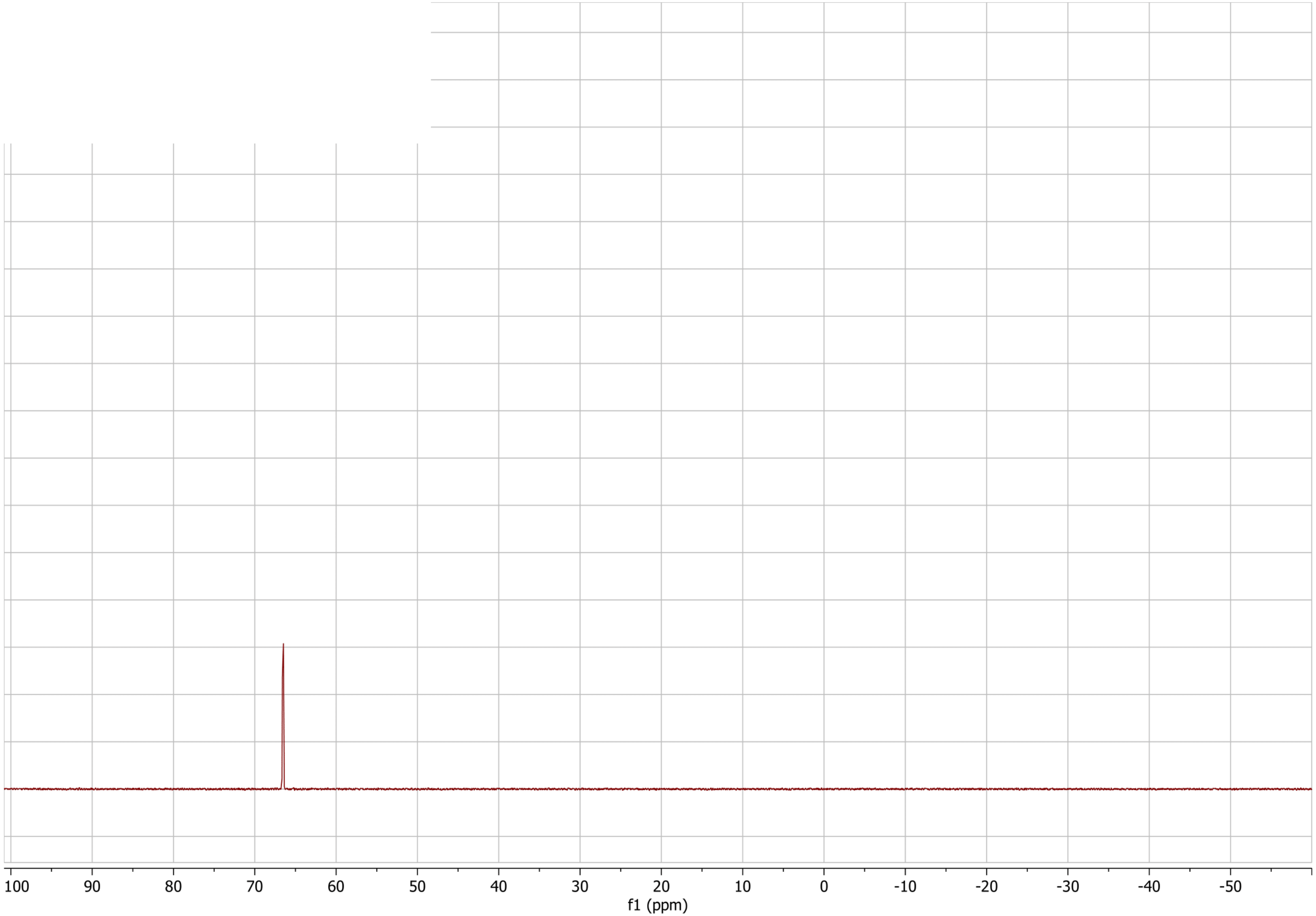

500 MHz ^{1}H spectrum in $CDCl_3$

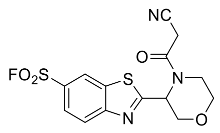

A-2a

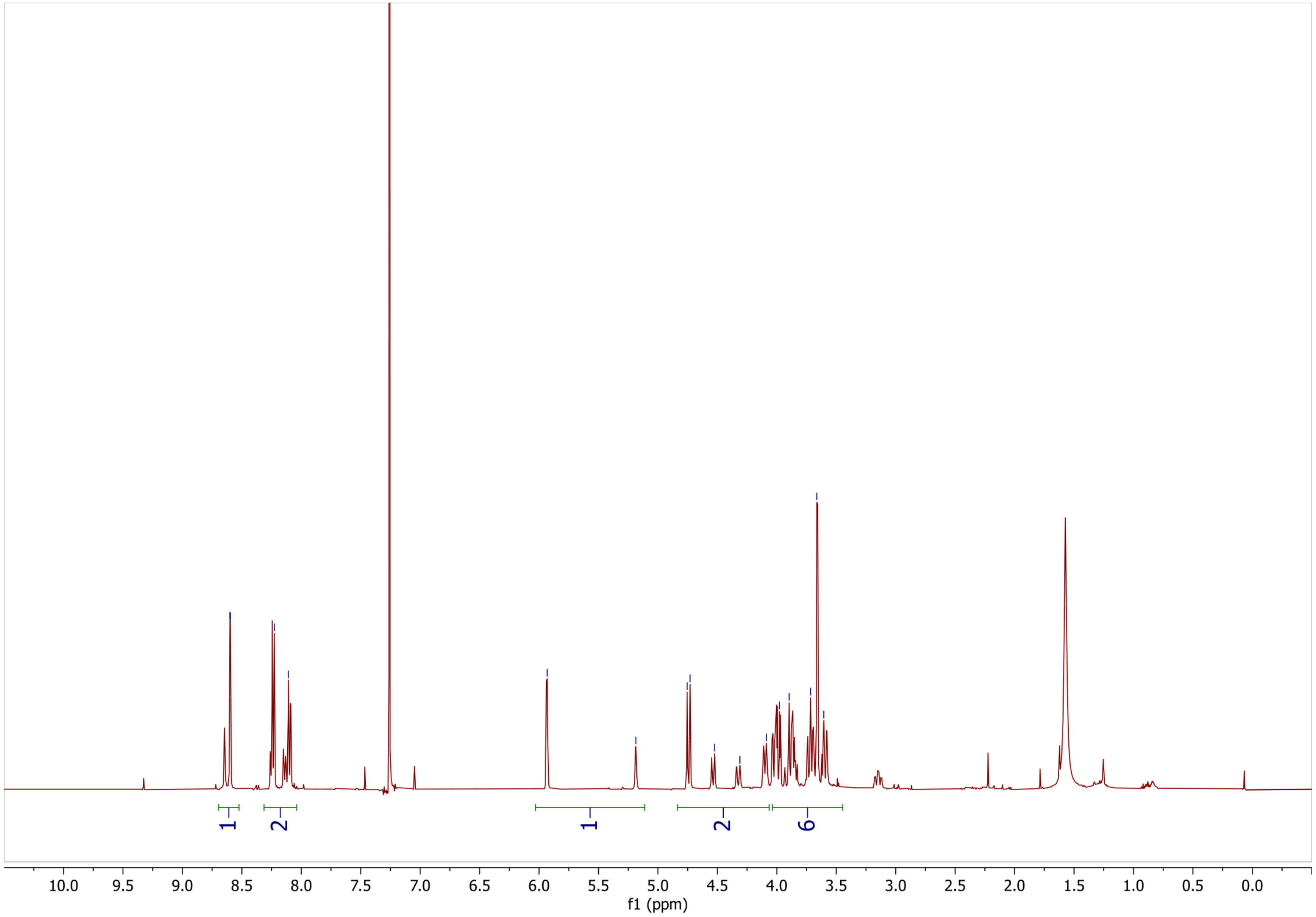

125 MHz ^{13}C spectrum in $CDCl_3$

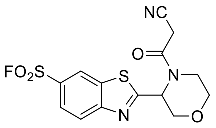

A-2a

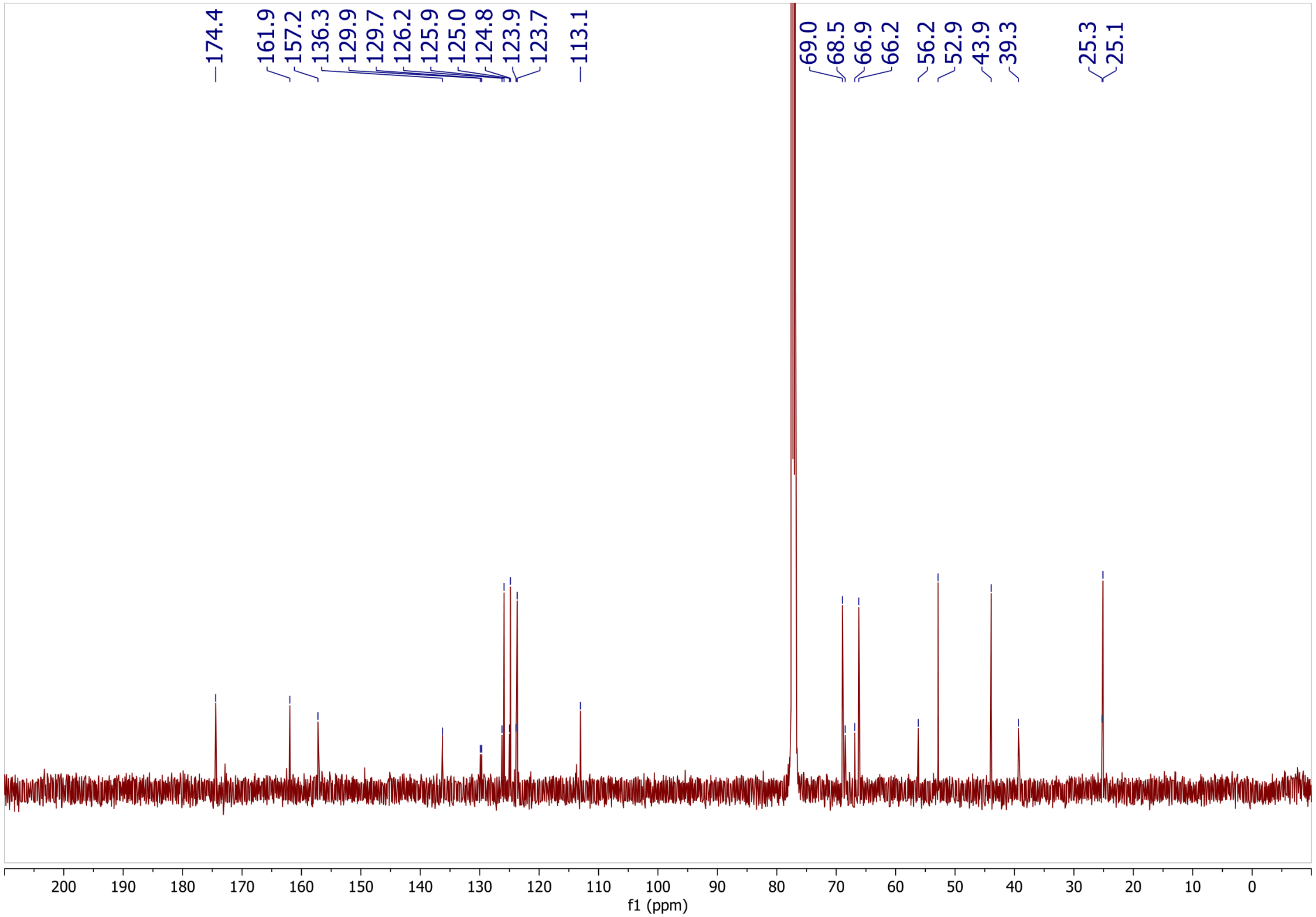

565 MHz ^{19}F spectrum in $CDCl_3$

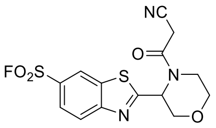

A-2a

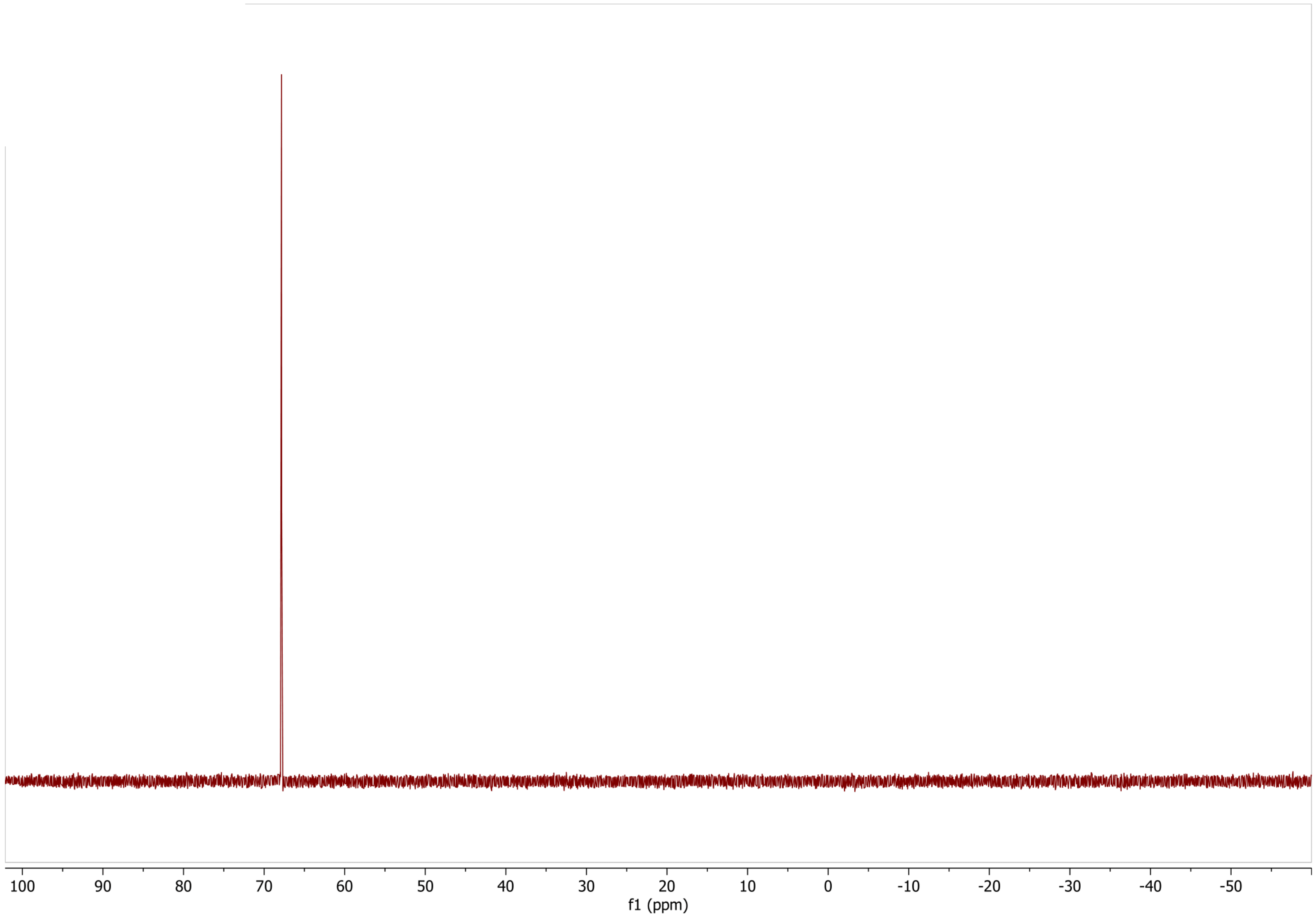

500 MHz ^{1}H spectrum in $CDCl_3$

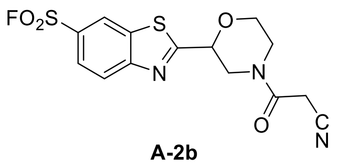

A-2b

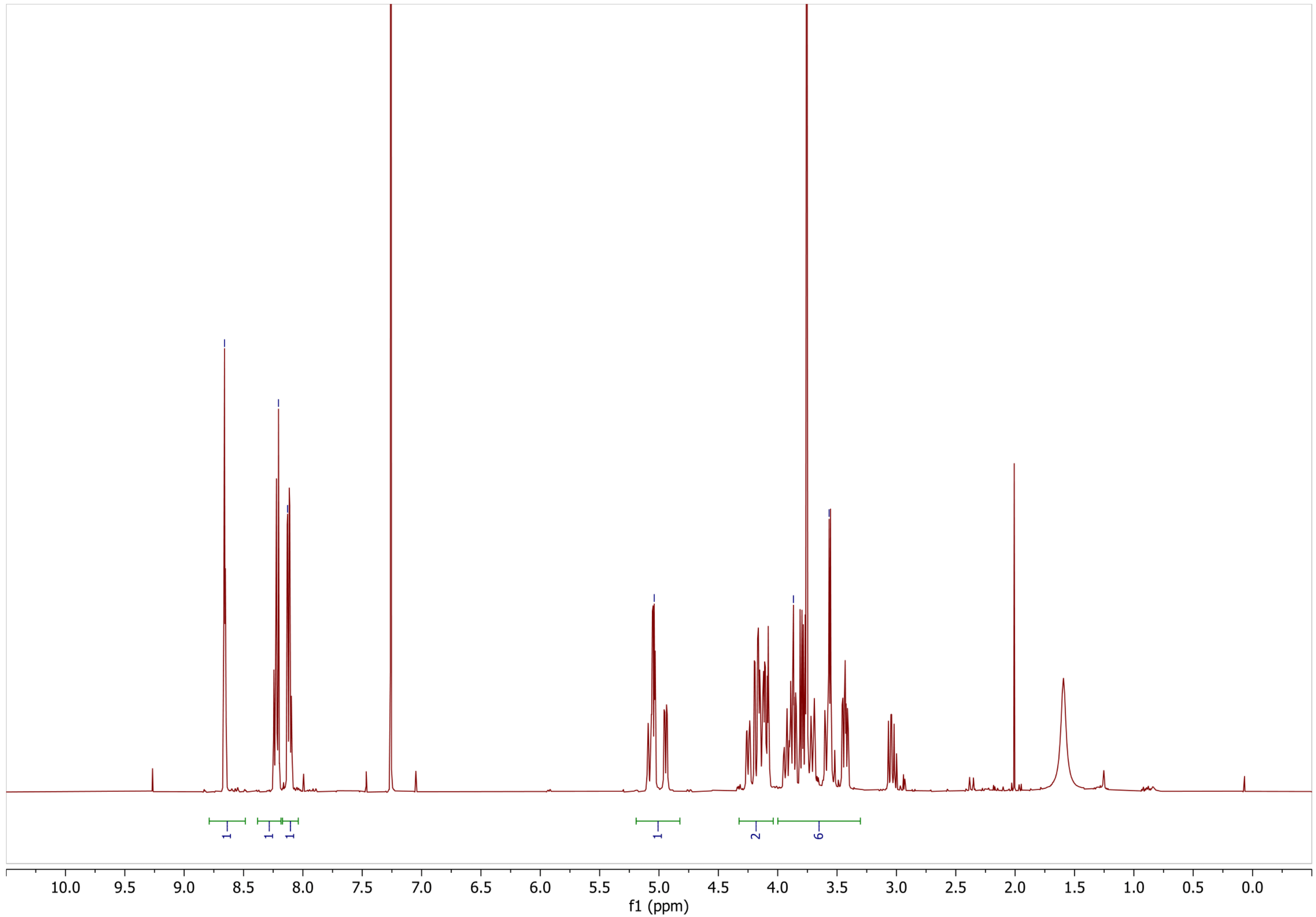

125 MHz ^{13}C spectrum in $CDCl_3$

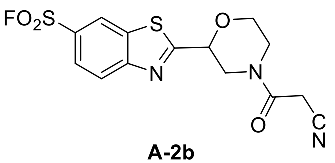

A-2b

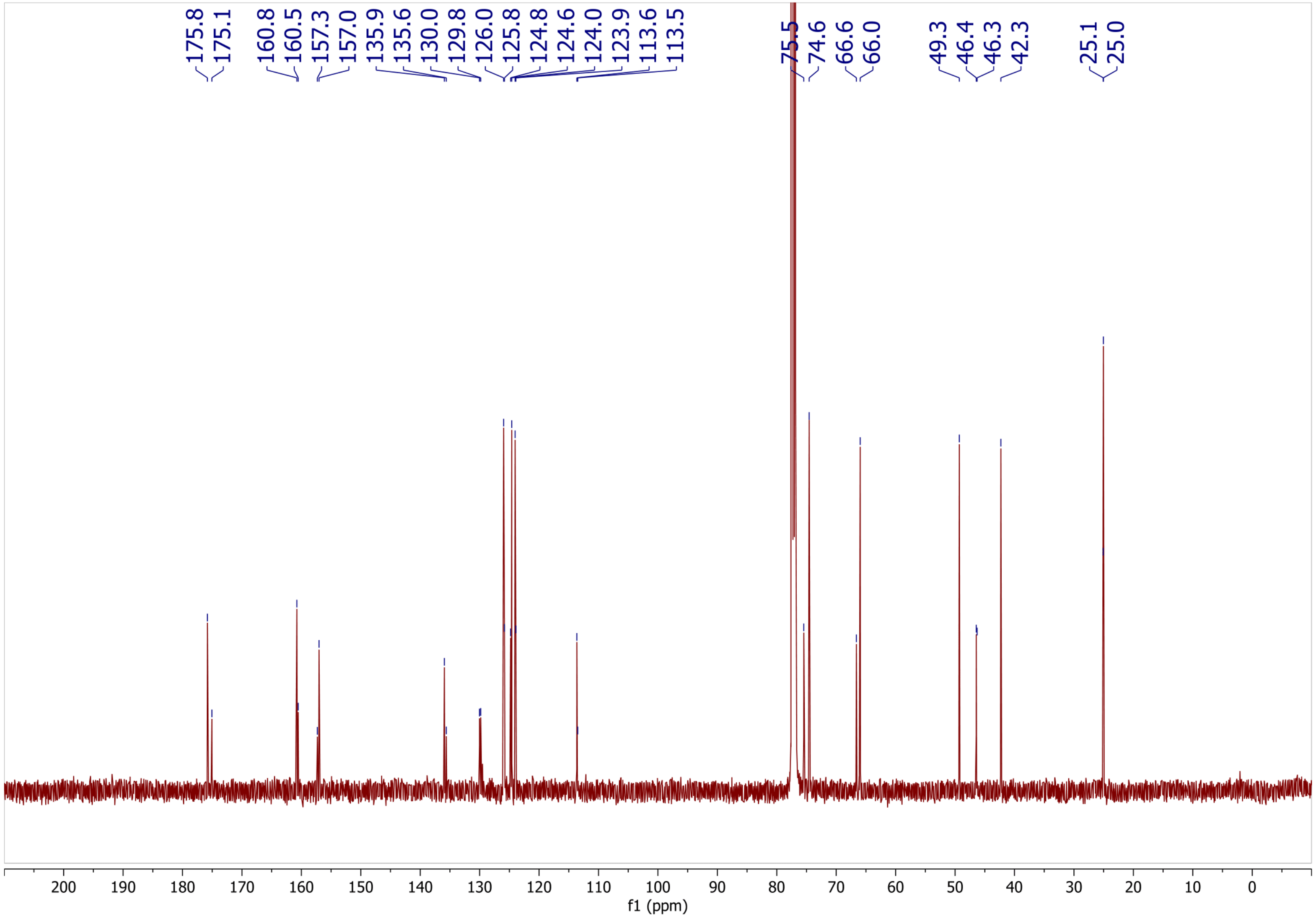

565 MHz ^{19}F spectrum in $CDCl_3$

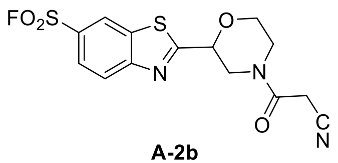

A-2b

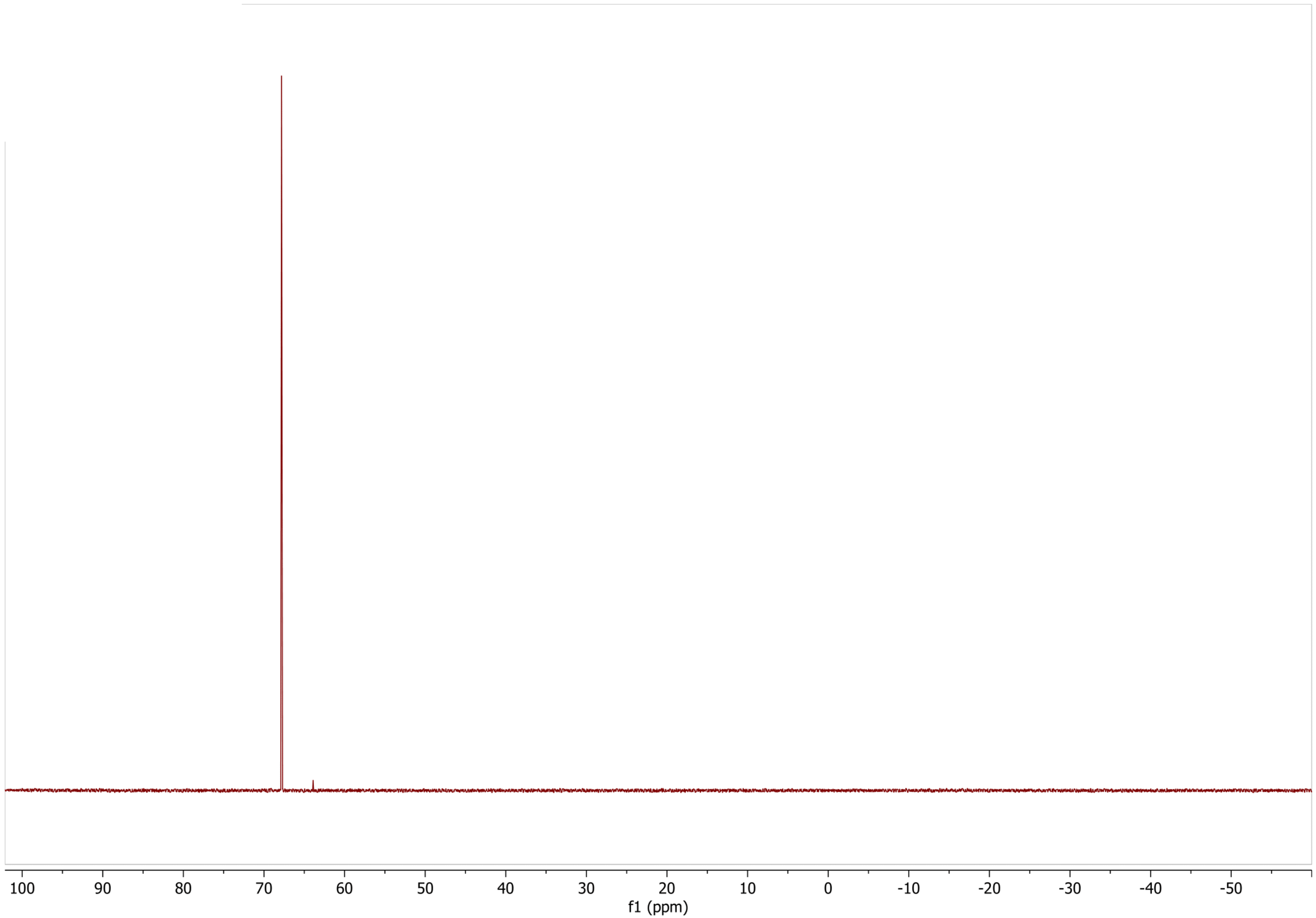

500 MHz ^1H spectrum in $CDCl_3$

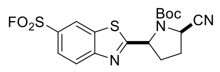

A-3a

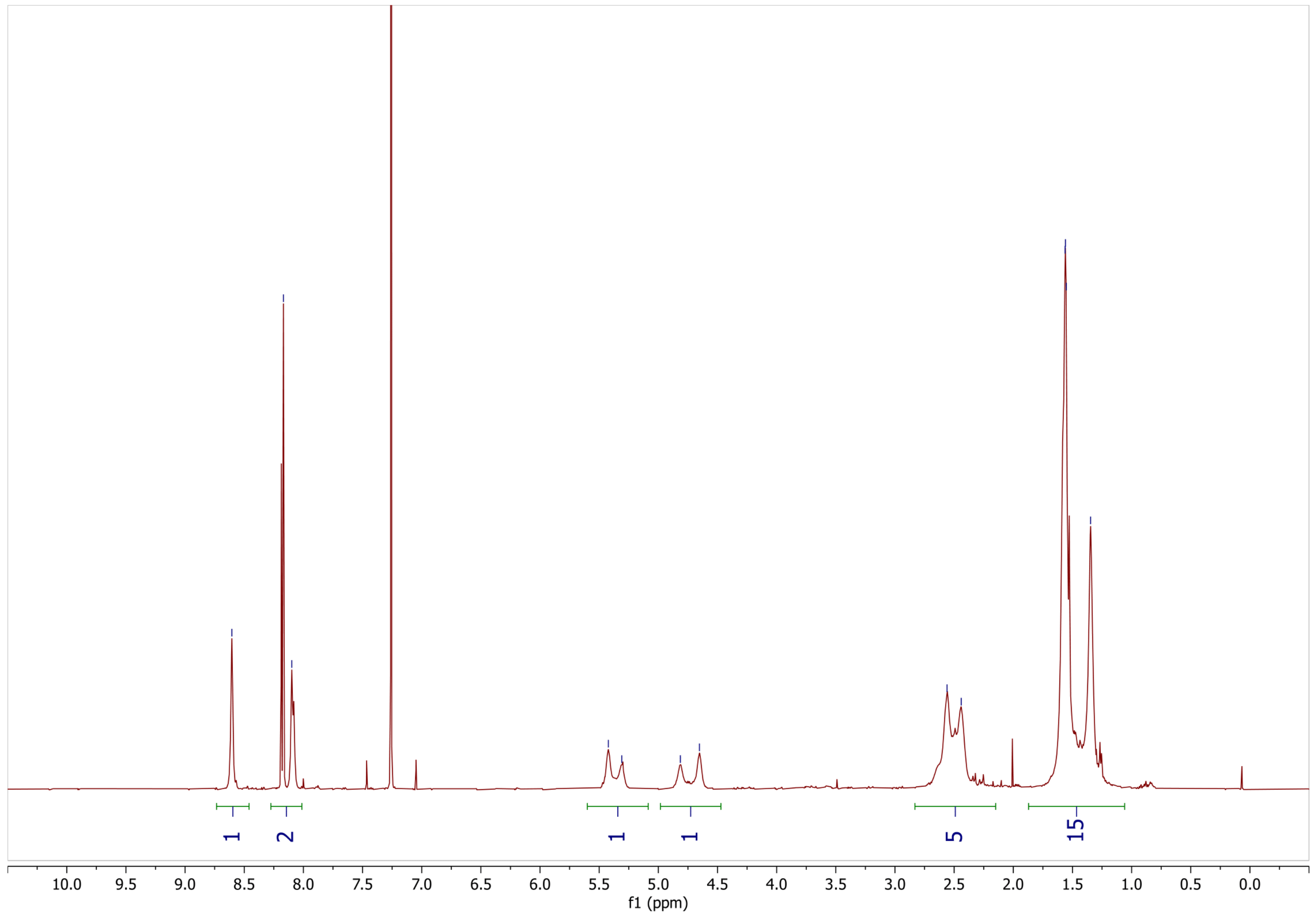

125 MHz ^{13}C spectrum in $CDCl_3$

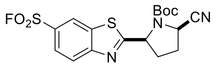

A-3a

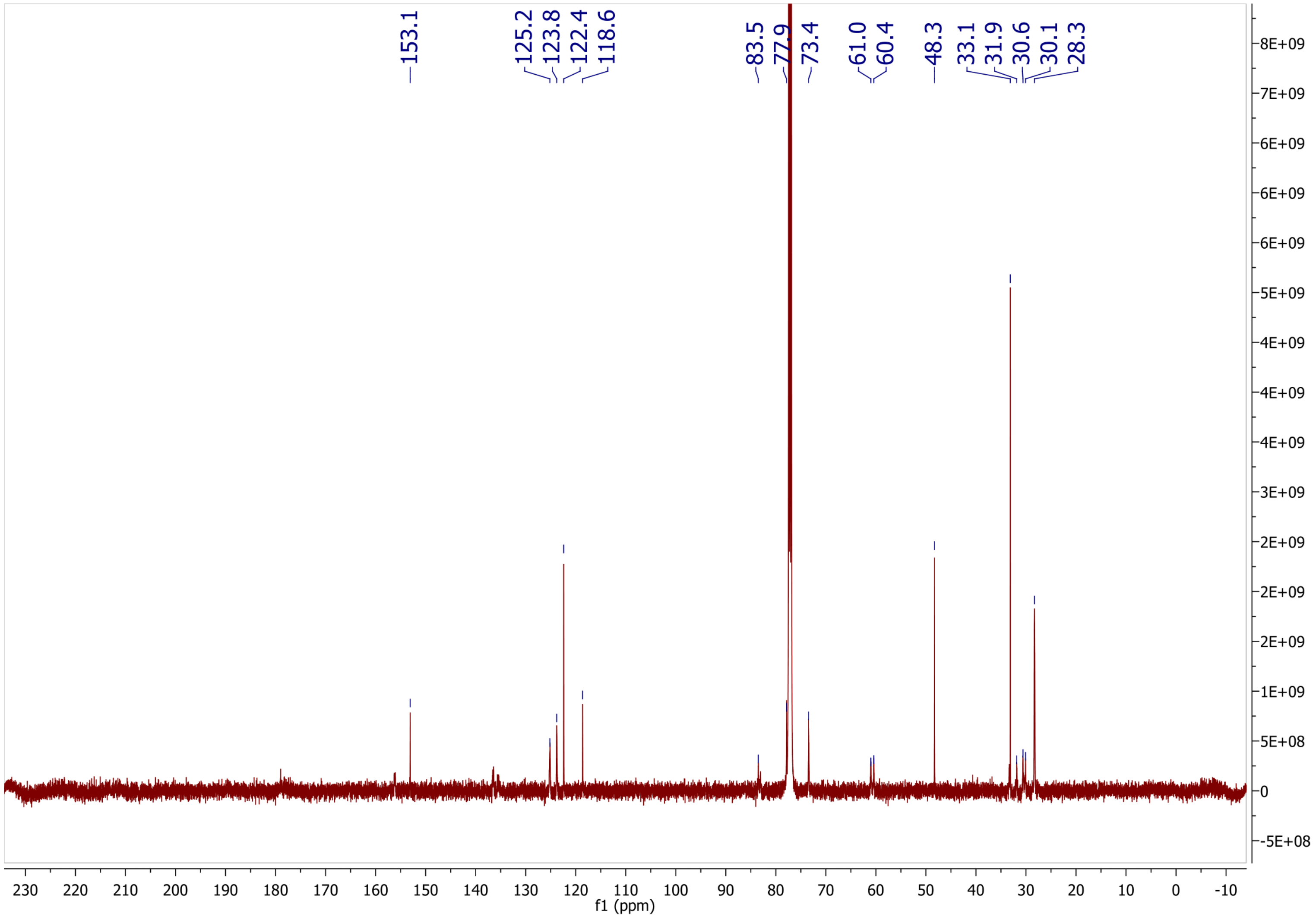

565 MHz ^{19}F spectrum in $CDCl_3$

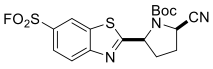

A-3a

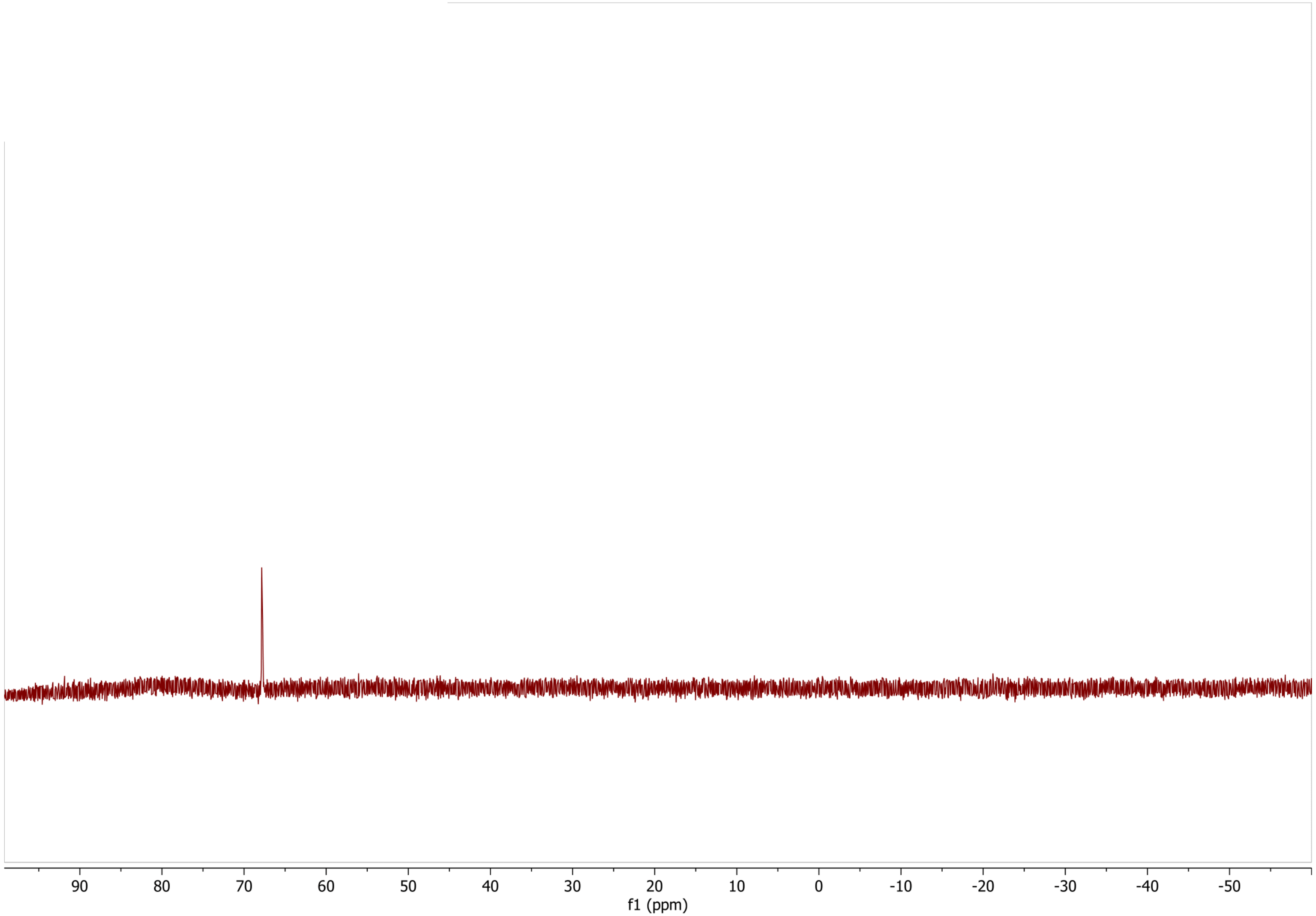

500 MHz ^{1}H spectrum in $CDCl_3$

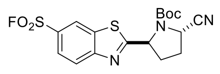

A-3b

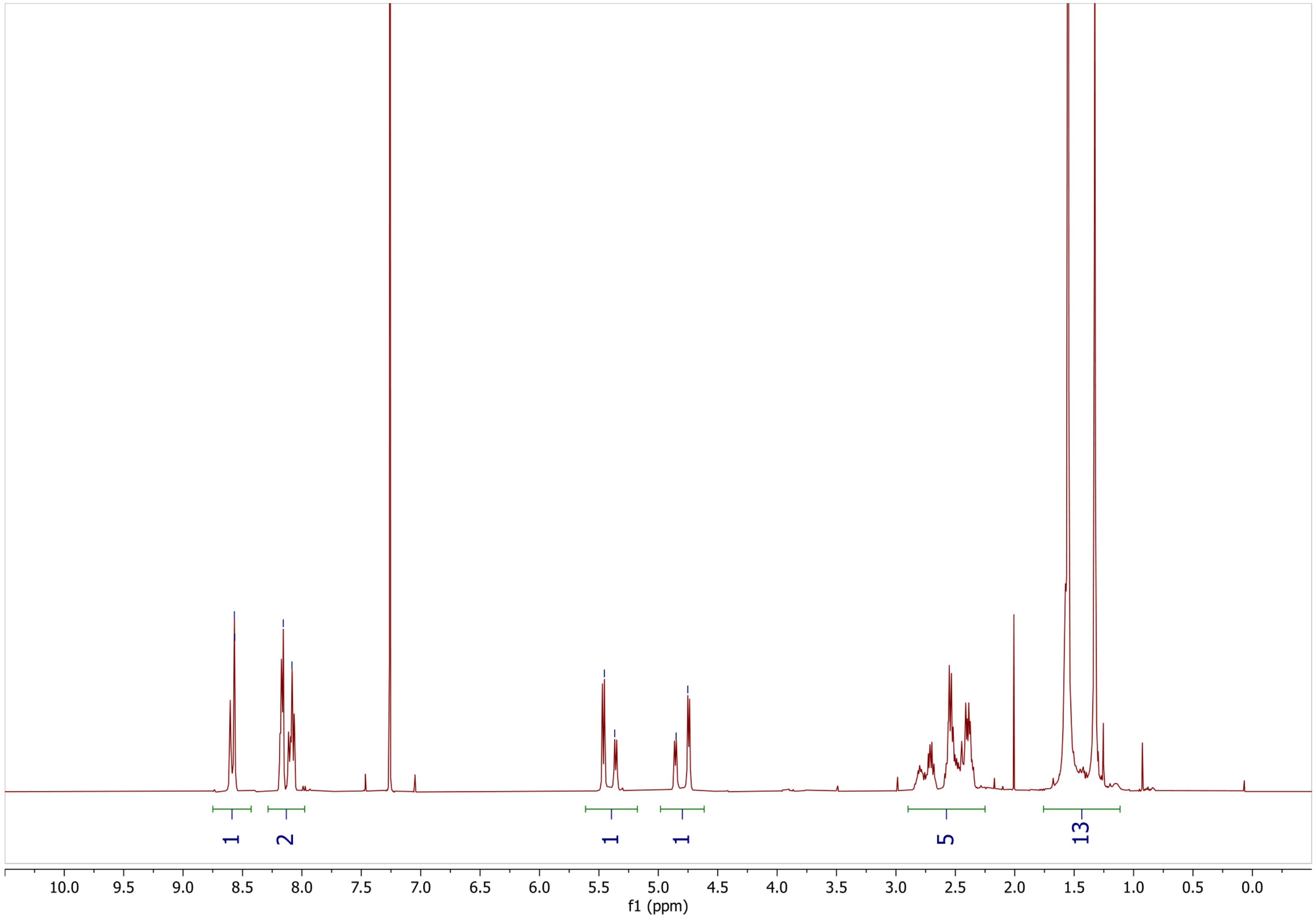

125 MHz ^{13}C spectrum in $CDCl_3$

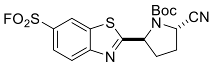

A-3b

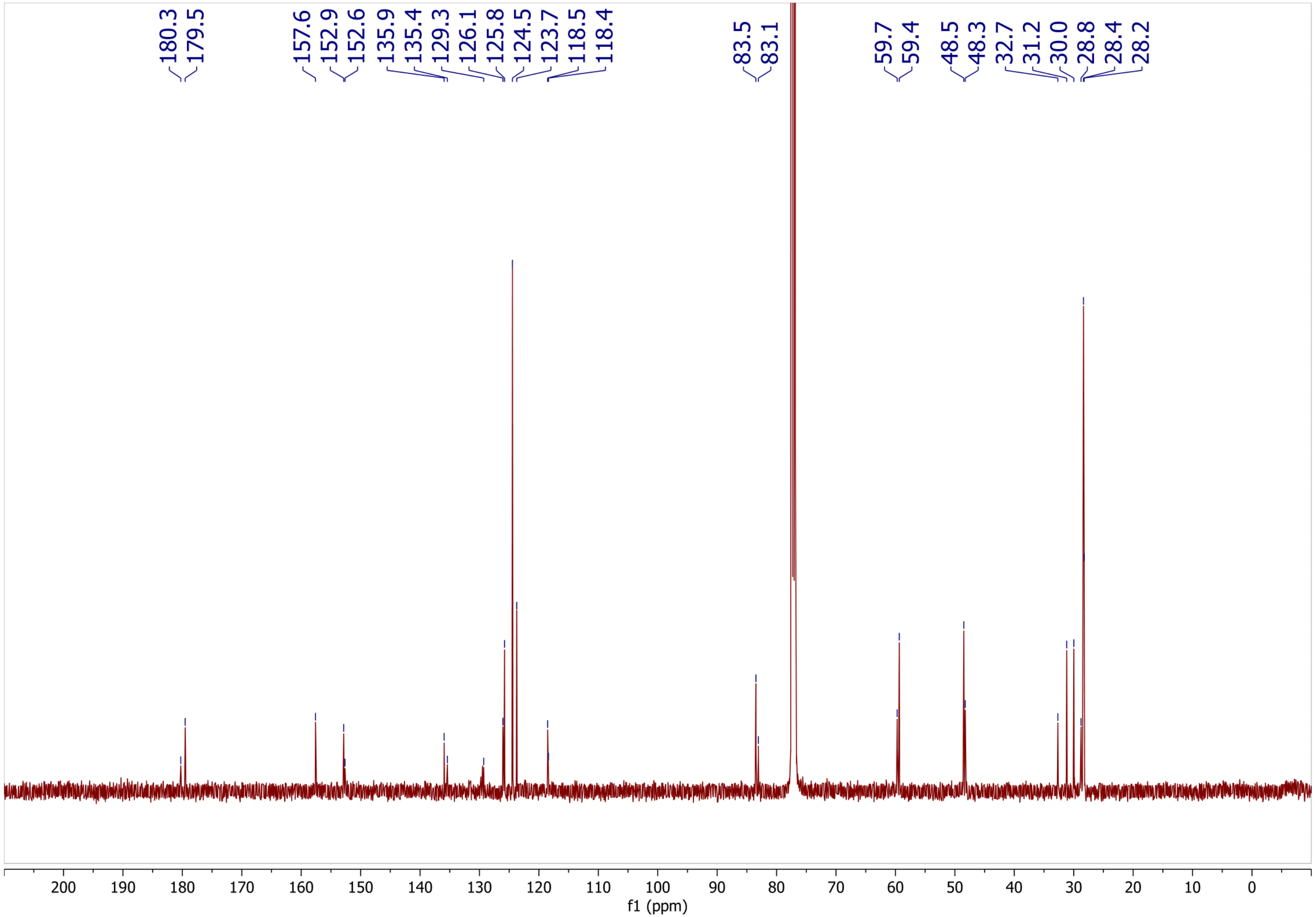

565 MHz ^{19}F spectrum in $CDCl_3$

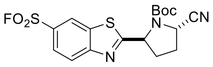

A-3b

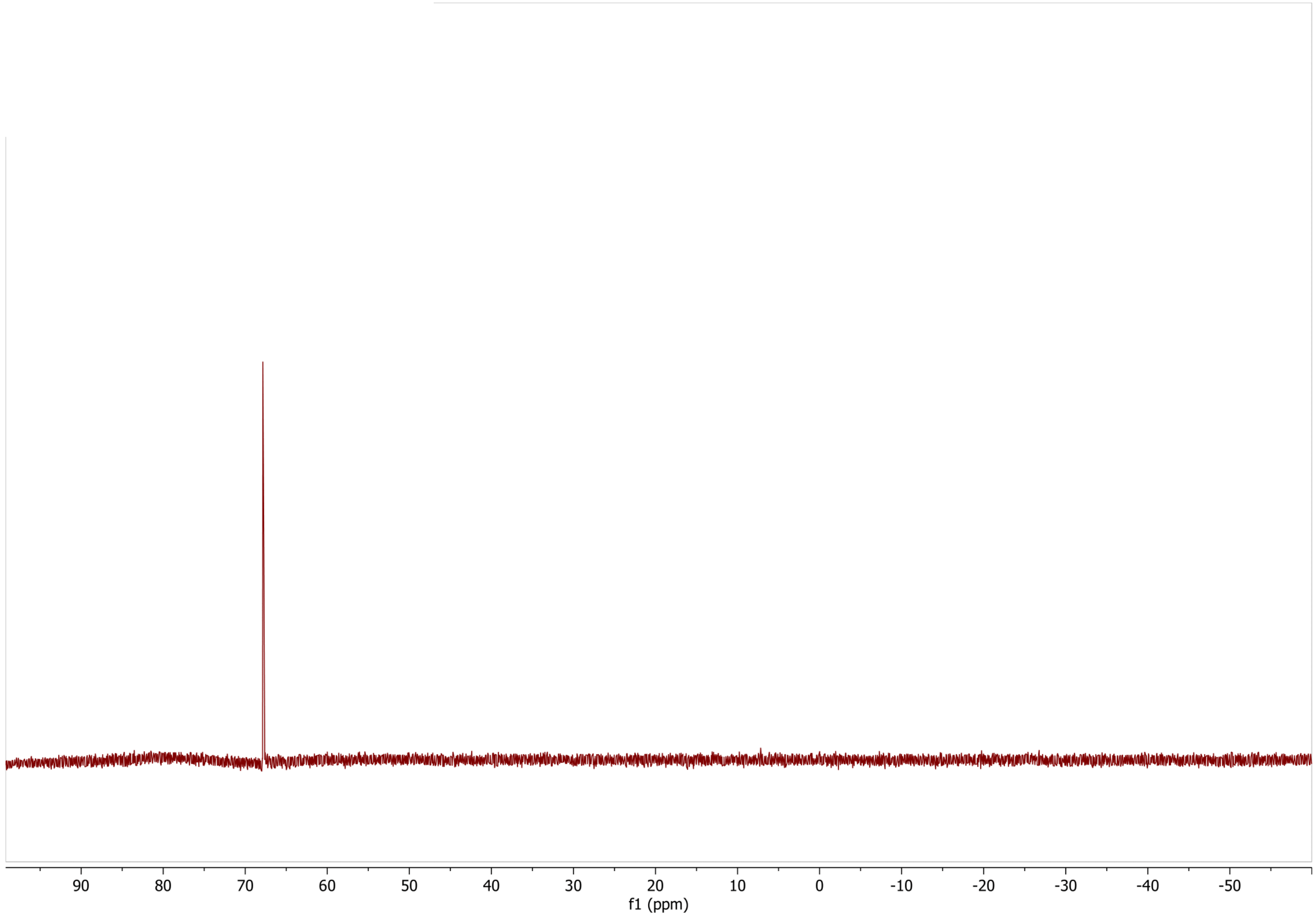

500 MHz ^{1}H spectrum in $CDCl_3$

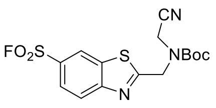

A-4

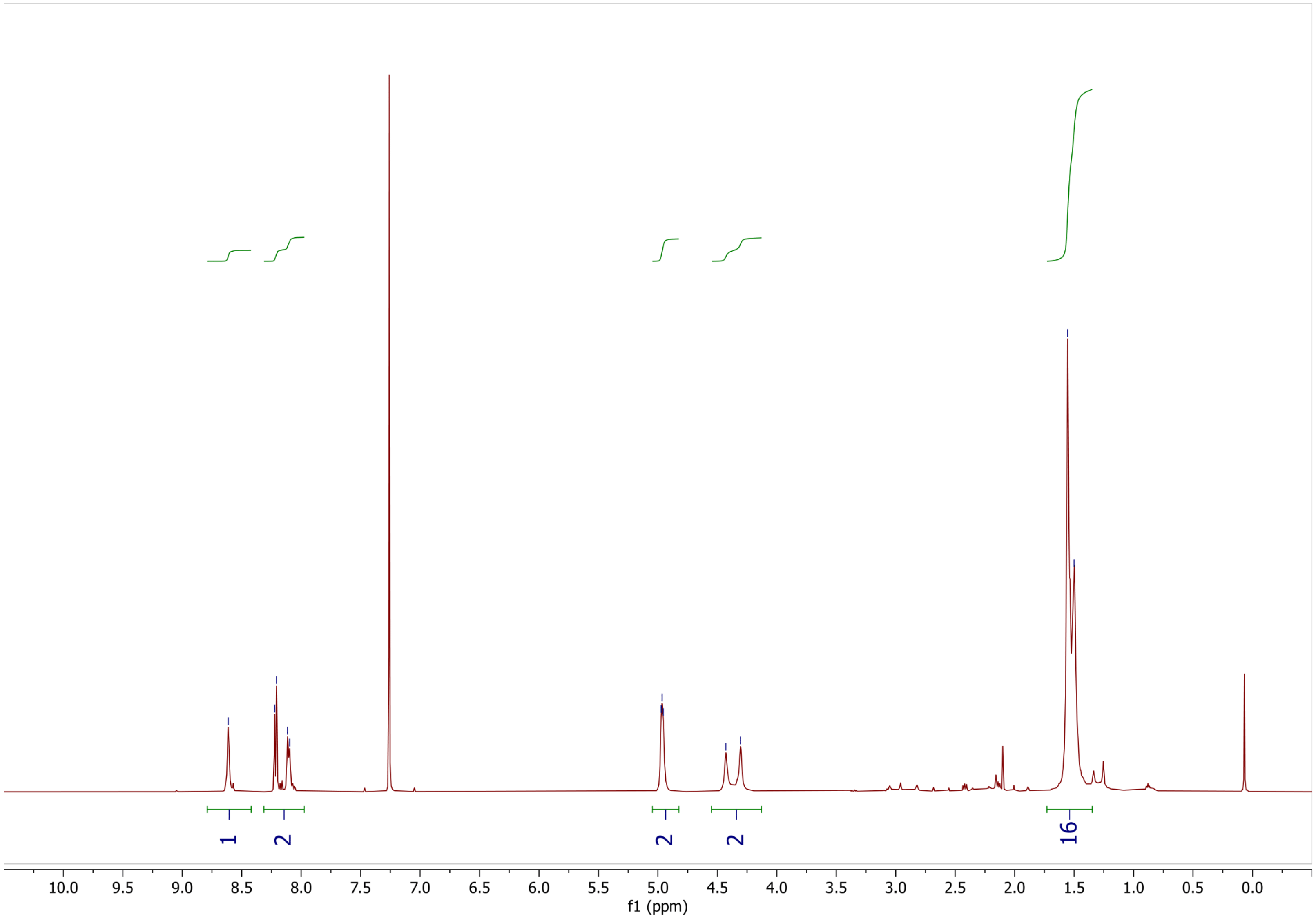

125 MHz ^{13}C spectrum in $CDCl_3$

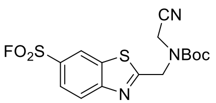

A-4

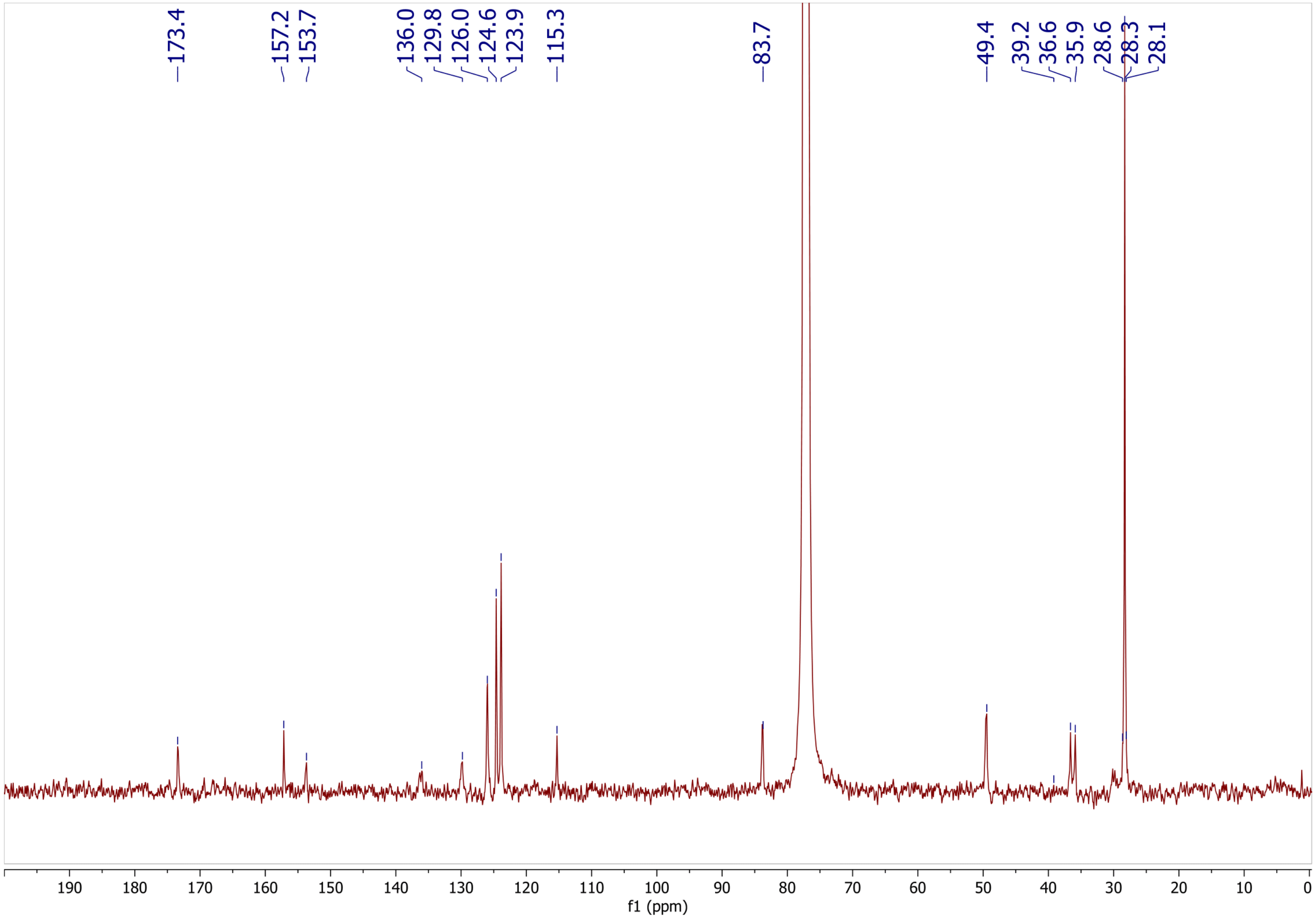

565 MHz ^{19}F spectrum in $CDCl_3$

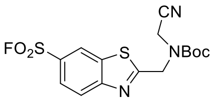

A-4

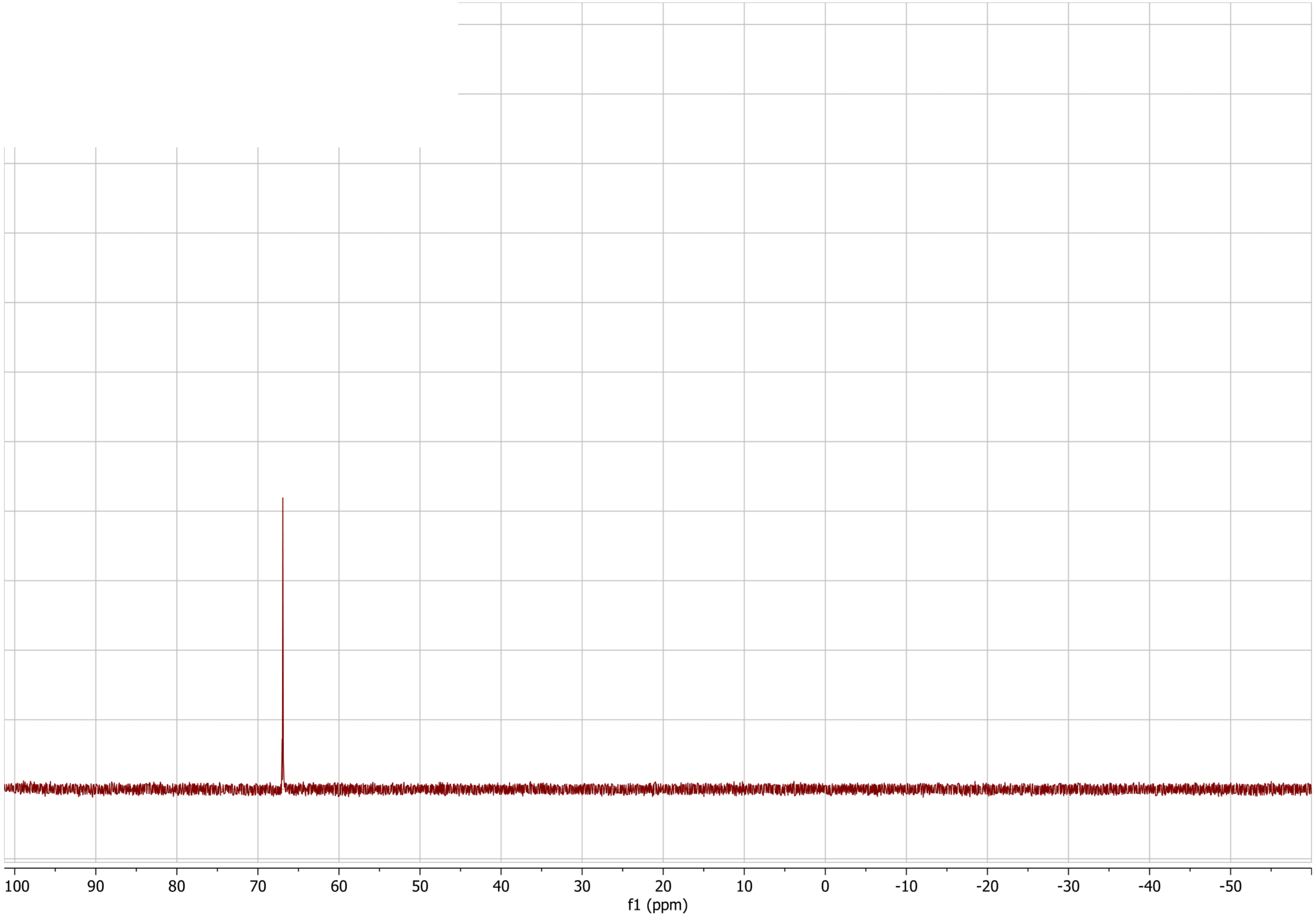

500 MHz ^{1}H spectrum in $CDCl_3$

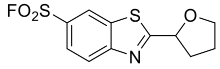

A-6

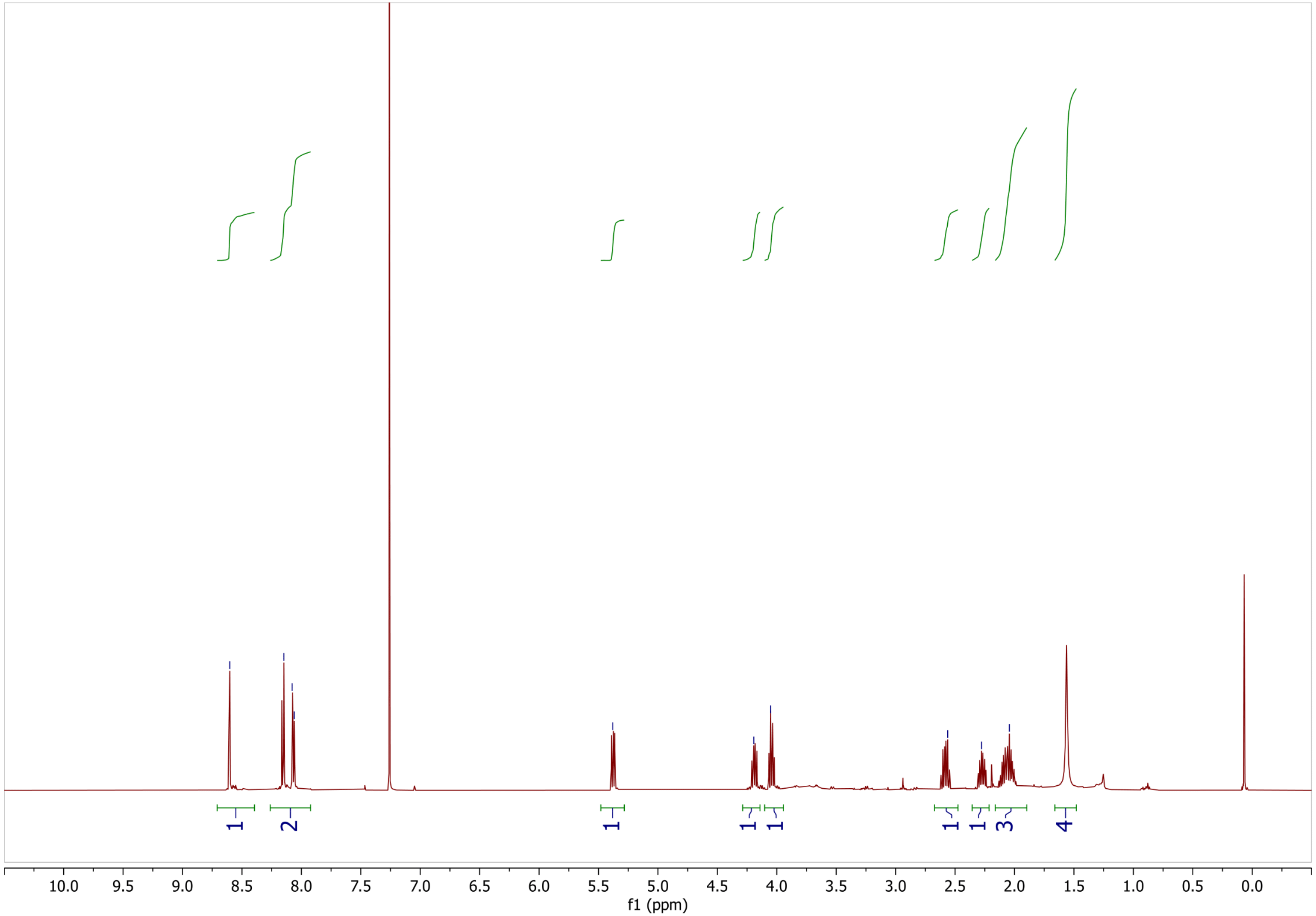

125 MHz ^{13}C spectrum in $CDCl_3$

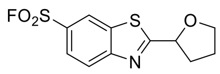

A-6

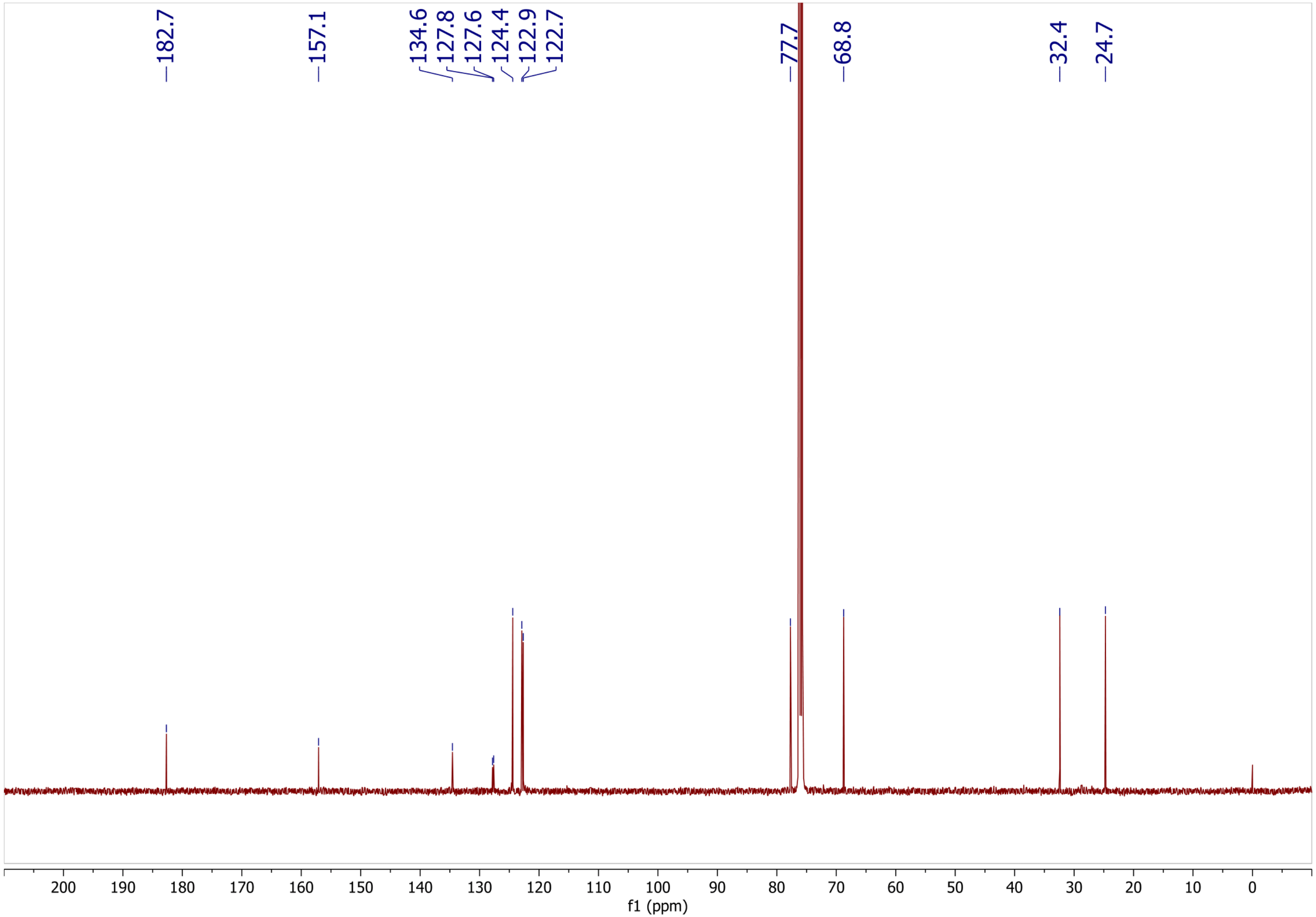

565 MHz ^{19}F spectrum in $CDCl_3$

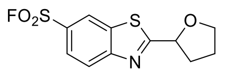

A-6

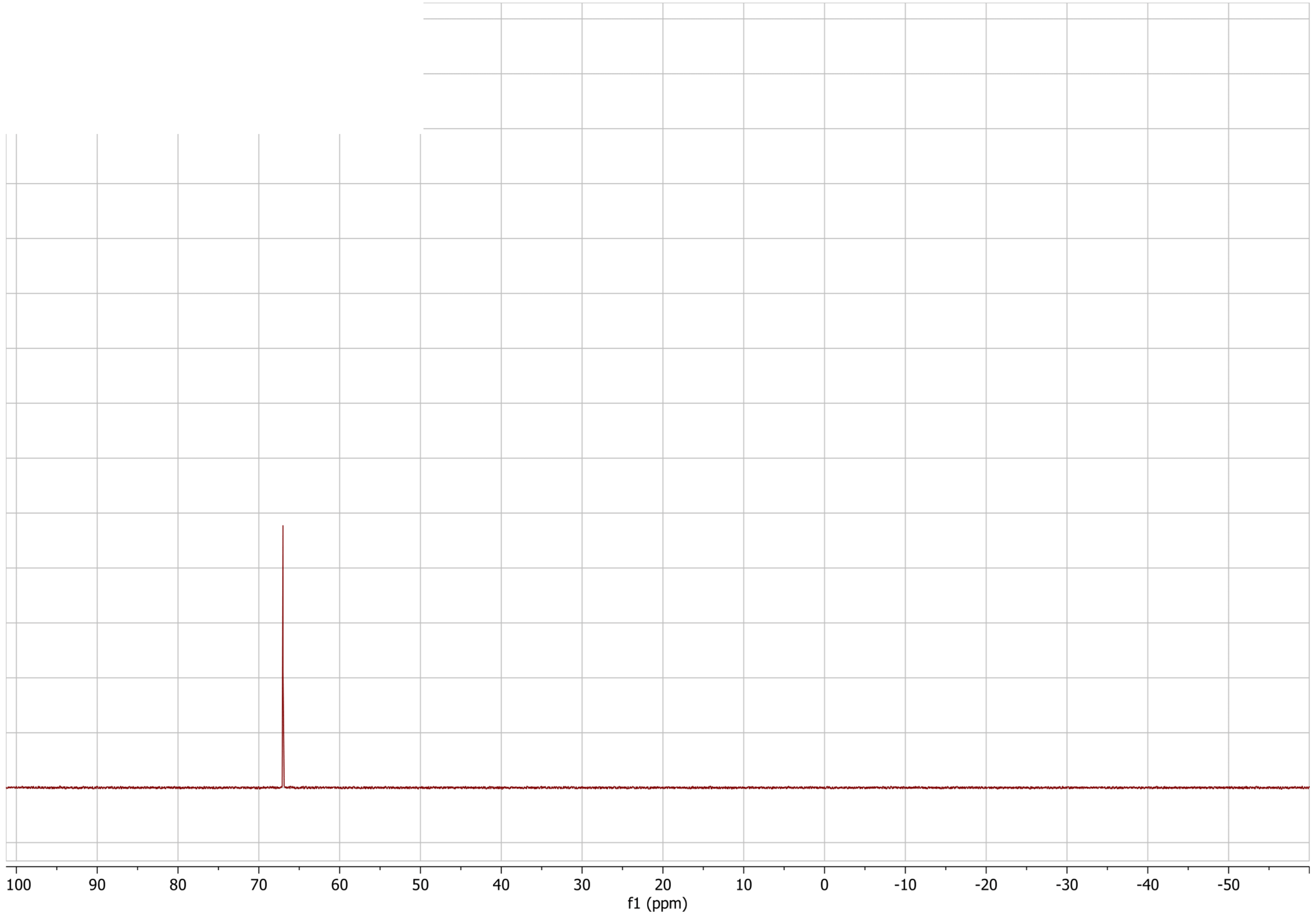

500 MHz ^{1}H spectrum in $CDCl_3$

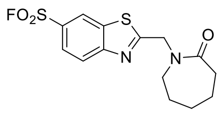

A-8

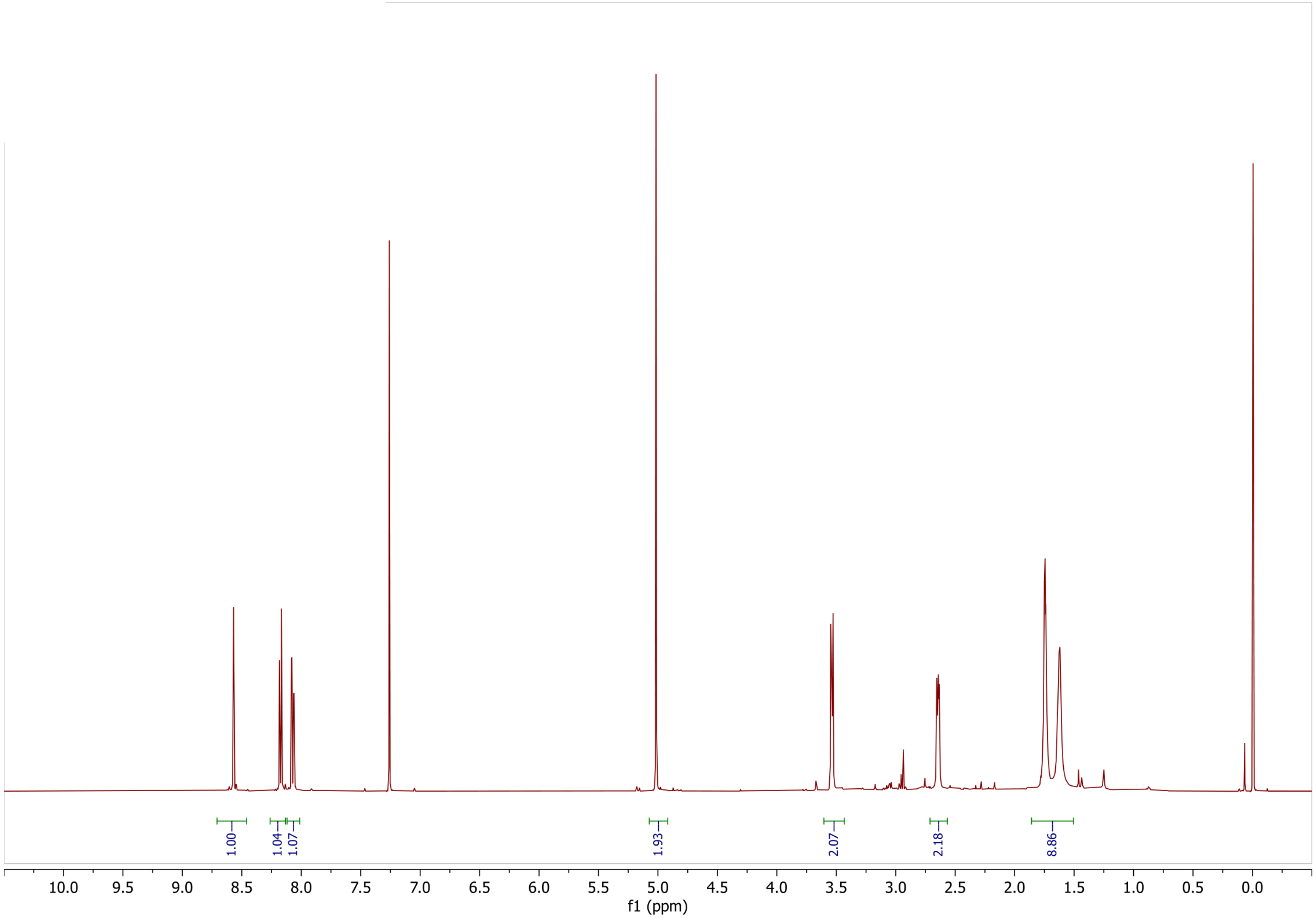

125 MHz ^{13}C spectrum in $CDCl_3$

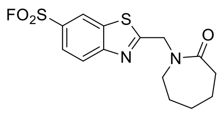

A-8

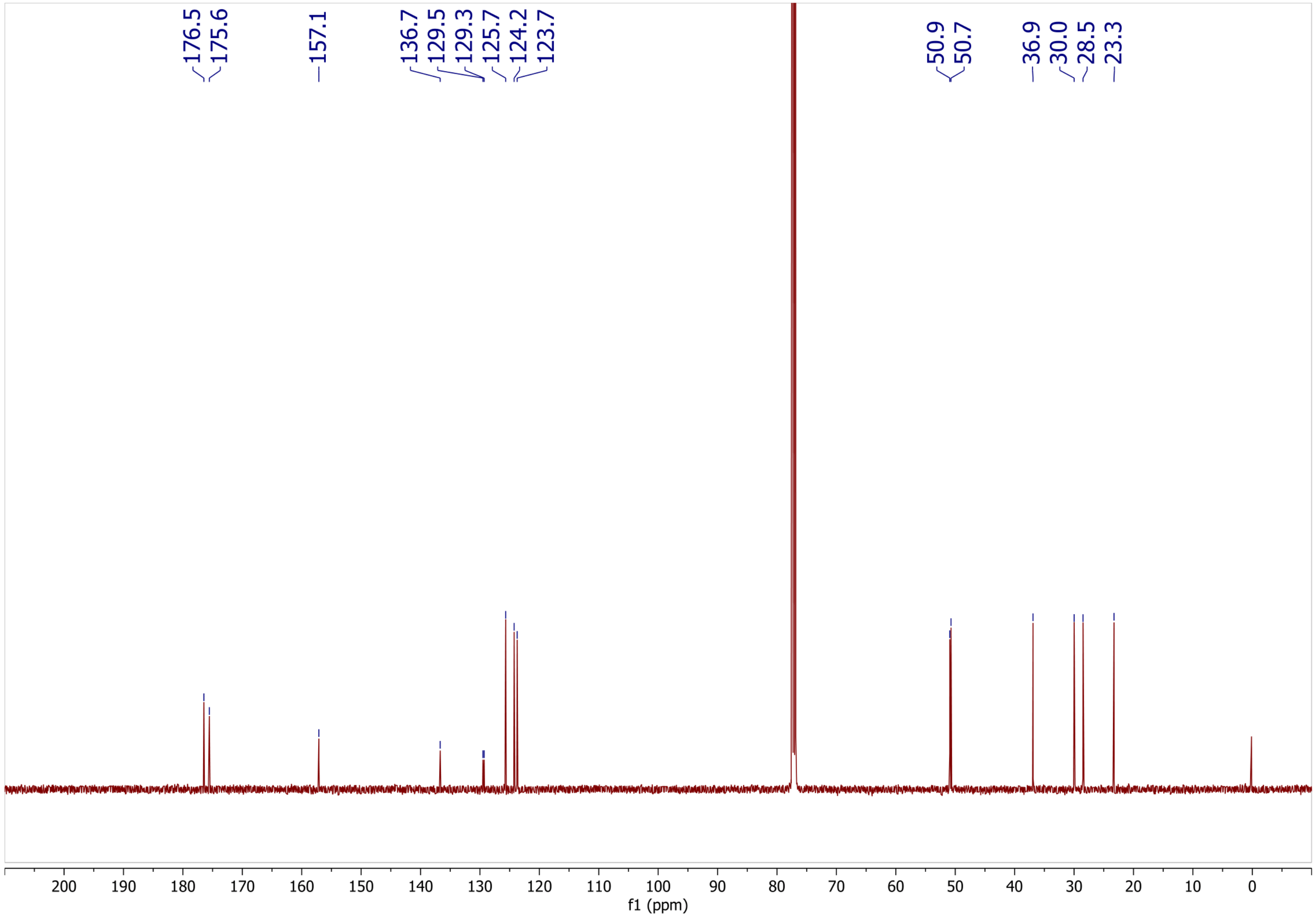

565 MHz ^{19}F spectrum in $CDCl_3$

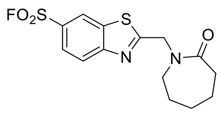

A-8

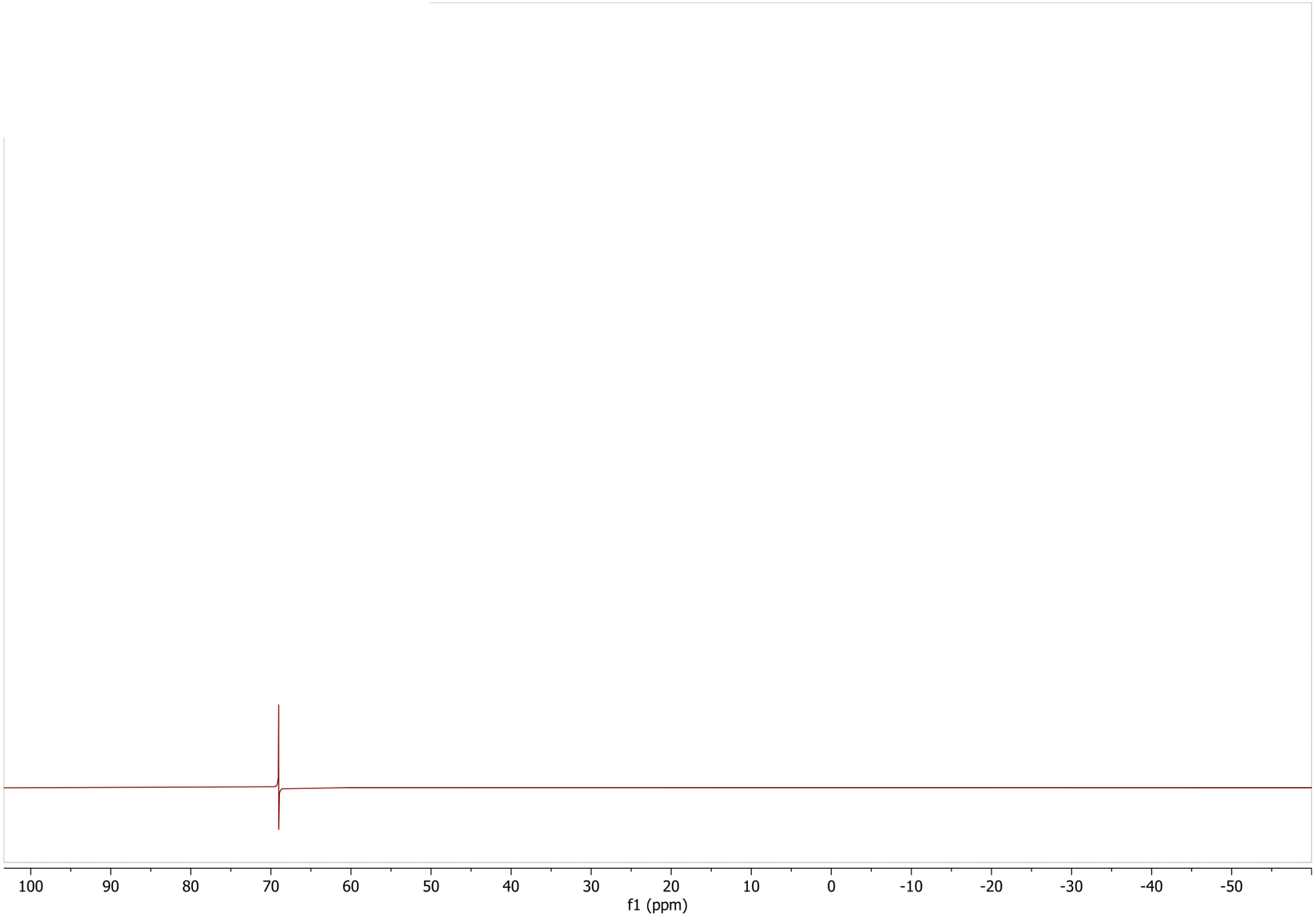

500 MHz ^{1}H spectrum in $CDCl_3$

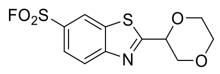

A-10

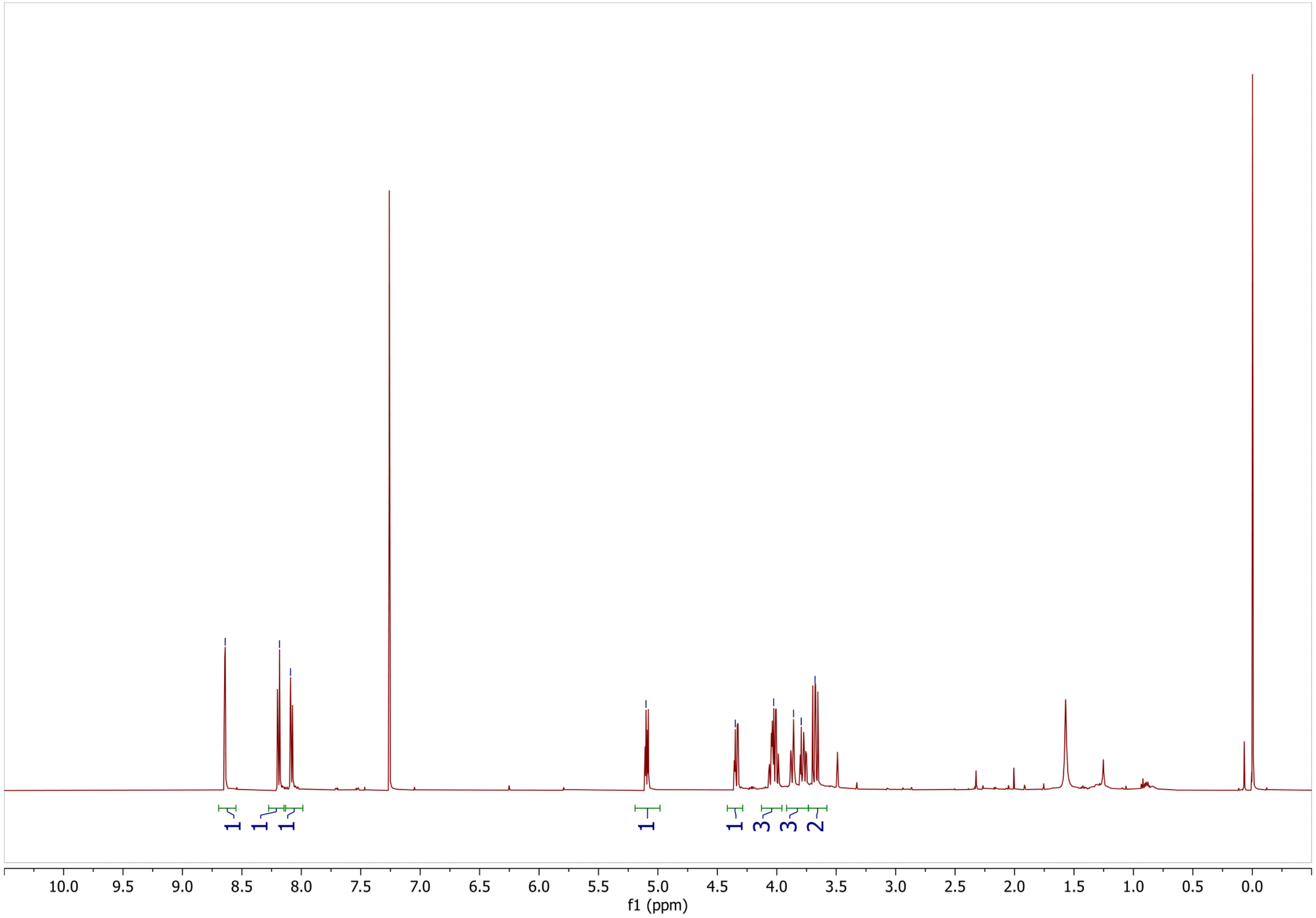

125 MHz ^{13}C spectrum in $CDCl_3$

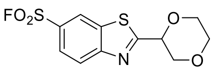

A-10

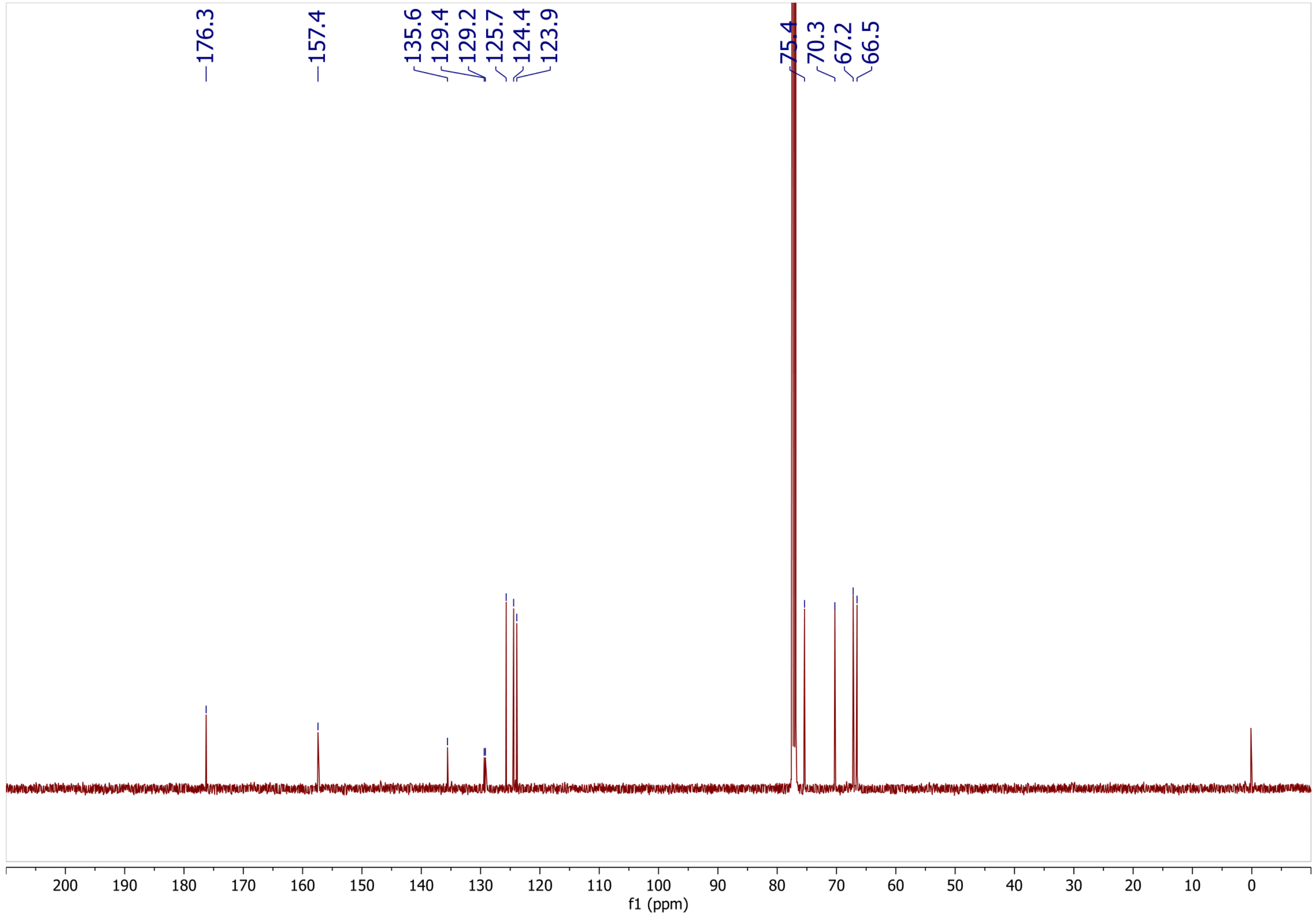

565 MHz ^{19}F spectrum in $CDCl_3$

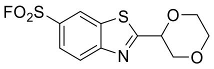

A-10

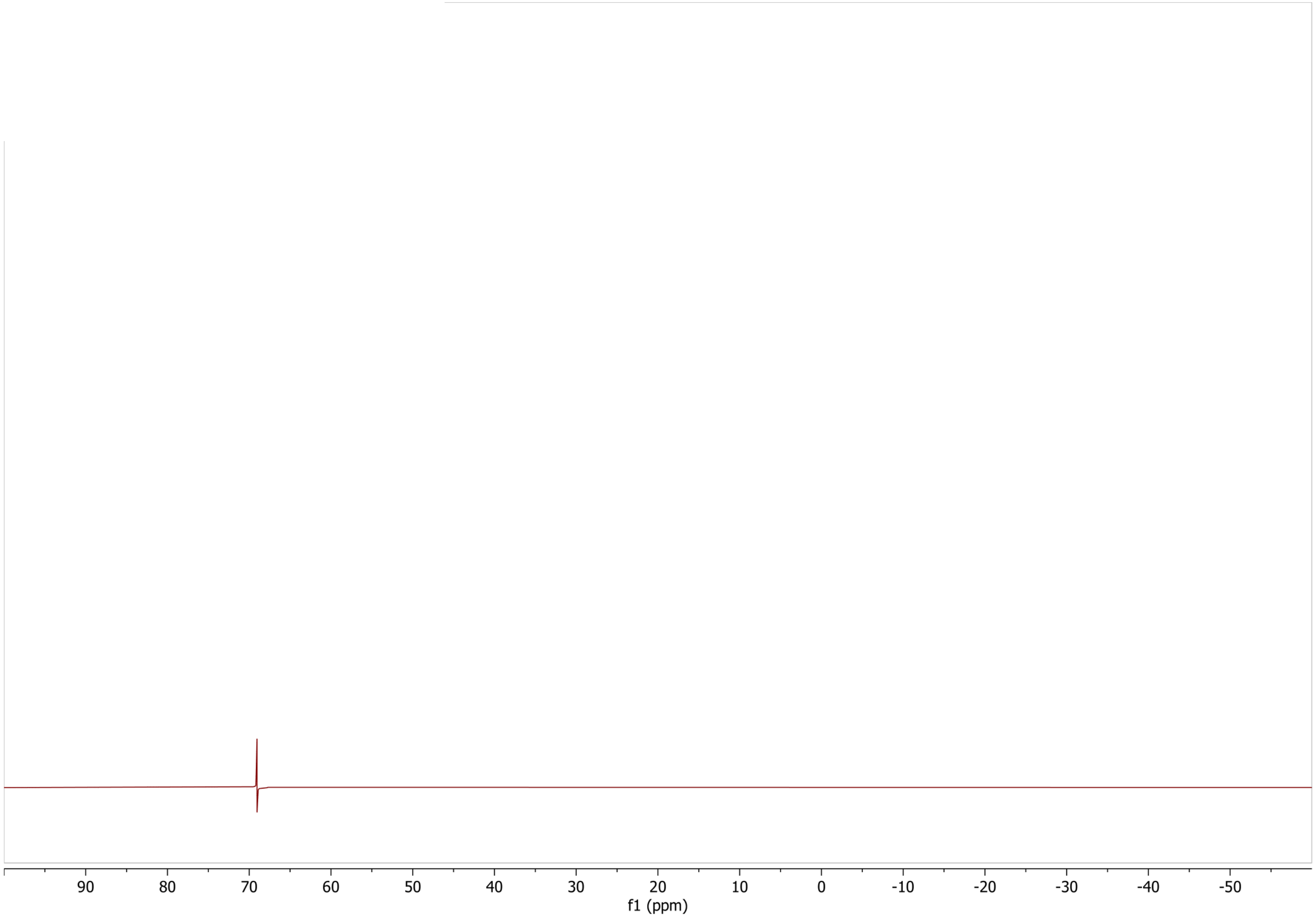

500 MHz ^{1}H spectrum in $CDCl_3$

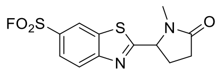

A-12

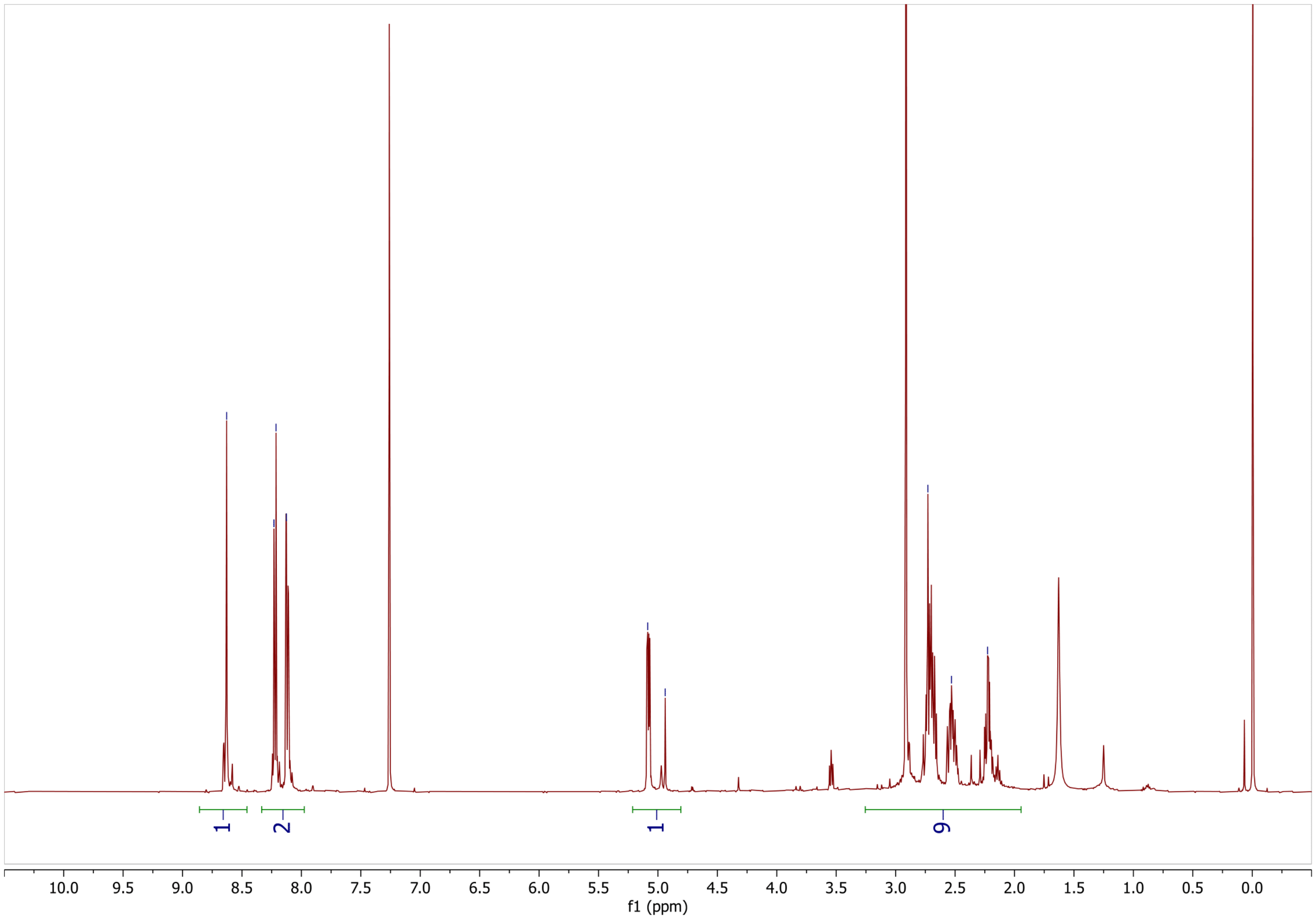

125 MHz ^{13}C spectrum in $CDCl_3$

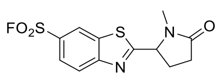

A-12

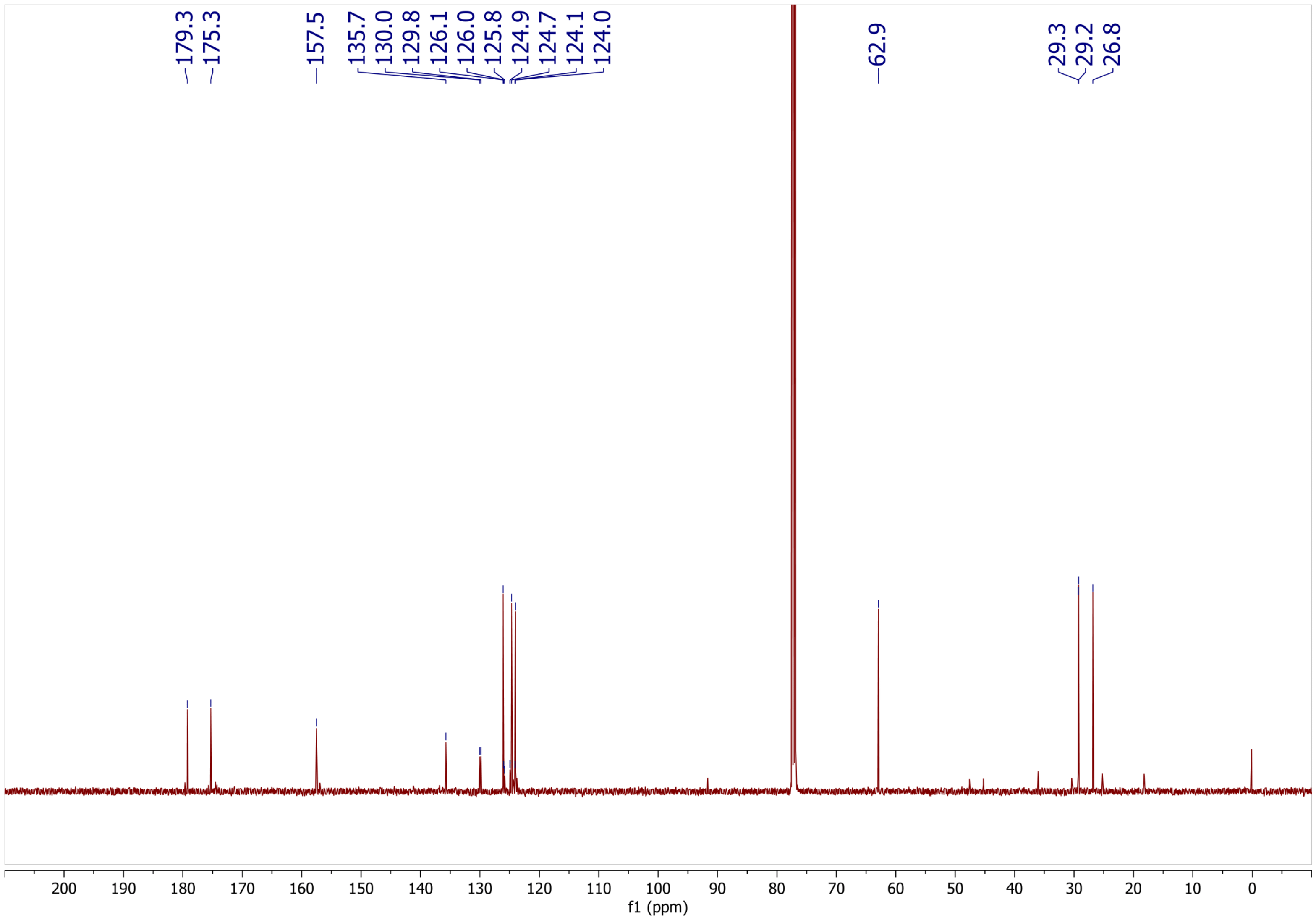

565 MHz ^{19}F spectrum in $CDCl_3$

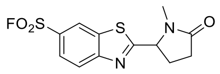

A-12

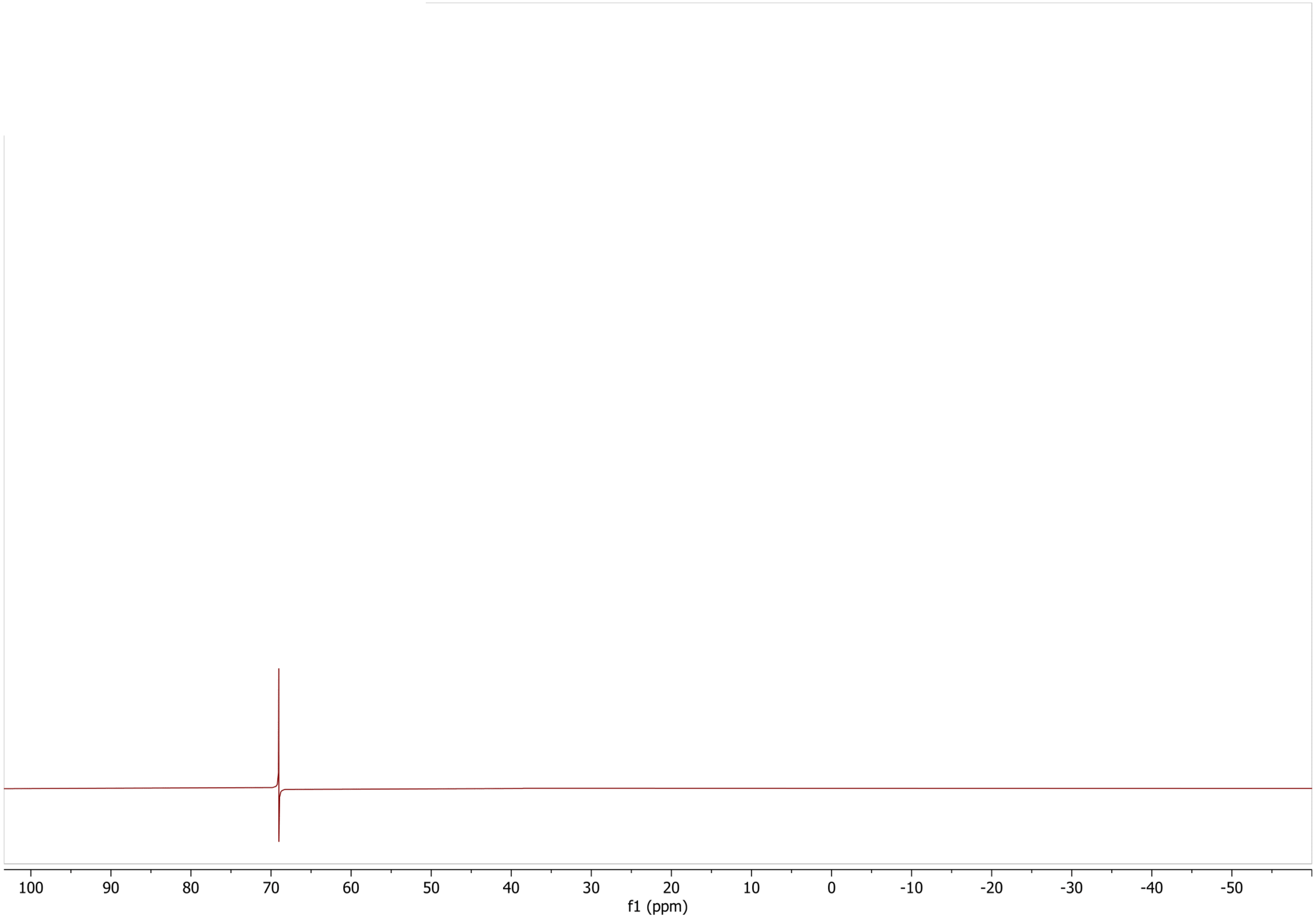

500 MHz ^{1}H spectrum in $CDCl_3$

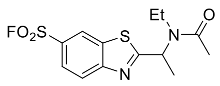

A-13

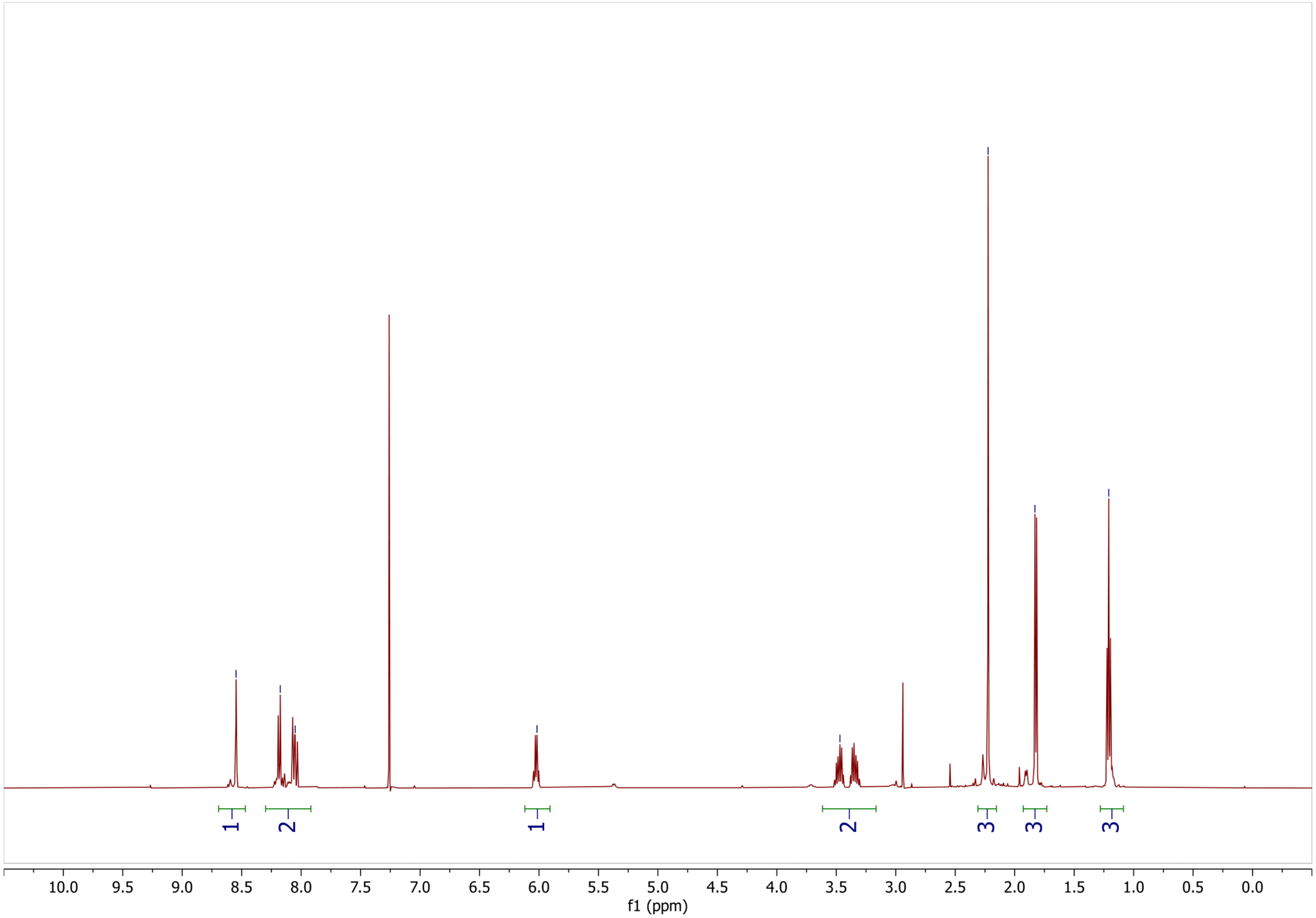

125 MHz ^{13}C spectrum in $CDCl_3$

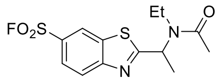

A-13

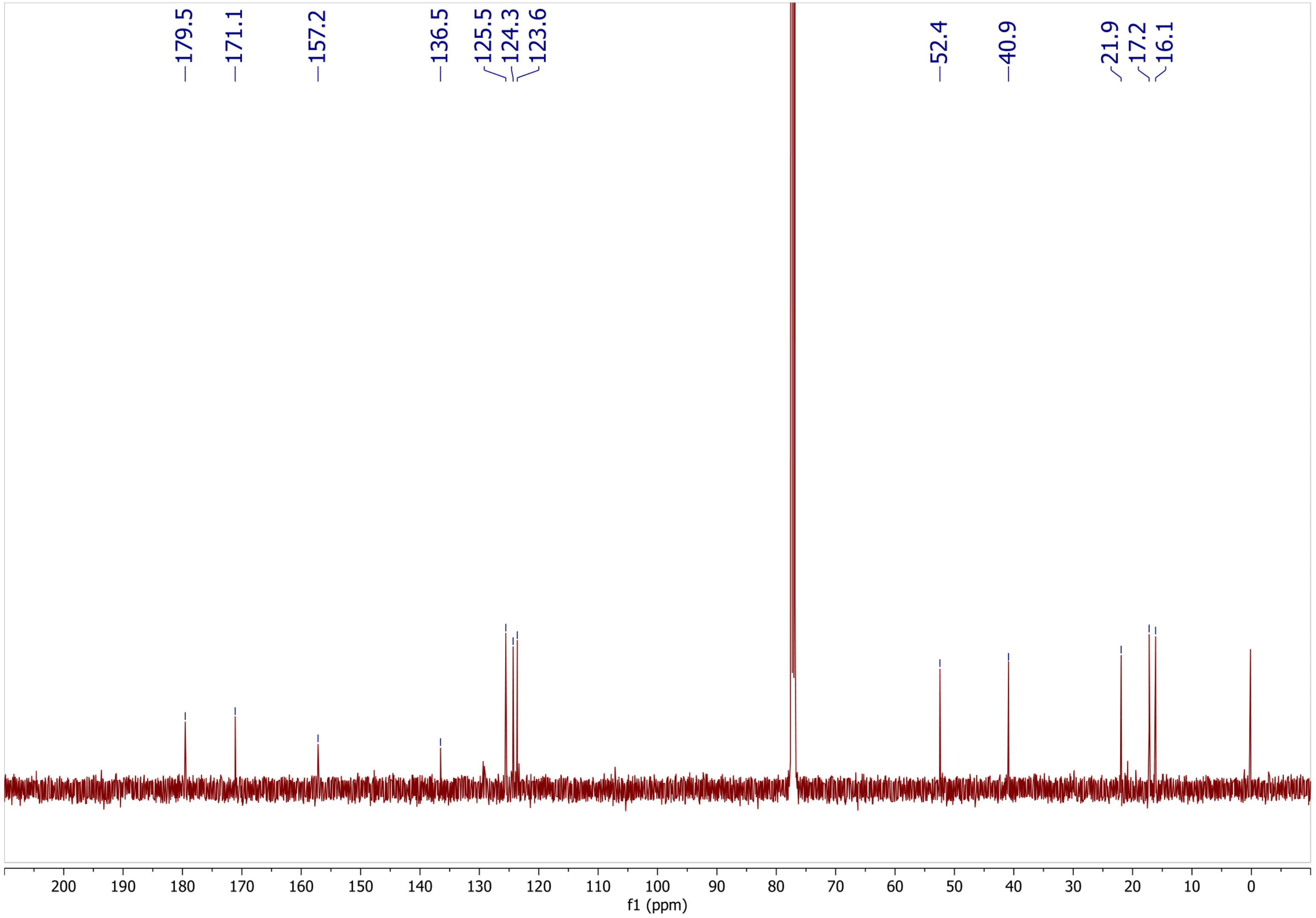

565 MHz ^{19}F spectrum in $CDCl_3$

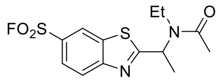

A-13

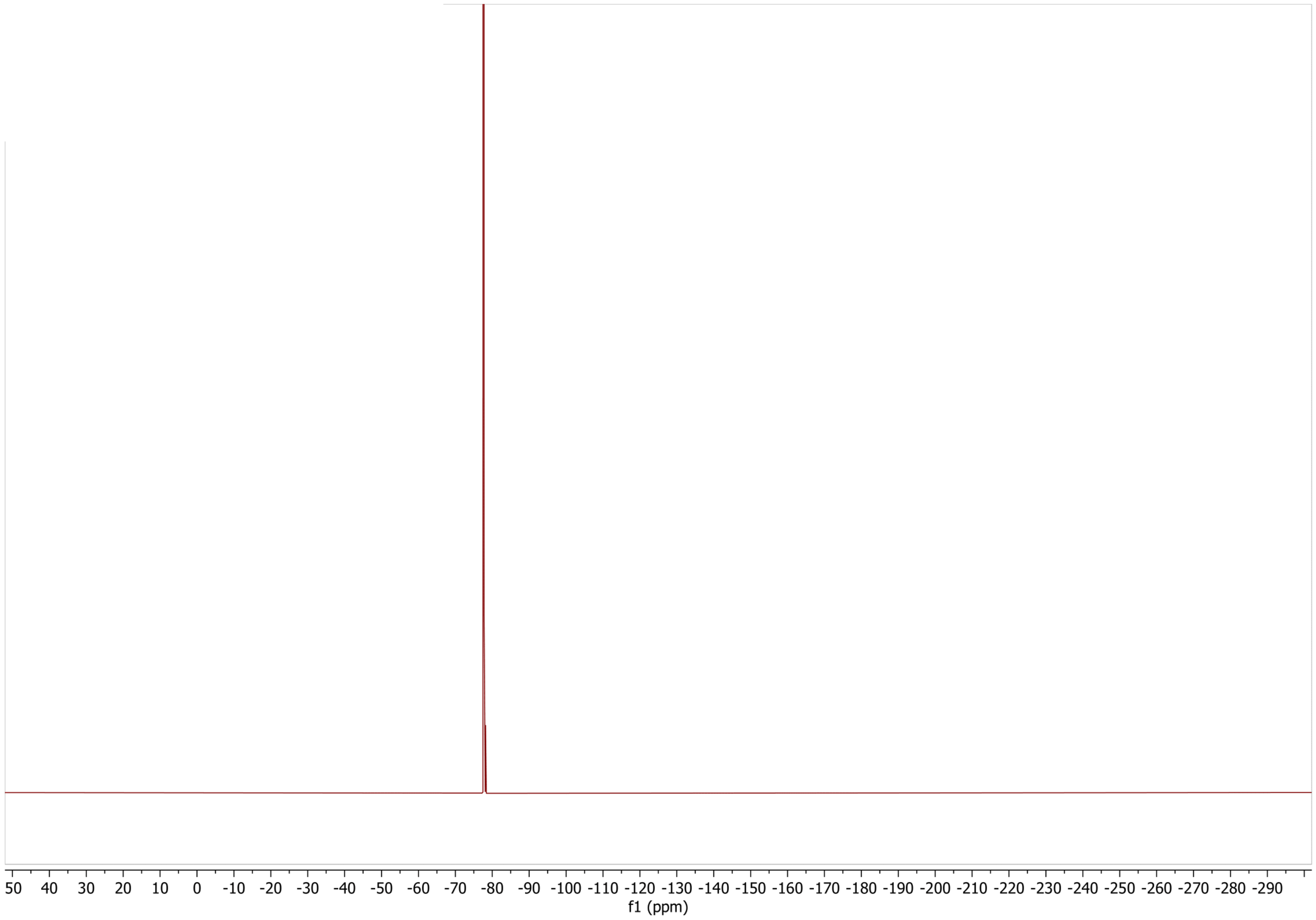

500 MHz ^{1}H spectrum in $CDCl_3$

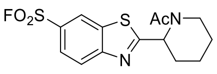

A-14

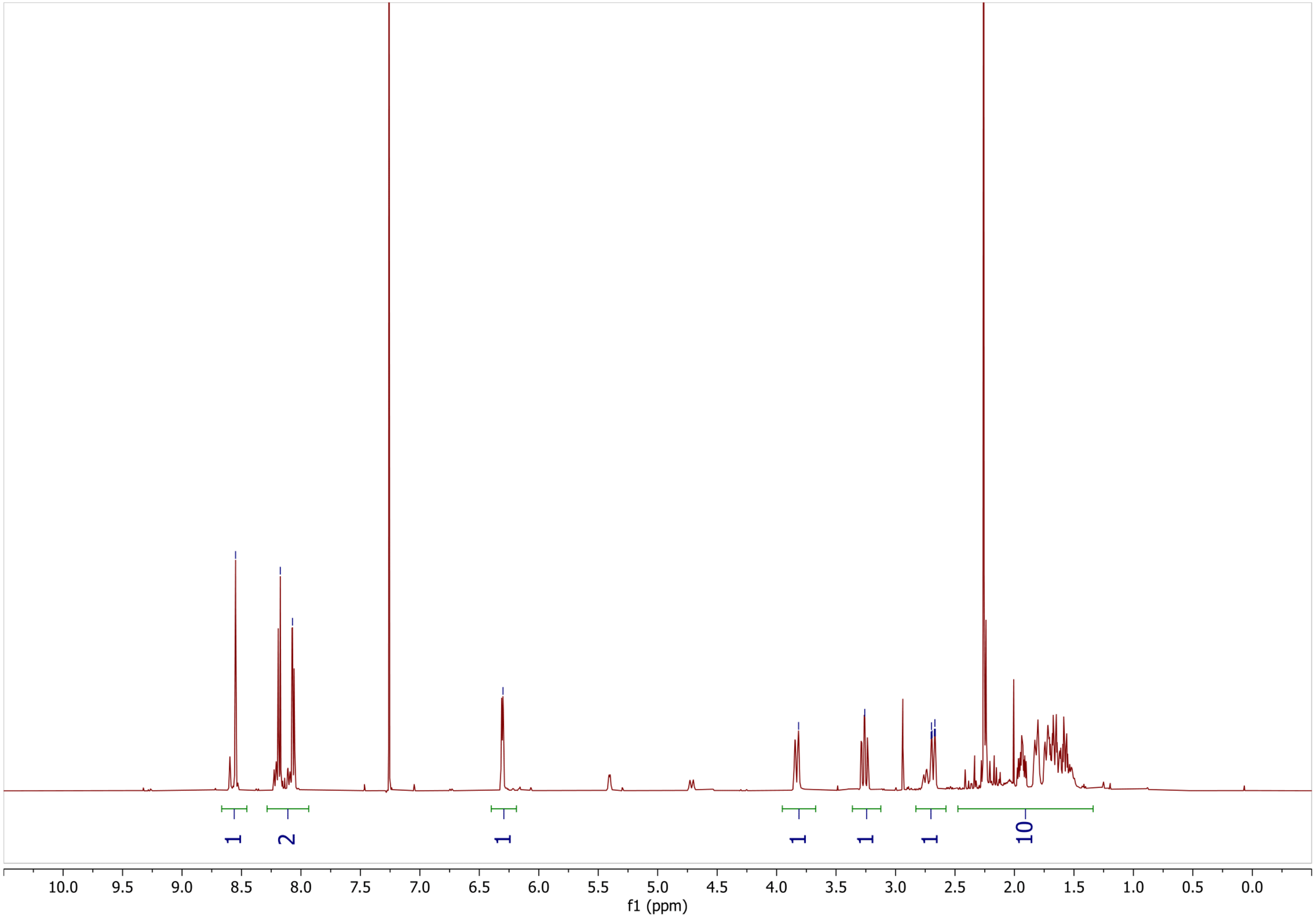

125 MHz ^{13}C spectrum in $CDCl_3$

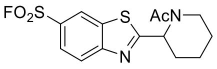

A-14

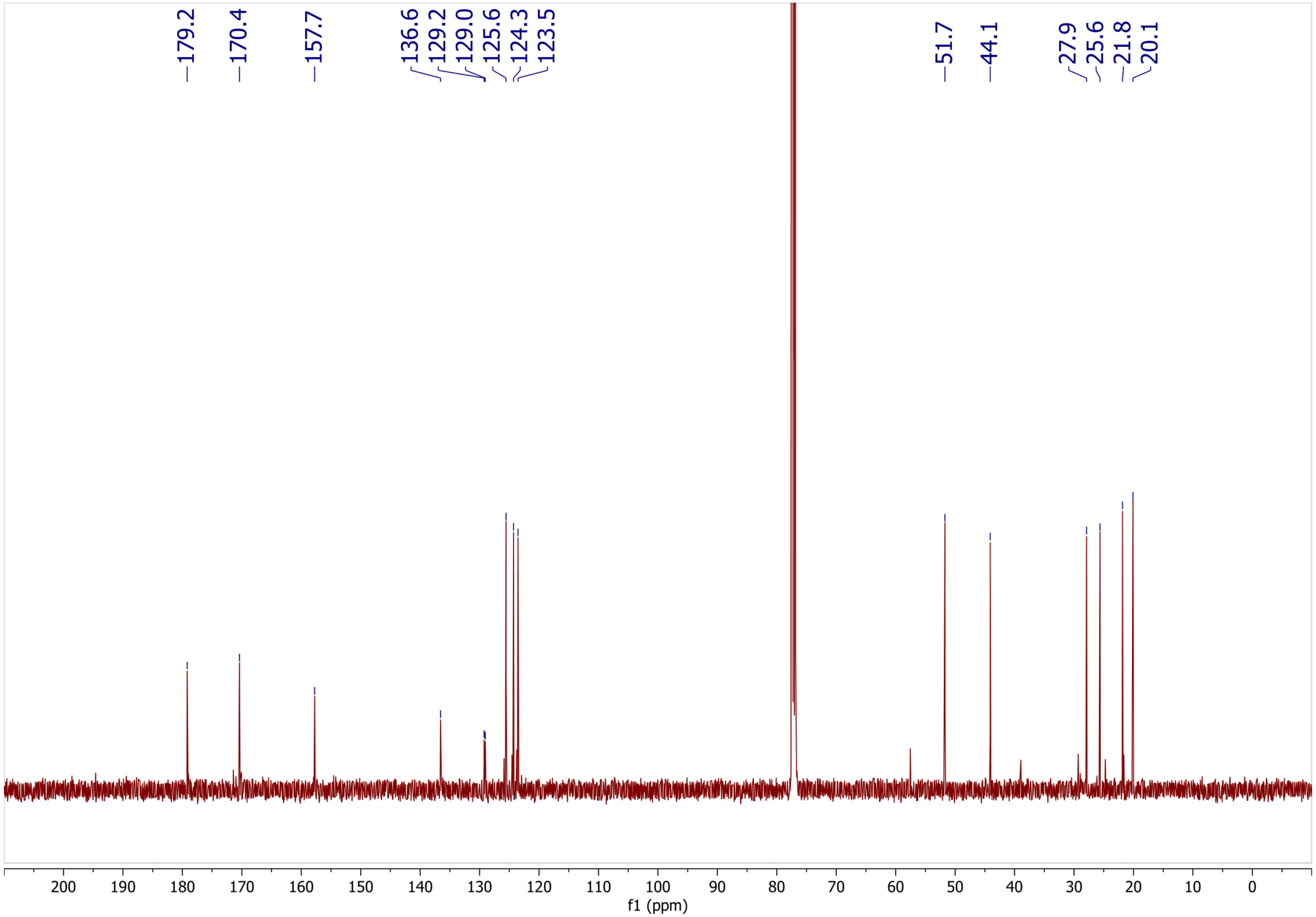

565 MHz ^{19}F spectrum in $CDCl_3$

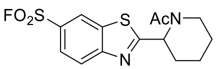

A-14

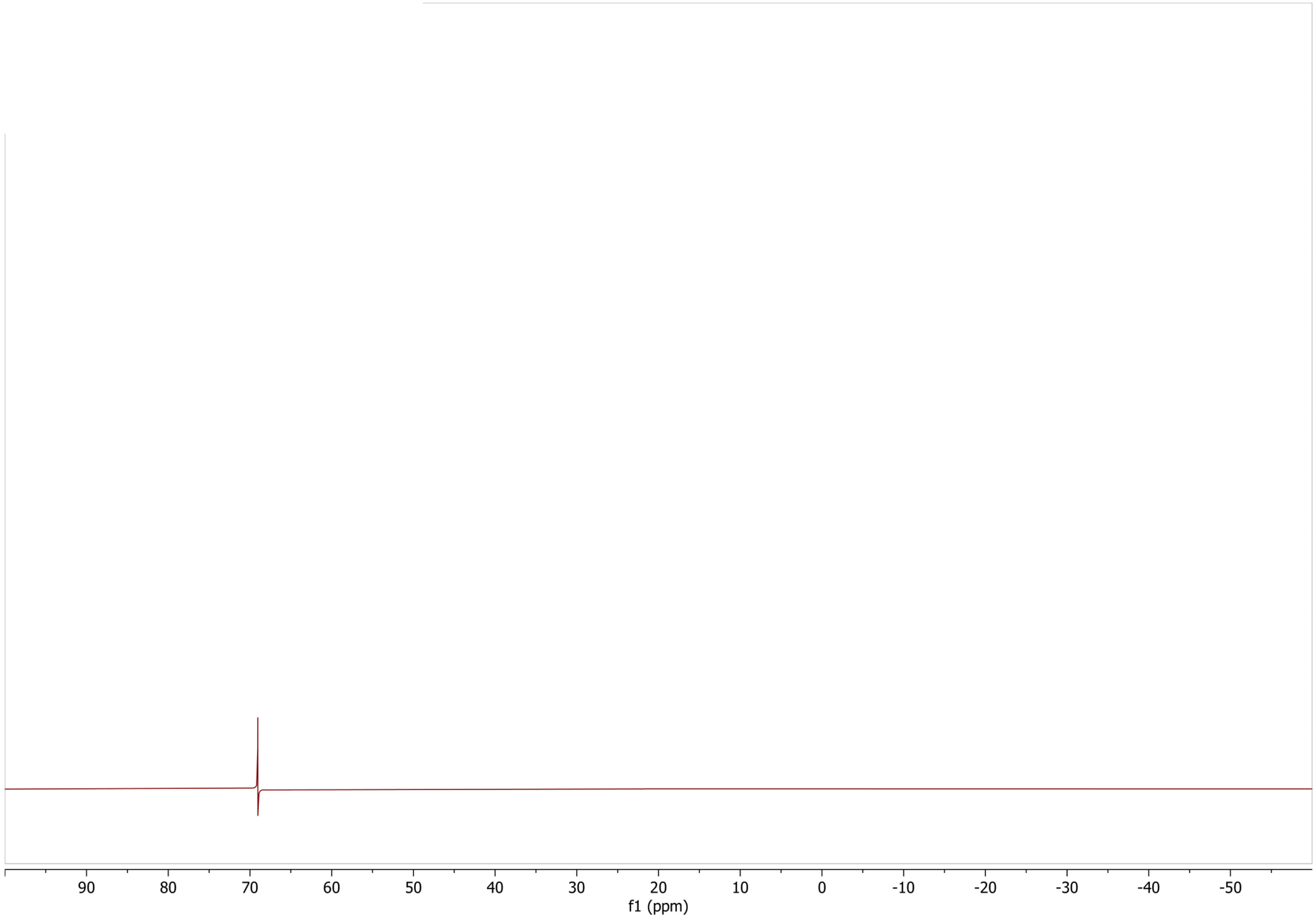

500 MHz ^1H spectrum in $CDCl_3$

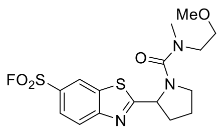

A-20

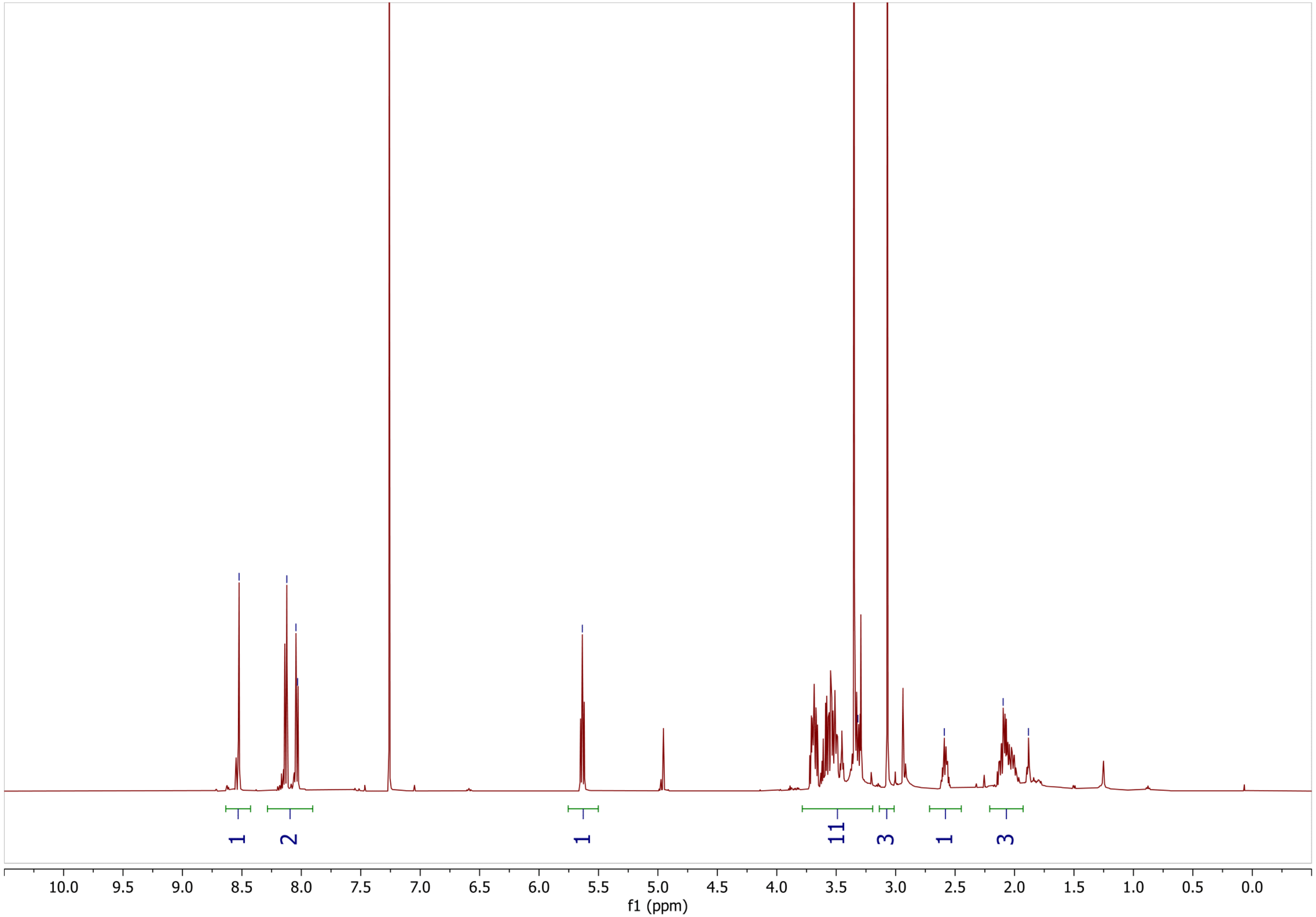

125 MHz ^{13}C spectrum in $CDCl_3$

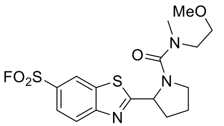

A-20

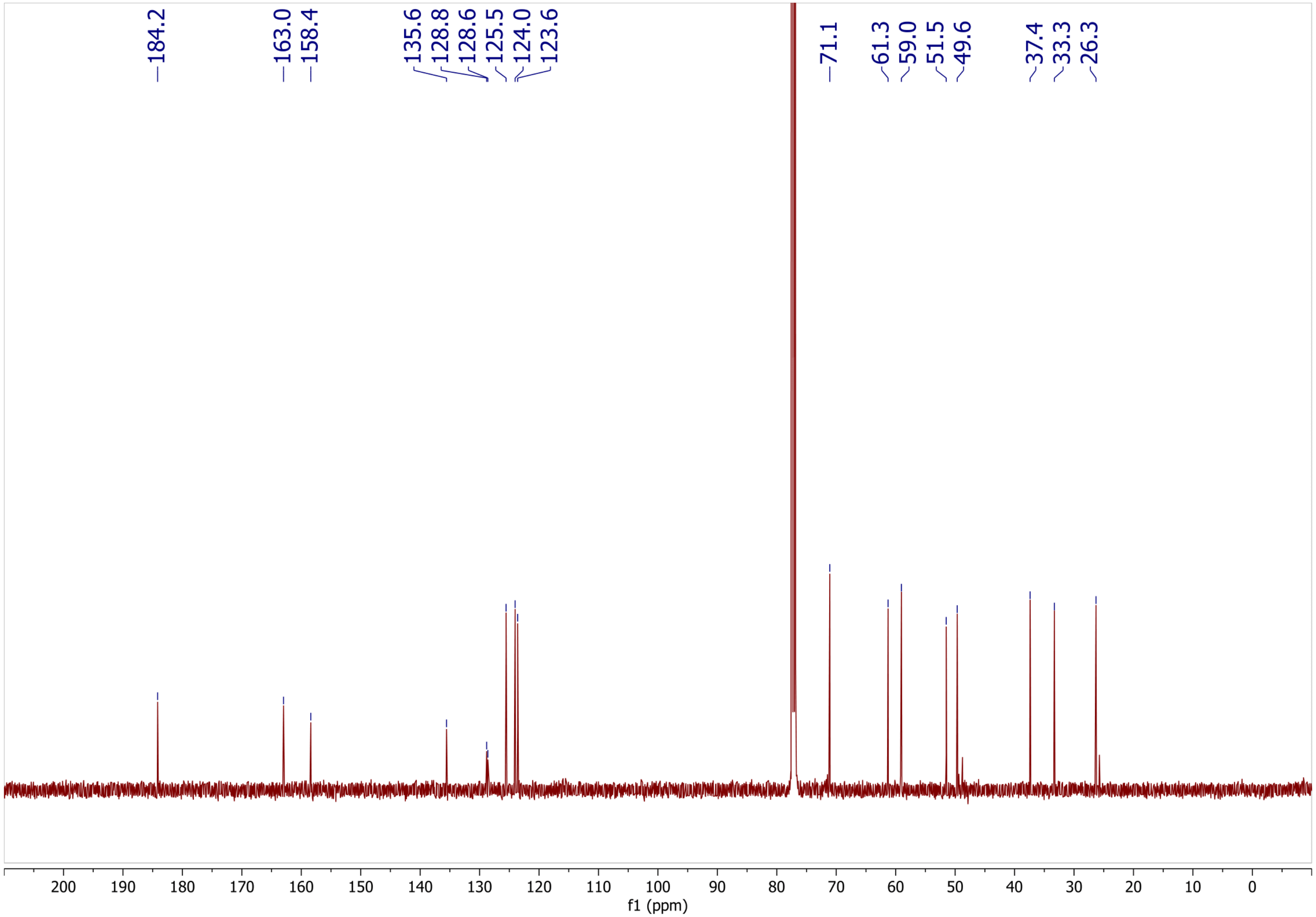

565 MHz ^{19}F spectrum in $CDCl_3$

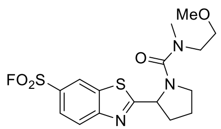

A-20

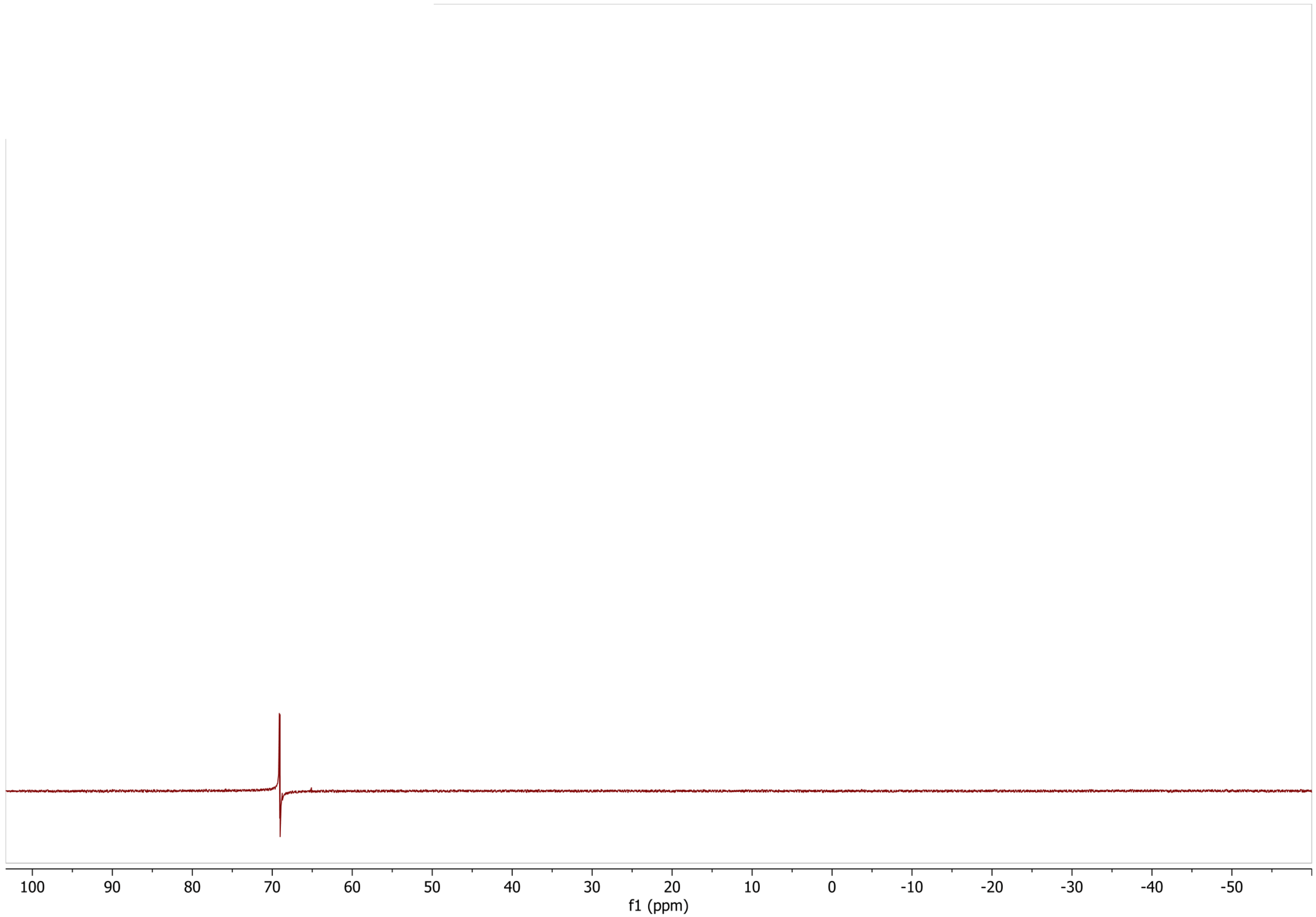

500 MHz ^{1}H spectrum in $CDCl_3$

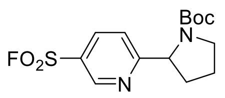

C-1

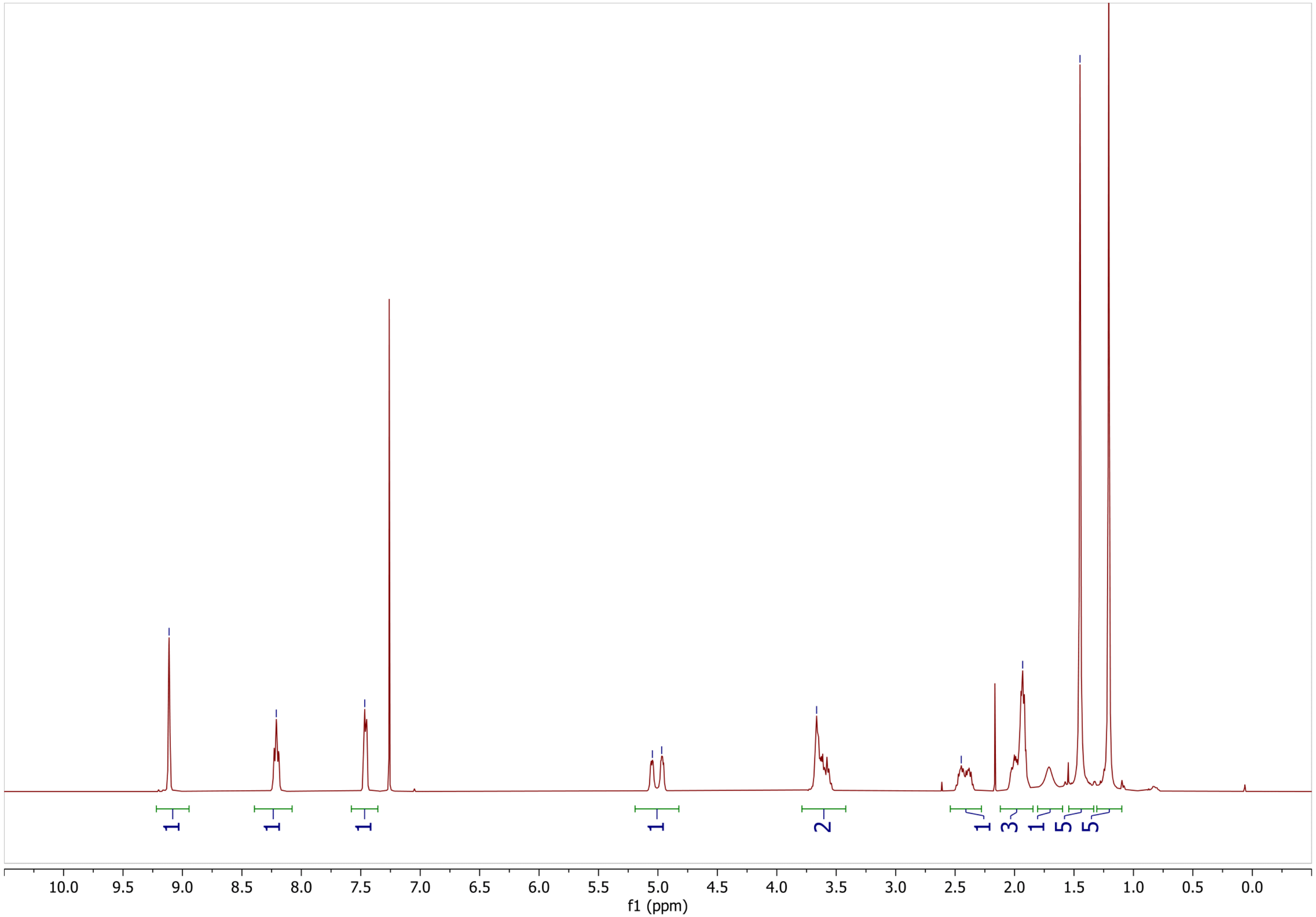

125 MHz ^{13}C spectrum in $CDCl_3$

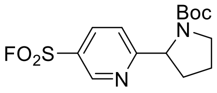

C-1

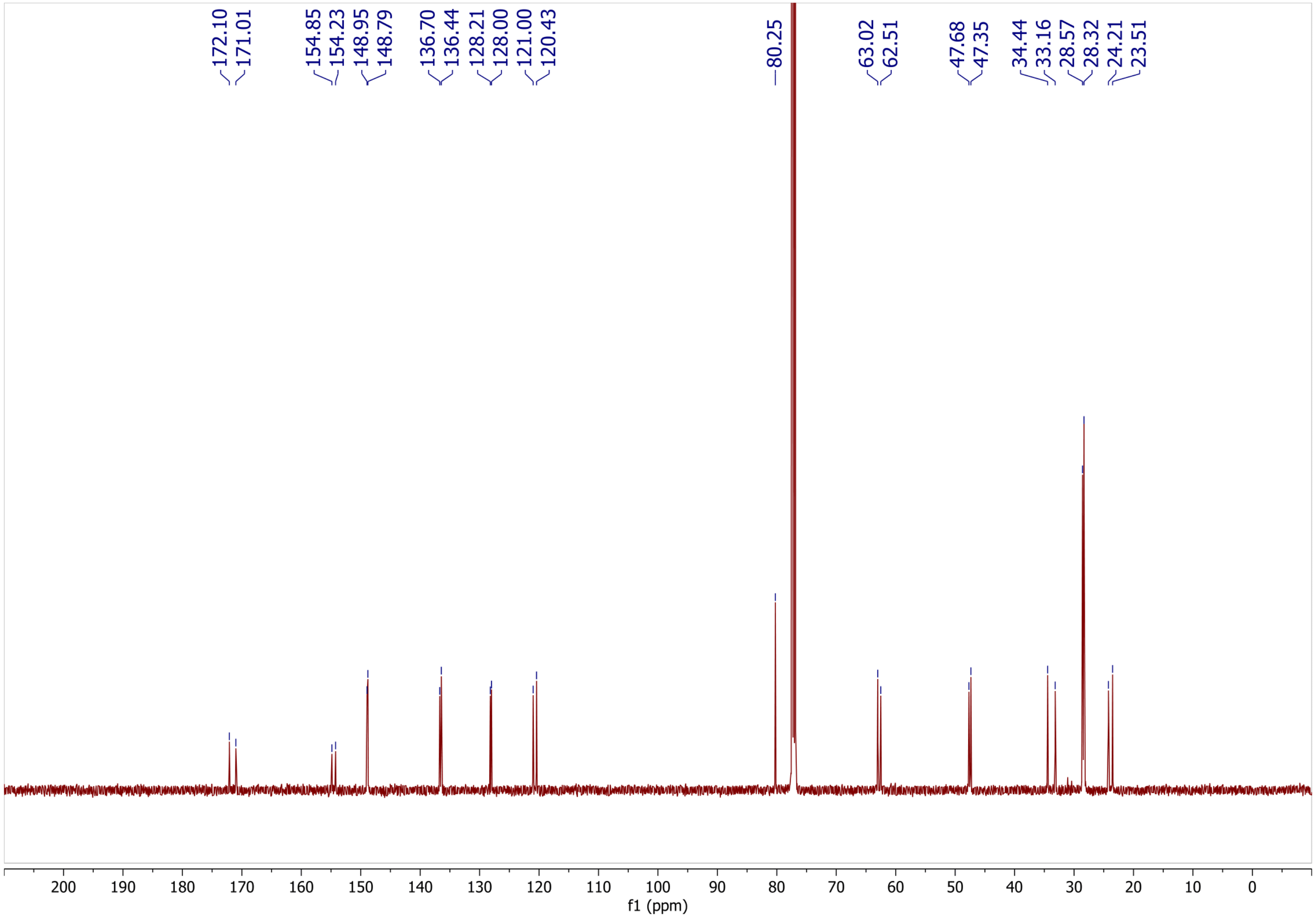

500 MHz ^{1}H spectrum in $CDCl_3$

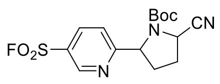

C-3

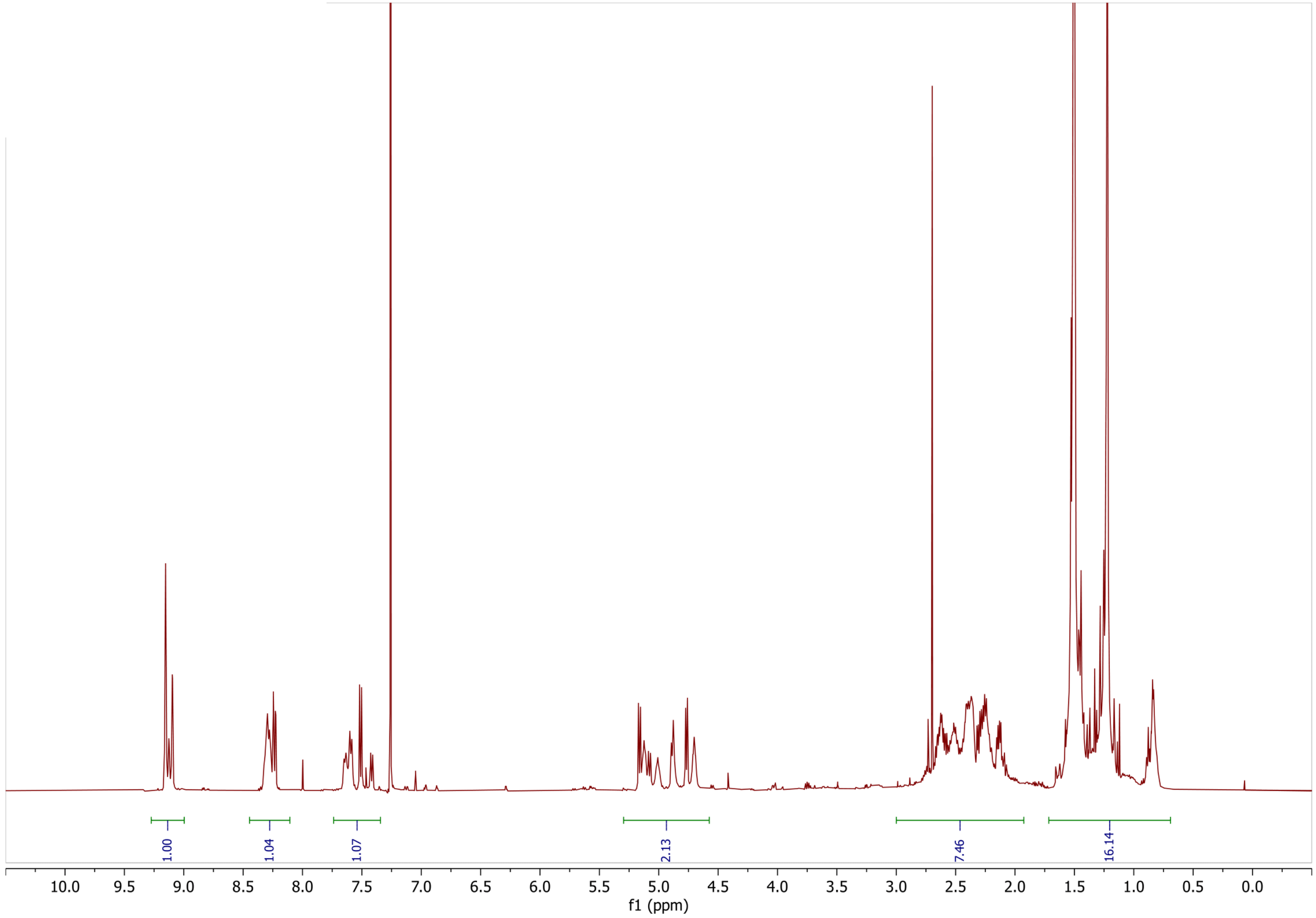

125 MHz ^{13}C spectrum in $CDCl_3$

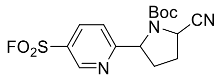

C-3

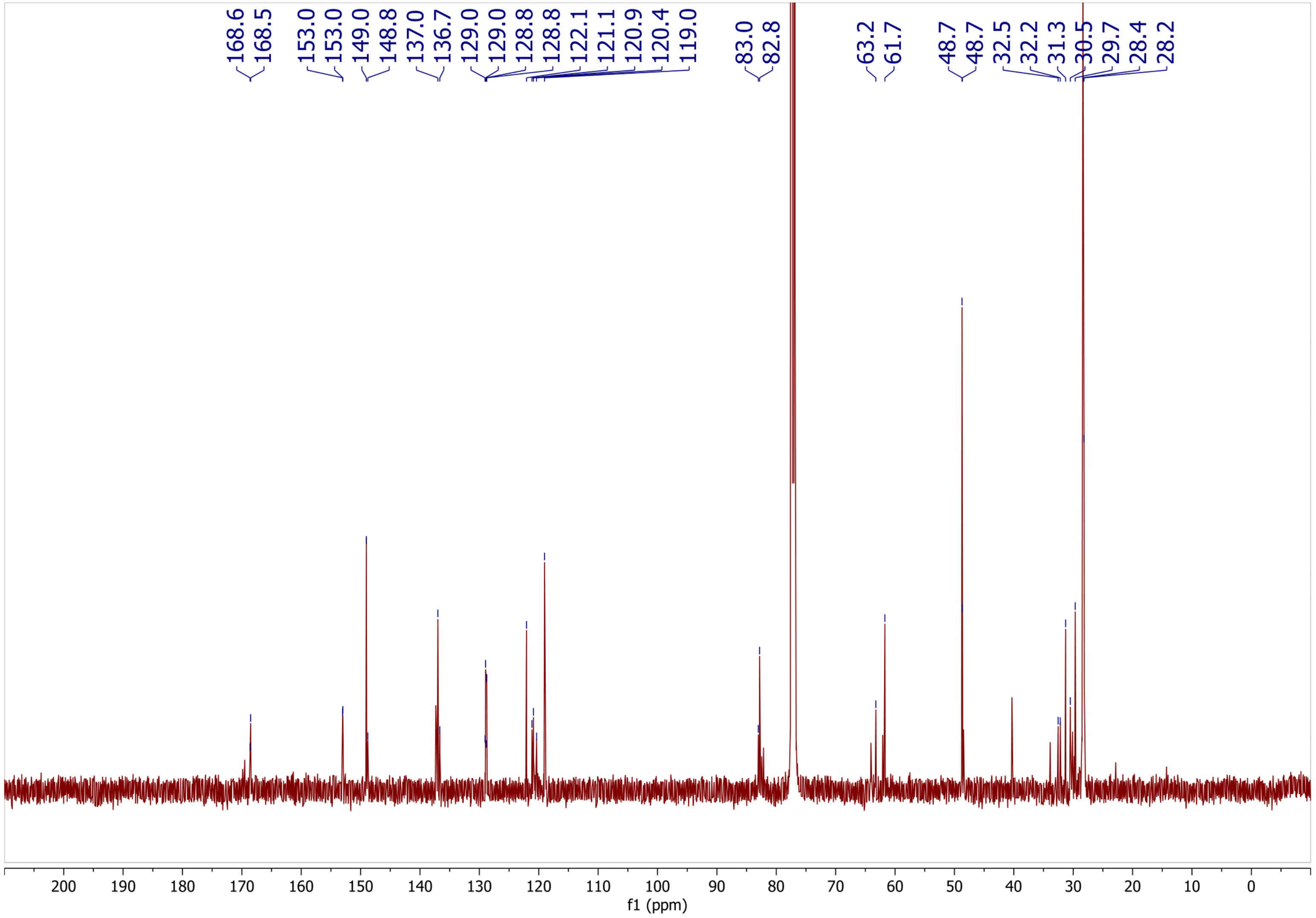

565 MHz ^{19}F spectrum in $CDCl_3$

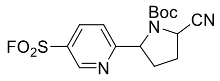

C-3

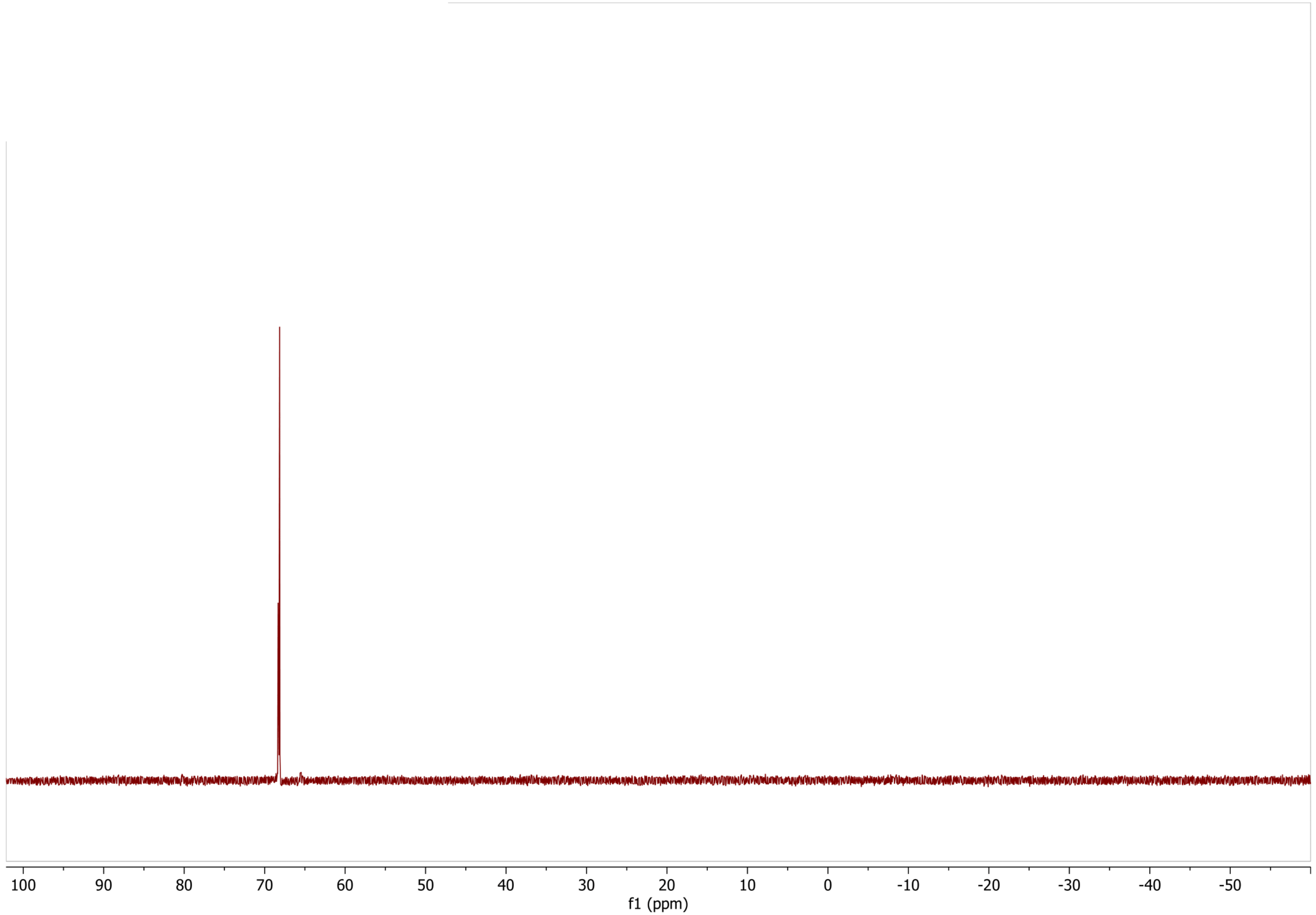

500 MHz ^{1}H spectrum in $CDCl_3$

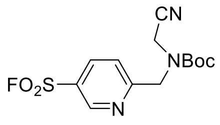

C-4

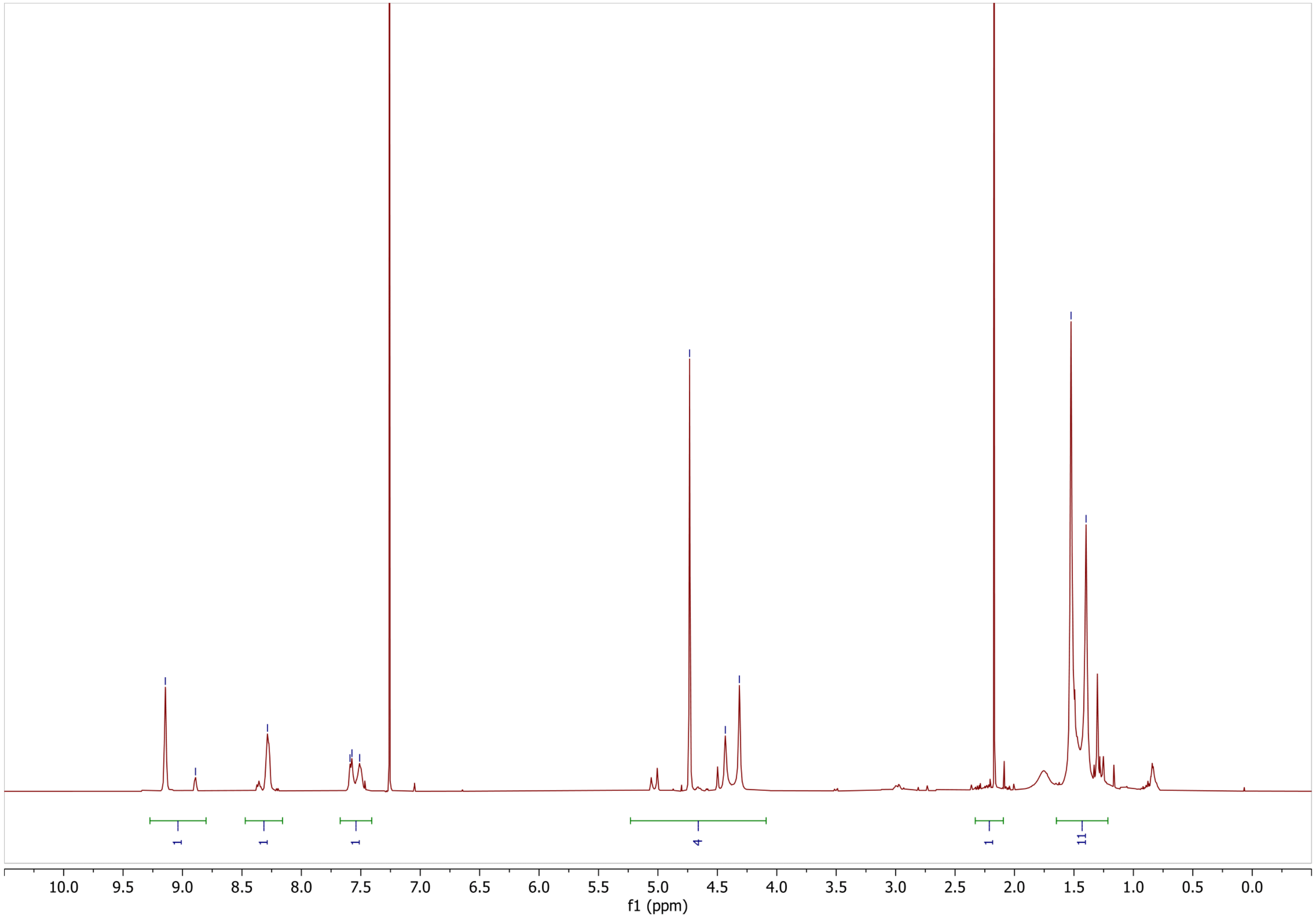

125 MHz ^{13}C spectrum in $CDCl_3$

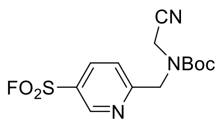

C-4

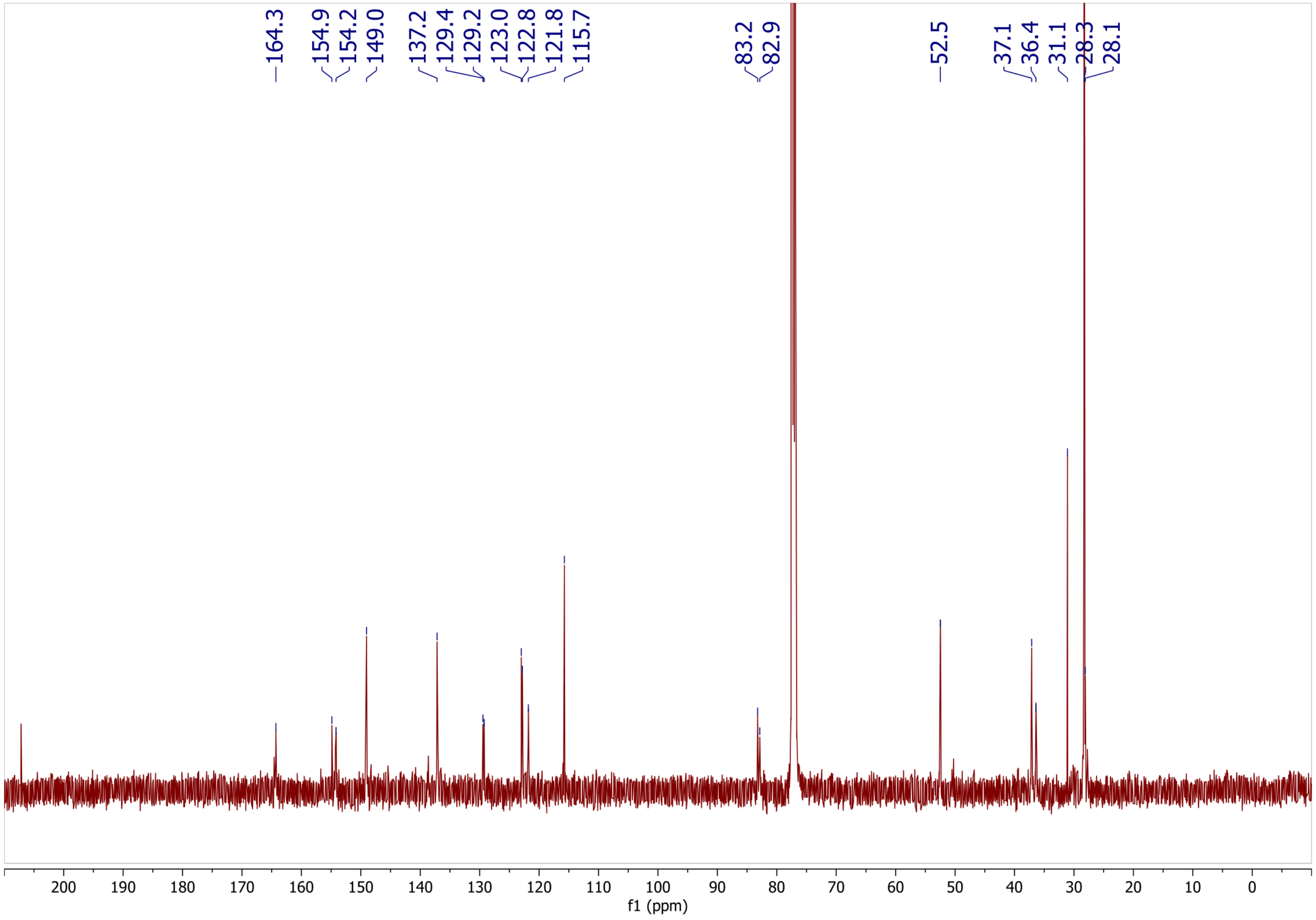

565 MHz ^{19}F spectrum in $CDCl_3$

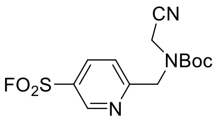

C-4

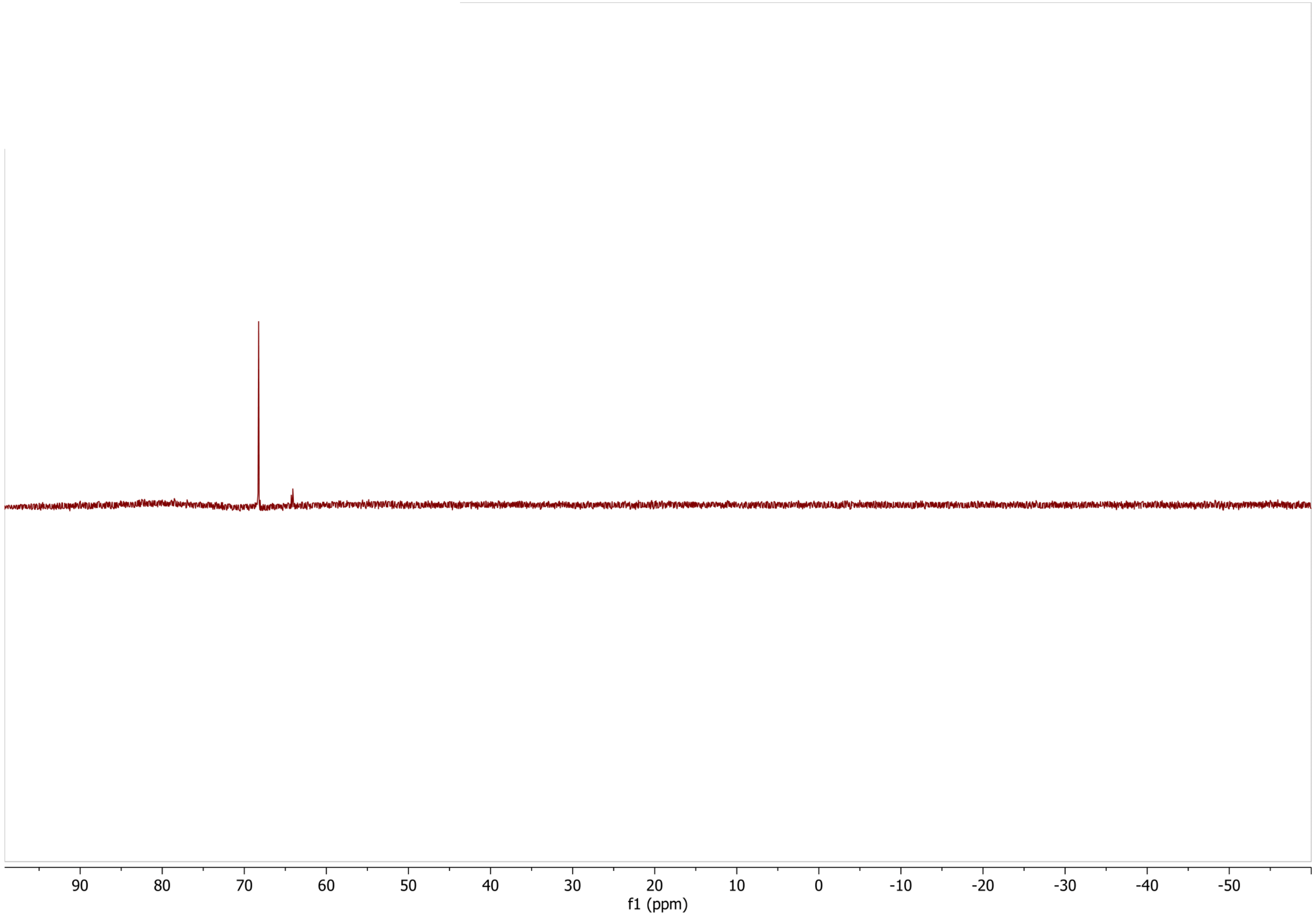

500 MHz ^{1}H spectrum in $CDCl_3$

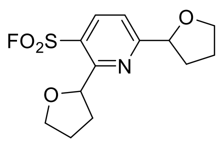

C-6

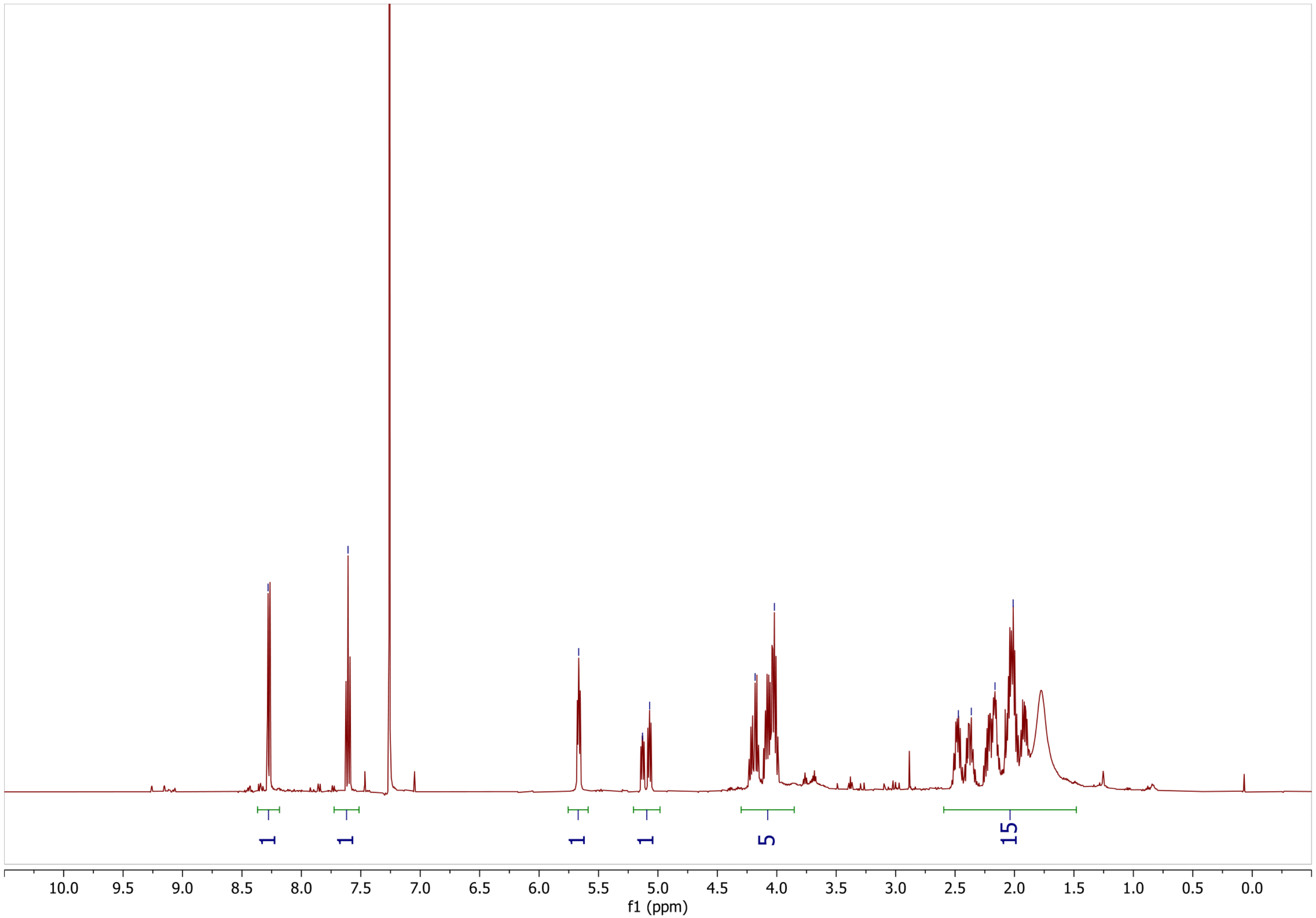

125 MHz ^{13}C spectrum in $CDCl_3$

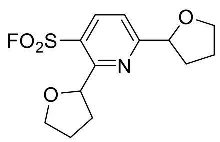

C-6

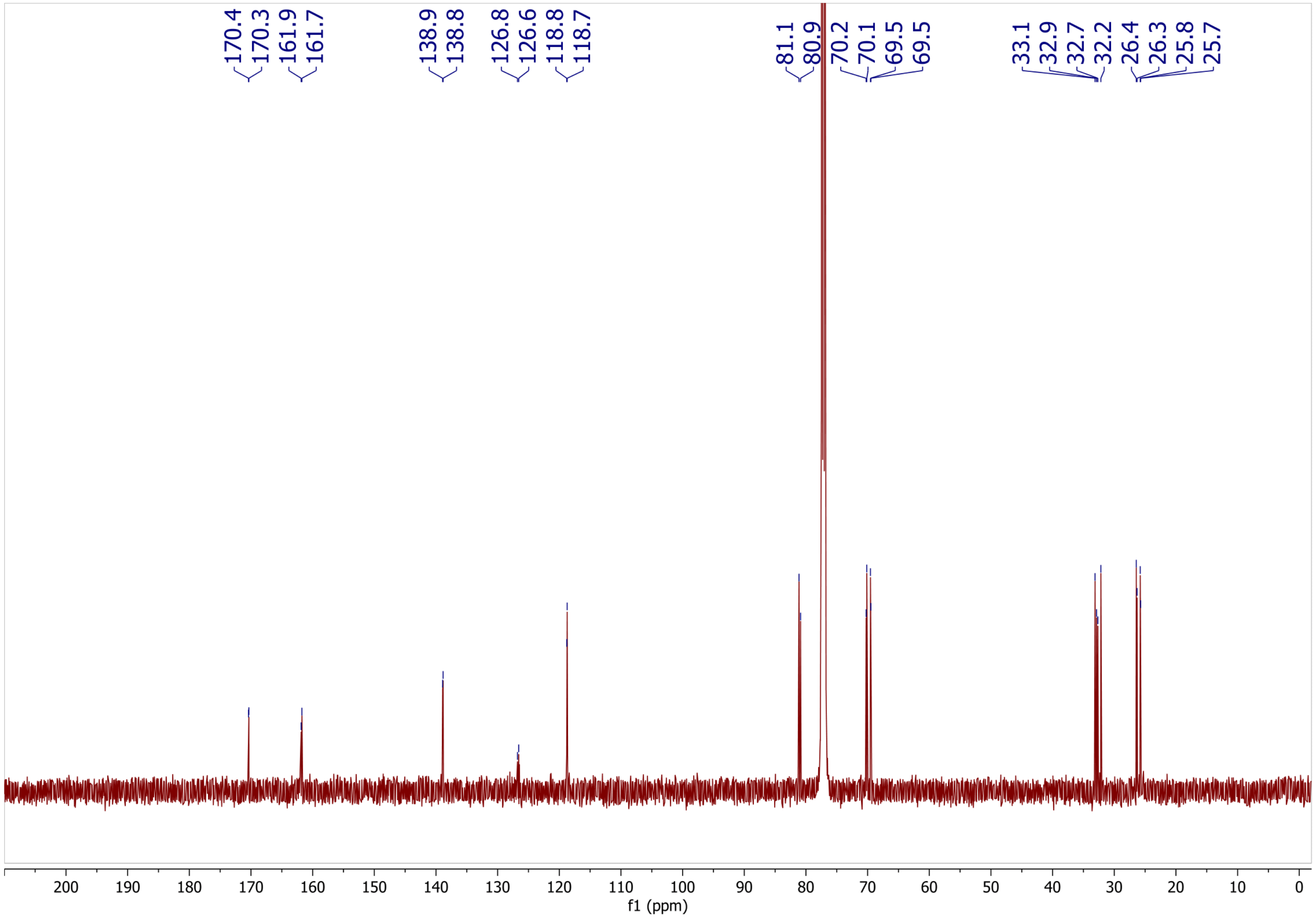

565 MHz ^{19}F spectrum in $CDCl_3$

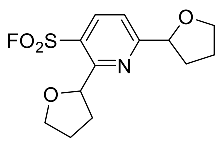

C-6

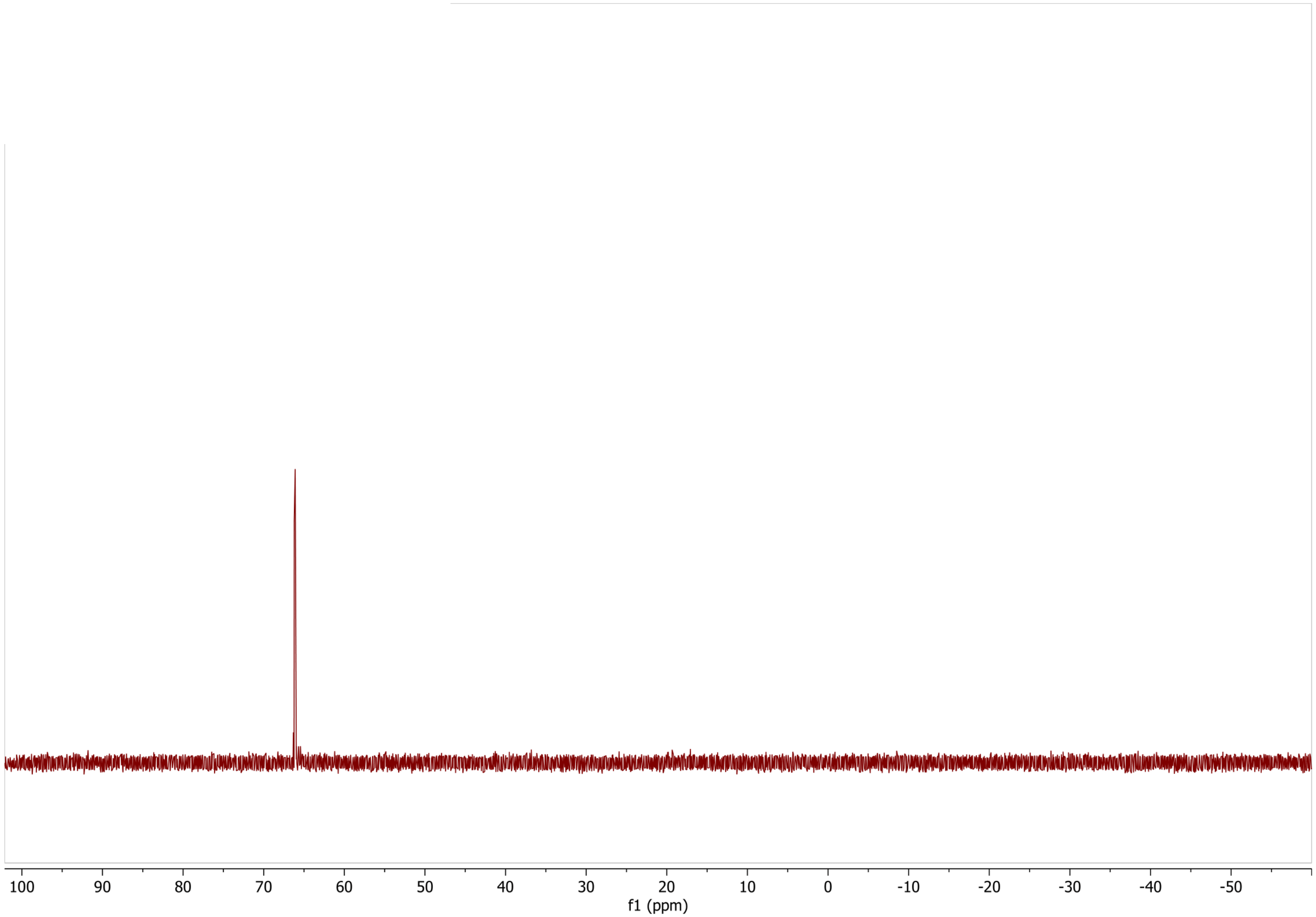

500 MHz ^1H spectrum in $CDCl_3$

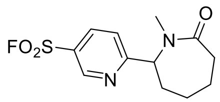

C-8a

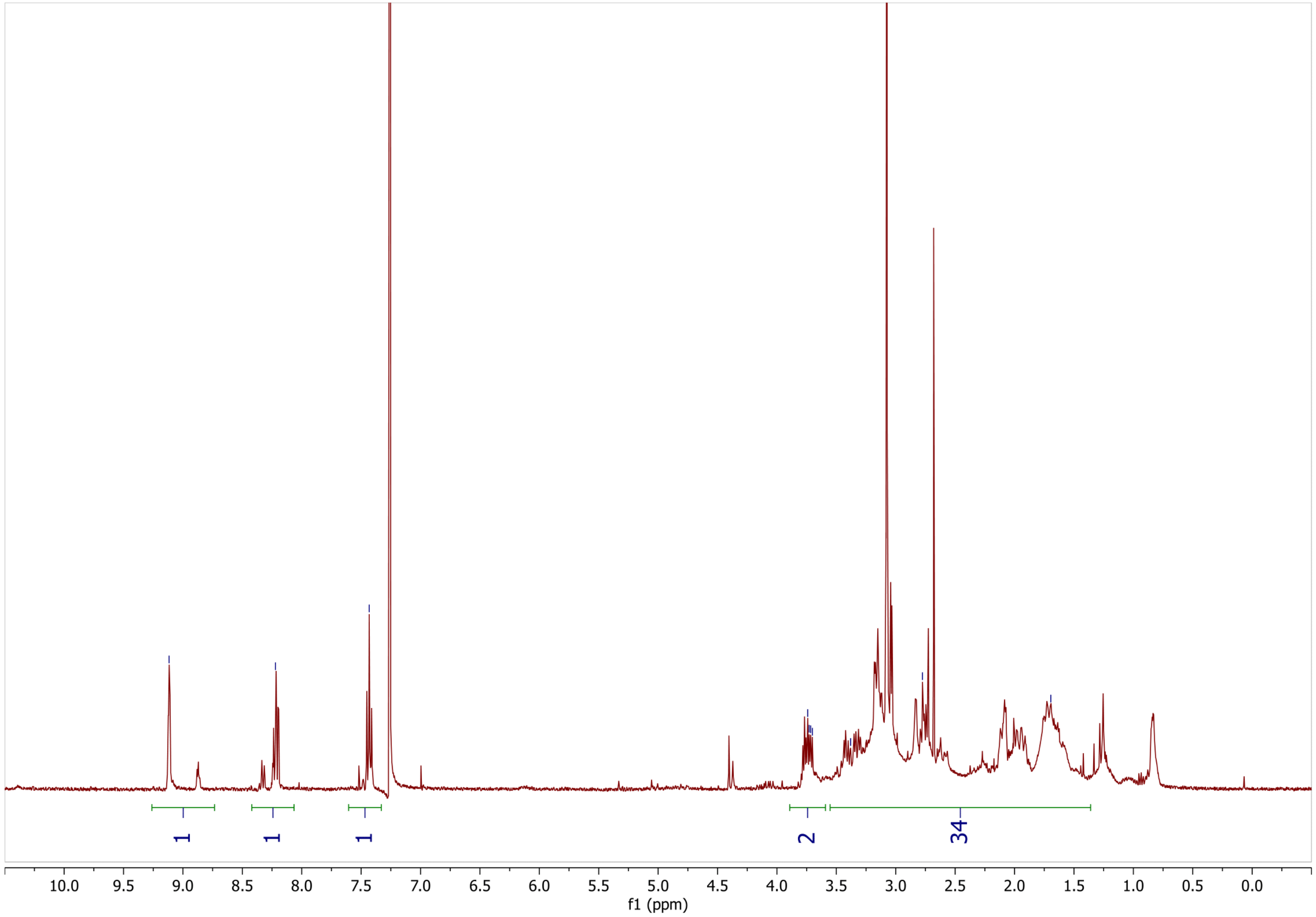

125 MHz ^{13}C spectrum in $CDCl_3$

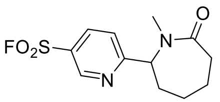

C-8a

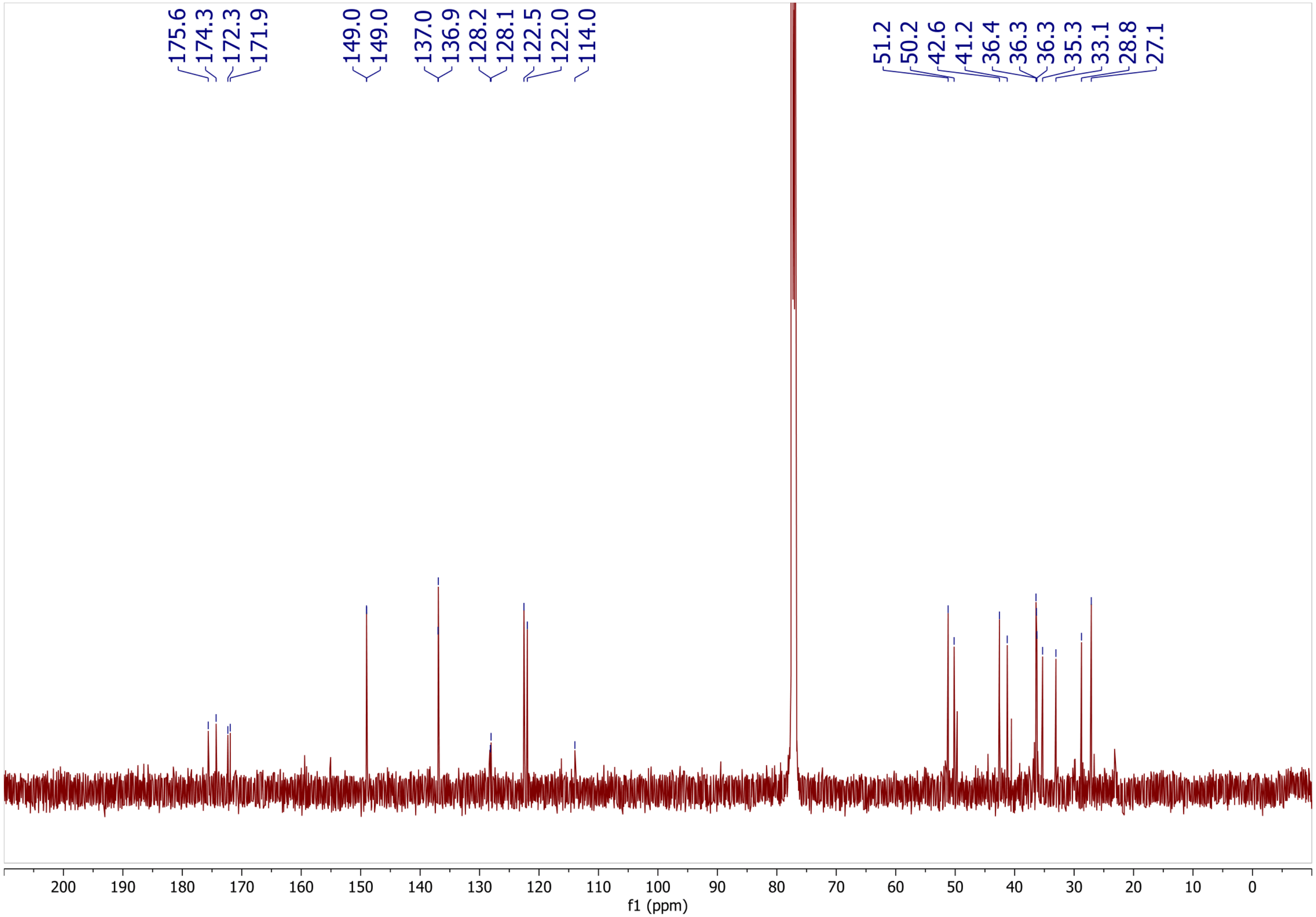

565 MHz ^{19}F spectrum in $CDCl_3$

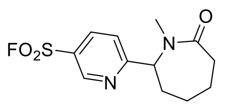

C-8a

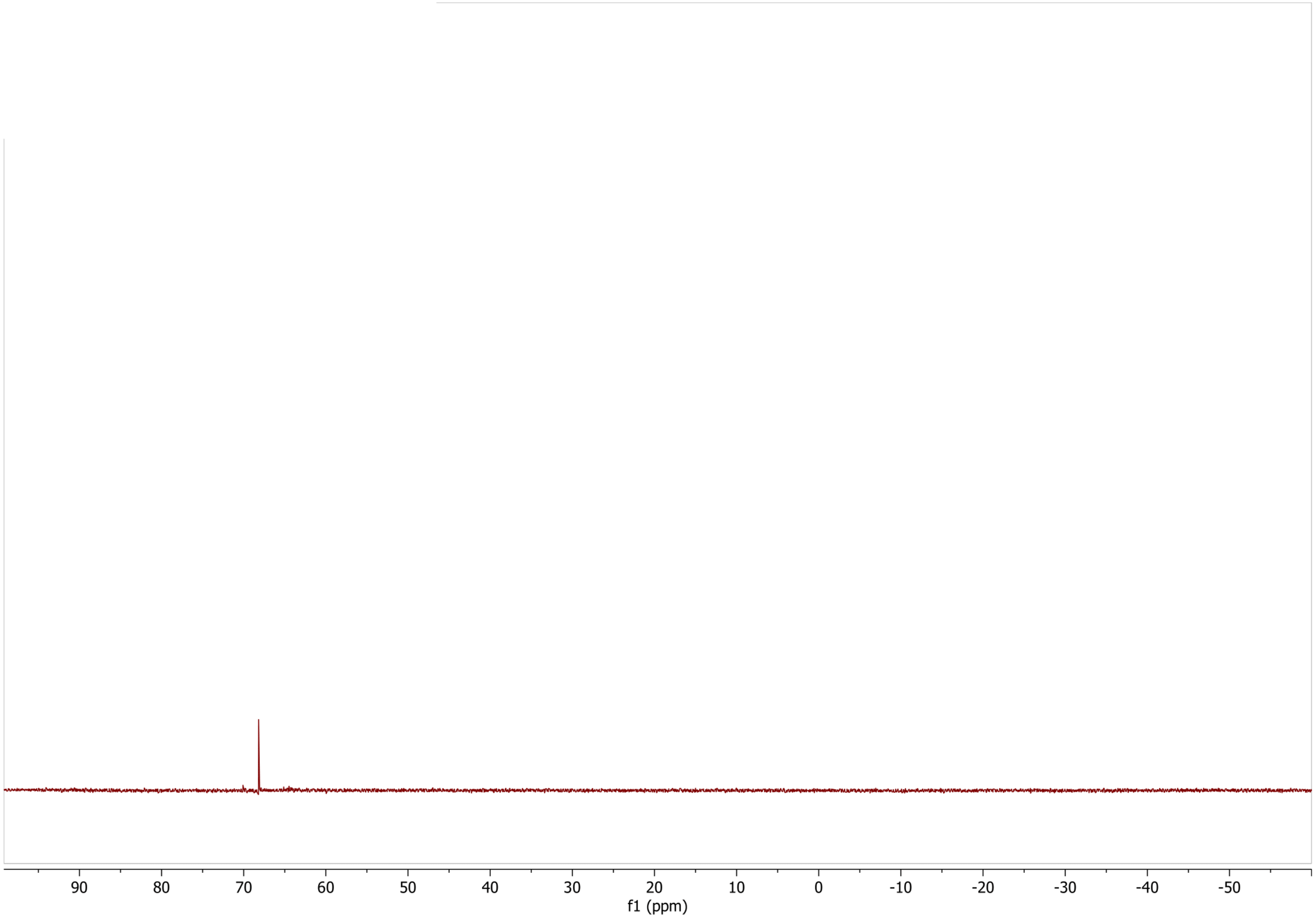

500 MHz ^{1}H spectrum in $CDCl_3$

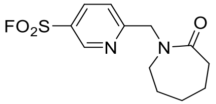

C-8b

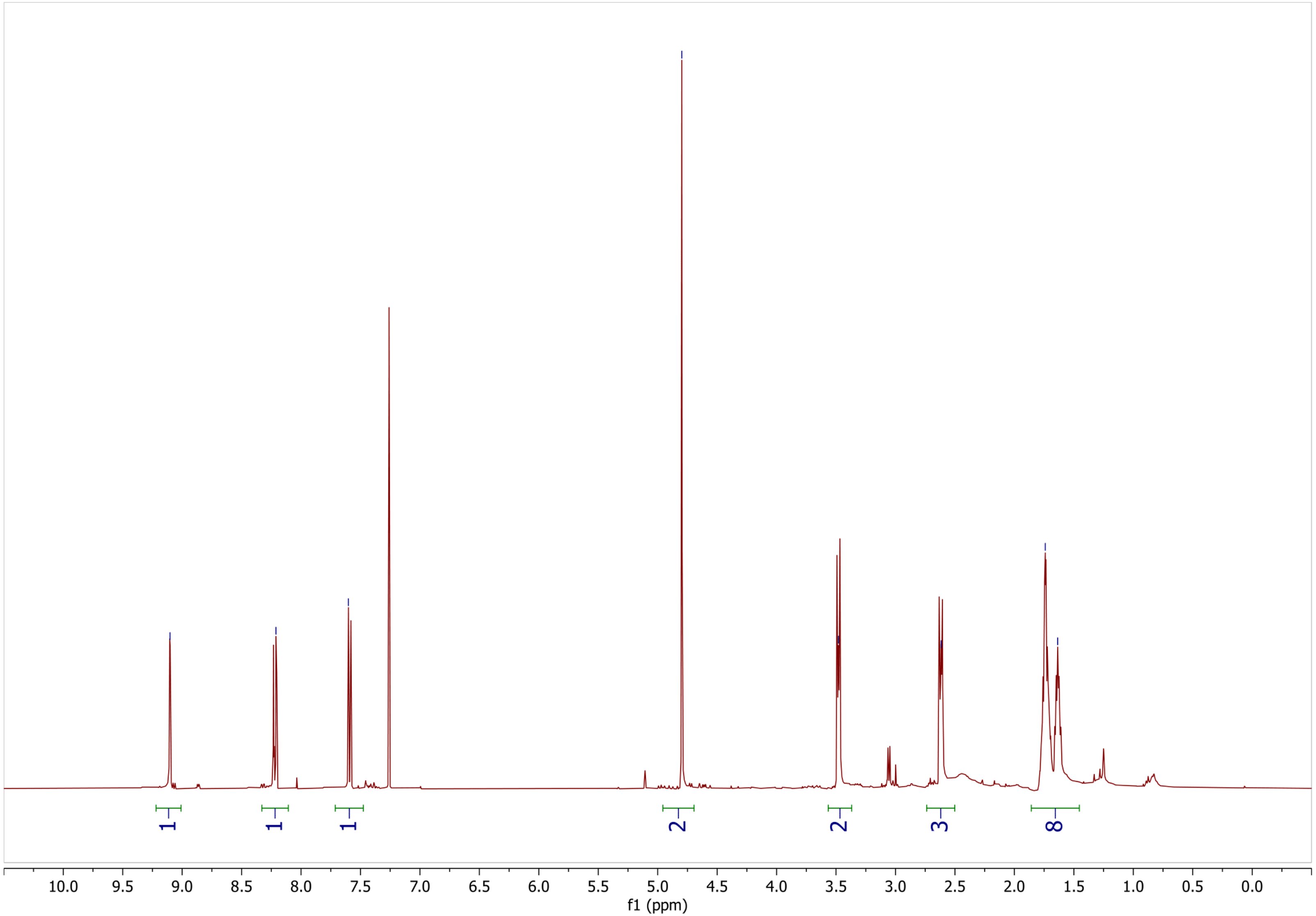

125 MHz ^{13}C spectrum in $CDCl_3$

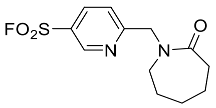

C-8b

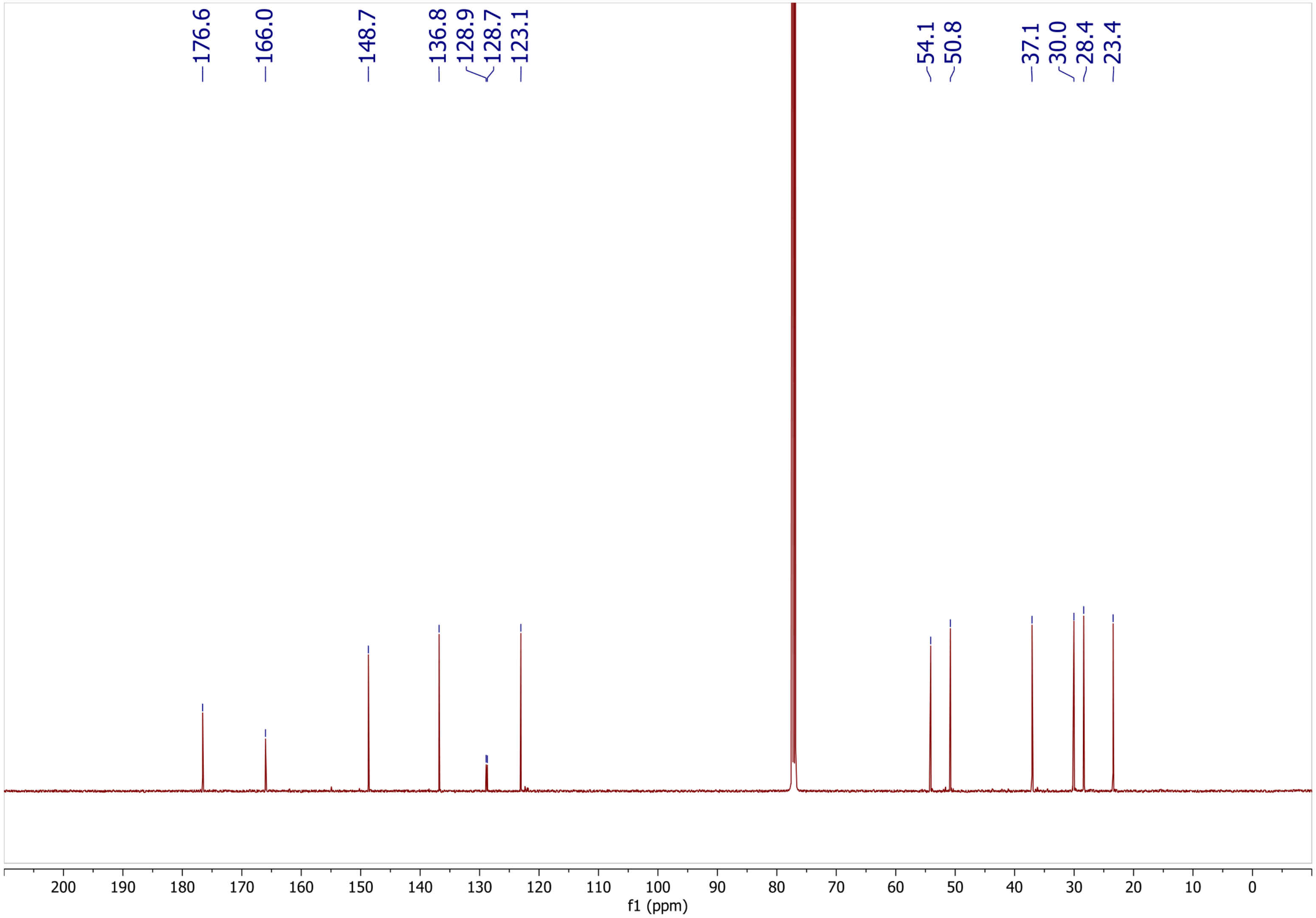

500 MHz ^{1}H spectrum in $CDCl_3$

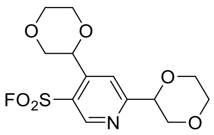

C-10a

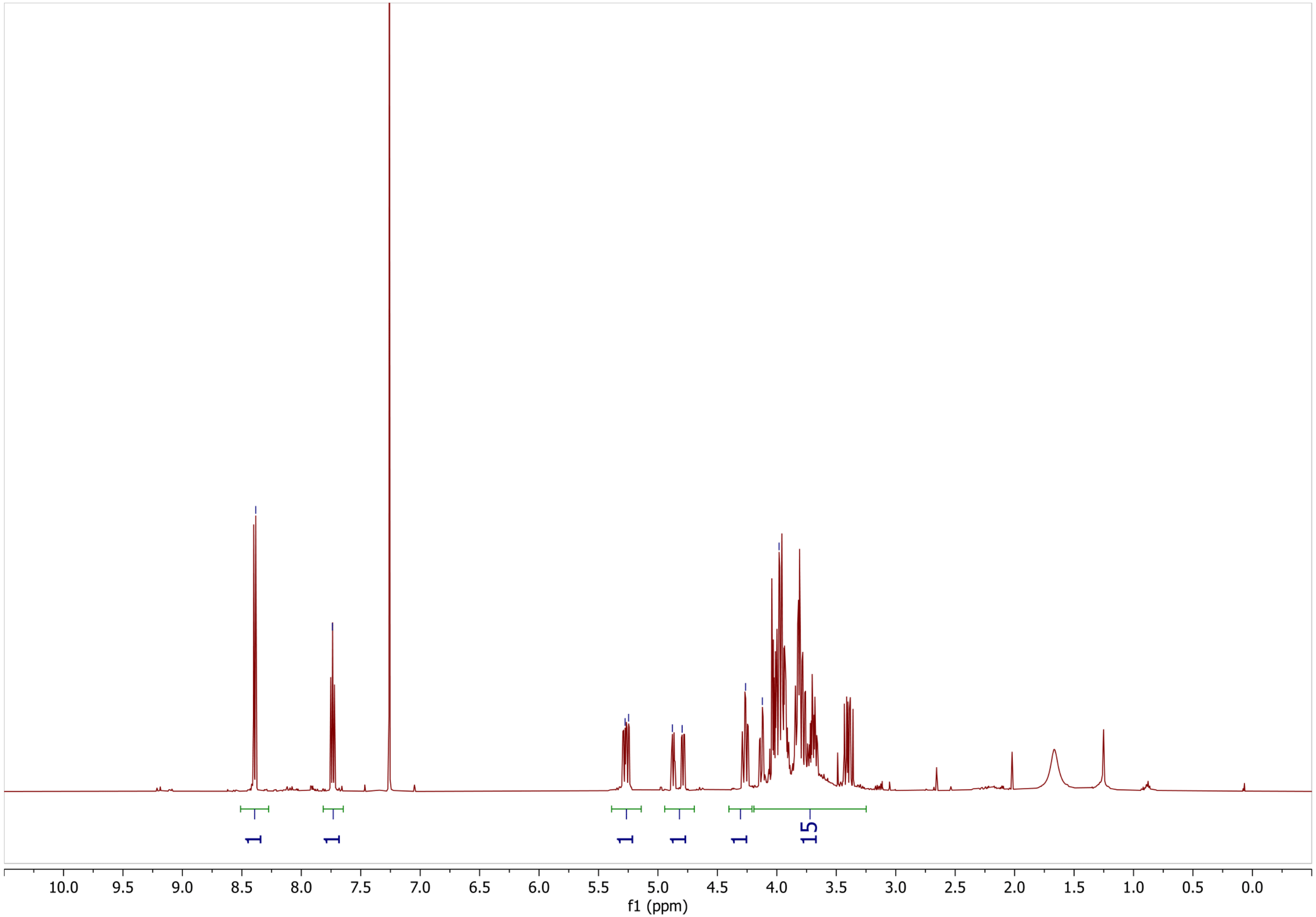

125 MHz ^{13}C spectrum in $CDCl_3$

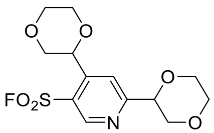

C-10a

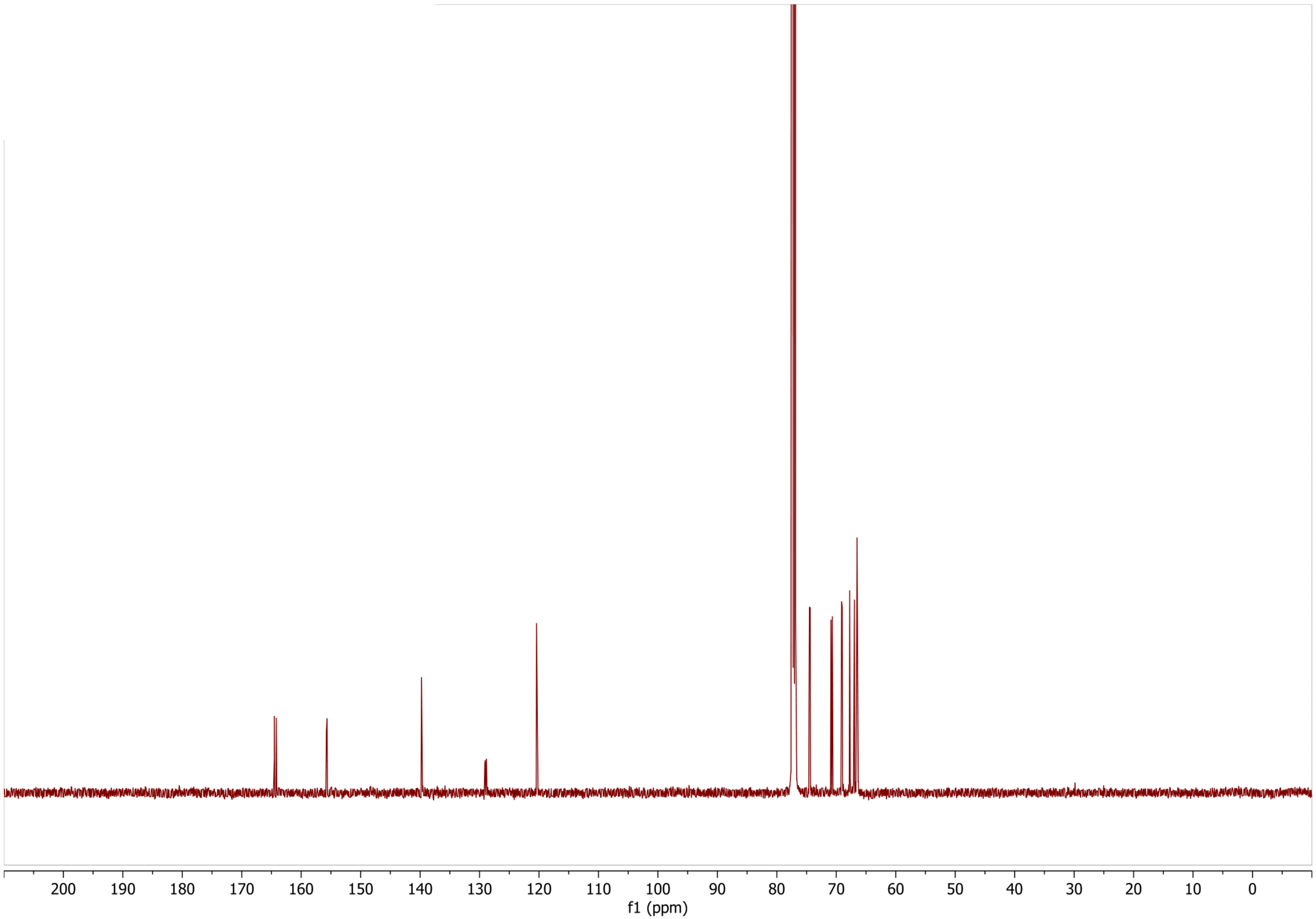

565 MHz ^{19}F spectrum in $CDCl_3$

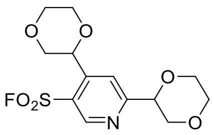

C-10a

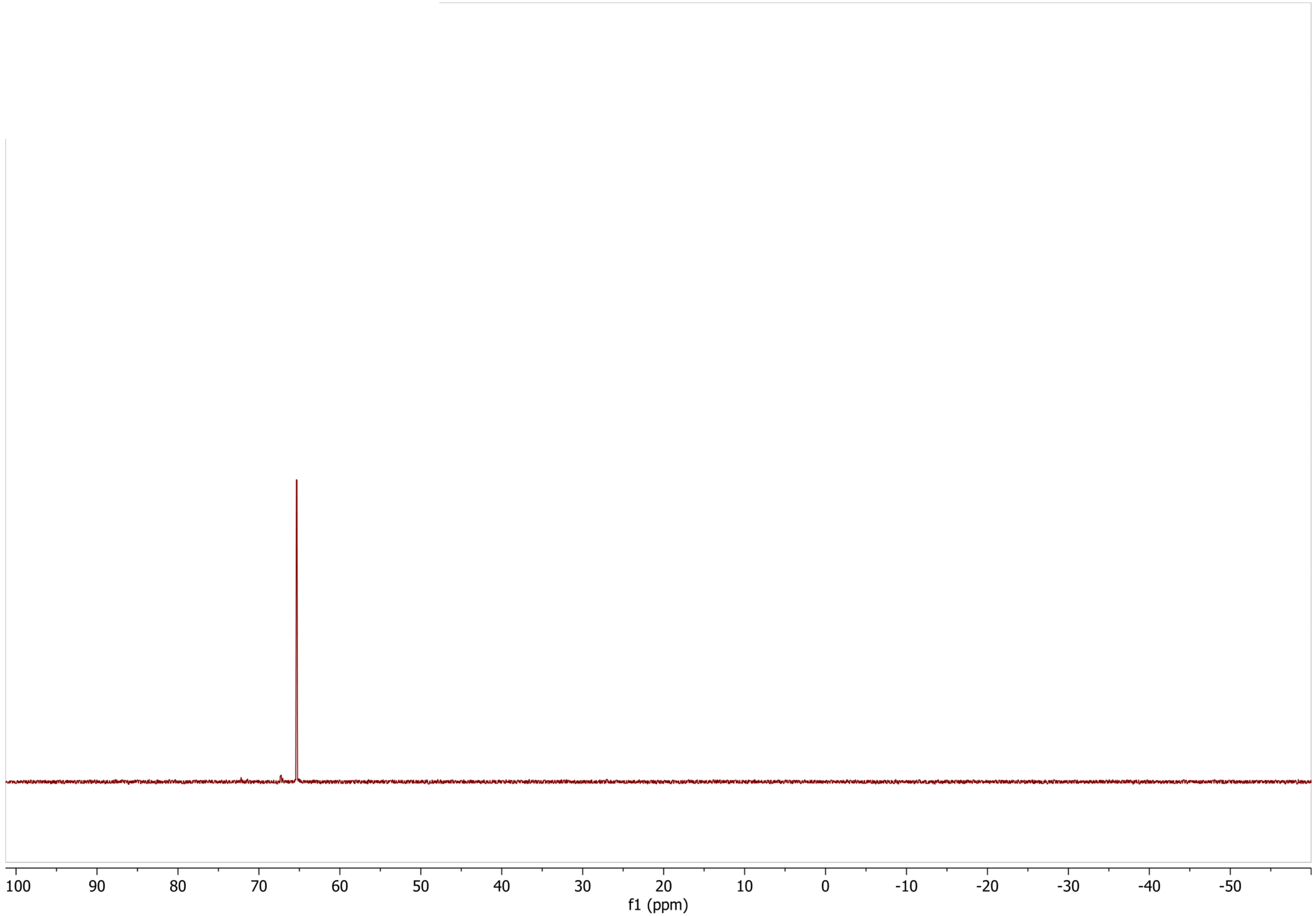

500 MHz ^1H spectrum in $CDCl_3$

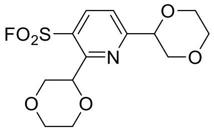

C-10b

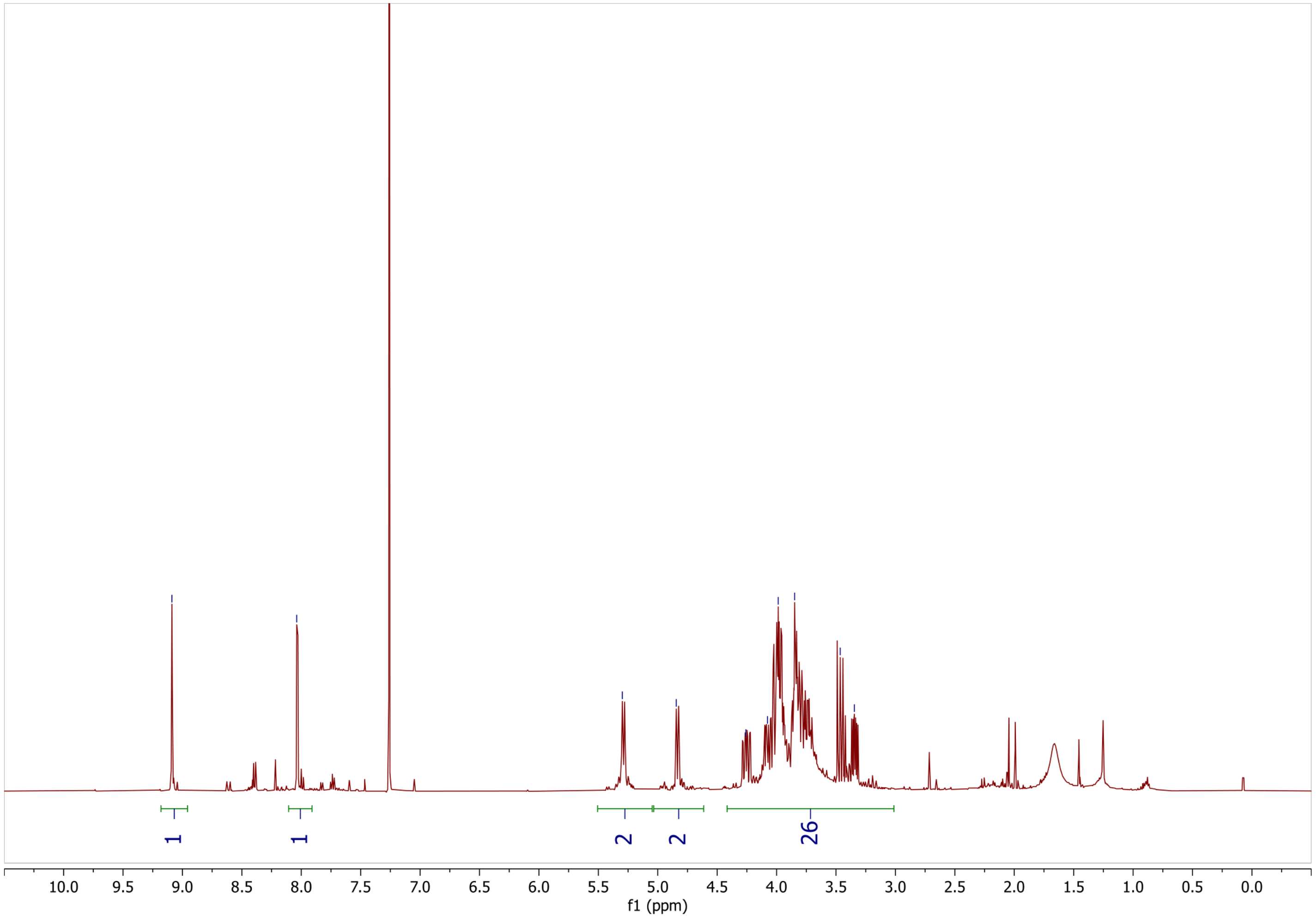

125 MHz ^{13}C spectrum in $CDCl_3$

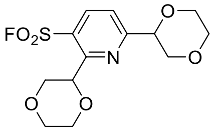

C-10b

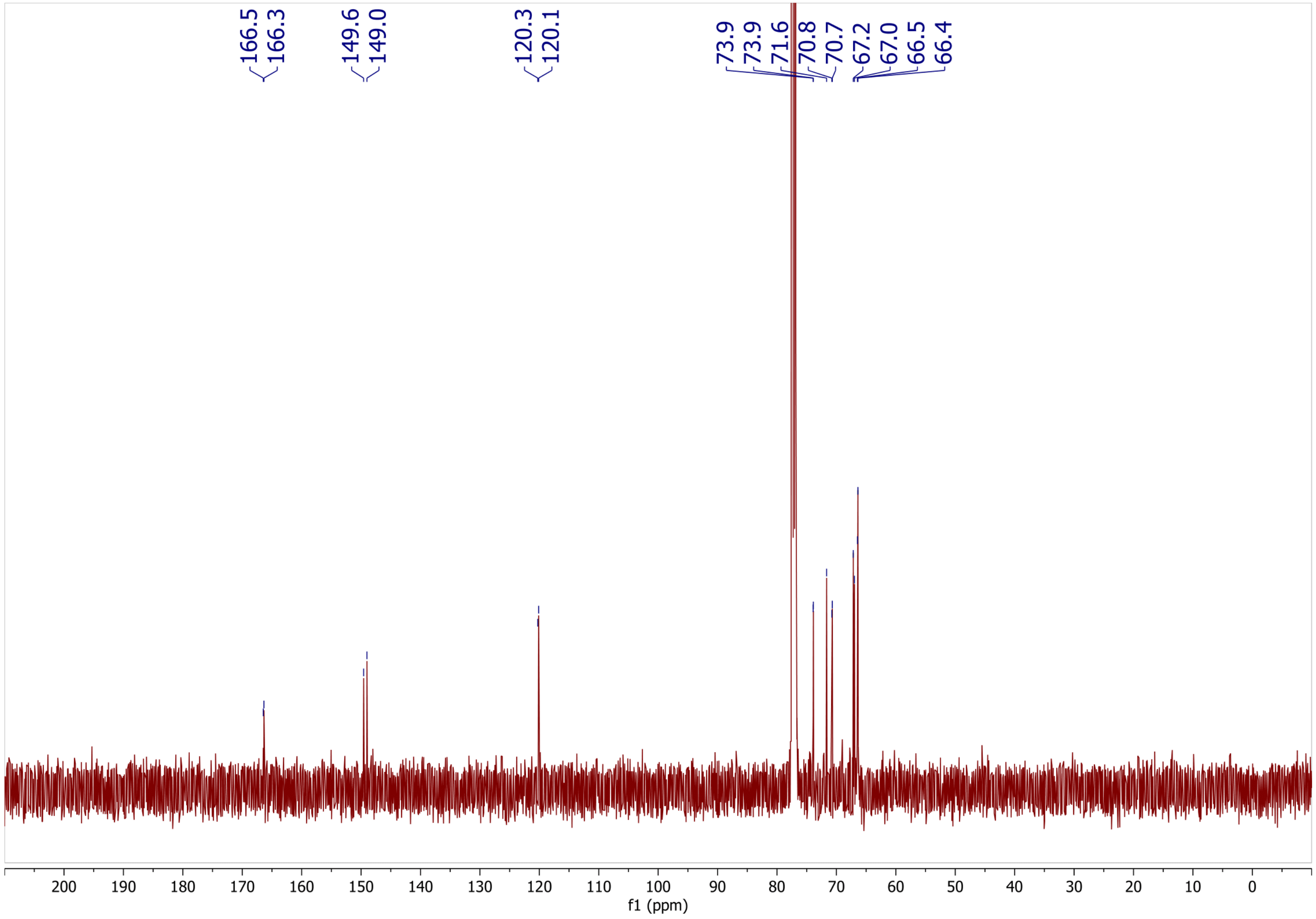

565 MHz ^{19}F spectrum in $CDCl_3$

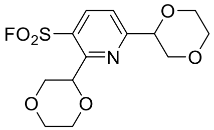

C-10b

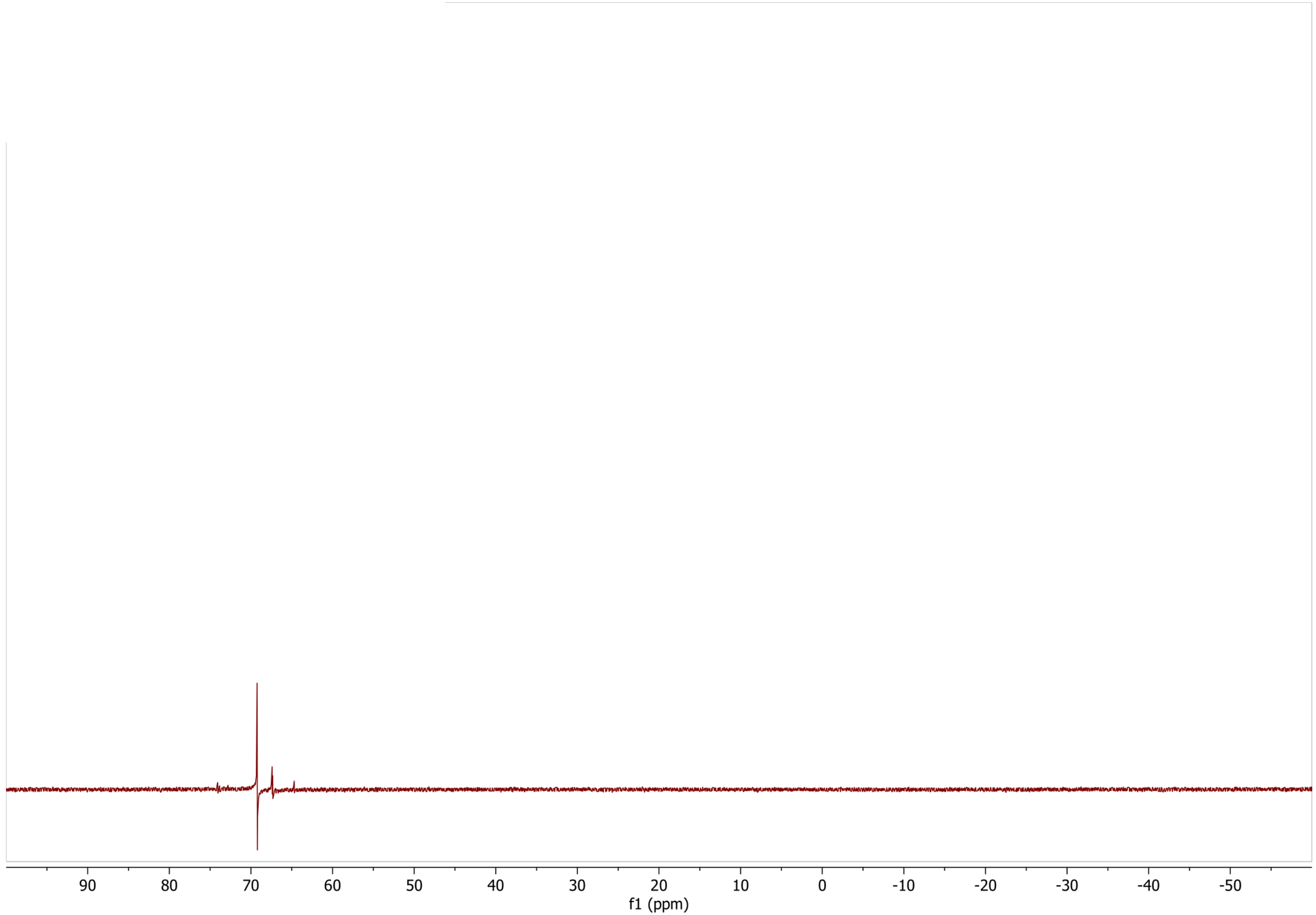

500 MHz ^{1}H spectrum in $CDCl_3$

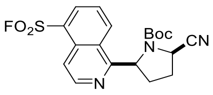

D-3a

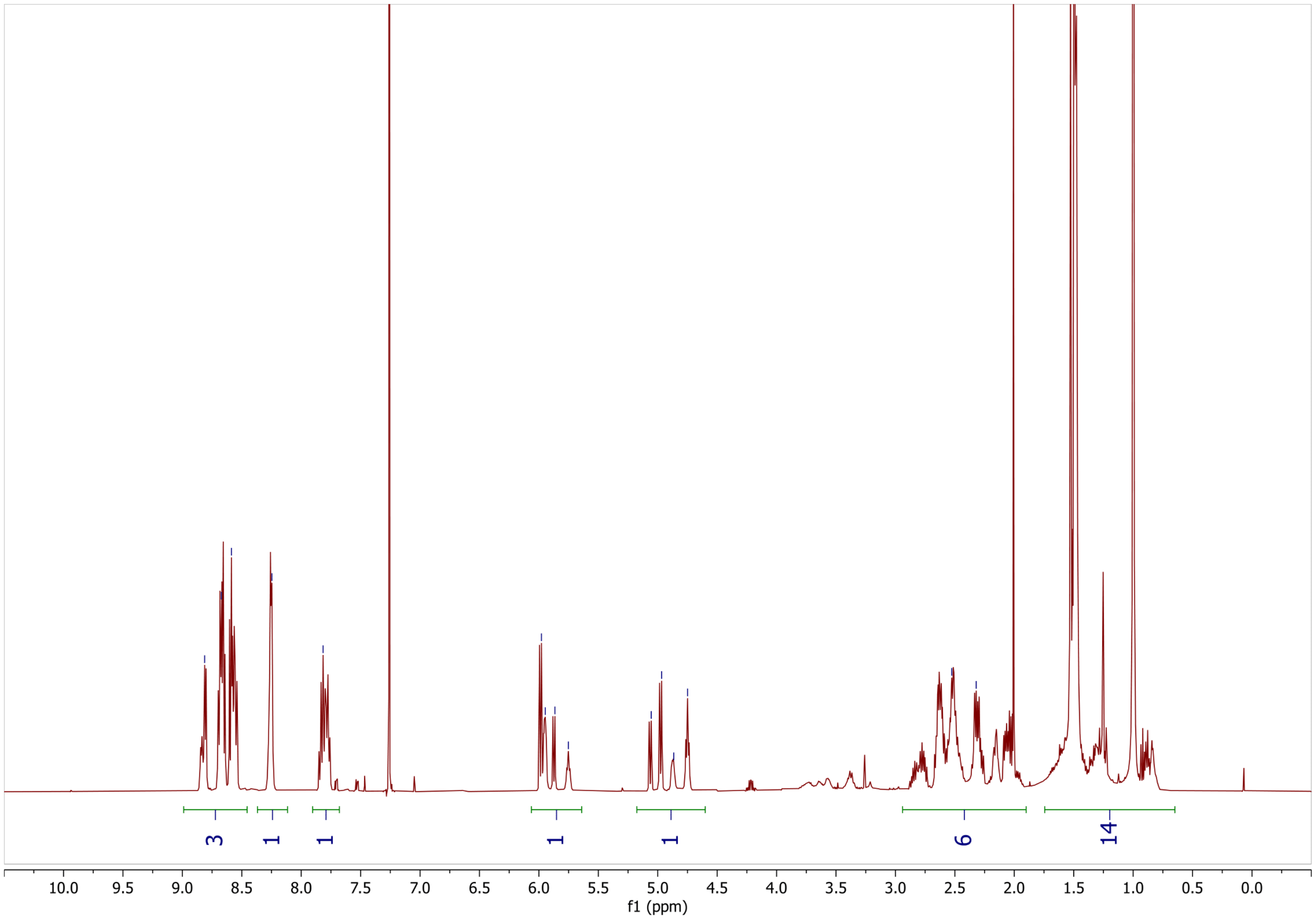

125 MHz ^{13}C spectrum in $CDCl_3$

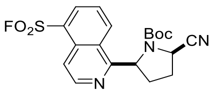

D-3a

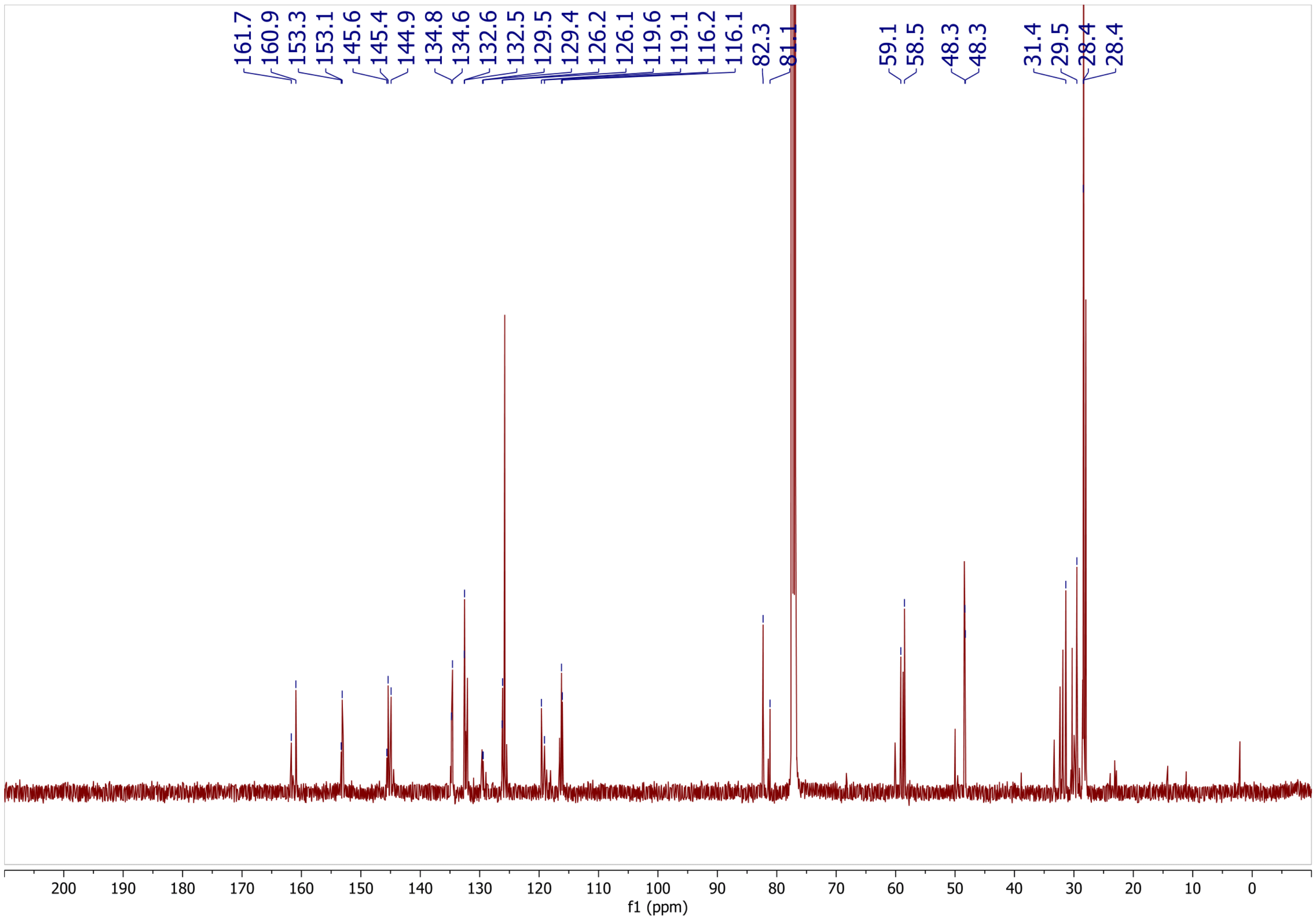

565 MHz ^{19}F spectrum in $CDCl_3$

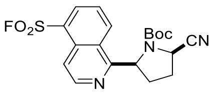

D-3a

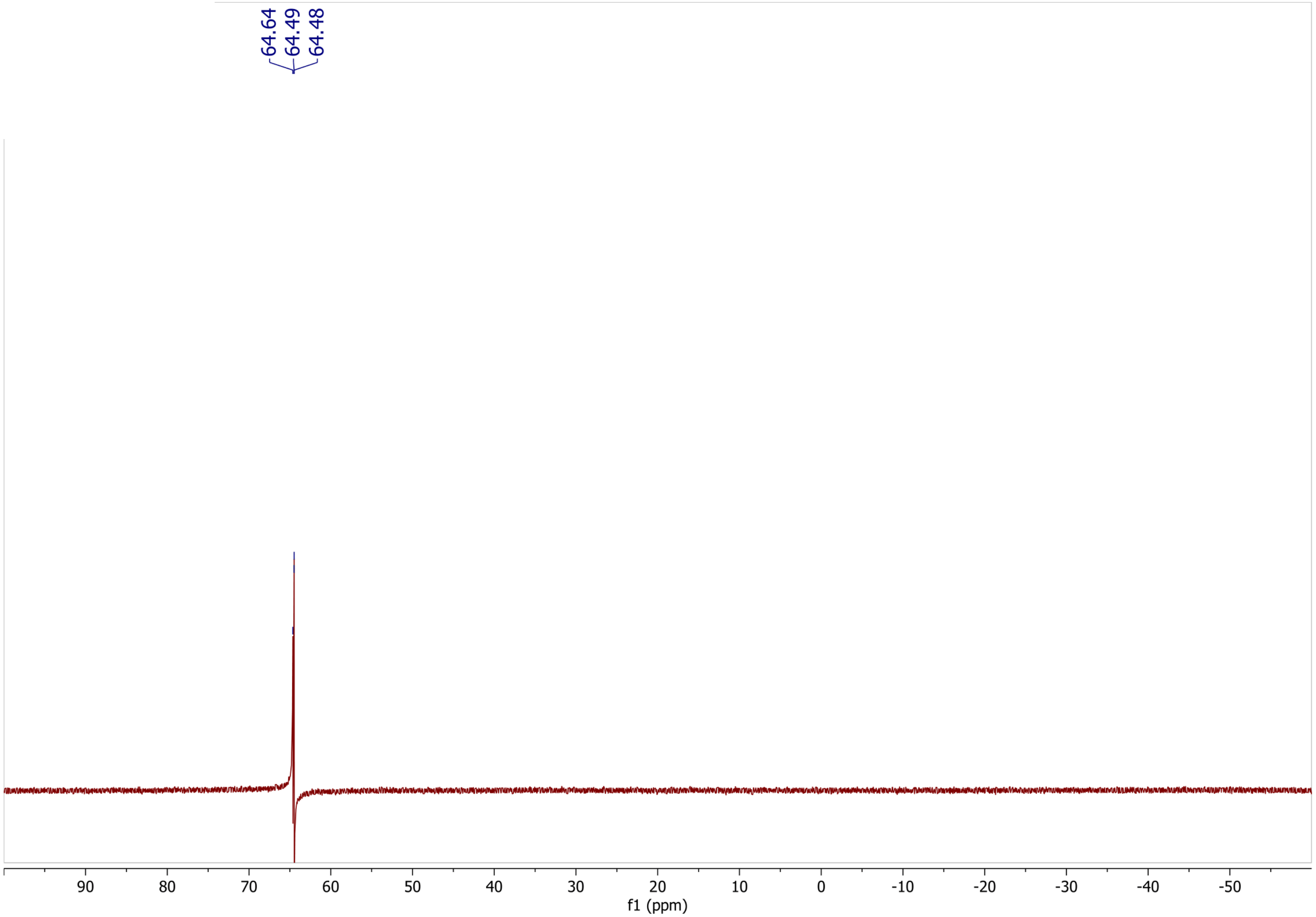

500 MHz ^{1}H spectrum in $CDCl_3$

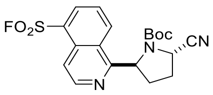

D-3b

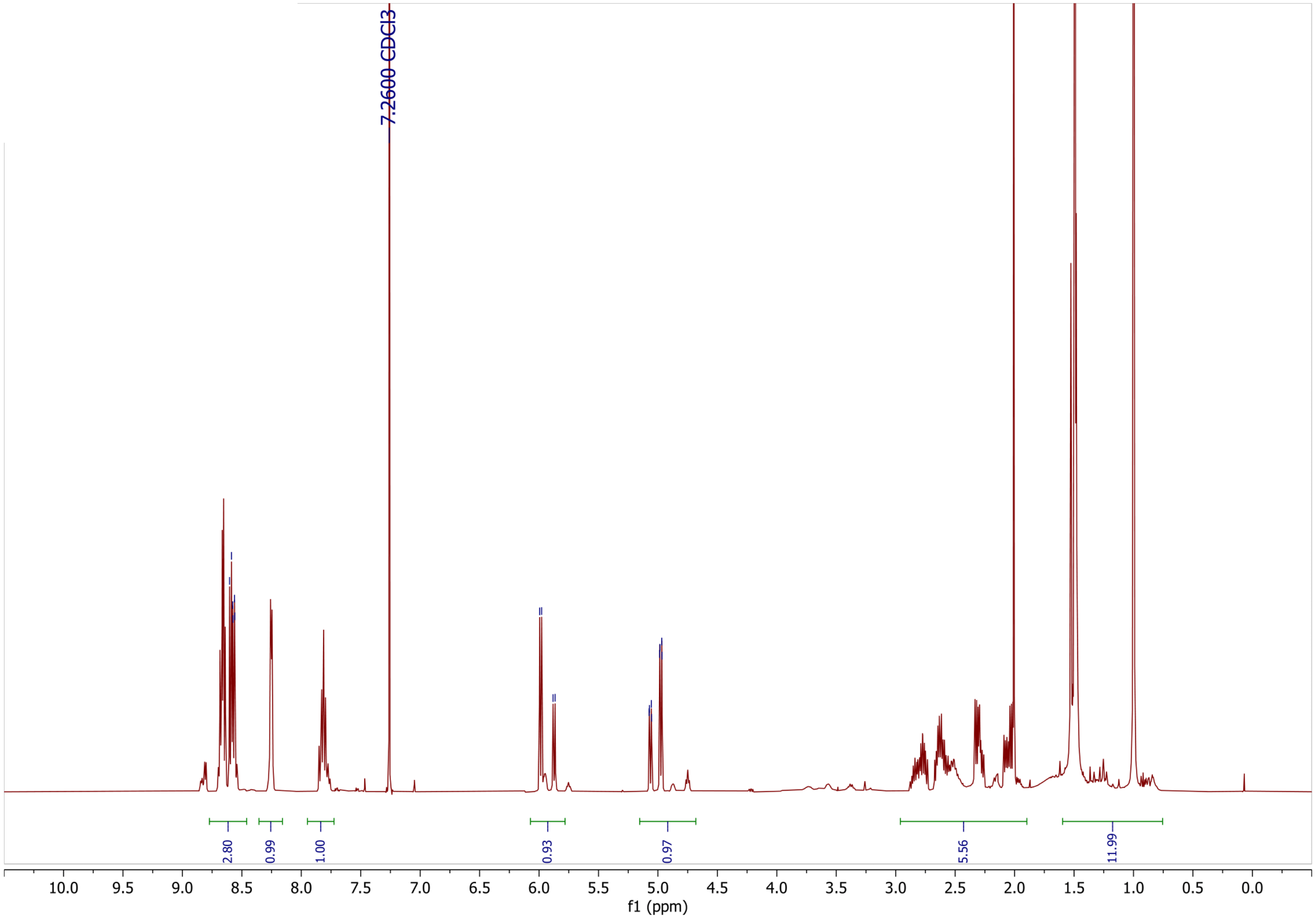

125 MHz ^{13}C spectrum in $CDCl_3$

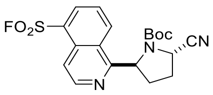

D-3b

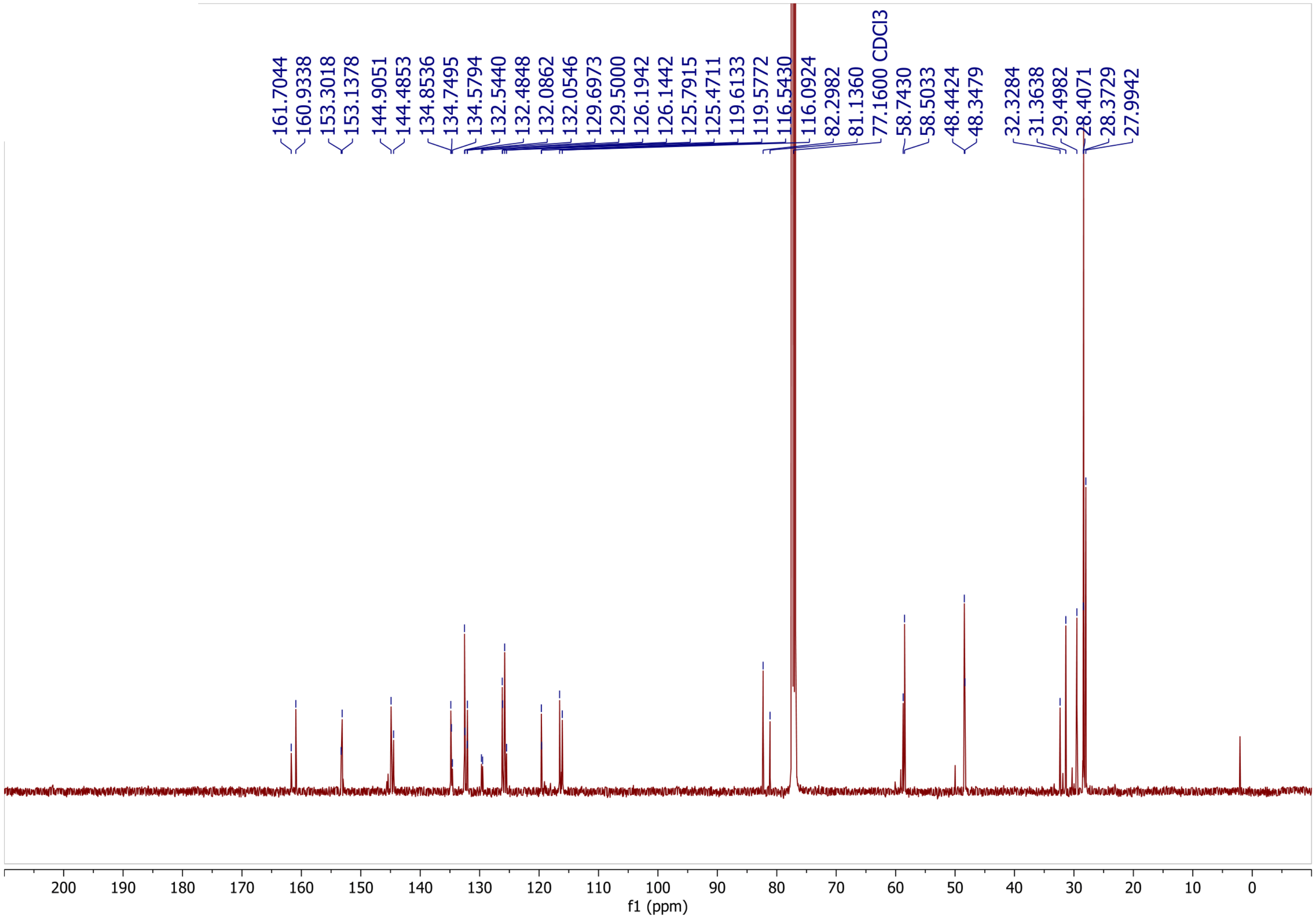

565 MHz ^{19}F spectrum in $CDCl_3$

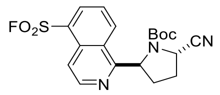

D-3b

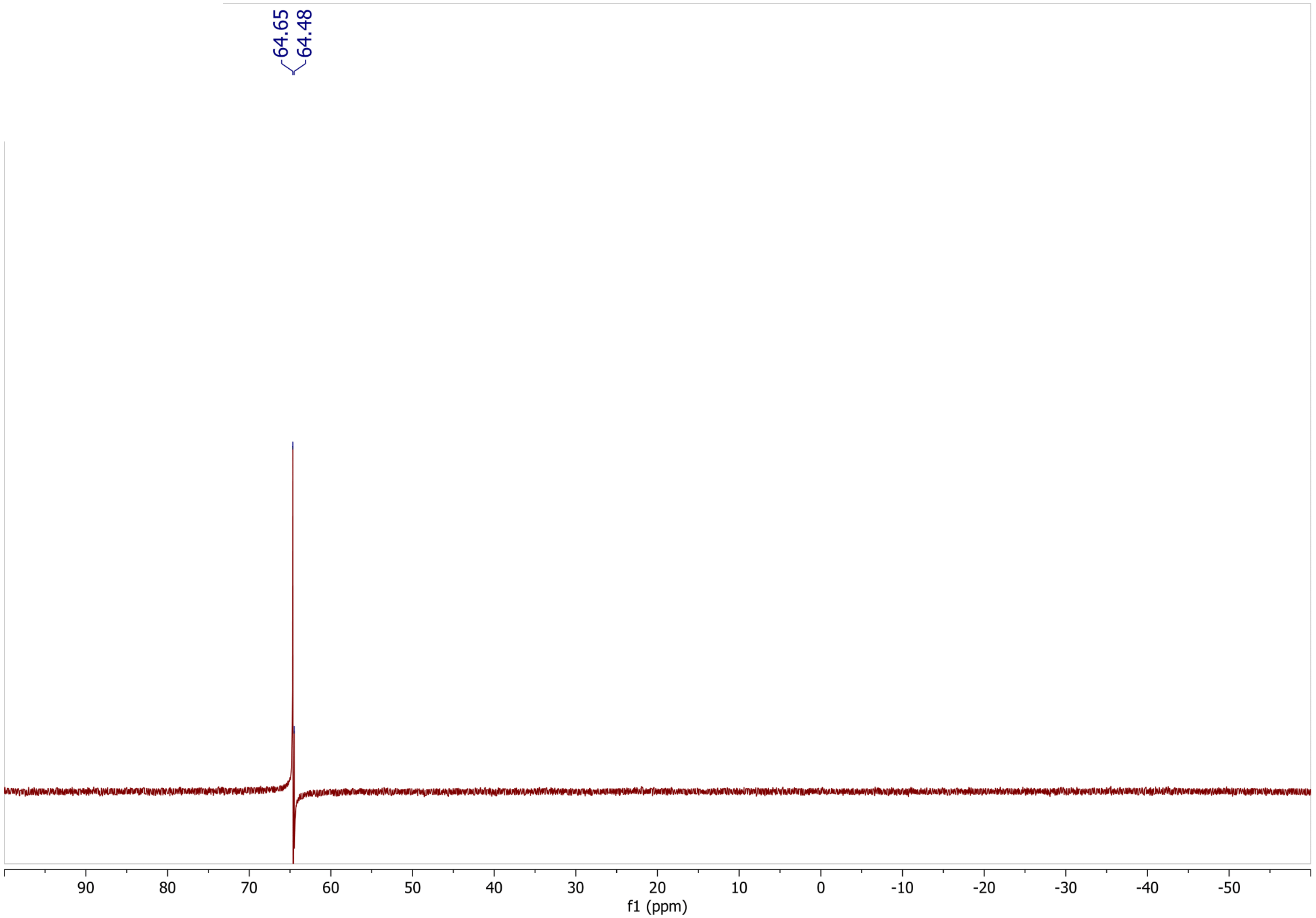

500 MHz ^1H spectrum in $CDCl_3$

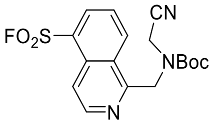

D-4

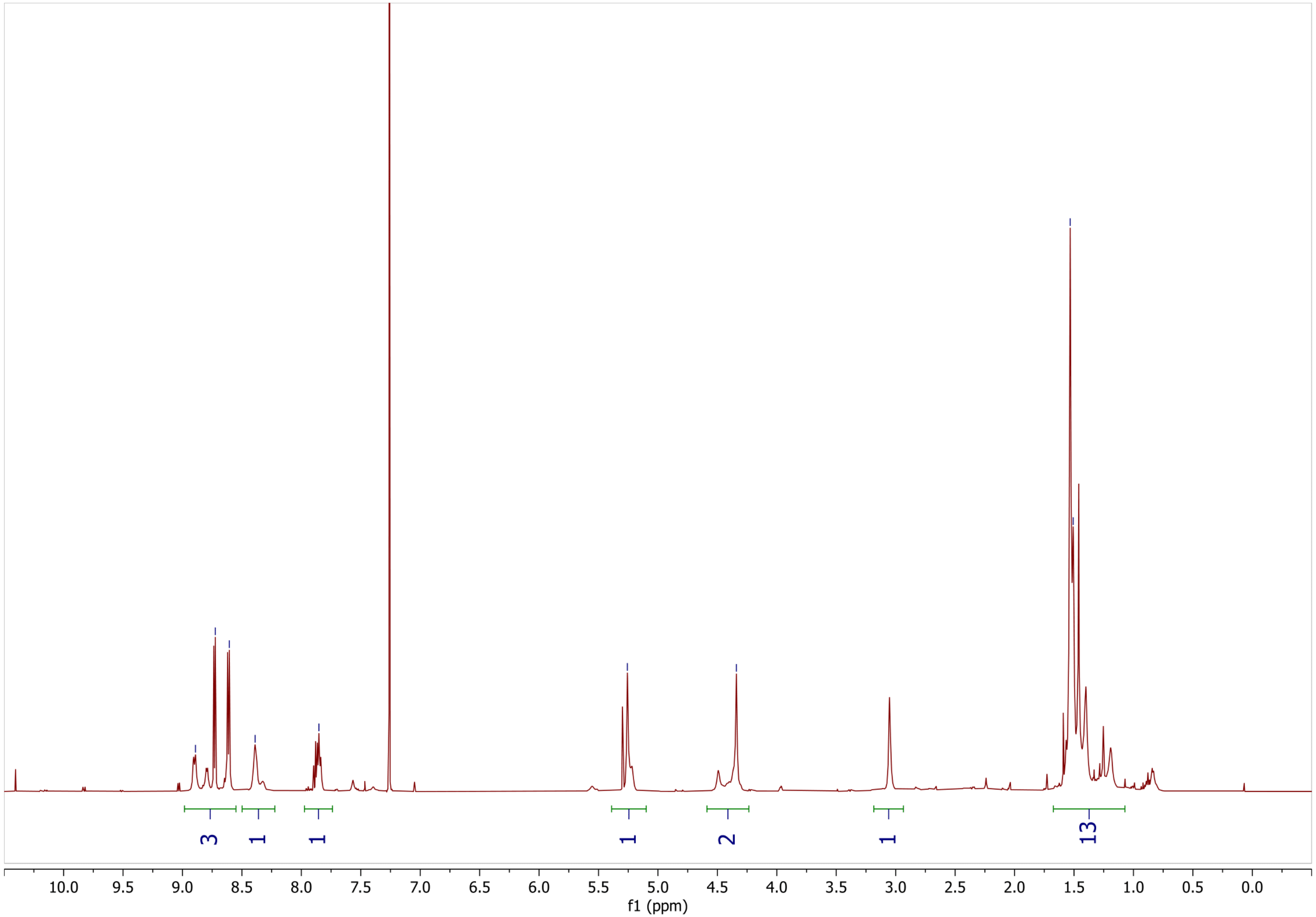

125 MHz ^{13}C spectrum in $CDCl_3$

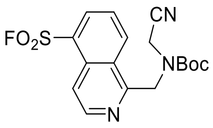

D-4

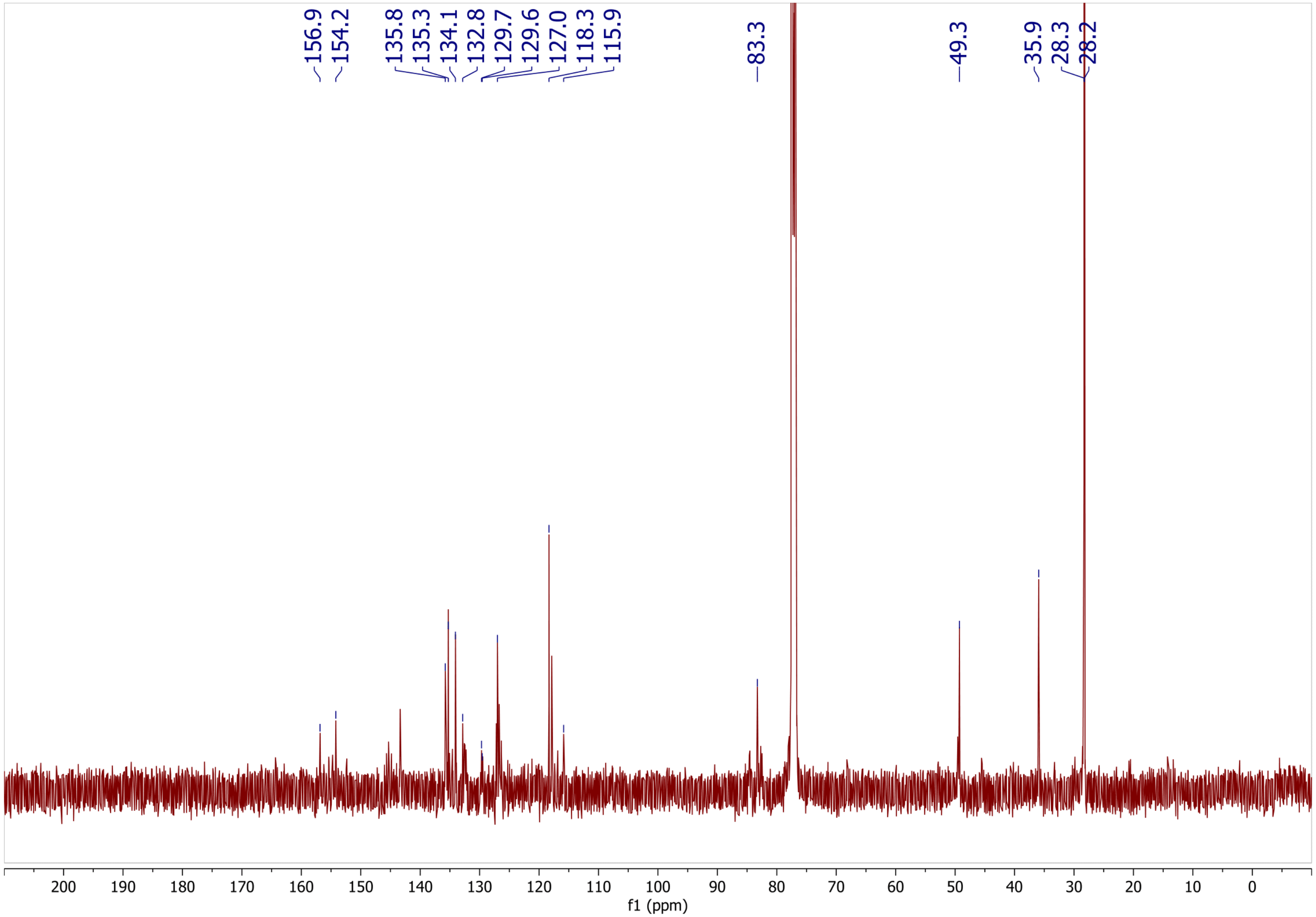

500 MHz ^{1}H spectrum in $CDCl_3$

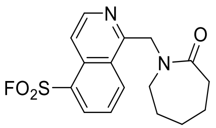

D-8

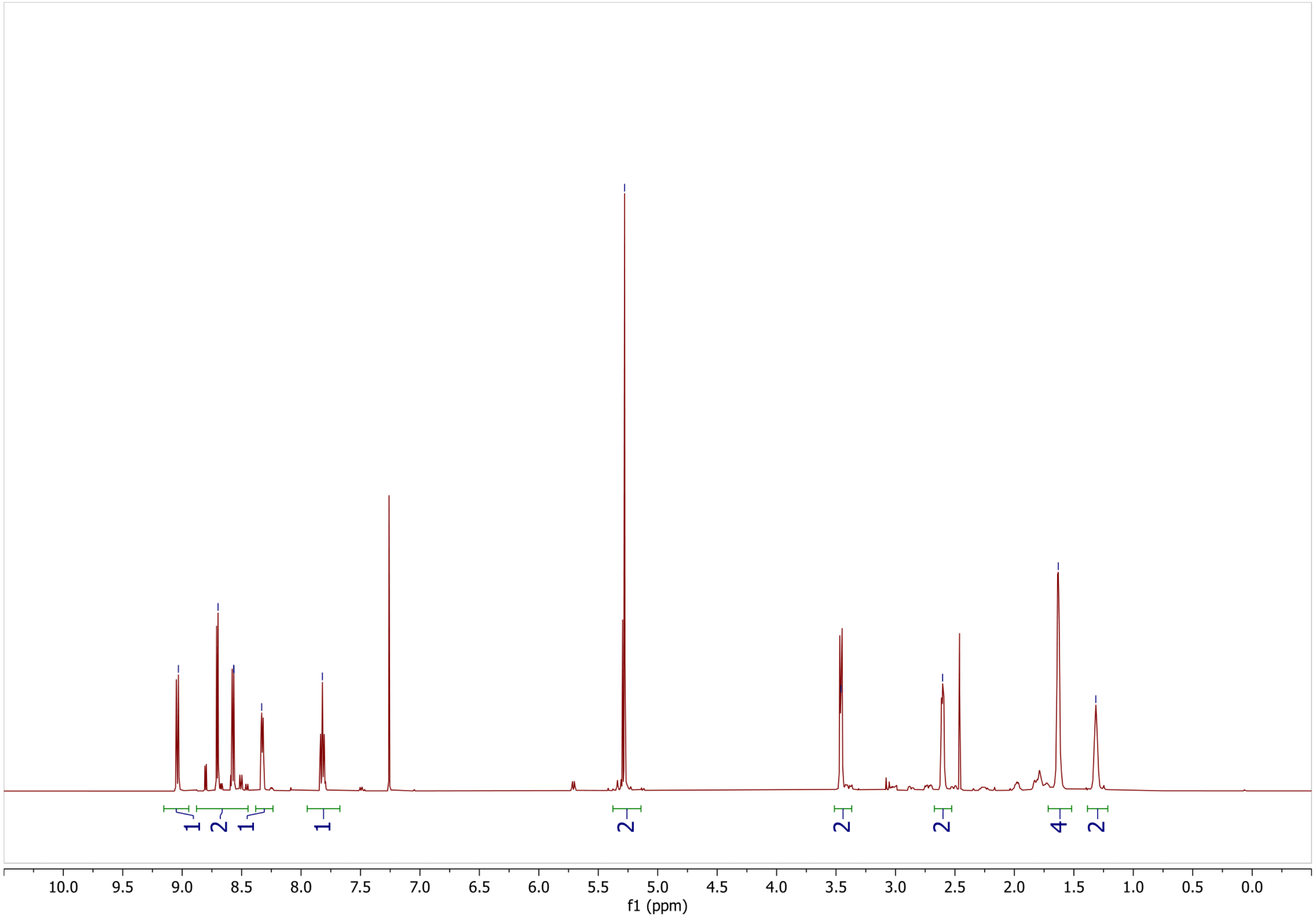

125 MHz ^{13}C spectrum in $CDCl_3$

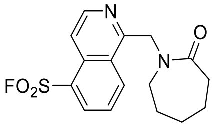

D-8

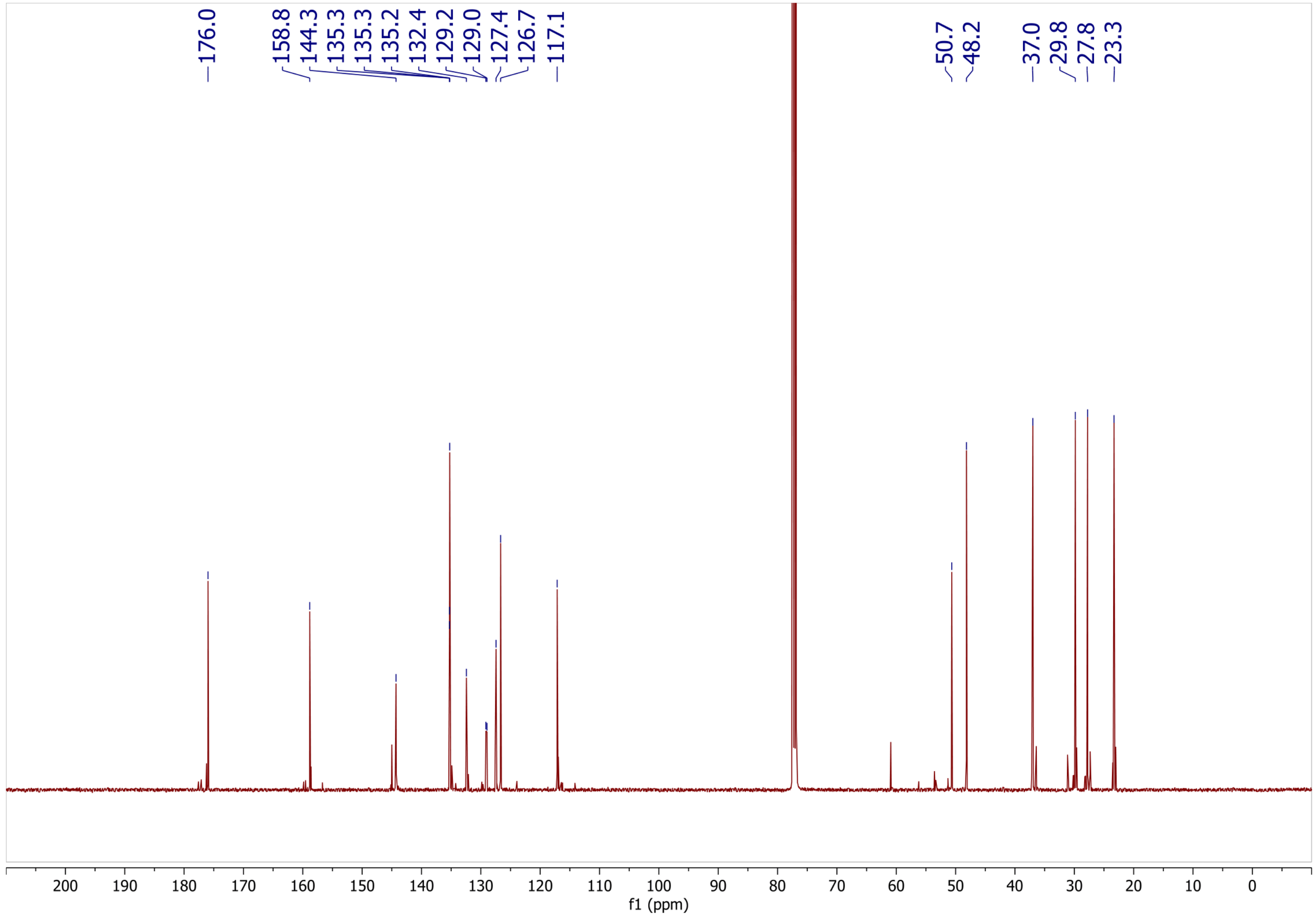

565 MHz ^{19}F spectrum in $CDCl_3$

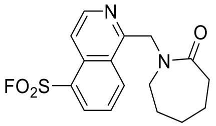

D-8

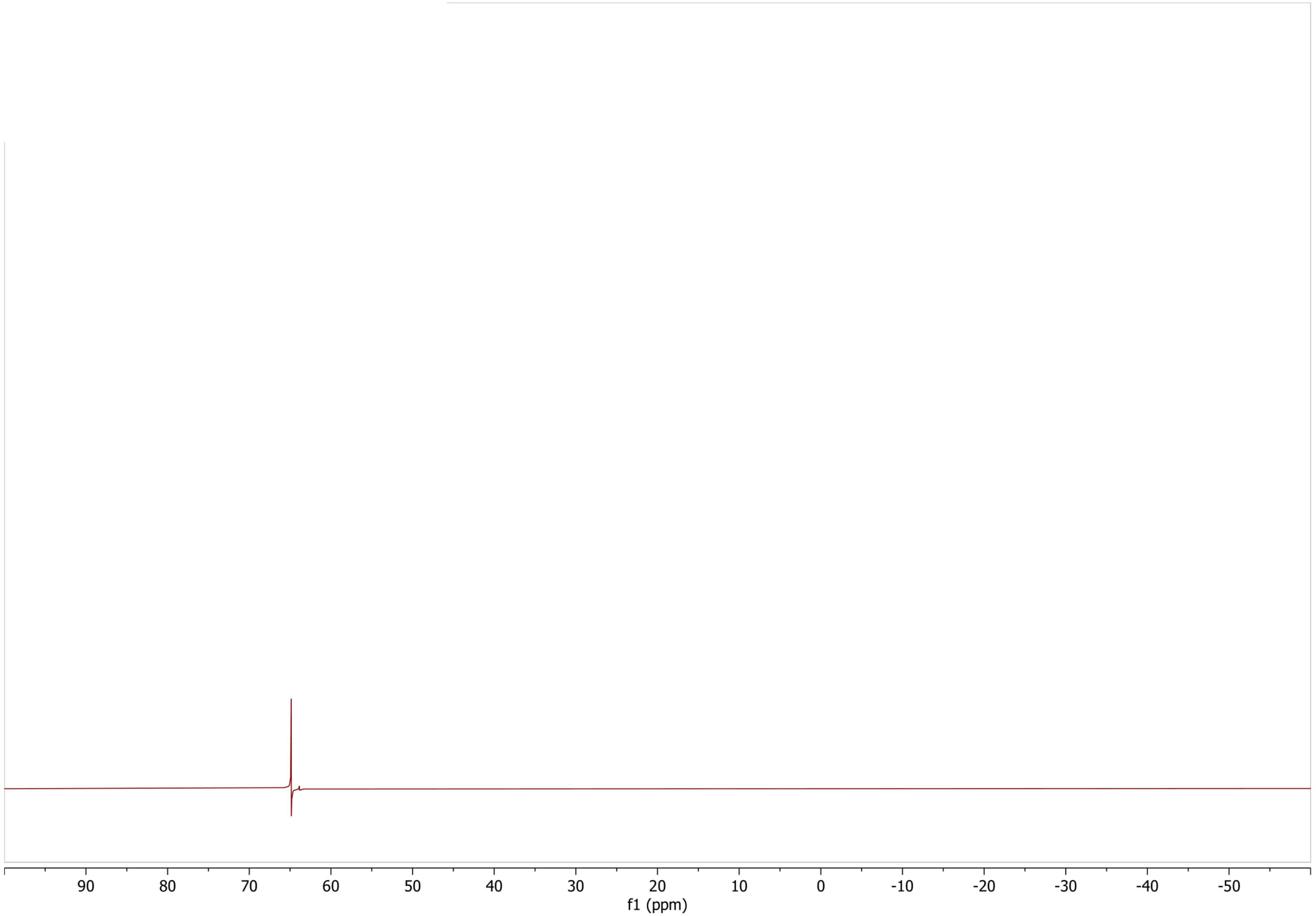

500 MHz ^{1}H spectrum in $CDCl_3$

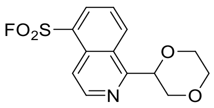

D-10a

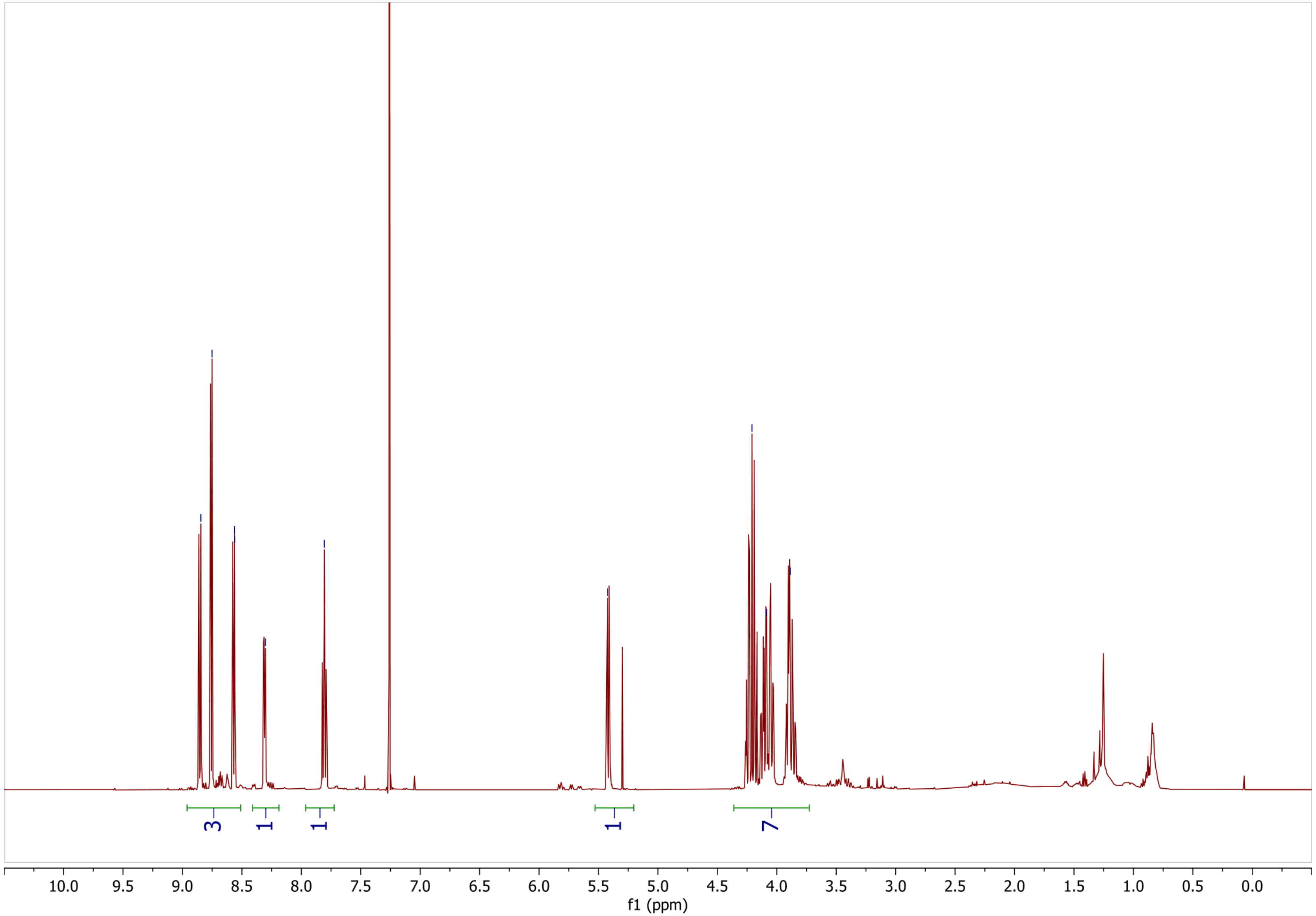

125 MHz ^{13}C spectrum in $CDCl_3$

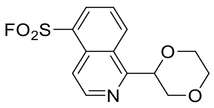

D-10a

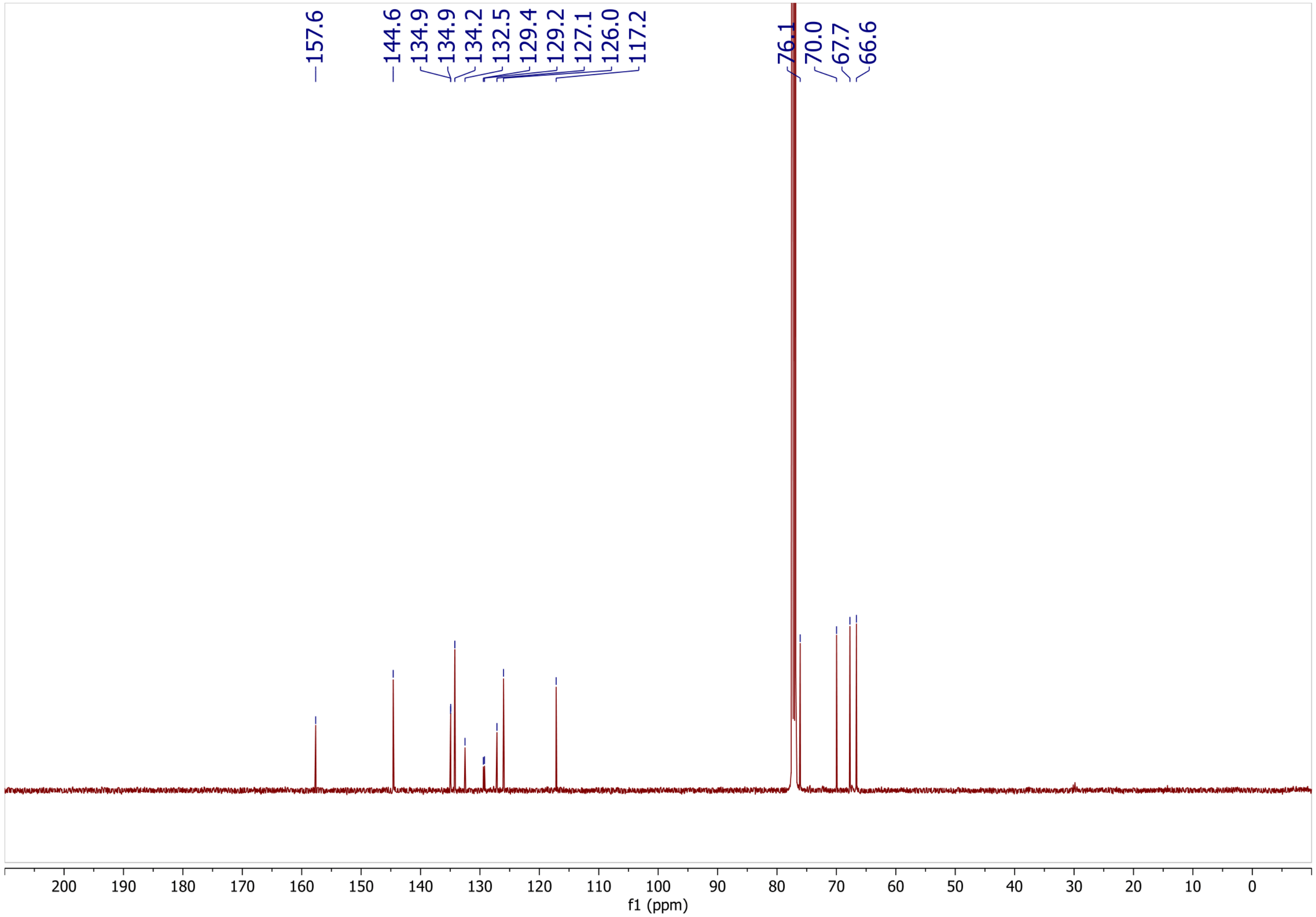

565 MHz ^{19}F spectrum in $CDCl_3$

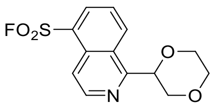

D-10a

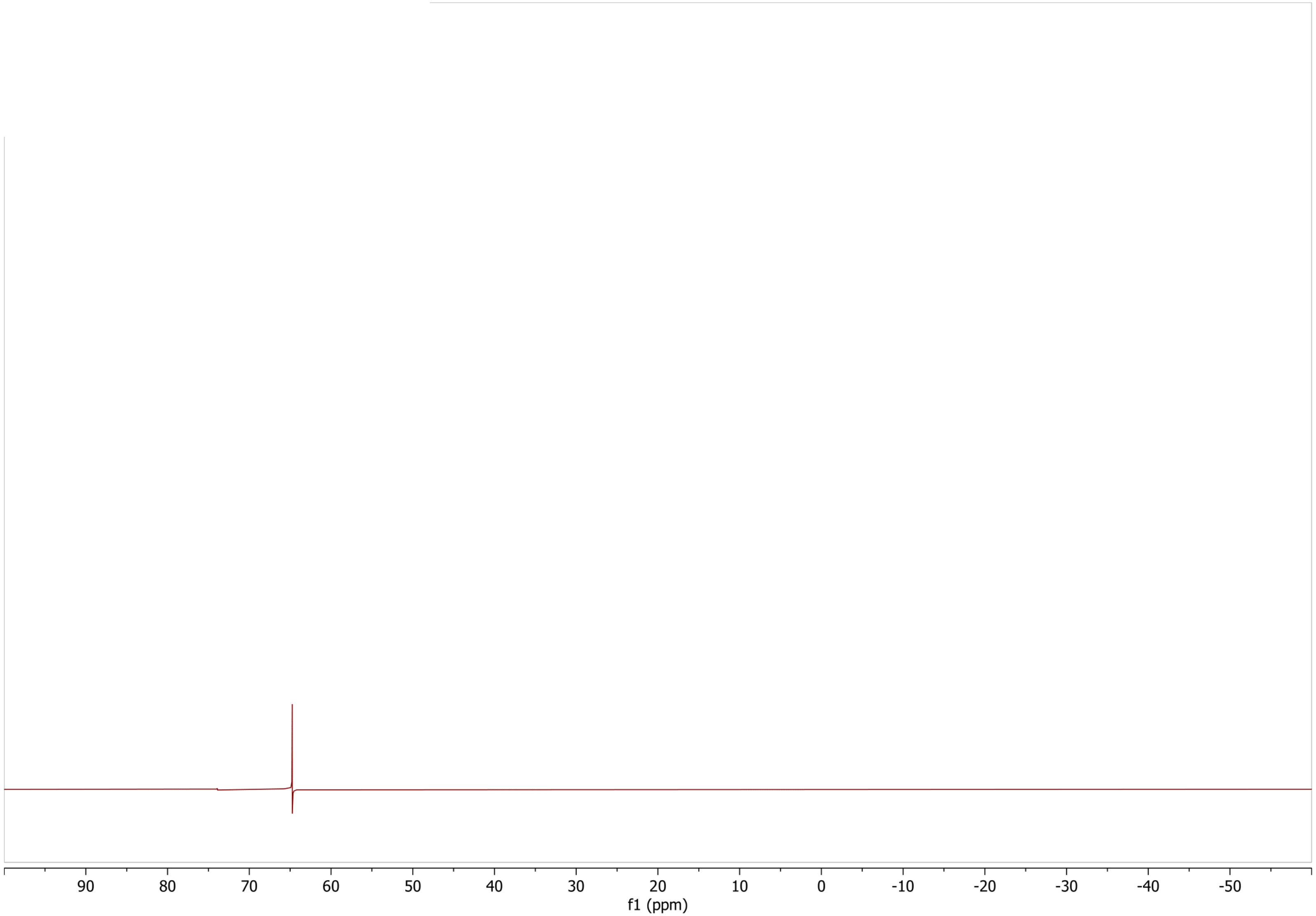

500 MHz ^1H spectrum in $CDCl_3$

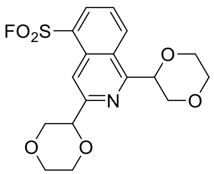

D-10b

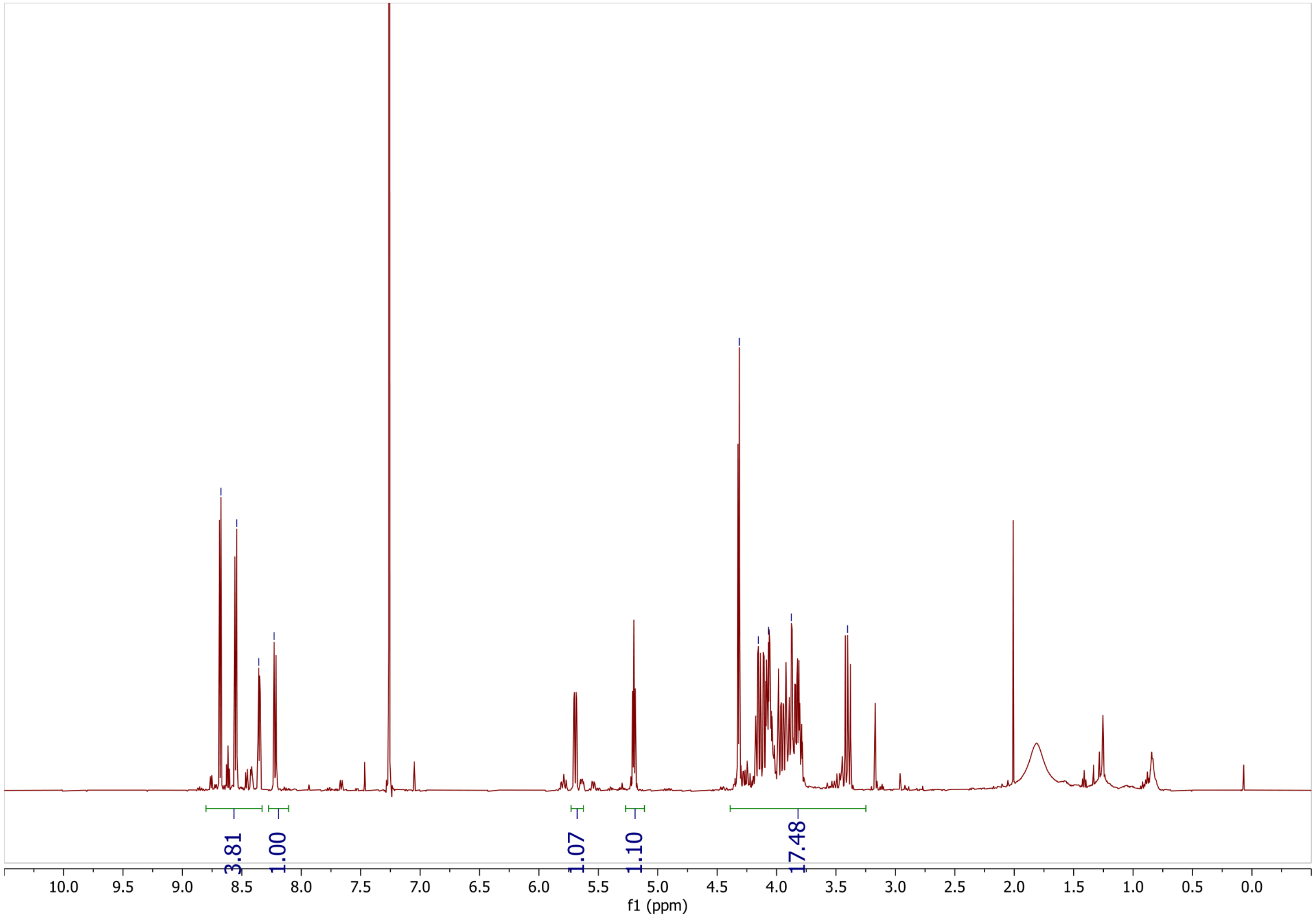

125 MHz ^{13}C spectrum in $CDCl_3$

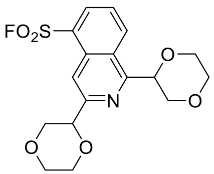

D-10b

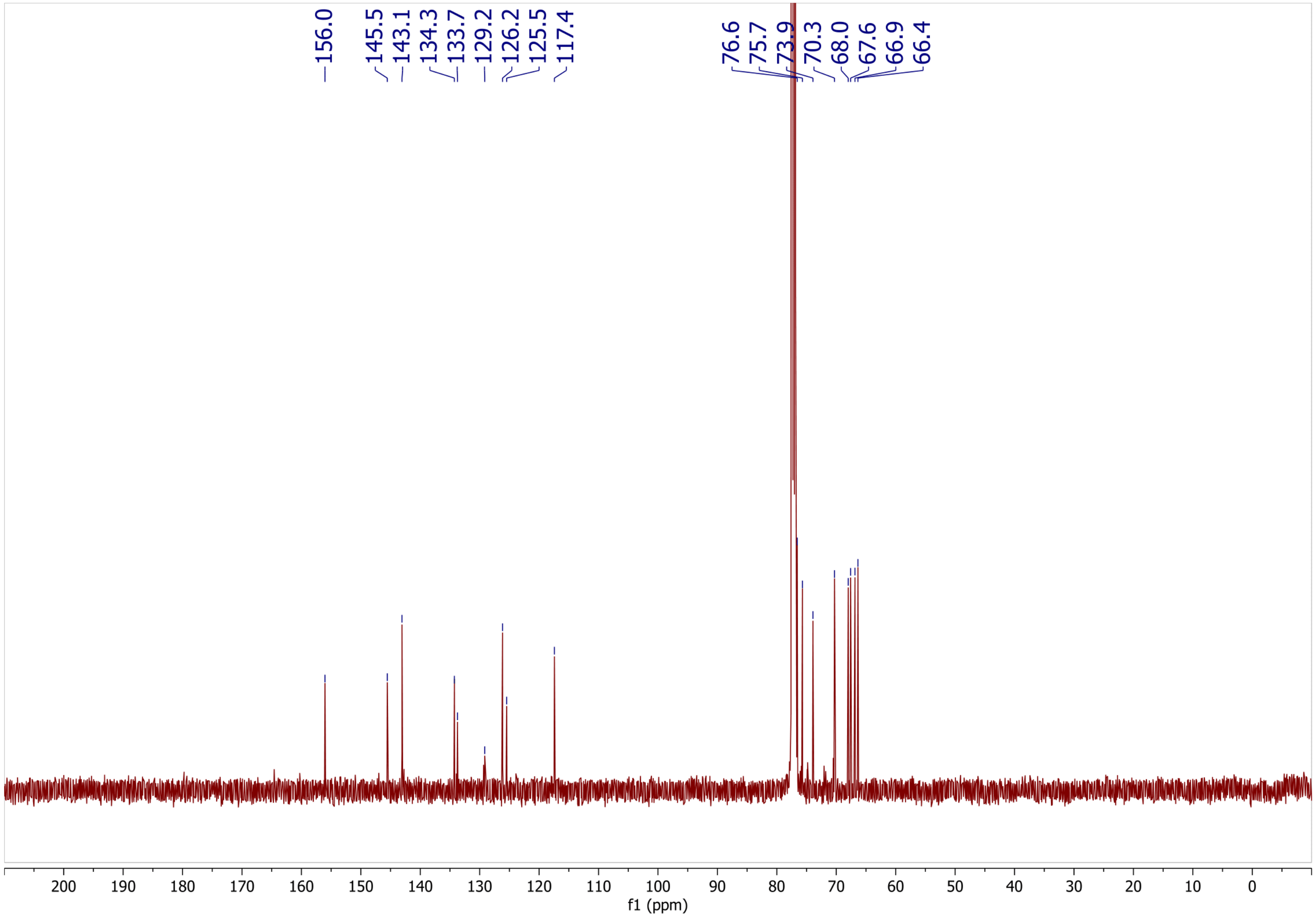

565 MHz ^{19}F spectrum in $CDCl_3$

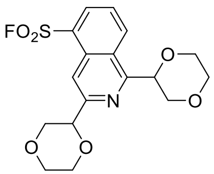

D-10b

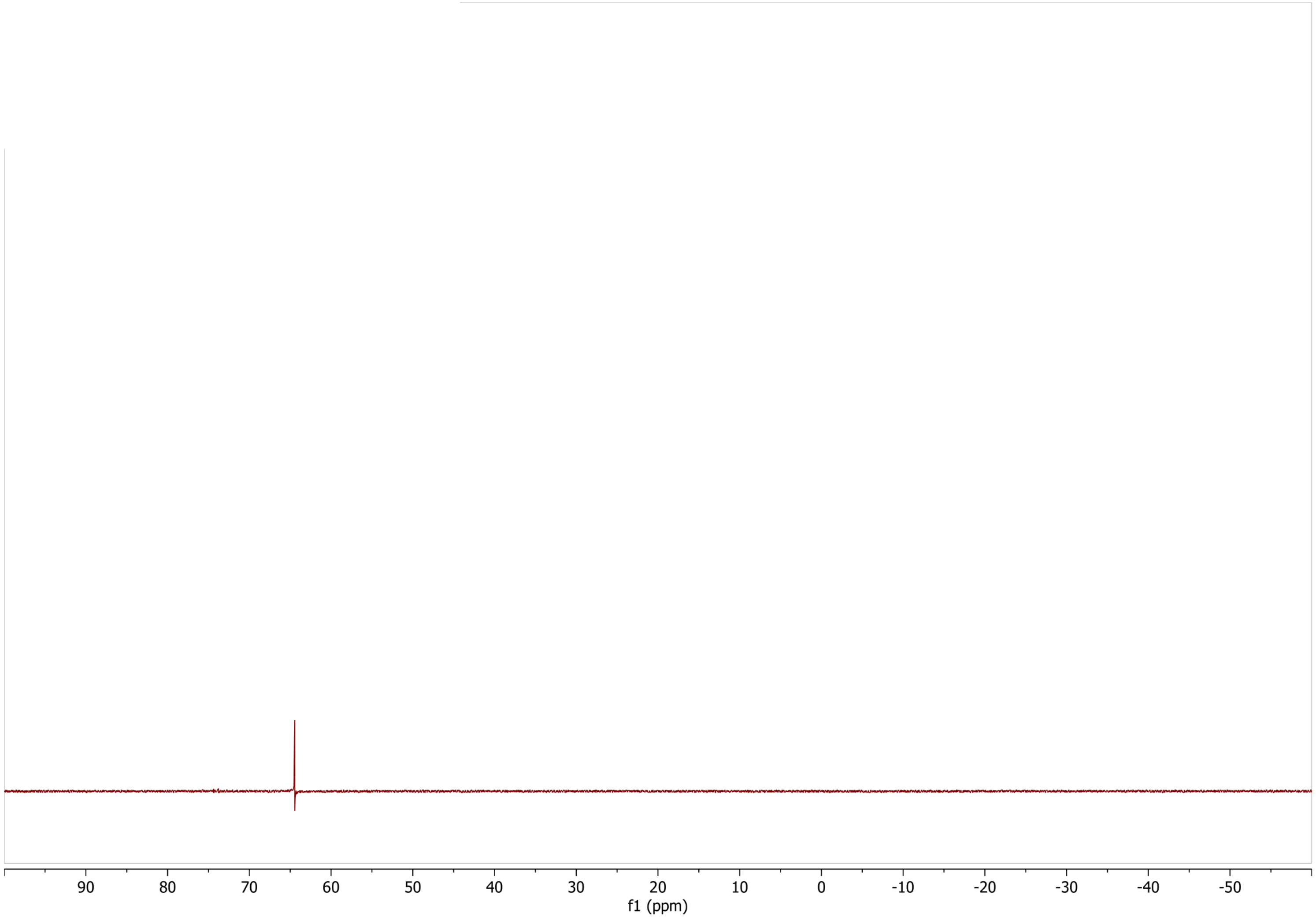

500 MHz ^{1}H spectrum in $CDCl_3$

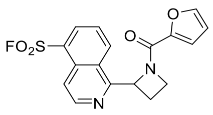

D-18

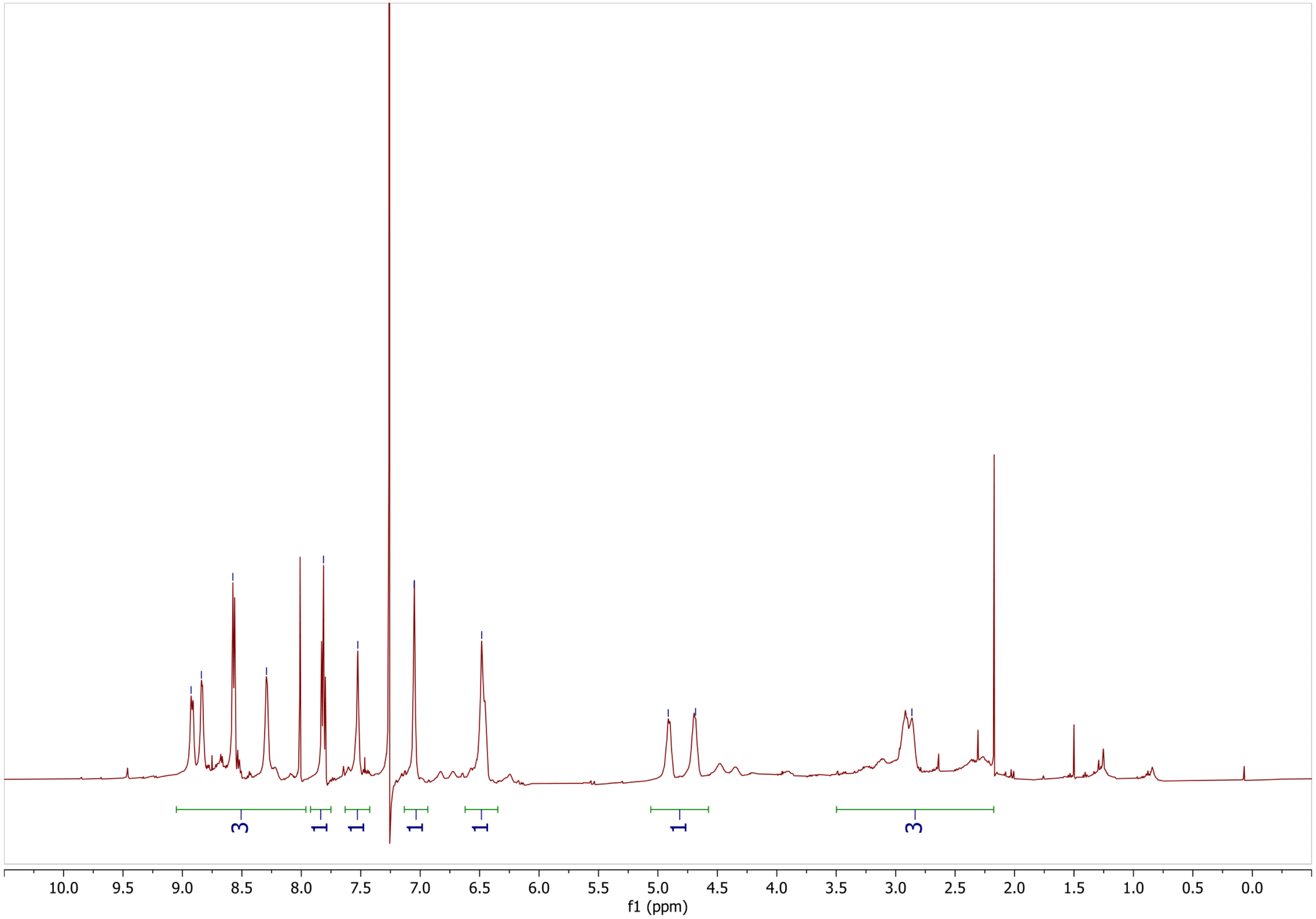

125 MHz ^{13}C spectrum in $CDCl_3$

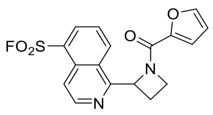

D-18

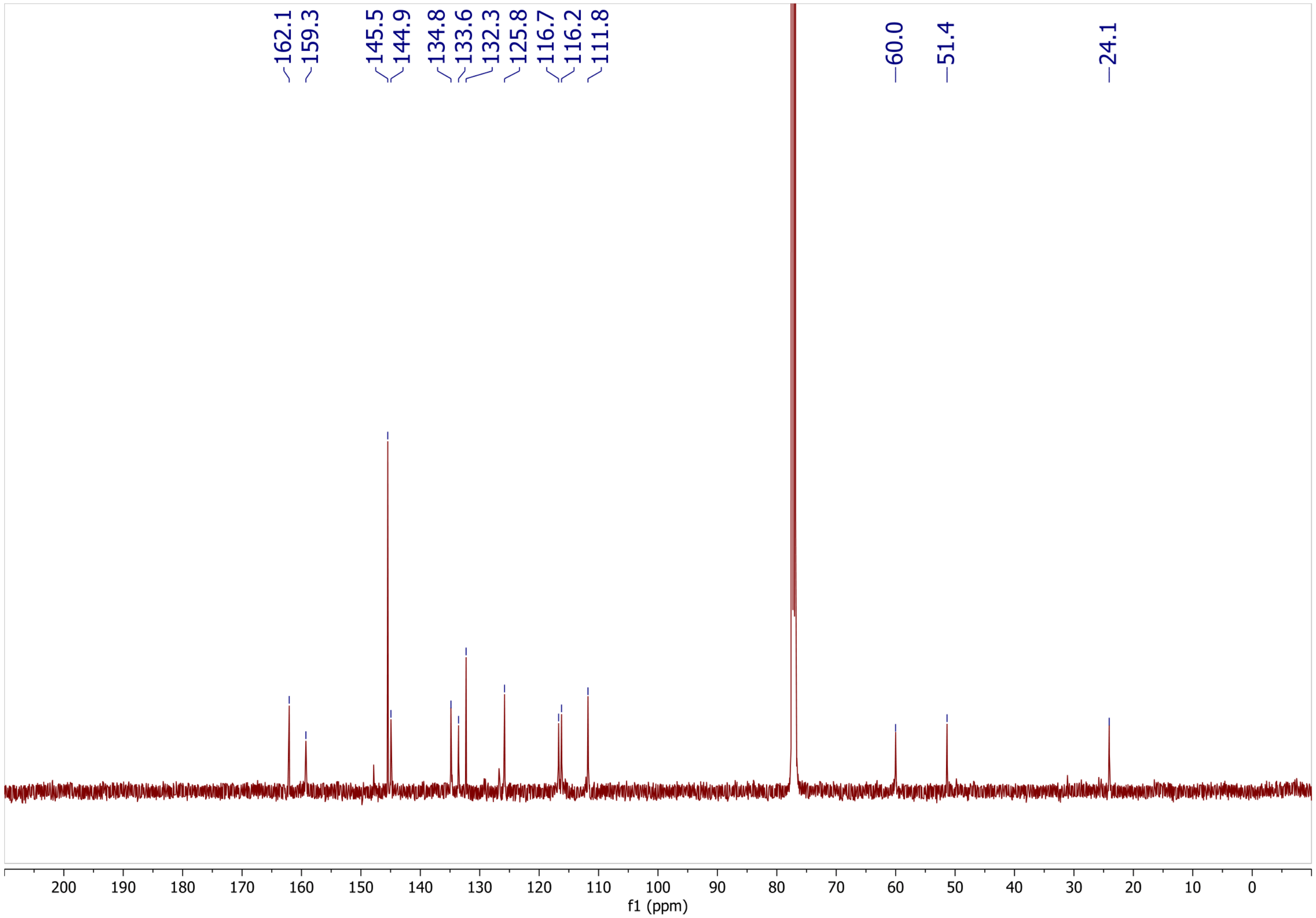

565 MHz ^{19}F spectrum in $CDCl_3$

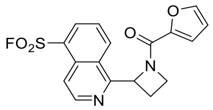

D-18

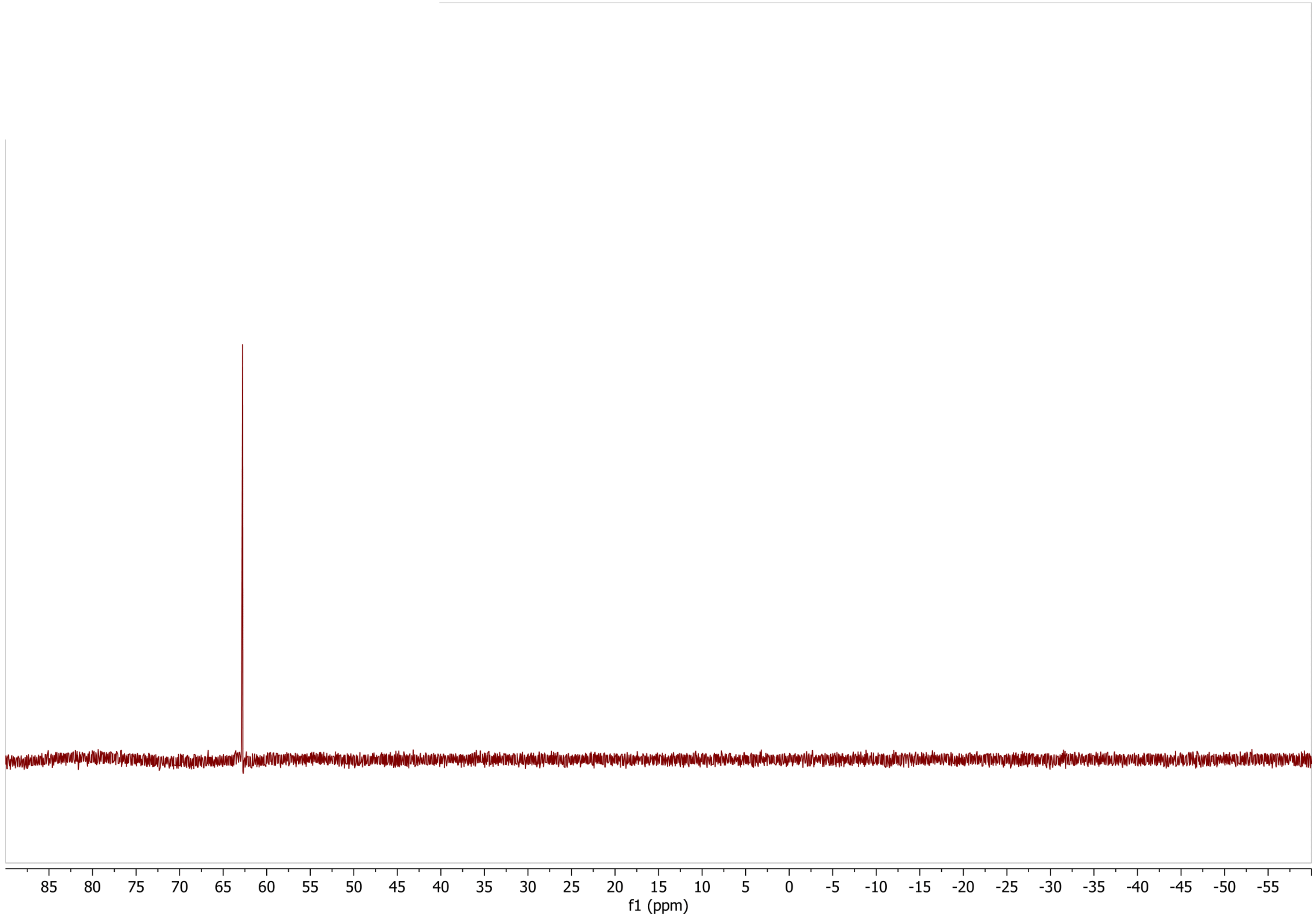

500 MHz ^{1}H spectrum in $CDCl_3$

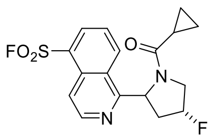

D-19b

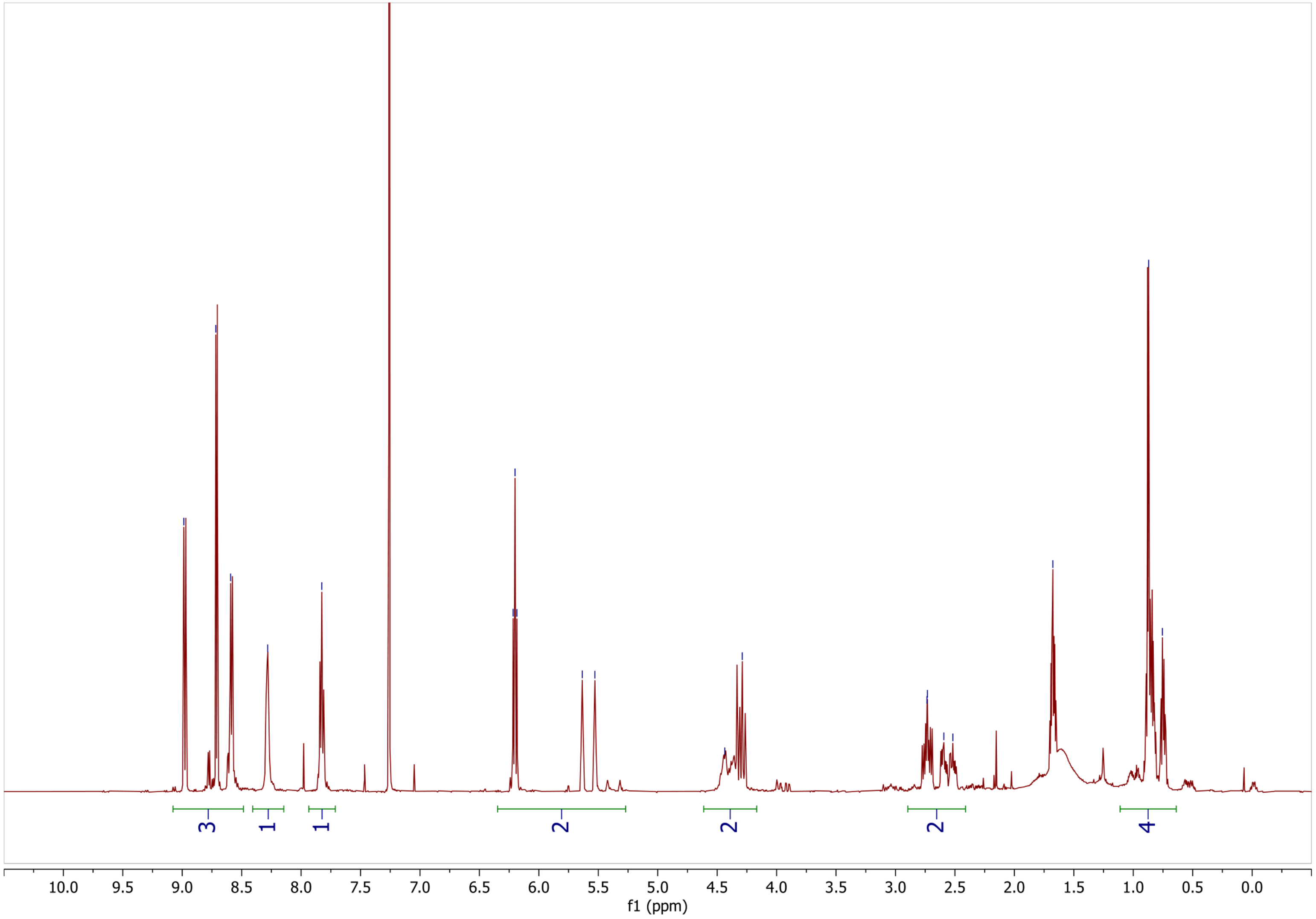

125 MHz ^{13}C spectrum in $CDCl_3$

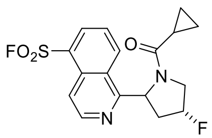

D-19b

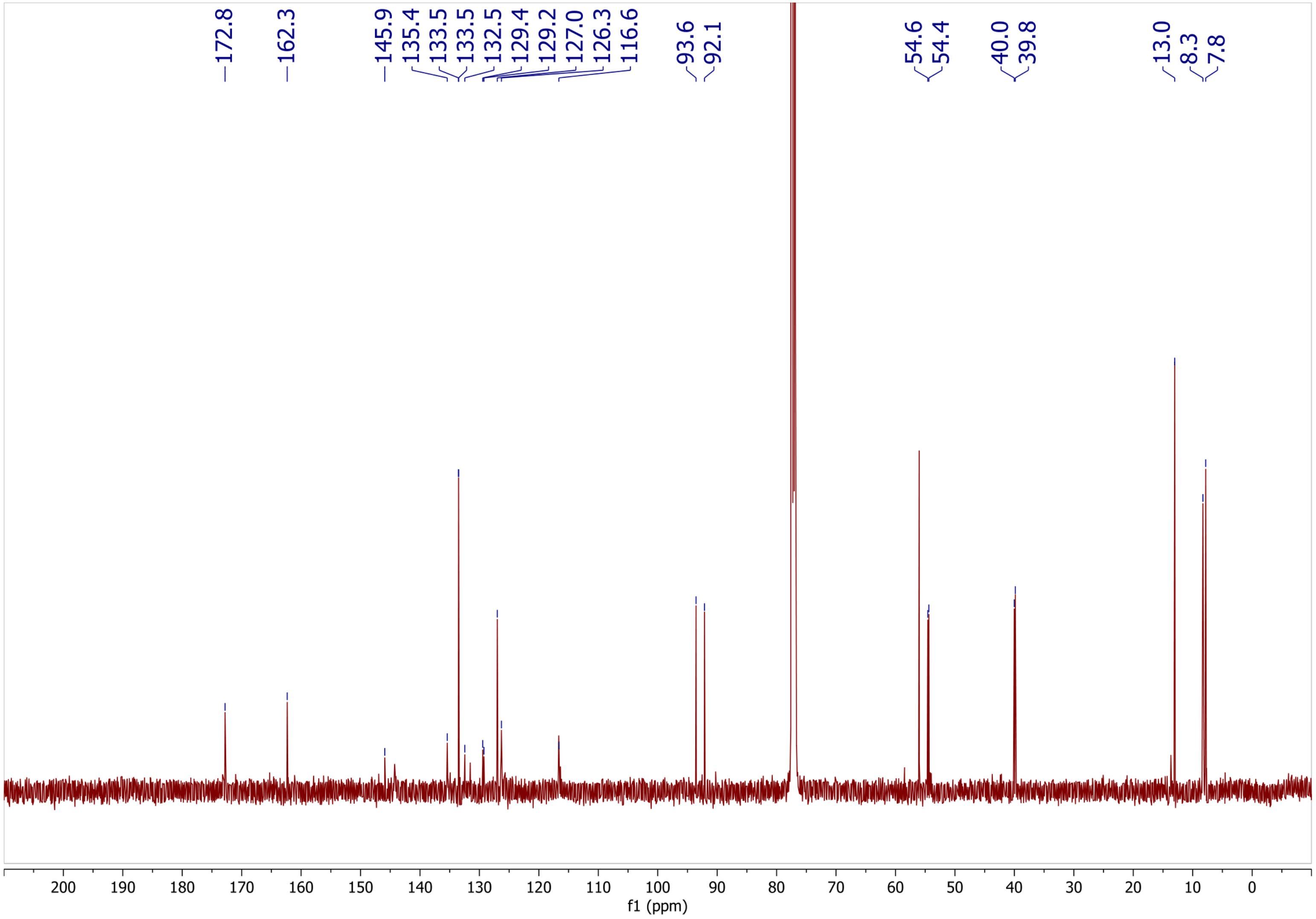

565 MHz ^{19}F spectrum in $CDCl_3$

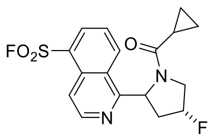

D-19b

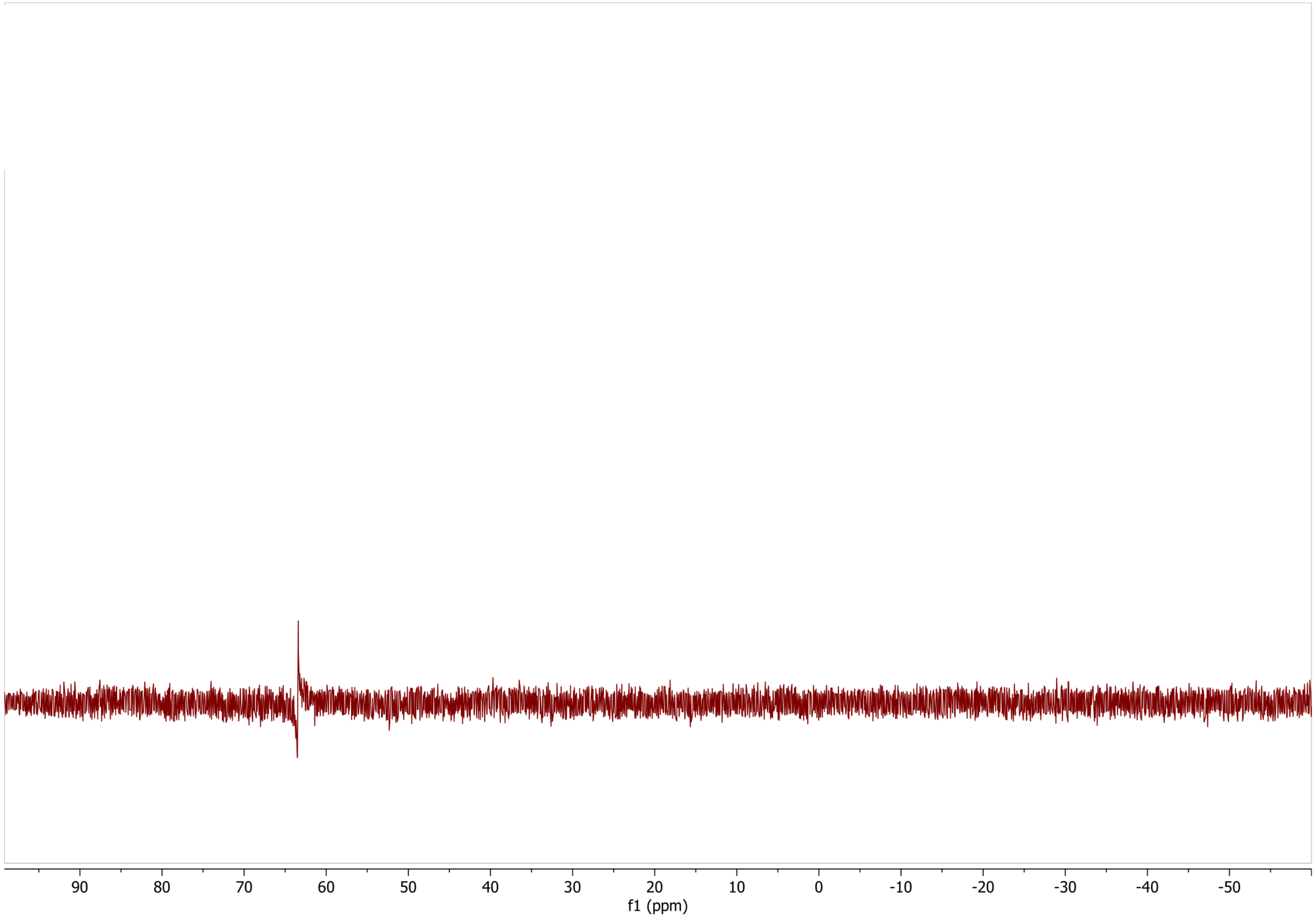

Supplement: Supplementary file 1 — Supplementary Information [file 42004_2024_1327_MOESM1_ESM.pdf]
