# Supplementary material for: Discovery of Trypanosoma brucei inhibitors enabled by a unified synthesis of diverse sulfonyl fluorides
Source: Commun Chem. 2024 Oct 19;7:237. doi: 10.1038/s42004-024-01327-8 (PMC11490619; doi:10.1038/s42004-024-01327-8)
Supplement: Supplementary file 2 — Description of Additional Supplementary Files [file 42004_2024_1327_MOESM2_ESM.pdf]

Description of Additional Supplementary Files

File name: Supplementary Data 1

Description: Primary data plotted in figures in the manuscript

File name: Supplementary Table 5

Description: Chemical proteomics with probe C-1alk. Identification of proteins following labelling with C-1alk, CuAAC, pull-down, tryptic digest and LC-MS/MS analysis using a timsTOF platform.

File name: Supplementary Table 6

Description: Chemical proteomics with probe C-1alk following pre-incubation with C-1 parent compound. Identification of proteins following labelling with C-1alk, CuAAC, pulldown, tryptic digest and LC-MS/MS analysis using a timsTOF platform.

File name: Supplementary Table 7

Description: Chemical proteomics with probe FP-biotin. Identification of proteins following labelling with FP-biotin +/- C-1 parent compound, CuAAC, pull-down, tryptic digest and LCMS/MS analysis using a timsTOF platform.

File name: Supplementary Table 8

Description: Comparison of hits from different datasets. A comparison of hits from Tables S5, S6 and S7
